# Insights into the identification and evolutionary conservation of key genes in the transcriptional circuits of meiosis initiation and commitment in budding yeast

**DOI:** 10.1002/2211-5463.13728

**Published:** 2023-11-14

**Authors:** Deepyaman Das, Anis Ahmad Chaudhary, Mohamed A. M. Ali, Abdullah S. Alawam, Hironmoy Sarkar, Soumita Podder

**Affiliations:** ^1^ Cell Biology and Bacteriology Laboratory, Department of Microbiology Raiganj University India; ^2^ Computational and Systems Biology Laboratory, Department of Microbiology Raiganj University India; ^3^ Department of Biology, College of Science Imam Mohammad Ibn Saud Islamic University (IMSIU) Riyadh Saudi Arabia; ^4^ Department of Biochemistry, Faculty of Science Ain Shams University Cairo Egypt

**Keywords:** evolutionary conservation, gene regulatory network, master regulator, protein–protein interaction network, *Saccharomyces cerevisiae*, transcription factor

## Abstract

Initiation of meiosis in budding yeast does not commit the cells for meiosis. Thus, two distinct signaling cascades may differentially regulate meiosis initiation and commitment in budding yeast. To distinguish between the role of these signaling cascades, we reconstructed protein–protein interaction networks and gene regulatory networks with upregulated genes in meiosis initiation and commitment. Analyzing the integrated networks, we identified four master regulators (MRs) [Ume6p, Msn2p, Met31p, Ino2p], three transcription factors (TFs), and 279 target genes (TGs) unique for meiosis initiation, and three MRs [Ndt80p, Aro80p, Rds2p], 11 TFs, and 948 TGs unique for meiosis commitment. Functional enrichment analysis of these distinct members from the transcriptional cascades for meiosis initiation and commitment revealed that nutritional cues rewire gene expression for initiating meiosis and chromosomal recombination commits cells to meiosis. As meiotic chromosomal recombination is highly conserved in eukaryotes, we compared the evolutionary rate of unique members in the transcriptional cascade of two meiotic phases of *Saccharomyces cerevisiae* with members of the phylum Ascomycota, revealing that the transcriptional cascade governing chromosomal recombination during meiosis commitment has experienced greater purifying selection pressure (*P* value = 0.0013, 0.0382, 0.0448, 0.0369, 0.02967, 0.04937, 0.03046, 0.03357 and < 0.00001 for *Ashbya gossypii*, *Yarrowia lipolytica*, *Debaryomyces hansenii*, *Aspergillus fumigatus*, *Neurospora crassa*, *Kluyveromyces lactis*, *Schizosaccharomyces pombe*, *Schizosaccharomyces cryophilus*, and *Schizosaccharomyces octosporus*, respectively). This study demarcates crucial players driving meiosis initiation and commitment and demonstrates their differential rate of evolution in budding yeast.

## Graphical abstract

To distinguish between the roles of signaling cascades for meiosis initiation and commitment in budding yeast, we reconstructed protein–protein interaction networks and gene regulatory networks with upregulated genes in both phases. Analyzing the integrated networks, we identified and performed functional enrichment of unique interactors in both the phases. Lastly, we compared the evolutionary rate for unique members in transcriptional cascade of two meiotic phases.

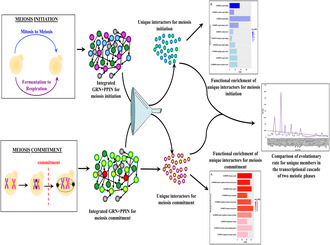

**Abbreviations**
CDS: coding sequence
DEG: differentially expressed gene
GRN: gene regulatory network
MRs: master regulators
PPIN: protein–protein interaction network
RTG: return to growth
SPM: sporulation media
TF: transcription factor
TG: target gene
YPA media: yeast extract‐peptone‐acetate media
YPD media: yeast extract–peptone–dextrose media

Meiotic process is the fundamental mechanism behind formation of viable gametes and production of offspring in eukaryotes [1]. The initiation of meiosis is a tightly regulated process and depending upon the species, a specific stimulus regulates entry into the meiotic divisions [2]. For example, initiation of meiosis during spermatogenesis in mice requires retinoic acid whereas, in *Caenorhabditis elegans*, the meiocytes progress through meiosis as they move away from the distal tip of the gonad [2]. Although meiotic divisions occur only in germ cells of multicellular eukaryotes, but in unicellular eukaryotes like budding yeasts, the decision to initiate meiosis has to be taken by the single cell at a critical stage of the life cycle [3]. Meiosis in budding yeast is initiated whenever a diploid MATa/MATα strain is subjected to both nitrogen and glucose starvation in the sporulation media (SPM) [3]. But initiation of meiosis does not commit the cells towards completion of meiosis [4]. Previous evidences reported that migration of spindle pole bodies toward the opposite poles could commit the cells for both meiosis and mitosis [5, 6]. Later on, return to growth (RTG) experiments conducted by Friedlander *et al*. showed that diploid budding yeasts when transferred from SPM to vegetative growth media within 5–6 h of meiosis, stalled their meiotic process and reverted to mitotic growth phase. So, apart from the signaling cascade which initiates meiosis, there is another distinct signaling pathway operating in the later stages of meiosis that results in separation of spindle pole bodies and thus commits the cells for completion of meiosis [4]. Thus, the signaling cascade for meiosis initiation must be unique compared to commitment.

In budding yeast, *IME1* (Inducer of Meiosis) is the most vital gene to be expressed during meiosis initiation in *Saccharomyces cerevisiae* [7]. The ability to enter meiosis is also controlled by the a1‐α2 repressor encoded by diploid MATa/MATα cells. The a1‐α2 repressor hinders the expression of *RME1* (Repressor of Meiosis 1) that shuts down meiosis induction by repressing expression of *IME1* (Initiator of Meiosis 1) during vegetative phase [7, 8]. Ime1p is a transcriptional activator of early meiosis‐specific genes which needs to be expressed in high amount in order to initiate meiosis [9]. Ume6p is a repressor which generally binds to promoter region of meiosis‐specific genes and prevents meiosis initiation during vegetative phase. Ime1p interacts with Ume6p to initiate transcription of meiosis‐specific genes. The serine threonine kinase Rim11p phosphorylates Ime1p and this allows its interaction with Ume6p. On binding with Ume6p, the transcription activation domain of Ime1p gets exposed and this Rim11p‐Ime1p‐Ume6p complex in turn activates transcription of early meiosis‐specific genes [10]. Whereas, budding yeasts become irreversibly committed to meiosis when they can no longer respond to changes in nitrogen and glucose fluctuations in the media. To achieve this, the budding yeasts deploy two mechanistic processes—firstly they inactivate the glucose signaling pathways in the cells and then separation of spindle pole bodies occur [11]. Ndt80p, which is vital for transcribing middle meiotic genes, was reported to be essential for the diploid cells to exit from pachytene stage of meiotic prophase and thereby committing the cells for meiosis [12, 13]. Again, mutation of spo14, which is responsible for prospore formation, was found to revert cells back to mitotic growth in RTG experiments [11]. This suggested that during commitment, the cells do not inhibit mitotic growth but rather a unique signaling cascade favors completion of meiosis. Thus, there might be two distinct transcriptional cascades for meiosis initiation and commitment.

Hence, we compared the transcriptional cascade obtained by integrating protein–protein interaction network (PPIN) and gene regulatory network (GRN) data for meiosis initiation and commitment. Our analysis reported that a total of 20 and 19 MRs might be controlling the meiosis initiation and commitment phases respectively and by comparing the unique transcriptional cascades from both the phases, we discovered 4 Master Regulators (MRs) [Ume6p, Msn2p, Met31p, and Ino2p], 3 Transcription Factors (TFs) [Rlm1p, Rdr1p, Fzf1p] and 279 Target Genes (TGs) to be unique for meiosis initiation, while 3 MRs [Ndt80p, Aro80p, and Rds2p], 11 TFs [Mss11p, Pdr3p, Rgt1p, Yrr1p, Rme1p, Mig3p, Kar4p, Cat8p, Gzf3p, Ecm22p, Gat3p], 948 TGs are found exclusively for meiosis commitment. Comparison of the functional and pathway enrichment analysis of the unique transcriptional cascades from both the phases revealed that chromosomal segregation might be committing the cells for meiosis. Lastly, our evolutionary gene conservation study of *S. cerevisiae* in phylum Ascomycota reported that transcriptional cascade for meiosis commitment in budding yeasts is more evolutionary conserved compared to meiosis initiation stage.

## Materials and methods

### Data processing to identify differentially expressed genes during meiosis initiation and commitment in *S. cerevisiae*, and spermatogenesis in mice

For identification of differentially expressed genes (DEGs), we considered microarray data GSE75257 [14] and GSE18181 [15] for meiosis initiation and GSE3815 and GSE3816 for meiosis commitment [4] in *S. cerevisiae* from NCBI Gene Expression Omnibus (GEO) database. We used the basic packages of the Bioconductor project based on limma v3.26.8 [16] for retrieval, logarithmic transformation, and quantile normalization of the data. For both the microarray data, we considered adjusted *P* value < 0.05 and |log_2_(FC)| > 1 as cut off.

### Reconstruction of protein–protein interaction network and gene regulatory network

For reconstructing PPIN, we used only physical interactions (viz. experiments and databases) from STRING v11 [17] application programming interface (API) in python v3.9 (Jupyter notebooks by Fernando Pérez and Brian Granger). For reconstructing GRN for budding yeast, we considered experimentally verified TF binding data from YEASTRACT+ [18] and Saccharomyces Genome Database (SGD) [19]. For determining MR in each case, we reconstructed a hierarchical network by integrating TF‐TF, TF‐TG data from respective GRN and TG‐TG data from respective PPIN and then used Network Analyzer in cytoscape v3.8.2 [20, 21] to calculate in‐degrees (in) and out‐degrees (out) for each node in the integrated networks. Then by following a previously devised *in‐silico* protocol [22, 23], we used the in‐degree (in) and out‐degree (out) of the intricate web of interactions in this directed network to create a hierarchical network or pyramidal tree. The following formula is then used to obtain the hierarchy index (*v*) for each node. we calculated hierarchy index (*v*) for all the nodes by using the following formula:

$$v=\left(\text{out}-\text{in}\right)/\left(\text{out}+\text{in}\right).$$

The hierarchy index measures how much the network has a pyramidal structure and ranges from 1 to 1 [24]. We have designated the dominating interactors, or those at the top of the hierarchy, as the network's master regulators. The most highly ranked among the hierarchy indices (*v*) i.e. = 1 were considered as MRs for the phase. All the networks were visualized using cytoscape v3.8.2 [20].

### Functional and pathway enrichment analysis

For GO (Gene Ontology) Biological Process (BP), GO Molecular Function (MF), and KEGG (Kyoto Encyclopedia of Genes and Genomes) pathway analysis, we used clusterprofiler v4.0.0 package [25] in r v4.1.1 and metascape 3.5 (based on code released on Aug 13, 2021) [26]. For clusterprofiler v4.0.0 and metascape 3.5, we considered an adj. *P* value < 0.05 and FDR < 0.05 as cut off, respectively. For GO functional and pathway enrichment analysis, hypergeometric test (one‐tailed variant of Fisher's exact test) was used to identify significant over‐represented GO terms and pathways.

### Evolutionary gene conservation study

For studying conservation of genes involved in two different meiotic phases in *S. cerevisiae*, we have traced orthologous pairs from *Ashbya gossypii*, *Yarrowia lipolytica*, *Aspergillus fumigatus*, *Neurospora crassa*, *Schizosaccharomyces pombe*, *Schizosaccharomyces cryophilus*, *Schizosaccharomyces octosporus*, *Candida albicans*, *Candida tropicalis* and *Candida auris* for both meiosis initiation and commitment genes of budding yeast by considering annotations from Ensembl Fungi Genes 53 in Ensembl fungi [27]. Along with one‐to‐one orthologous type and confidence score = 1, a cut off of sequence identity ≥ 60% for *A. gossypii* and ≥ 30% for rest of the species was considered to determine orthologous pairs. As the ortholog data for *Debaryomyces hansenii* and *Kluyveromyces lactis* was not available in Ensembl fungi [27], so we downloaded peptide sequences for the genes of *D. hansenii* and *K. lactis* from UniProt [28] and performed blastp to compare similarity in peptide sequences with the genes from both meiosis initiation and commitment transcriptional cascades of budding yeast. A cut off of sequence identity ≥ 70% was considered to determine orthologous pairs for both the species. Later on, to deduce evolutionary rate (ω) of these pairs of orthologous genes, we calculated the nonsynonymous to synonymous divergence ratio (dN/dS) using codeml program of PAML (version 4.9) by following Nei and Gojobori 1986 method [29, 30]. For this, initially we downloaded the coding sequence (CDS) of the genes for *A. gossypii*, *Y. lipolytica*, *Asp. fumigatus*, *N. crassa*, *Sc. pombe*, *Sc. cryophilus*, *Sc. octosporus*, *C. albicans*, *C. tropicalis*, and *C. auris* from Ensembl Fungi (version‐53) [27]. Whereas, the CDS of the genes for *D. hansenii* and *K. lactis* were downloaded using Entrez Direct (EDirect) [31]. To calculate dN/dS using codeml, we initially aligned the peptide sequences using clustal omega [32] and then a codon‐based nucleic acid alignment was retrieved by using PAL2NAL [33]. For each of the resulting alignments, we calculated dN/dS by using codeml program [30, 34]. Whereas, for mega v11 [35] we aligned the peptide sequences for the pair of orthologous genes using clustal w [36] and then performed “Codon‐based *Z*‐test for selection” for negative selection to identify significance of purifying selection for each pair by considering a Prob. value < 0.05 as cut off. Lastly, to remove outliers during evolutionary rate comparison between the phases, Rosner's generalized extreme Studentized deviate test (*P* value < 0.05 as cut off).

### Statistical tests and graphical plots

All the statistical tests were performed in r v4.1.1, and the graphical plots were generated in r v4.1., python v3.9 [37] and cytoscape 3.8.2 [20].

## Results

### Identification of master regulators that control a signaling cascade for initiating meiosis in *S. cerevisiae*

**Fig. 1:**
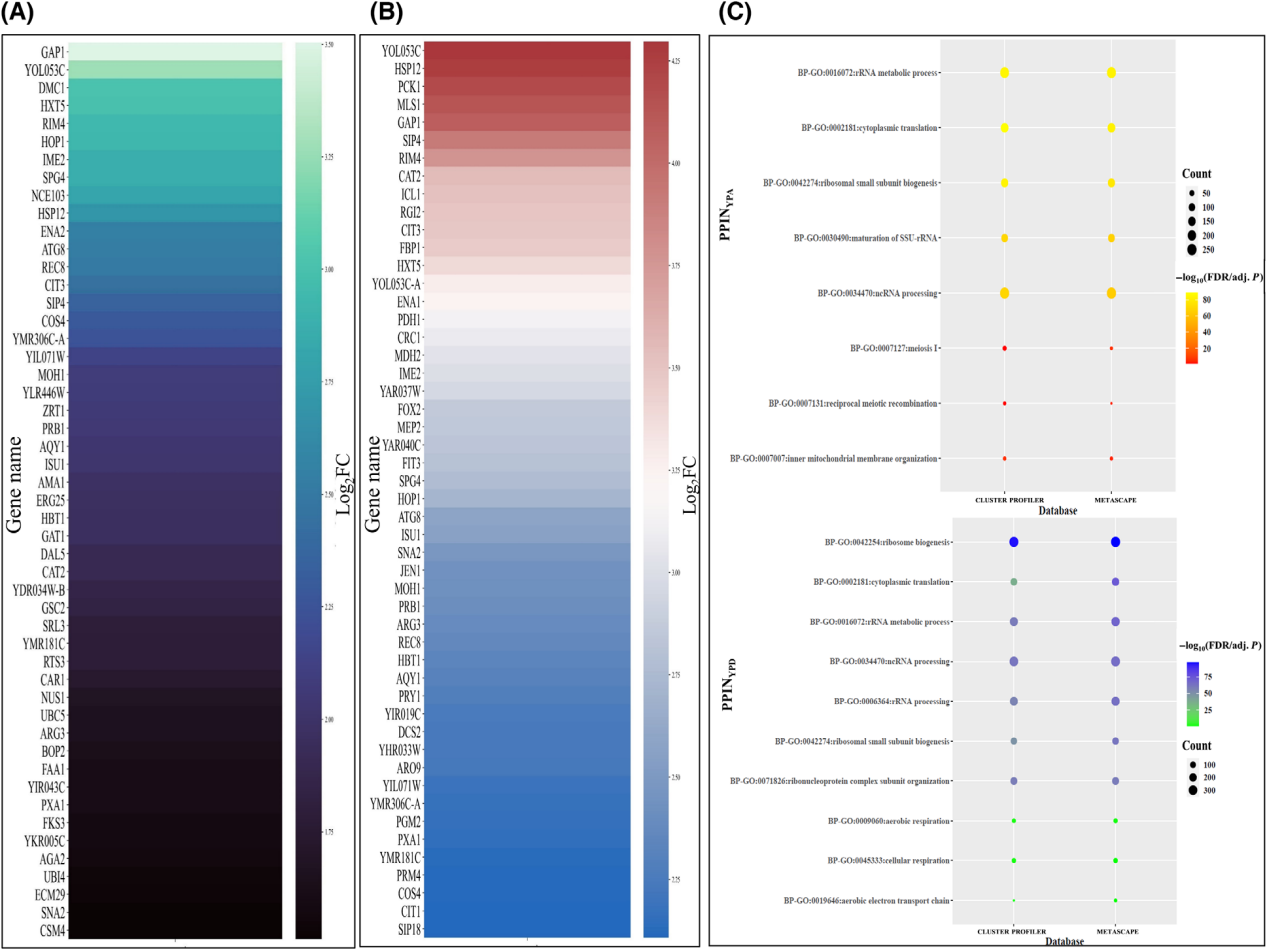
Heatmap of upregulated genes during meiosis initiation and functional enrichment of the PPIN for meiosis initiation (A) Heatmap showing expression (Log_2_FC) of top 50 significantly upregulated genes after 1.5 h in SPM when compared to YPA media. (B) Heatmap showing expression (Log_2_FC) of top 50 significantly upregulated genes after 1.5 h in SPM when compared to YPD media. (C) GO Biological Process enrichment of PPIN_YPA_ and PPIN_YPD_.

**Fig. 2:**
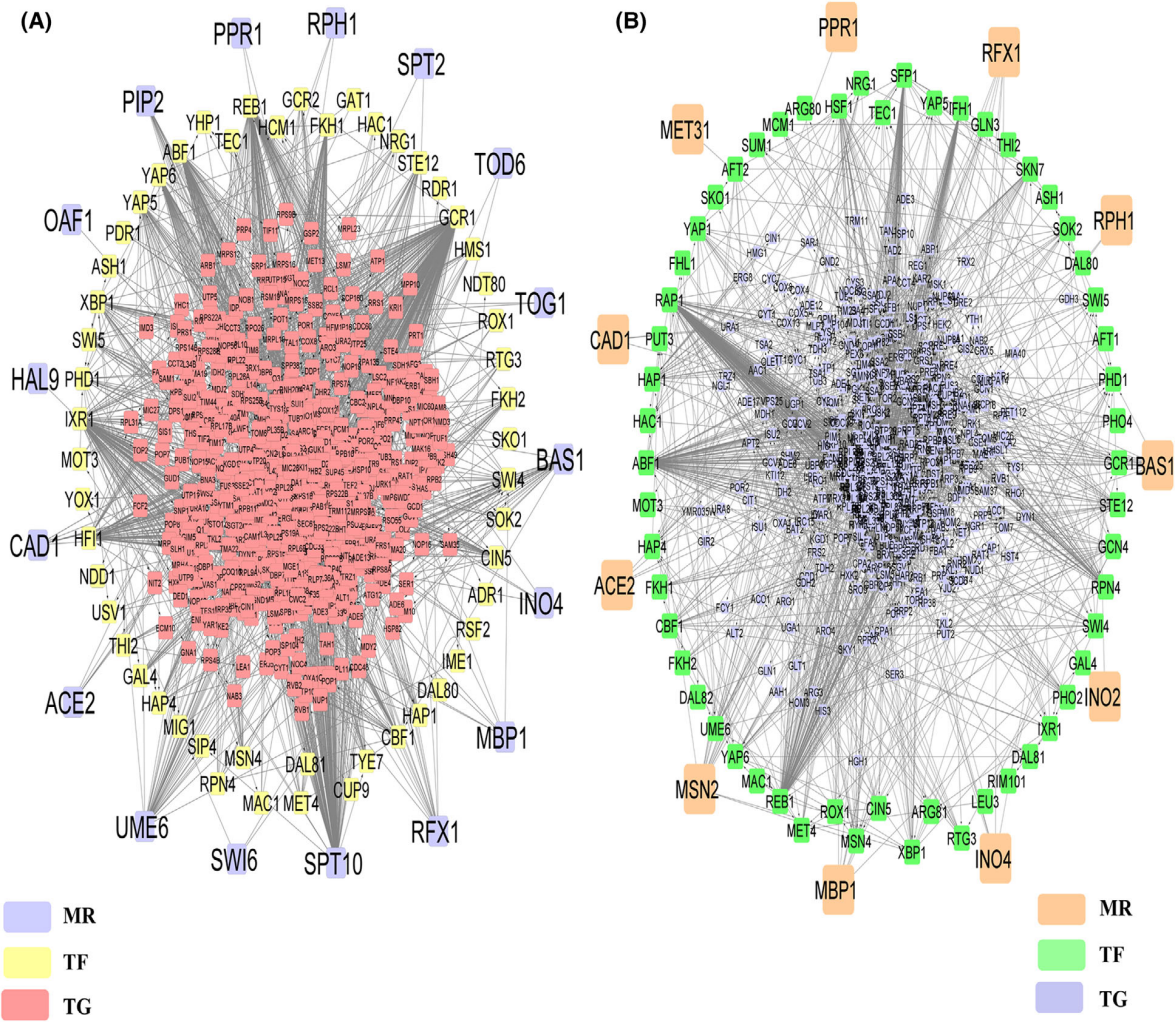
Hierarchical cascade of MRs for meiosis initiation. The network is built in the form hierarchical structure with TFs with highest *v* value (i.e., MRs) at the periphery followed by rest TFs and TGs form the centre. (A) Hierarchical cascade obtained by integrating PPIN_YPA_ and GRN_YPA_. The network consists of 863 nodes and 24 853 edges (B) Hierarchical cascade obtained by integrating PPIN_YPD_ and GRN_YPD_. The network consists of 540 nodes and 9386 edges.

Meiosis in *S. cerevisiae* is initiated by a synchronized signaling cascade [38] with the genes responsible for meiosis initiation being strongly upregulated within 1.5 h of growth in SPM [15]. Thus, initially, we have screened significantly upregulated genes from two datasets—GSE75257 [14] and GSE18181 [15] by comparing transcriptomic data of diploid cells grown in YPA media and 1.5 h in SPM and identified 81 common upregulated genes between these two datasets (Fig. 1A; File S1). Similarly, from both these datasets, we also identified 162 common upregulated genes by comparing cells grown in YPD media and 1.5 h in SPM (Fig. 1B; File S1). With these two sets of DEGs, we have reconstructed two PPIN‐PPIN_YPA_ and PPIN_YPD_ which are dedicated for meiosis initiation stage. Here, we have made two individual networks for studying meiosis initiation as changing the control variable by keeping experimental condition intact could skew the results [39]. For both scenarios, the control variable changes, that is, the cells are either grown in YPD media or YPA media, so combining these two sets of upregulated genes could be erroneous. The PPIN_YPA_ consists of 1176 nodes and 35 525 edges whereas, PPIN_YPD_ consists of 1735 nodes and 36 932 edges (Figs S1 and S2). Subsequently, we have performed functional enrichment analyses of interacting proteins in PPIN_YPA_ and PPIN_YPD_ using two databases—metascape and clusterprofiler [25, 26]. Common biological processes of these two databases were considered and we have identified 224 and 345 GO: Biological Processes for PPIN_YPA_ and PPIN_YPD_, respectively. Among the GO functions, translation specific biological processes (BPs) (GO:0006413, GO:0002183, GO:0002181) along with meiotic cell cycle‐specific BPs (GO:0051321, GO:1903046) were found to be enriched in PPIN_YPA_ (Fig. 1C; File S2). Whereas, PPIN_YPD_ (Fig. 1C; File S2) showed enrichment of aerobic respiration‐specific GO BPs (GO:0009060, GO:0045333, GO:0019646). Now, it is conceivable that during the meiosis initiation, a signaling cascade is instigated which brings about a surge in protein expression of meiosis‐specific genes [40]. Also, the aerobic respiratory process and meiosis are so interlinked that many genes have already been proven to be required simultaneously for both the processes [41]. From functional enrichment results, we have retrieved 930 and 1296 meiosis‐related genes for PPIN_YPA_ and PPIN_YPD_, respectively. Since these genes might be involved in various signaling pathways during the meiosis initiation, we intended to study the regulation of these crucial genes. To do this, we have reconstructed two gene regulatory networks: (GRNs)‐GRN_YPA_ (Fig. S3) and GRN_YPD_ (Fig. S4), with the TFs of these genes, retrieved from experimental TF‐binding data of SGD and YEASTRACT+ [18, 19]. Combining the data from the both databases, we found that 114 TFs that might be controlling the expression of 754 TGs in GRN_YPA_ (Fig. S3) and 69 TFs might be controlling expression of 479 TGs in GRN_YPD_ (Fig. S4). As the TFs that regulate more than the average number of TGs regulated by TFs in these GRNs might be most important in driving the phases, so we have calculated the mean of total number of TGs regulated by each TF and considered it as cut off for determining the vital TFs for the phases [22]. Accordingly, by considering a cut off > 11 (for GRN_YPD_) and > 27 (for GRN_YPA_), we received top 12 TFs for GRN_YPD_ and 22 TFs for GRN_YPA_. Next, we wanted to determine MRs that might be regulating transcriptional hierarchy during meiosis initiation. So, by integrating PPIN and GRN data (PPIN_YPA_, PPIN_YPD_, GRN_YPA_, and GRN_YPD_), we collected three sets of interactions: TF‐TF, TF‐TG, and TG‐TG interactions. Then by following a protocol described in our earlier study [22], we identified 17 and 11 MRs that might initiate meiosis when diploid budding yeast are transferred from YPA and YPD media, respectively, to SPM (Fig. 2A,B). Taken together, we could conclude that 27 common TFs, which has been obtained by combining the top 12 TFs from GRN_YPD_ and top 22 TFs from GRN_YPA_ might be most important for meiosis initiation (Table 1; Figs S3 and S4). Also, 20 TFs, which has been obtained by combining 17 and 11 TFs from GRN_YPD_ and GRN_YPA_, respectively, might be acting as MRs for initiating a transcriptional cascade during the meiosis initiation process (Fig. 2).

**Table 1:** Top TFs with their no. of TGs involved in regulatory networks of meiosis initiation and commitment. The top TFs acting as MRs and their mutant phenotype for sporulation has been specified. # represents no TGs in the respective network.

TF	MR	Meiosis initiation		Meiosis commitment	Phenotype	References
		TGs in GRN_YPA_	TG in GRN_YPD_	TG in GRN_commit_		
Ste12	No	####	18	47	Abnormal sporulation	PMID:14752166
Yap5	No	29	####	77	Abnormal sporulation	PMID:14752166
Sfp1	No	419	49	653	Sporulation absent	PMID:22384326
Tup1	No	56	####	101	Sporulation absent	PMID:12586695
Ume6	Initiation	36	####	####	Sporulation absent	PMID:12586695
Spt10	Initiation and commitment	159	####	194	Sporulation decreased	PMID:12586695
Spt3	No	####	####	48	Sporulation decreased	PMID:12586695
Xbp1	No	30	####	104	Sporulation decreased	PMID:10611226
Abf1	No	70	78	127	####	####
Bas1	Initiation and commitment	####	12	####	####	####
Cbf1	No	####	####	88	####	####
Cin5	No	####	####	46	####	####
Cst6	No	215	####	255	####	####
Fhl1	No	87	####	112	####	####
Fkh1	No	56	####	140	####	####
Fkh2	No	####	####	47	####	####
Gcn4	No	37	####	62	####	####
Gcr1	No	225	####	288	####	####
Hap1	No	####	17	####	####	####
Hfi1	No	60	####	71	####	####
Hsf1	No	168	29	199	####	####
Ifh1	No	114	44	111	####	####
Ixr1	No	117	####	265	####	####
Mbp1	Initiation and commitment	####	####	46	####	####
Met32	No	61	####	113	####	####
Msn2	Initiation	44	14	79	####	####
Rap1	No	215	131	314	####	####
Reb1	No	116	93	201	####	####
Rpn4	No	####	34	55	####	####
Skn7	No	####	13	####	####	####
Yap1	No	93	####	205	####	####
Yap6	No	33	####	64	####	####

### Identification of master regulators that initiates a signaling cascade for committing the cells for meiosis in *S. cerevisiae*

**Fig. 3:**
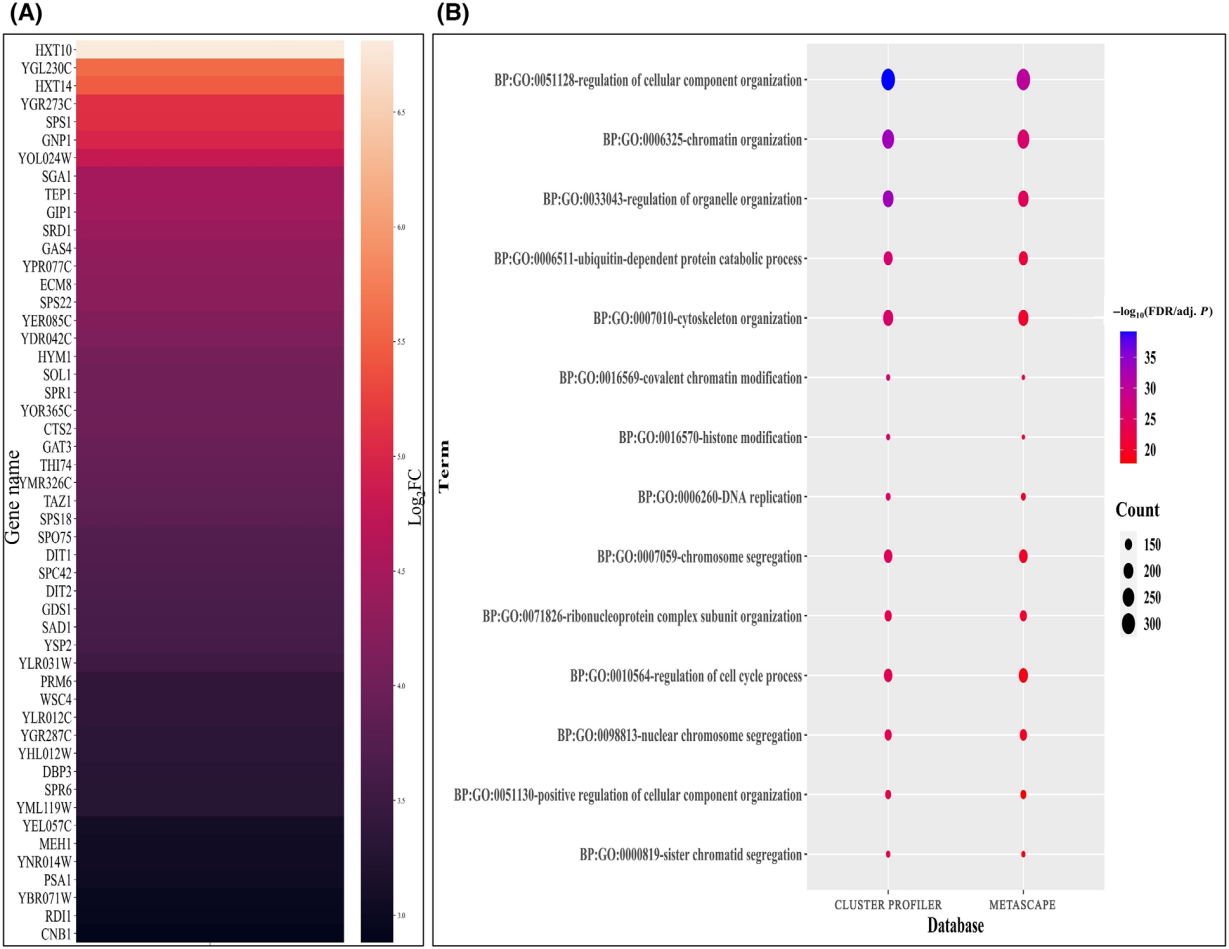
Heatmap of upregulated genes during meiosis commitment and functional enrichment of the PPIN for meiosis commitment (A) Heatmap showing expression (Log_2_FC) of top 50 significantly upregulated genes during meiosis commitment phase (B) GO Biological Process enrichment of PPIN_commit_.

**Fig. 4:**
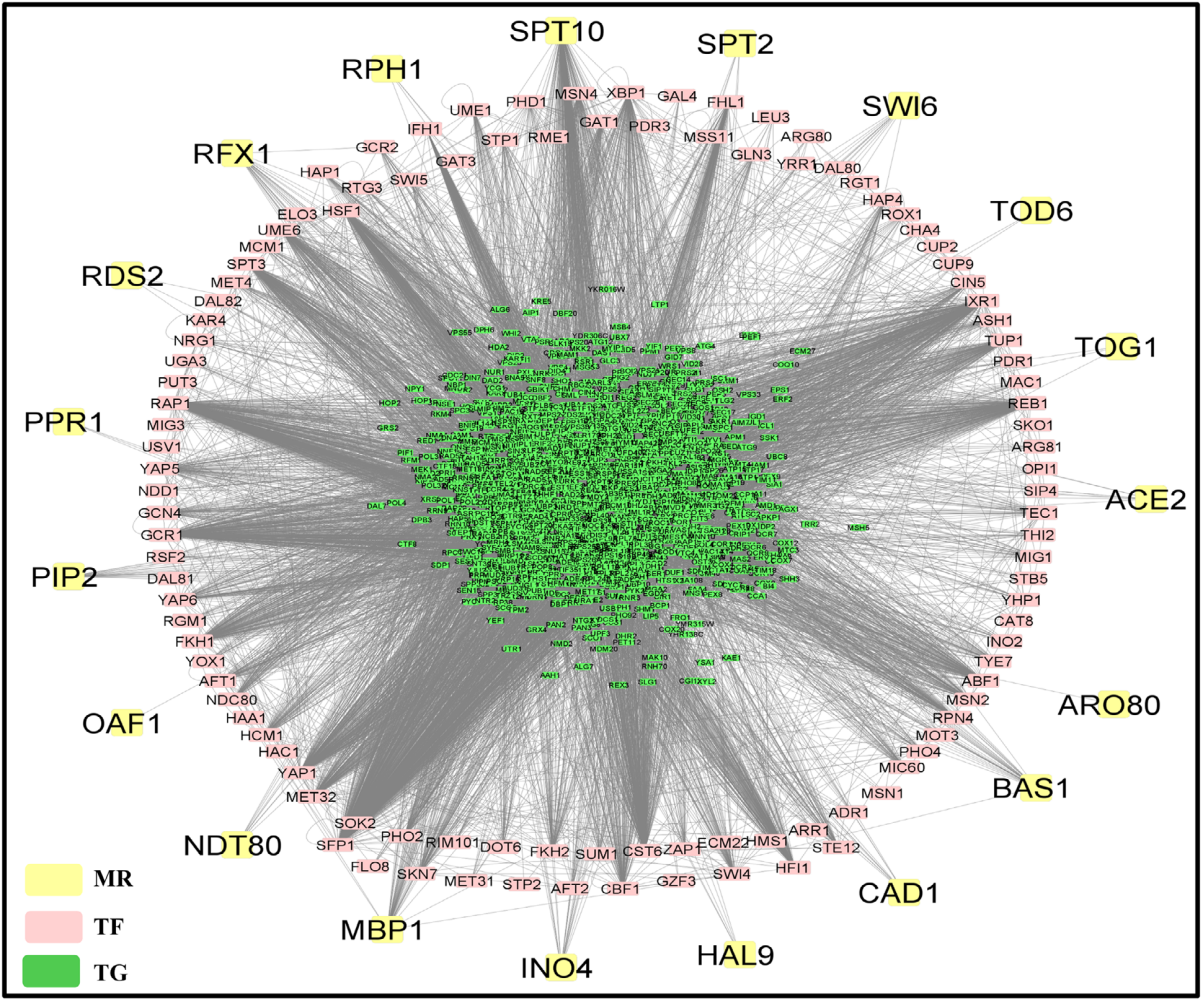
Hierarchical cascade of MRs for meiosis commitment. The network is built in the form hierarchical structure with TFs with highest *v* value (i.e., MRs) at the periphery followed by rest TFs and TGs form the centre. This hierarchical cascade has been obtained by integrating PPIN_commit_ and GRN_commit_. The network consists of 1660 nodes and 54 853 edges.

Similar to meiosis initiation, we aimed to investigate the gene pool involved in the signaling cascade that might be instigating meiosis commitment. For this, we have analyzed microarray data available in GSE3815 [4] and retrieved 566 upregulated DEGs by comparing non‐committed stage (2, 3 and 4 h of growth in SPM) with committed stage (5, 6 and 7 h of growth in SPM) of meiosis (File S3). Now, it could be assumed that among upregulated DEGs in committed cells that are being downregulated after transferring to YPD, are the genes that are responsible for meiosis commitment. So, for this, we compared transcriptomic data available in GSE3816 [4] of cells grown for 5, 6 and 7 h in SPM with cells transferred at 5, 6 and 7 h of sporulation to YPD and harvested for 40 min after the transfer. We retrieved a total of 689 genes that are being downregulated on transferring committed cells to YPD. After mapping these downregulated genes among 566 upregulated DEGs at commitment stage, a total of 156 genes were retrieved to be among the 566 gene list. So, after eliminating these 156 genes, we got 410 genes for further downstream analysis (Fig. 3A; File S3). Using these genes, we reconstructed a PPIN_commit_ consisting of 2627 nodes and 39 406 edges (Fig. S5). Functional enrichment analysis of this PPIN in clusterprofiler and metascape showed 565 common GO BPs are enriched with functions like‐ “regulation of cellular component organization” (GO:0051128), “chromatin organization” (GO:0006325) and “sister chromatid segregation” (GO:0000819) (Fig. 3B; File S4). We identified a total of 2101 functionally enriched genes and then by following a similar *in‐silico* protocol that we had used in the earlier section for unraveling TFs for meiosis initiation phase, we unraveled the regulators that might be controlling gene expression during meiosis commitment. So, by combining the experimental TF‐binding data from SGD and YEASTRACT+, we reconstructed GRN_commit_ (Fig. S6), which consists of 1573 TGs targeted by 126 TFs. Similar to meiosis initiation, here also we obtained 28 top TFs by considering mean of number of TGs across TFs as cut off (Table 1). Next, by integrating this PPIN and GRN data for commitment to meiosis, we generated three sets of interactions: TF‐TF, TF‐TG, and TG‐TG, and calculated MRs of the integrated network. We have identified a total of 19 MRs that might be initiating a signaling cascade for committing the cells to meiosis (Fig. 4).

### Exploring transcriptional cascade unique to meiosis initiation and meiosis commitment phase

**Fig. 5:**
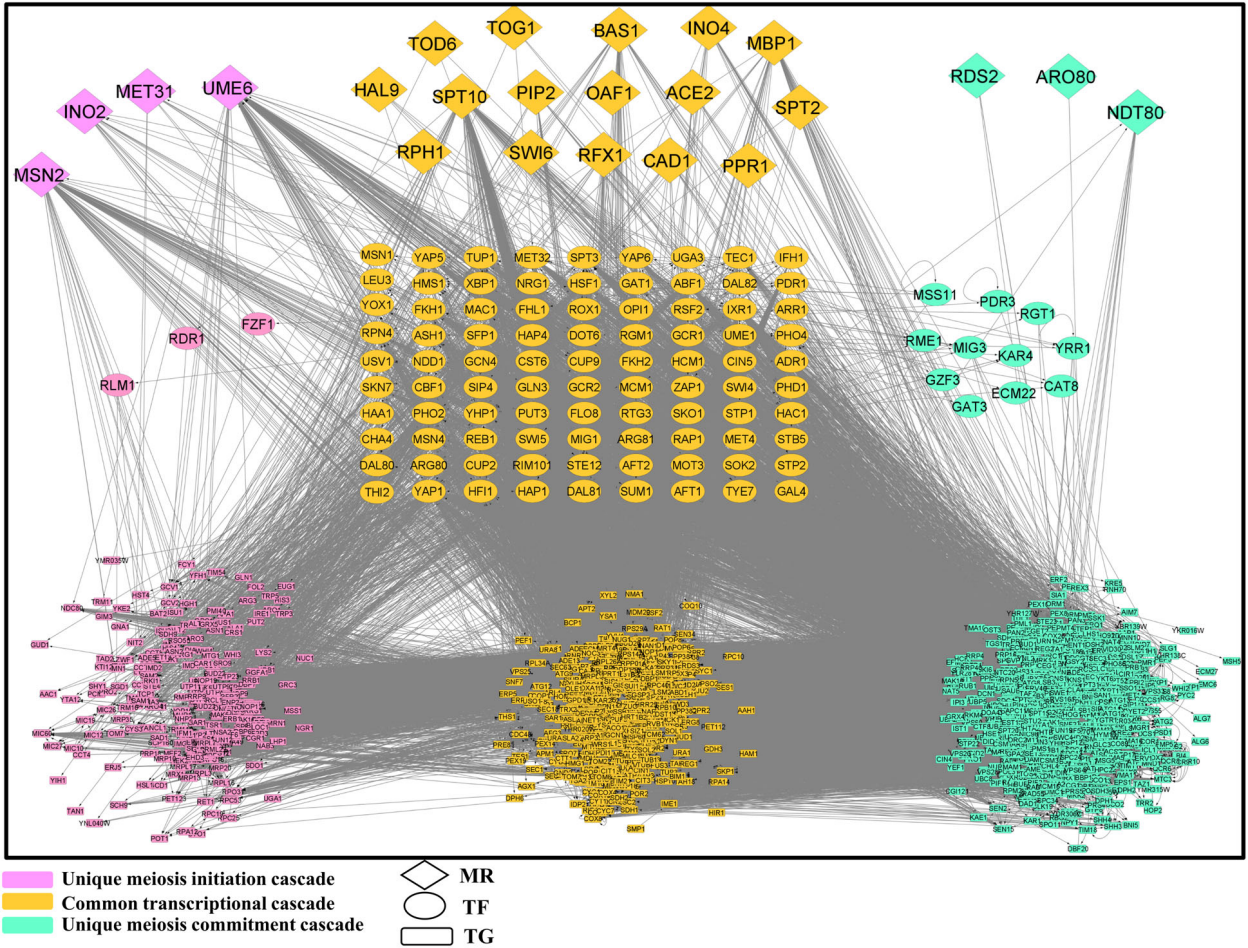
Comparison of the transcriptional cascades for meiosis initiation and commitment. The unique transcriptional cascade for meiosis initiation and commitment is on the left‐ and right‐hand side of the figure, respectively. The common transcriptional cascade for both the phases is in the middle. The network consists of 1963 nodes and 67 711 edges.

**Fig. 6:**
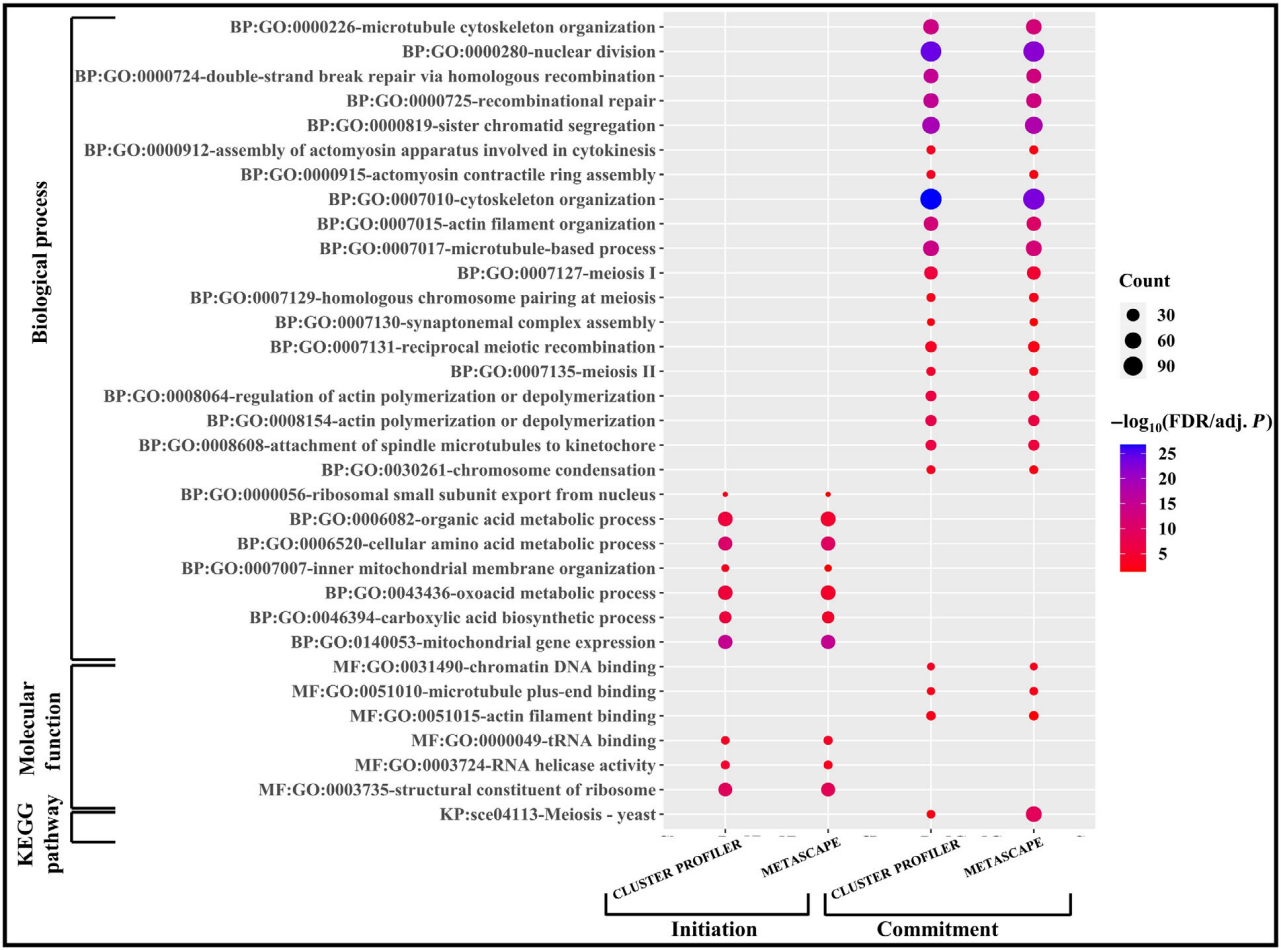
Comparison of functional and pathway enrichment between the distinct transcriptional cascades of meiosis initiation and commitment. The figure depicts comparison of the enrichment of important GO Biological Processes, GO Molecular Functions and KEGG pathways of the distinct transcriptional cascades for meiosis initiation and commitment.

In earlier sections, we have showed that there are 27 TFs which target a large pool of genes for meiosis initiation and 20 TFs might act as MRs of the regulatory network. While we have received 28 TFs and 19 MRs for meiosis commitment phase. Comparing the regulatory elements of these two phases we have noticed that out of 28 TFs for meiosis commitment, 24 TFs are also involved in meiosis initiation. Moreover, 16 MRs are common between meiosis initiation and commitment phase. It is quite obvious that the gene expression pattern during meiosis initiation and commitment follows a distinct pattern with certain pathways being unique to the particular phase [4]. Thus, we were interested to compare the distinctive molecular cascade for meiosis initiation and commitment to explore the molecular pathways that are unique to both of these phases. For this, we have screened out unique transcriptional cascade for meiosis initiation and commitment from the integrated PPIN and GRN data. Our analysis showed that 4 MRs and 3 TFs are unique to meiosis initiation. Whereas 3 MRs and 11 TFs are unique to meiosis commitment (Fig. 5). However, a plethora of TGs, that is, 279 and 948 TGs are unique for meiosis initiation and commitment, respectively. So, the unique transcriptional cascades for meiosis initiation and commitment contain 286 (4 MRs, 3 TFs, 279 TGs) and 962 (3 MRs, 11 TFs and 948 TGs) genes, respectively. Then, to get insights into transcriptional pathways that are unique to both of the phases, we have performed functional and pathway enrichment analysis of these 286 and 962 unique members from transcriptional cascade of meiosis initiation and commitment using clusterprofiler and metascape. Literary evidences suggested that commitment to meiosis is attained when spindle pole body migrate to opposite poles [5]. Our functional enrichment results also echoed a similar fact (Fig. 6; File S5) i.e., “attachment of spindle microtubules to kinetochore” (GO:0008608), “cytoskeleton organization” (GO:0007010) were enriched biological processes for the unique transcriptional cascade of meiosis commitment. Also, GO Molecular functions (MFs)—“chromatin DNA binding” (GO:0031490), “microtubule plus‐end binding” (GO:0051015) and KEGG pathways like—“Meiosis – yeast” (sce04113) were found to be enriched. Thus, it could be stated that meiosis commitment could be triggered by the interplay of 962 genes which might induce microtubule movement during chromosomal segregation and thereby commit the cells for meiosis. Previously, it was documented that meiosis initiation is commenced by a surge in synthesis of new proteins that will be required for the meiotic process [40], and during this phase, nutrient starvation instigates expression of genes that reprogram the vital metabolic pathways [41]. In our study, we have found that 279 meiosis initiating genes are enriched with GO BPs—“ribosomal small subunit export from nucleus” (GO:0000056), “mitochondrial gene expression” (GO:0140053) and “carboxylic acid biosynthetic process” (GO:0046394) and GO: MFs “structural constituent of ribosome” (GO:0003735), “tRNA binding” (GO:0000049) (Fig. 6; File S5). Thus, functional enrichment analysis confirmed that the genes have the potential to instigate meiosis phase.

### Investigating evolutionary rate of unique transcriptional cascade for meiosis initiation and commitment

Our results show that initiation of microtubular movements for chromosomal segregation commits the cells for meiosis. It is already evidenced that the process of chromosomal segregation and recombination during meiosis of budding yeast is highly conserved among eukaryotes [42]. Since a highly conserved mechanisms segregates duplicated chromosomes evenly to daughter cells at the metaphase–anaphase transition in budding yeasts, so we hypothesized that the unique transcriptional cascade regulating meiosis commitment might be more evolutionary conserved compared to the meiosis initiation phase. Accordingly, we compared the evolutionary rate of the unique transcriptional cascade for meiosis initiation and commitment.

**Fig. 7:**
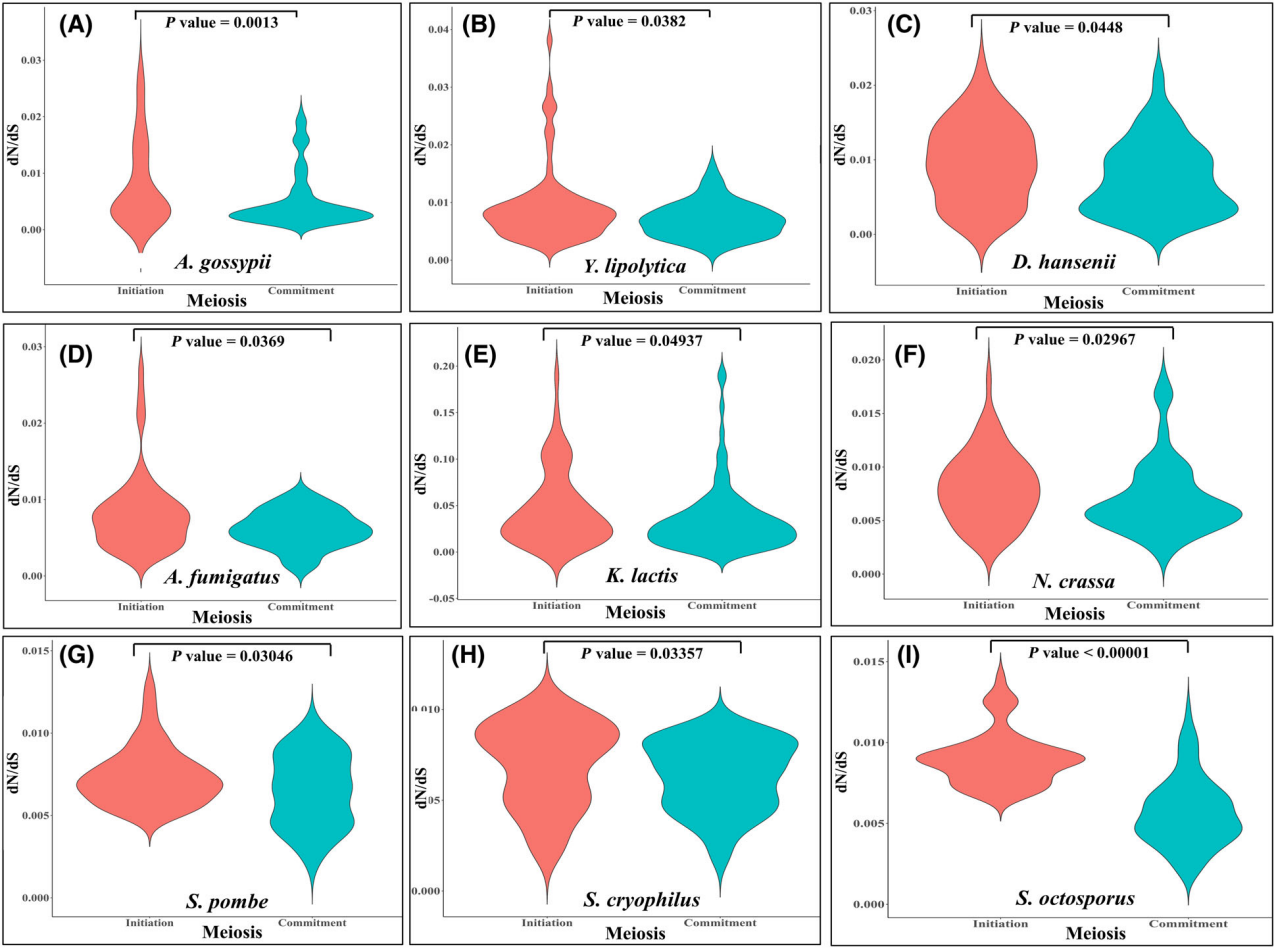
Violin plot comparing the evolutionary rate of unique transcriptional cascades of meiosis initiation and commitment in *Saccharomyces cerevisiae* with nine species from phylum Ascomycota. Evolutionary rate differences between unique transcriptional cascades of (A) *Ashbya gossypii* meiosis initiation (*n* = 77) and commitment (*n* = 148) (B) *Yarrowia lipolytica* meiosis initiation (*n* = 78) and commitment (*n* = 112) (C) *Debaryomyces hansenii* meiosis initiation (*n* = 42) and commitment (*n* = 78) (D) *Aspergillus fumigatus* meiosis initiation (*n* = 58) and commitment (*n* = 87) (E) *Kluyveromyces lactis* meiosis initiation (*n* = 60) and commitment (*n* = 95) (F) *Neurospora crassa* meiosis initiation (*n* = 60) and commitment (*n* = 83) (G) *Schizosaccharomyces pombe* meiosis initiation (*n* = 60) and commitment (*n* = 92) (H) *Schizosaccharomyces cryophilus* meiosis initiation (*n* = 64) and commitment (*n* = 80) (I) *Schizosaccharomyces octosporus* meiosis initiation (*n* = 52) and commitment (*n* = 86).

For our gene conservation study we considered 12 species of fungi, namely—*A. gossypii*, *D. hansenii*, *Y. lipolytica*, *Asp. fumigatus*, *N. crassa*, *K. lactis*, *Sc. pombe*, *Sc. cryophilus*, *Sc. octosporus*, *C. albicans*, *C. tropicalis*, and *C. auris*, all of which belong to the same phylum of *S. cerevisiae*, that is, Ascomycota [43, 44]. So, all these species can serve as a good model for studying evolutionary gene conservation of *S. cerevisiae*. Initially, we have traced orthologous pairs for both the phases (Table 2). Then, we have calculated their nonsynonymous to synonymous substitution ratio (ω = dN/dS) using codeml function of PAML v4.0 [45]. It is known that the genes having this ratio lower than one (ω < 1), are undergoing purifying selection, while ω = 1 and ω > 1 indicate neutral evolution, and positive selection, respectively [46]. Strikingly, the orthologous pairs in both the phases, which have significant dN/dS ratio (MEGA *P* value < 0.05), have ω < 1, that is, all of these genes are undergoing purifying selection. Then, for comparing the average dN/dS values of the orthologous pairs between both the phases, initially we removed outliers from the data by performing Rosner's generalized extreme Studentized deviate test (*P* value < 0.05 as cut off) and then we also removed noticeable outliers in specific cases where the sequence identity is not correlated with the dN/dS values. Among the MRs for meiosis initiation, we found *MSN2* in *D. hansenii* and *K. lactis* with ω values—0.0086 and 0.0687 respectively. Similarly, for meiosis commitment, we found *RDS2* as a MR in *D. hansenii* (ω: 0.0044) and *Y. lipolytica* (ω: 0.0062). Except for *K. lactis*, both *MSN2* and *RDS2* had ω values less than the median of the species concerned (median ω for meiosis initiation and commitment for *D. hansenii =* 0.00925 and 0.0067, respectively; median ω for meiosis commitment in *Y. lipolytica* = 0.00695). This depicts that these two MRs of the transcriptional cascades are more evolutionarily conserved. Conspicuously, on comparing the evolutionary rate of the genes, we found that there is significant difference between dN/dS ratio of unique transcriptional cascade for meiosis initiation and commitment respectively in all species except *Candida* spp. (Fig. 7; File S6). Also, the unique transcriptional cascade for meiosis commitment has a significantly higher purifying selection pressure compared to meiosis initiation phase in the phylum Ascomycota except the *Candida* spp. (Fig. 7; File S6). There was no significant difference (Mann–Whitney *U* test *P* value = 0.1816, 0.07417 and 0.0738 for *C. albicans*, *C. tropicalis* and *C. auris* respectively) between dN/dS ratios of unique transcriptional cascade for meiosis initiation (*n* = 92/ω = 0.023, *n* = 91/ω = 0.027 and *n* = 21/ω = 0.020 for *C. albicans*, *C. tropicalis* and *C. auris* respectively) and commitment (*n* = 173/ω = 0.019, *n* = 182/ω = 0.021 and *n* = 37/ω = 0.039 for *C. albicans*, *C. tropicalis* and *C. auris* respectively) in all the *Candida* spp. *Candida* spp. undergoes a parasexual cycle instead of meiosis and this might be the reason behind no significant difference in dN/dS ratio between the phases [44]. So, our results justify the fact that the distinct transcriptional cascade for meiosis commitment which culminates in chromosomal segregation is more evolutionary conserved than meiosis initiation phase in budding yeast.

**Table 2:** Number of orthologs for *Saccharomyces cerevisiae* in 12 species from phylum Ascomycota.

Species	Meiosis initiation	Meiosis commitment
*S. cerevisiae–A. gossypii*	113	204
*S. cerevisiae–D. hansenii*	99	160
*S. cerevisiae–Y. lipolytica*	54	86
*S. cerevisiae–Asp. fumigatus*	80	119
*S. cerevisiae–N. crassa*	91	117
*S. cerevisiae–K. lactis*	125	198
*S. cerevisiae–Sc. pombe*	107	151
*S. cerevisiae–Sc. cryophilus*	105	144
*S. cerevisiae–Sc. octosporus*	102	145
*S. cerevisiae–C. albicans*	140	256
*S. cerevisiae–C. tropicalis*	134	251
*S. cerevisiae–C. auris*	29	52

## Discussion

The diploid MATa/MATα cells can initiate meiotic division if they are starved for nitrogen and glucose in SPM [47]. The sporulation process is initiated only after 1.5 h of growth in SPM [15] when early meiotic genes like *IME1*, *RAD51* are strongly upregulated. Meiosis initiation does not commit the cells to meiosis completion [4]. Experiments performed by Friedlander *et al*. [4] reported that diploid cells are not capable of reverting to vegetative growth after 5–6 h of growth in SPM even if suitable mitotic growth conditions (YPD) are provided. Thus, the signaling cascades for initiating meiosis and committing for meiosis the cells must be different.

Accordingly, for meiosis initiation, enrichment of genes for protein translational machinery, metabolic, and meiotic processes depicted previously reported finding that the starvation for fermentable sugar and nitrogen causes expression of genes that reorient metabolic pathways during meiosis initiation [41]. Whereas, the genes for chromosomal segregation were enriched in commitment stage of meiosis. Our comparative study for the regulatory network governing both the phases (initiation and commitment) of budding yeast also portraits a similar picture. For meiosis initiation, we found Ume6p and Msn2p not only among the top TFs but also as unique MRs. Ume6p, which is found to regulate 38 TGs and 6 TFs, was reported to activate transcription of *IME1* for meiosis initiation [48]. Also, Msn2p and Msn4p together promote upstream activating sequence (UAS) to activate transcription of *IME1* in presence of acetate [49]. Although, Ime1p is the master regulator of meiosis initiation [50] but Ume6p controls the expression of Ime1p, so Ime1p was not reported in our findings. Whereas, Spt10p was found to be a common MR and top TF for both meiosis initiation and commitment (Table 1). Deletion of both ume6 and spt10 were found to halt sporulation [51]. Thus, this phenotypic data of mutants showed that Ume6p and Spt10p must be of primary importance for further studies on meiotic process in budding yeasts. Other top TFs and their mutant's sporulation phenotype has been delineated in Table 1. Coincidently, among the MRs for meiosis commitment we found Swi6p might control expression of 12 TGs. Swi6p was reported to regulate meiotic recombination events [52]. Similarly, we identified Ndt80p as an unique MR and this gene was reported to commit cells for meiosis by mediating exit from meiotic prophase [12]. Among the top TFs for meiosis commitment and initiation we found Sfp1p to regulate significant number of TGs in GRN_YPD_, GRN_YPA_ as well as GRN_commit_ (Table 1). Strikingly, mutation studies reported that diploid cells enter the sporulation pathway but remain blocked in the meiotic prophase on deletion of both Ndt80 and Sfp1 [51, 53]. Lastly, our functional enrichment analysis of the distinctive signaling cascade for meiosis initiation and commitment confirmed our results that although meiosis initiation brings about a radical change in gene expression in response nutritional cues, but commitment is achieved when the microtubules from the divided spindle bodies begin chromosomal segregation.

The meiotic process, specially the events resulting in chromosomal segregation, recombination and double stranded break (DSB) repair, are tightly regulated and irreversible [42, 54, 55]. Moreover, chromosomal segregation and recombination is critical for maintaining genetic and phenotypic variability in a species, so this process is evolutionary conserved in eukaryotes including *S. cerevisiae* [42, 54]. Since, functional enrichment of the unique transcriptional cascade for meiosis commitment depicted that chromosomal segregation events are decisive for committing cells to meiosis, so we noticed that dN/dS ratios of the unique players regulating both the phases proved that the transcriptional cascade governing meiosis commitment is more evolutionary conserved. Among the orthologous pairs, we found important evolutionary conserved interactors, like—*DMC1* and *SMC2*, that mediate the meiotic recombination process not only in budding yeast but also in other eukaryotes. Dmc1p mediates strand exchange and homology search by forming nucleoprotein filaments on single stranded DNA in budding yeasts [56]. Similarly in *A. gossypii*, *dmc1* has similar roles in meiotic recombination events and also the rate of sporulation in *dmc1* mutants decreases in this species [57]. Dmc1p, which exhibits RecA like strand exchange activity, was earlier reported to be evolutionary conserved between *D. hansenii*, *Sc. pombe* and *S. cerevisiae* but not in *Y. lipolytica* [58, 59]. Coincidently, in our study, we found that *DMC1* has orthologs DEHA2E16742p (RefSeq ID: XM_460030.1) and *dmc1* in *D. hansenii* and *S. pombe*, respectively. On the other hand, Smc2p belongs to structural maintenance of chromosomes (SMC) family which is important for chromosomal condensation and mediating chromosomal segregation along with Smc1p during meiosis in budding yeasts [60]. Whereas, in *A. gossypii*, AGOS_AGR236W, which has earlier been documented to have conserved regions of eukaryotic SMC ATPases [61], was found to be the ortholog for *SMC2* of budding yeast. We also found the orthologs YALI0_F24783g, SPOG_02952, SOCG_03338 and *cut14* for *SMC2* in *Y. lipolytica*, *Sc. cryophilus*, *Sc. octosporus*, and *Sc. pombe*, respectively in our study, which belongs to SMC family of proteins for the species [62, 63]. Thus, our study shows that budding yeast cells are committed to meiosis when microtubular movements for chromosomal segregation begins and the transcriptional cascade governing this process is more evolutionary conserved than that of meiosis initiation process in *A. gossypii*, *D. hansenii*, *Y. lipolytica*, *Asp. fumigatus*, *N. crassa*, *K. lactis*, *Sc. pombe*, *Sc. cryophilus*, *Sc. octosporus*, *C. albicans*, *C. tropicalis* and *C. auris*. Literary evidences showed that the core and essential genes are more evolutionary conserved among species [64]. Thus, the presence of core and essential genes in the dataset might show a biased trend of the evolutionary conserved pattern and could be erroneous in demarcating more evolutionary conserved meiotic phase. However, analyzing the genes (123) identified as core and essential genes by Peter *et al*. [65] with our dataset comprising of 582 genes, it showed only 20 genes, that is, only 3.4% are intersecting with their dataset. Thus, it could be conferred that our results are not driven by the presence of evolutionary conserved genes in any of the datasets. Interestingly, we found that there is not any significant difference between the evolutionary rate of transcriptional cascade for meiosis initiation and commitment in any of the *Candida* species considered. This is probably due to the fact that *Candida* sp. undergoes a parasexual cycle instead of meiosis using evolutionary conserved genes like *DMC1* [44]. Similar to the reports, we also found *DLH1* and CTRG_03542 as orthologs in *C. albicans* and *C. tropicalis*, respectively, for *DMC1* in our study. In parasexual cycle, the diploid cells mate to form tetraploid cell without a proper meiotic recombination event and so our results showed no difference in the evolutionary rate of transcriptional cascade for meiosis initiation and commitment for the *Candida* spp. [66]. Further studies are required to elucidate the extent of evolutionary conservation of the meiosis commitment stage in higher eukaryotes.

## Conclusion

The knowledge about the intricate molecular signaling along with its transcriptional regulation which distinguishes meiosis initiation from commitment phase in budding yeast has not been elucidated earlier. So, in this study, we performed *in silico* analyses by reconstructing phase specific PPIN and GRN for meiosis initiation and commitment in budding yeast. We compared the distinct signaling cascade for both the phases and then explored evolutionary gene conservation of the genes for meiosis initiation and commitment of *S. cerevisiae* with 12 species in phylum Ascomycota. Our study identifies potential phase specific molecular players which can be validated experimentally in future. Also, our gene conservation study demarcates meiosis commitment stage to be more evolutionary conserved than the genes of initiation phase in budding yeasts.

## Conflict of interest

The authors declare no conflict of interest.

### Peer review

The peer review history for this article is available at https://www.webofscience.com/api/gateway/wos/peer‐review/10.1002/2211‐5463.13728.

## Author contributions

DD, SP, and HS conceived the idea. SP and DD designed the workflow. DD performed the analysis, drafted the manuscript, and generated the figures. AAC, MAMA, and ASA reviewed and edited the manuscript. HS and SP analyzed the data, prepared the manuscript, and supervised the work.

## Supporting information


**Fig. S1.** Protein–protein Interaction network (PPIN_YPA_) consisting of 1176 nodes and 35 525 edges. The yellow color represents upregulated genes.

**Fig. S2.** Protein–protein Interaction network (PPIN_YPD_) consisting of 1735 nodes and 36 932 edges. The red color represents upregulated genes.

**Fig. S3.** Gene regulatory network (GRN_YPA_) consisting of 865 nodes and 3030 edges.

**Fig. S4.** Gene regulatory network (GRN_YPD_) consisting of 546 nodes and 746 edges.

**Fig. S5.** Protein–protein Interaction network (PPIN_commit_) consisting of 2627 nodes and 39 406 edges. The green color represents upregulated genes.

**Fig. S6.** Gene regulatory network (GRN_commit_) consisting of 1668 nodes and 5111 edges.

**File S1.** Upregulated genes during meiosis initiation in *S. cerevisiae*.

**File S2.** GO Biological Process Enrichment of PPIN_YPA_ and PPIN_YPD_.

**File S3.** Upregulated genes during meiotic commitment in *S. cerevisiae*. The upregulated genes are obtained from comparing transcriptome of non‐committed cells (2,3 and 4 h of growth in SPM) with committed cells (5,6 and 7 h of growth in SPM) of meiosis and then the genes that are downregulated on transferring committed cells to YPD were eliminated from the upregulated set of genes. The remaining genes are the genes for meiosis commitment.

**File S4.** GO Biological Process Enrichment of PPIN_commit_.

**File S5.** Functional enrichment of the distinct transcriptional cascade of meiosis initiation and commitment.

**File S6.** dN/dS values of the genes from distinct transcriptional cascade of meiosis initiation and commitment of *S. cerevisiae* with their orthologs in 12 species.

## Data Availability

The datasets GSE75257, GSE18181, GSE3815 and GSE3816 are publicly available in NCBI Gene Expression Omnibus (GEO) database.
