# Supplementary figures and images for: Insights into the identification and evolutionary conservation of key genes in the transcriptional circuits of meiosis initiation and commitment in budding yeast
Source: FEBS Open Bio. 2023 Nov 14;13(12):2290–305. doi: 10.1002/2211-5463.13728 (PMC10699112; doi:10.1002/2211-5463.13728)

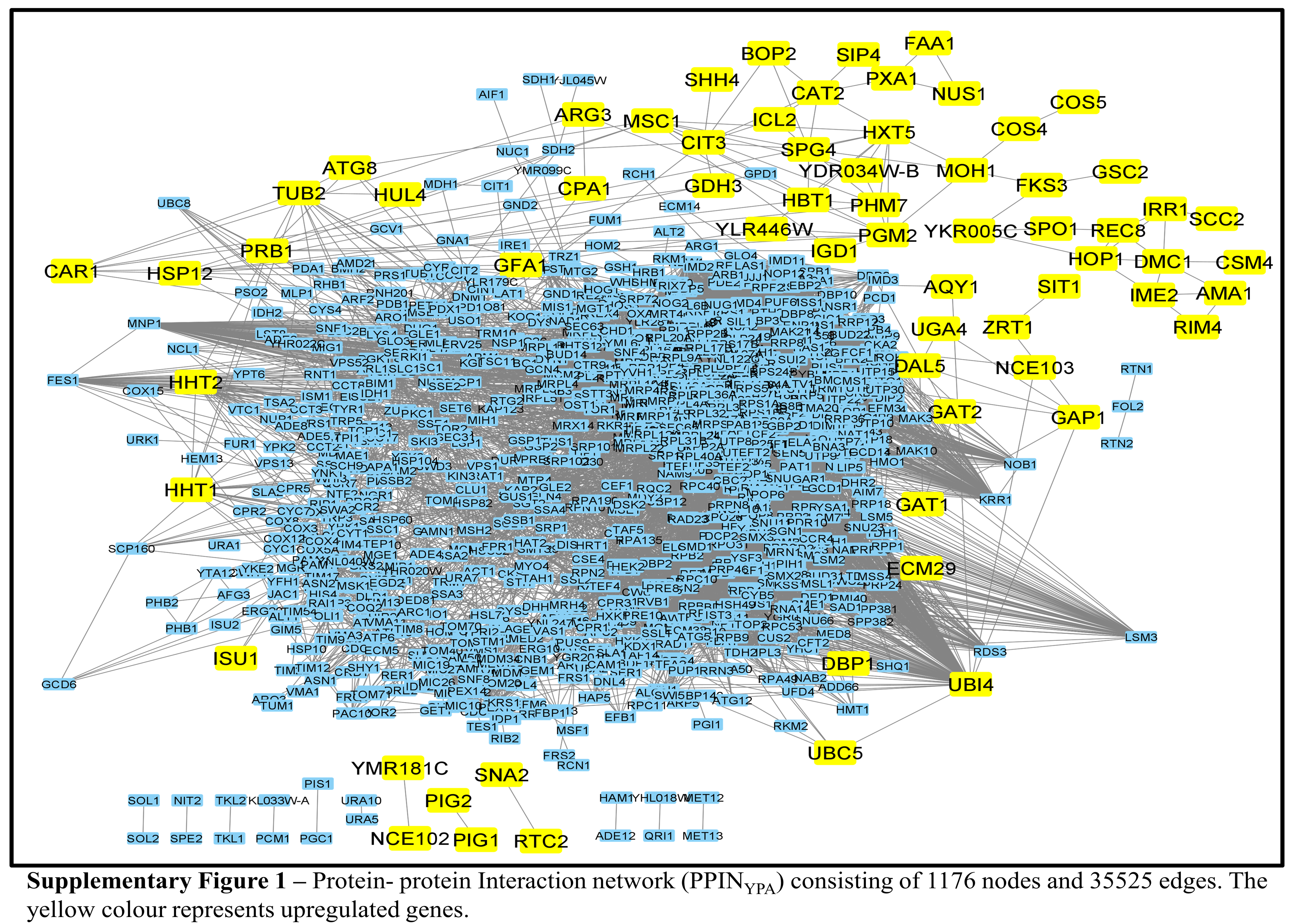

Supplement: Supplementary file 1 — Fig. S1. Protein–protein Interaction network (PPINYPA) consisting of 1176 nodes and 35 525 edges. The yellow color represents upregulated genes. [file FEB4-13-2290-s001.tif]

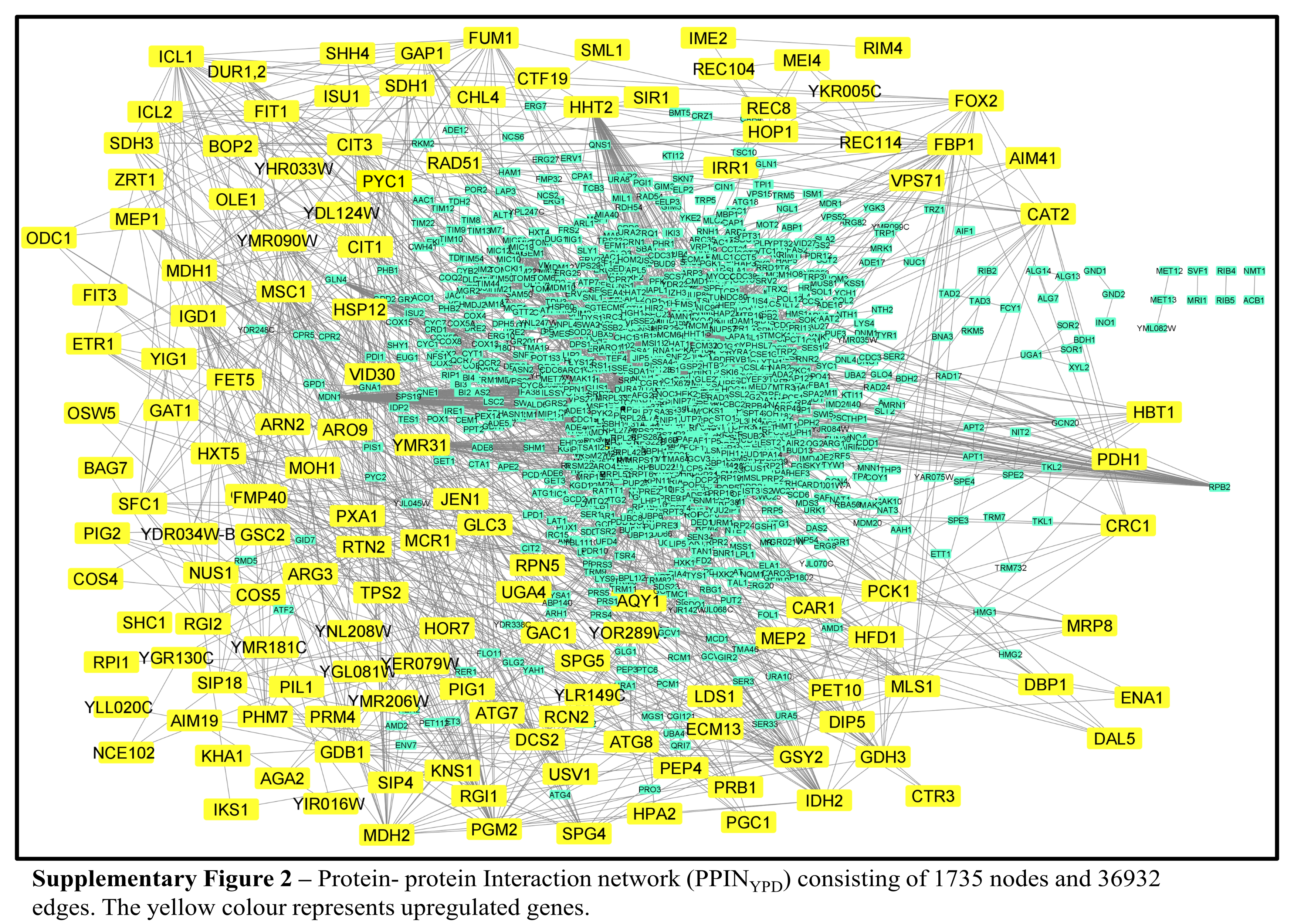

Supplement: Supplementary file 2 — Fig. S2. Protein–protein Interaction network (PPINYPD) consisting of 1735 nodes and 36 932 edges. The red color represents upregulated genes. [file FEB4-13-2290-s003.tif]

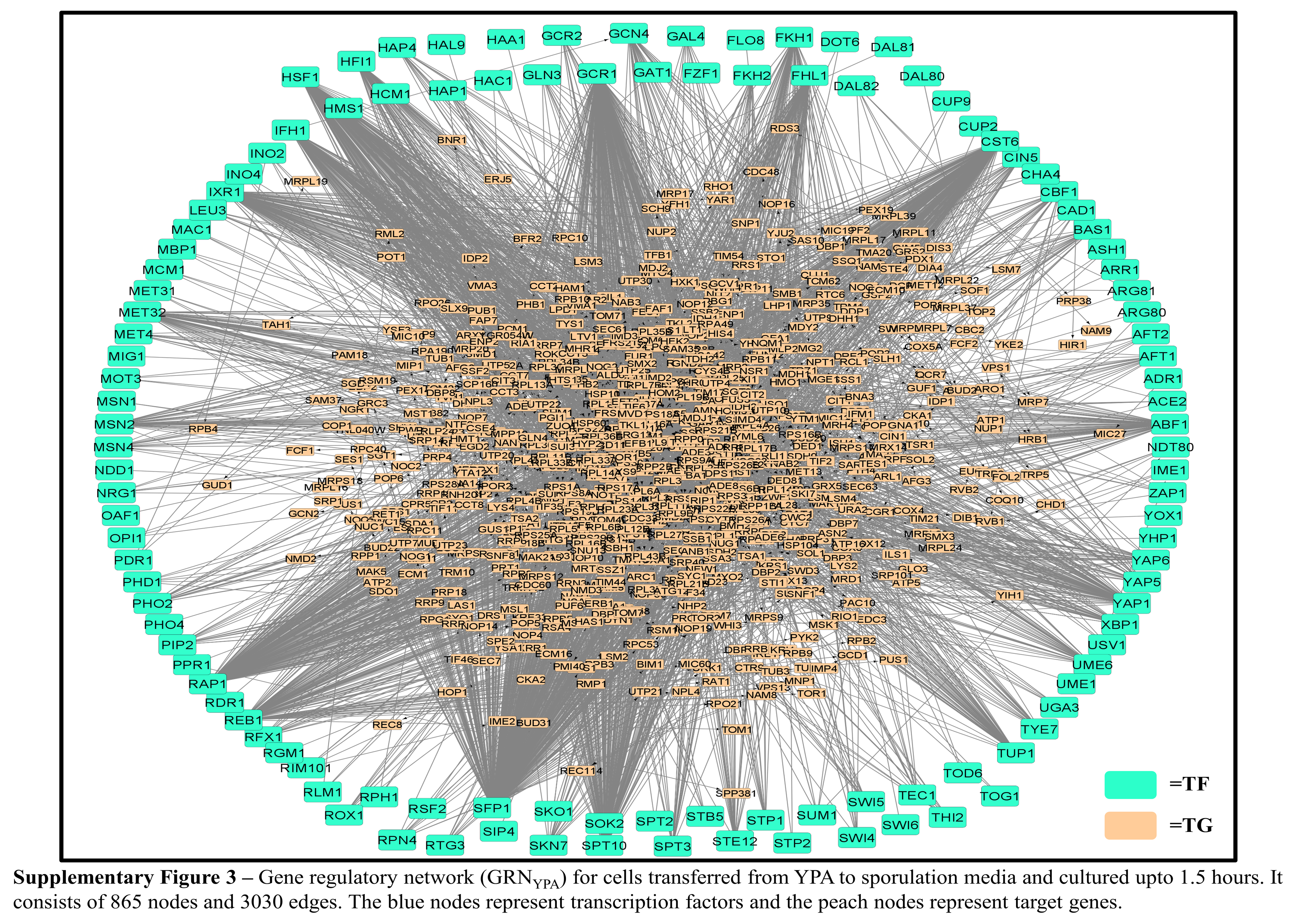

Supplement: Supplementary file 3 — Fig. S3. Gene regulatory network (GRNYPA) consisting of 865 nodes and 3030 edges. [file FEB4-13-2290-s004.tif]

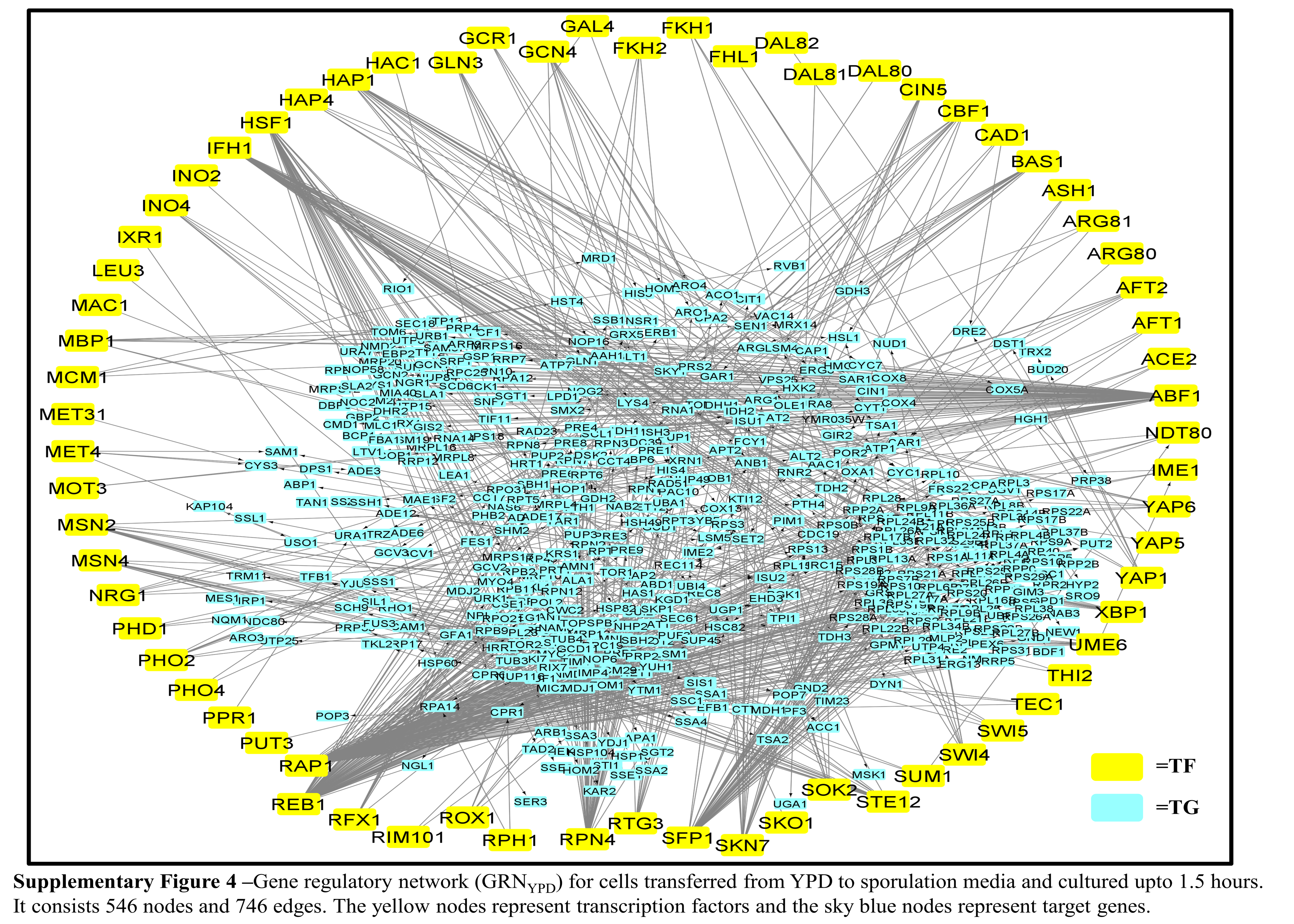

Supplement: Supplementary file 4 — Fig. S4. Gene regulatory network (GRNYPD) consisting of 546 nodes and 746 edges. [file FEB4-13-2290-s010.tif]

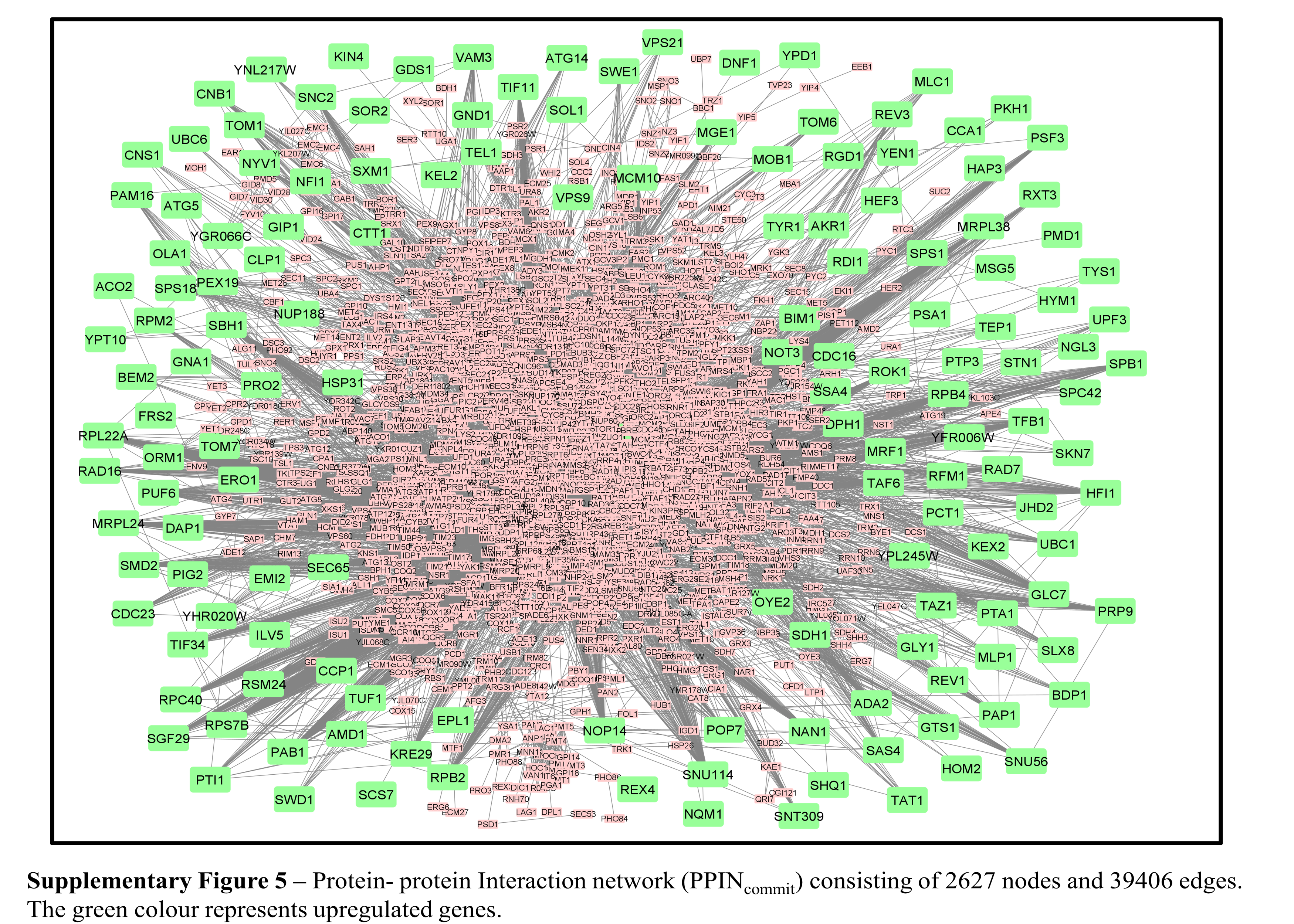

Supplement: Supplementary file 5 — Fig. S5. Protein–protein Interaction network (PPINcommit) consisting of 2627 nodes and 39 406 edges. The green color represents upregulated genes. [file FEB4-13-2290-s007.tif]

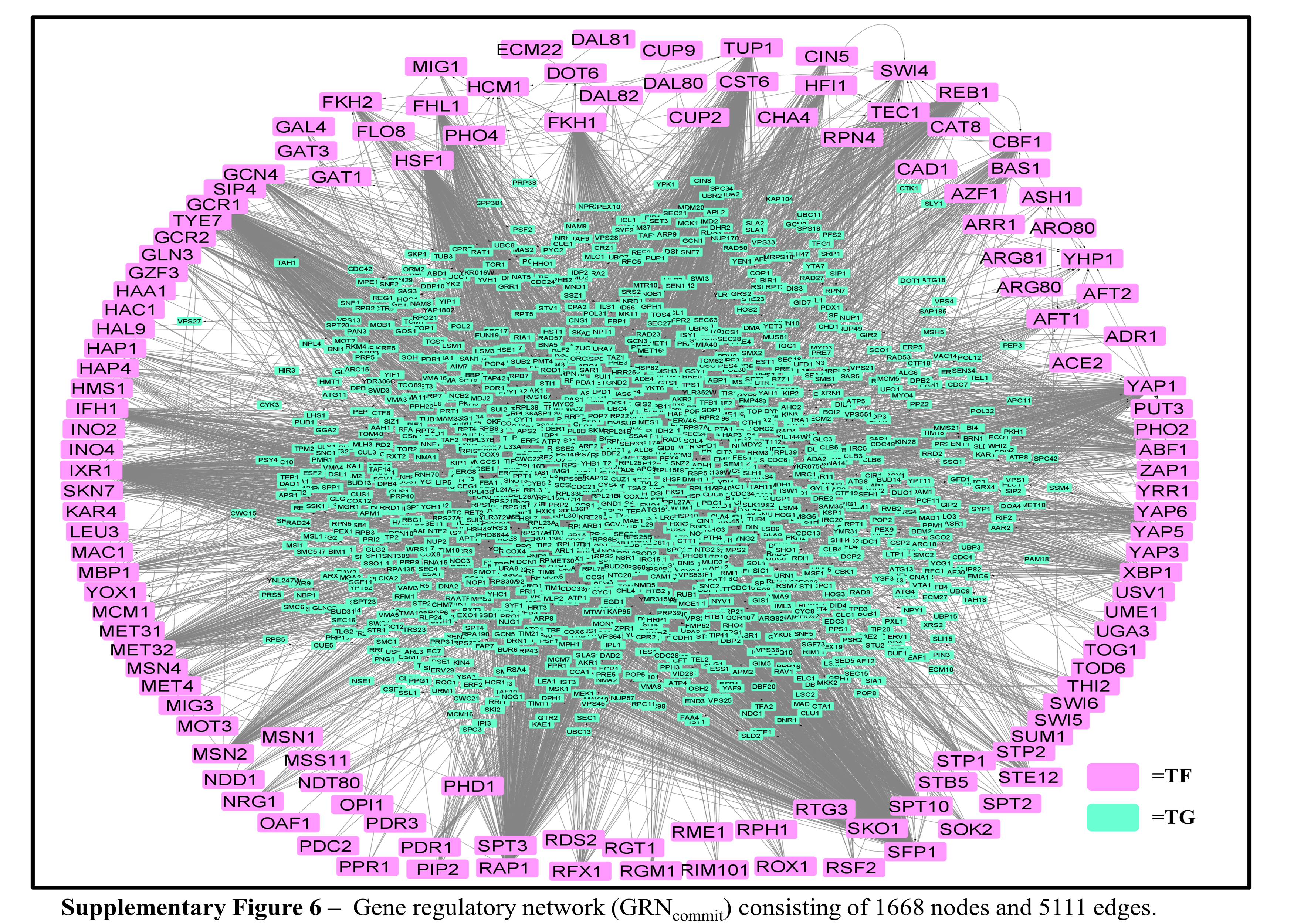

Supplement: Supplementary file 6 — Fig. S6. Gene regulatory network (GRNcommit) consisting of 1668 nodes and 5111 edges. [file FEB4-13-2290-s011.tif]
