# Supplementary material for: Insights into the identification and evolutionary conservation of key genes in the transcriptional circuits of meiosis initiation and commitment in budding yeast
Source: FEBS Open Bio. 2023 Nov 14;13(12):2290–305. doi: 10.1002/2211-5463.13728 (PMC10699112; doi:10.1002/2211-5463.13728)
Supplement: Supplementary file 7 — File S1. Upregulated genes during meiosis initiation in S. cerevisiae. [file FEB4-13-2290-s006.pdf]

Supplementary File 1- Upregulated genes during meiosis initiation in S.cerevisiae						
**YPD vs 1.5 hours in SPM**				**YPA vs 1.5 hours in SPM**		
**Genename/ORFs**	**GSE18181**	**GSE75257**		**Genename/ORFs**	**GSE18181**	**GSE75257**
YOL053C	4.297489	3.9766863		GDH3	1.333136	1.341275
HSP12	4.258812	3.629544		MOH1	2.083454	1.949716
PCK1	4.193705	3.7227573		ATG8	2.523074	2.381187
MLS1	4.134643	3.8691288		HHT1	1.501291	1.205999
SIP4	3.905167	3.9165261		RTC2	1.245766	1.093884
RIM4	3.668647	3.7744996		NUS1	1.679063	1.429397
CAT2	3.552031	3.4726431		UGA4	1.407227	1.303503
ICL1	3.526548	3.0330296		HBT1	1.961689	1.491599
RGI2	3.495895	2.9329348		YDR034W-B	1.762363	1.861271
CIT3	3.49312	3.4669408		ENA2	2.543033	2.509766
FBP1	3.464577	2.5631112		UBC5	1.659075	1.424623
HXT5	3.389918	3.1596904		SCC2	1.226626	1.289654
GAP1	3.357729	4.0759644		SNA2	1.511926	1.276092
YOL053C-A	3.281123	3.0998678		PRB1	2.015789	2.049888
ENA1	3.239656	2.9852143		SIT1	1.124399	1.021735
PDH1	3.126136	2.9824118		DMC1	2.910382	3.006143
CRC1	3.075871	2.9466887		HSP12	2.700725	1.826935
MDH2	3.036477	2.8751645		GAT1	1.958813	1.95728
IME2	2.999724	2.8956649		TUB2	1.340242	1.037595
YAR040C	2.831802	2.7789163		COS4	2.28717	1.725704
YAR037W	2.816078	2.9628267		IGD1	1.209082	1.119465
FIT3	2.80856	2.5476906		AGA2	1.562227	1.319822
MEP2	2.732125	2.8441001		ZRT1	2.058481	1.747199
HOP1	2.714131	2.706591		GSC2	1.836899	1.551095
FOX2	2.66534	2.8653849		ERG25	1.970304	1.657342
ATG8	2.564528	2.5834264		RTS3	1.594401	1.776055
ISU1	2.560132	1.9927319		AMA1	1.974037	1.713133
SPG4	2.526663	2.7650679		RIM4	2.936604	2.730231

SNA2	2.480032	2.0710152
MOH1	2.430899	2.1565441
PRB1	2.411218	2.0979088
ARG3	2.383657	2.2572641
REC8	2.363645	2.34168
JEN1	2.357913	2.4401345
HBT1	2.330726	1.7826431
AQY1	2.308277	2.2106842
PRY1	2.280199	1.9658508
YIR019C	2.245536	2.2071977
DCS2	2.244322	2.2169585
ARO9	2.235503	1.948975
YIL071W	2.185799	1.9132236
YHR033W	2.172214	2.2377549
YMR306C-A	2.168661	1.9640495
PGM2	2.158945	1.791769
PXA1	2.153107	2.0115266
YMR181C	2.12596	1.8856225
PRM4	2.121515	1.7104806
COS4	2.116918	1.6162445
CIT1	2.112919	2.0715907
SIP18	2.107593	1.4356747
SPG5	2.094586	1.7905972
SIR1	2.089476	1.3952479
YIG1	2.043424	2.0932401
GAC1	2.042298	1.8440004
NCE102	2.024339	1.9547486
PEP4	2.022666	1.6726274
ICL2	2.017884	2.0076634
ECM13	2.015802	1.5509073
ZRT1	2.006821	1.4653801
CAR1	1.994588	1.9712034

ECM29	1.530277	1.202405
HXT5	2.997334	2.744538
IRR1	1.477401	1.228878
PIG2	1.01208	1.046536
YIL071W	2.13485	1.661107
HOP1	2.93389	2.900916
YIR043C	1.611089	1.374137
PRY1	1.063323	1.014244
ARG3	1.657301	1.5161
SIP4	2.344054	2.202496
IME2	2.870437	2.795819
HUL4	1.079912	1.051298
DAL5	1.613563	1.902372
COS5	1.491074	1.263851
GFA1	1.263227	1.012051
YKR005C	1.320489	1.576139
GAP1	3.503191	3.354574
SRL3	1.789969	1.411302
UBI4	1.539235	1.481122
SHH4	1.502931	1.223619
BOP2	1.514075	1.631554
PIG1	1.390255	1.301576
YLR446W	2.078203	1.808742
CAT2	1.90053	1.581491
YML089C	1.32247	1.207413
YML090W	1.089097	1.011772
MSC1	1.459671	1.030079
PGM2	1.234406	1.036689
SPG4	2.696156	2.868975
GAT2	1.350698	1.078049
YMR181C	1.778206	1.724205
YMR306C-A	2.248908	2.05465

SHH4	1.956589	1.5126452
SFC1	1.951671	1.6673325
YMR206W	1.901385	1.751786
UBI4	1.896947	1.7479435
HOR7	1.890916	1.894805
ETR1	1.888634	1.8471427
FIT1	1.88497	1.3408502
AGA2	1.879282	1.5833035
IDH2	1.874095	1.6849704
PHM7	1.855423	1.5915904
RAD51	1.85126	1.3781885
ARN2	1.839367	1.1653464
PET10	1.82124	2.0246918
YDR034W-B	1.803621	1.7008675
DAL5	1.776615	1.9990611
GSC2	1.773315	1.6332019
CTR3	1.713862	1.1970809
YMR31	1.668917	1.4970454
BAG7	1.668061	1.0318367
RTN2	1.665944	1.4245102
MRP8	1.665098	1.8060151
YGL081W	1.661617	1.6606522
BOP2	1.660125	1.8033273
YKR005C	1.659395	1.6968514
GSY2	1.654681	1.5884407
FUM1	1.616083	1.4471902
GAT1	1.609288	1.6832368
MSC1	1.603491	1.3294904
YLR149C	1.594658	1.5752555
IGD1	1.588997	1.8004886
DIP5	1.582015	1.7113419
GDB1	1.573312	1.4308405

FKS3	1.581139	1.533525
SPO1	1.210958	1.248272
HHT2	1.508907	1.077681
NCE103	2.813121	1.763772
YOL053C	3.277127	2.98785
PHM7	1.492638	1.19024
CPA1	1.487391	1.282393
FAA1	1.611811	1.267881
MNE1	1.505697	1.391275
CAR1	1.731348	1.636592
DBP1	1.185907	1.400719
ISU1	2.019679	1.488052
PXA1	1.608497	1.400087
YPL199C	1.502058	1.185027
CSM4	1.511416	1.385649
YPL276W	1.17044	1.243668
CIT3	2.444412	2.133473
ICL2	1.20118	1.0686
REC8	2.503348	2.470651
NCE102	1.333873	1.267189
AQY1	1.936067	2.027284

SDH1	1.568794	1.3048343
TPS2	1.564459	1.0244231
YIR043C	1.557258	1.2485663
OLE1	1.541853	1.4502276
PIG2	1.530999	1.5285441
NUS1	1.522194	1.3542413
GLC3	1.500457	1.4836461
YNL208W	1.493459	1.2903925
COX9	1.492003	1.0707784
YLL020C	1.473439	1.3096835
OSW5	1.473277	1.1733384
MNE1	1.464507	1.3337541
PIG1	1.464333	1.496487
MET28	1.463966	1.1488835
USV1	1.452091	1.3222068
KHA1	1.448506	1.3988227
GAD1	1.440991	1.2672112
AIM41	1.438864	1.3983059
RGI1	1.434815	1.2102973
SPO12	1.433521	1.1578246
MEP1	1.419751	1.4088088
KNS1	1.412726	1.2843681
DUR1,2	1.389409	1.681194
RCN2	1.388308	1.4078127
HPA2	1.381302	1.1015899
YOR052C	1.377974	1.4569382
HHT2	1.351546	1.0274489
IRR1	1.349632	1.1661504
YPR098C	1.336485	1.0947785
COS5	1.333635	1.0829966
IKS1	1.320923	1.4100796
RTS3	1.320643	1.5639126

RPI1	1.314462	1.3322751
YKL066W	1.312018	1.2172579
MDH1	1.307049	1.1299307
SHC1	1.306271	1.1412283
REC104	1.30379	1.4014416
YOR289W	1.299282	1.2605989
CTF19	1.295658	1.2081224
YGR130C	1.291862	1.0275917
CPS1	1.27399	1.4996845
YDL124W	1.271998	1.240463
AIM19	1.254926	1.0027098
MCR1	1.252198	1.2645347
GDH3	1.238526	1.262666
YER079W	1.232269	1.0617199
FMP40	1.206185	1.2205866
UGA4	1.201919	1.0806649
ODC1	1.201002	1.307703
RPN5	1.188541	1.0787871
YPL276W	1.179213	1.3158541
SML1	1.161886	1.0504137
LDS1	1.161441	1.2350725
FET5	1.15498	1.0742328
CHL4	1.122509	1.060894
PYC1	1.120746	1.113026
VPS71	1.12013	1.0614827
ATG7	1.113325	1.1489977
VID30	1.111717	1.0264503
SDH3	1.109818	1.0994449
YMR090W	1.106071	1.1657358
MEI4	1.091312	1.1170487
YPR150W	1.088296	1.3162224
PGC1	1.085583	1.0059064

YER010C	1.066167	1.0443289
DBP1	1.062275	1.1997634
REC114	1.059624	1.0880325
YIR016W	1.046273	1.0272947
HFD1	1.033351	1.1317468
PIL1	1.016487	1.0221166
