# Supplementary material for: Insights into the identification and evolutionary conservation of key genes in the transcriptional circuits of meiosis initiation and commitment in budding yeast
Source: FEBS Open Bio. 2023 Nov 14;13(12):2290–305. doi: 10.1002/2211-5463.13728 (PMC10699112; doi:10.1002/2211-5463.13728)
Supplement: Supplementary file 8 — File S2. GO Biological Process Enrichment of PPINYPA and PPINYPD. [file FEB4-13-2290-s008.pdf]

Supplementary File 2 -GO Biological Process Enrichment of PPIN $_{YPA}$ and PPIN $_{YPD}$

Table 2 -GO Biological Process Enrichment of PPIN$_{YPD}$

GO	Description	Count Metascape	FDR Metascape	Count Cluster profiler	p.adjust Cluster Profiler
GO:0042254	ribosome biogenesis	345	2.15733E-97	324	1.28E-95
GO:0034470	ncRNA processing	308	1.70119E-66	286	1.53E-62
GO:0016072	rRNA metabolic process	266	1.21245E-69	240	4.93E-60
GO:0071826	ribonucleoprotein complex subunit organization	156	6.07052E-60	153	6.11E-59
GO:0022618	ribonucleoprotein complex assembly	151	4.66752E-58	148	7.01E-57
GO:0006364	rRNA processing	251	6.06558E-65	228	1.89E-56
GO:0042274	ribosomal small subunit biogenesis	135	1.46559E-62	123	5.24E-50
GO:0042273	ribosomal large subunit biogenesis	116	2.227E-50	108	1.08E-45
GO:0016071	mRNA metabolic process	198	8.75978E-32	199	4.18E-39
GO:0006403	RNA localization	108	1.50258E-25	132	3.49E-38
GO:0002181	cytoplasmic translation	180	6.76964E-74	139	4.50E-38
GO:0030490	maturation of SSU-rRNA	113	1.65018E-52	98	9.90E-38
GO:0050657	nucleic acid transport	94	1.02235E-22	118	8.62E-35
GO:0050658	RNA transport	92	6.84426E-22	116	6.87E-34
GO:0051236	establishment of RNA localization	92	6.84426E-22	116	6.87E-34
GO:0000460	maturation of 5.8S rRNA	82	4.00389E-33	80	8.28E-34
GO:0000466	maturation of 5.8S rRNA from tricistronic rRNA transcript (SSU-rRNA 5.8S rRNA LSU-rRNA)	82	4.00389E-33	80	8.28E-34
GO:0006913	nucleocytoplasmic transport	133	5.00647E-35	123	8.28E-34
GO:0051169	nuclear transport	133	5.00647E-35	123	8.28E-34
GO:0000462	maturation of SSU-rRNA from tricistronic rRNA transcript (SSU-rRNA 5.8S rRNA LSU-rRNA)	102	1.65634E-48	88	9.64E-34
GO:0042255	ribosome assembly	70	4.28624E-30	69	7.96E-30

GO:0071426	ribonucleoprotein complex export from nucleus	102	1.80277E-33	92	1.28E-29
GO:0071166	ribonucleoprotein complex localization	102	1.99062E-32	92	9.31E-29
GO:0051168	nuclear export	104	2.39517E-31	95	1.61E-28
GO:0006405	RNA export from nucleus	67	9.31202E-17	92	2.35E-28
GO:0000469	cleavage involved in rRNA processing	72	1.38101E-26	70	8.70E-28
GO:0006397	mRNA processing	129	2.24524E-21	130	7.01E-27
GO:0000398	mRNA splicing via spliceosome	86	2.94747E-23	86	2.43E-26
GO:0000377	RNA splicing via transesterification reactions with bulged adenosine as nucleophile	86	7.60457E-23	86	6.24E-26
GO:0000375	RNA splicing via transesterification reactions	89	9.20302E-22	89	5.79E-25
GO:0000478	endonucleolytic cleavage involved in rRNA processing	56	2.17074E-25	54	1.49E-24
GO:0000479	endonucleolytic cleavage of tricistronic rRNA transcript (SSU-rRNA 5.8S rRNA LSU-rRNA)	56	2.17074E-25	54	1.49E-24
GO:0008380	RNA splicing	97	6.89103E-20	97	2.29E-23
GO:0031503	protein-containing complex localization	110	2.73333E-24	100	4.17E-23
GO:0000470	maturation of LSU-rRNA	59	3.69404E-25	53	1.57E-22
GO:0015931	nucleobase-containing compound transport	101	4.13553E-14	124	2.27E-22
GO:0071428	rRNA-containing ribonucleoprotein complex export from nucleus	63	2.98495E-27	56	3.23E-22
GO:0000463	maturation of LSU-rRNA from tricistronic rRNA transcript (SSU-rRNA 5.8S rRNA LSU-rRNA)	48	2.7864E-20	46	7.46E-21
GO:0006399	tRNA metabolic process	112	1.83285E-18	101	6.28E-20

GO:0000447	endonucleolytic cleavage in ITS1 to separate SSU-rRNA from 5.8S rRNA and LSU-rRNA from tricistronic rRNA transcript (SSU-rRNA 5.8S rRNA LSU-rRNA)	44	4.97477E-21	42	2.15E-19
GO:0000956	nuclear-transcribed mRNA catabolic process	78	4.54344E-15	79	1.50E-18
GO:0000054	ribosomal subunit export from nucleus	48	1.80181E-19	43	1.65E-16
GO:0090501	RNA phosphodiester bond hydrolysis	105	2.52056E-13	105	2.13E-16
GO:0006402	mRNA catabolic process	81	5.17882E-13	82	2.15E-16
GO:0000967	rRNA 5'-end processing	35	2.54276E-14	35	3.00E-16
GO:0033750	ribosome localization	48	1.0317E-18	43	5.69E-16
GO:0006626	protein targeting to mitochondrion	48	3.61873E-13	48	5.80E-16
GO:0006401	RNA catabolic process	92	8.87274E-13	91	9.90E-16
GO:0017038	protein import	77	7.03839E-13	76	1.17E-15
GO:0070925	organelle assembly	106	4.57602E-13	105	1.17E-15
GO:0000480	endonucleolytic cleavage in 5'-ETS of tricistronic rRNA transcript (SSU-rRNA 5.8S rRNA LSU-rRNA)	32	1.3061E-13	32	2.00E-15
GO:0019693	ribose phosphate metabolic process	74	2.22503E-11	76	2.07E-15
GO:0000472	endonucleolytic cleavage to generate mature 5'-end of SSU-rRNA from (SSU-rRNA 5.8S rRNA LSU-rRNA)	33	4.04889E-14	33	3.57E-15
GO:0034655	nucleobase-containing compound catabolic process	100	1.12178E-11	98	1.11E-14
GO:0070585	protein localization to mitochondrion	49	6.27972E-11	49	1.45E-13
GO:0072655	establishment of protein localization to mitochondrion	49	6.27972E-11	49	1.45E-13
GO:0000027	ribosomal large subunit assembly	35	2.54276E-14	35	1.45E-13
GO:0044265	cellular macromolecule catabolic process	185	1.12493E-09	181	2.15E-13
GO:0009259	ribonucleotide metabolic process	68	1.10054E-09	70	2.19E-13

GO:0090502	RNA phosphodiester bond hydrolysis endonucleolytic	79	3.22567E-11	78	2.52E-13
GO:0034471	ncRNA 5'-end processing	40	2.03553E-12	37	5.47E-13
GO:0000028	ribosomal small subunit assembly	26	1.3061E-13	26	6.44E-13
GO:0051028	mRNA transport	59	5.77017E-10	59	9.02E-13
GO:0140053	mitochondrial gene expression	97	2.78639E-13	99	1.07E-12
GO:0008033	tRNA processing	73	2.18418E-09	73	1.17E-12
GO:0000966	RNA 5'-end processing	40	6.45475E-12	37	1.59E-12
GO:0090305	nucleic acid phosphodiester bond hydrolysis	129	7.68896E-10	127	1.89E-12
GO:0032543	mitochondrial translation	86	3.44926E-14	88	2.21E-12
GO:0006163	purine nucleotide metabolic process	64	4.35269E-09	66	3.42E-12
GO:0019439	aromatic compound catabolic process	103	2.8133E-09	101	3.42E-12
GO:0006415	translational termination	25	7.6102E-11	24	8.22E-12
GO:0016973	poly(A)+ mRNA export from nucleus	30	2.18418E-09	30	1.28E-11
GO:0046700	heterocycle catabolic process	102	9.30372E-09	100	1.29E-11
GO:0044270	cellular nitrogen compound catabolic process	103	1.85354E-08	101	2.66E-11
GO:1901361	organic cyclic compound catabolic process	104	2.68106E-08	102	3.79E-11
GO:0043144	sno(s)RNA processing	34	2.65263E-09	34	4.04E-11
GO:0097064	ncRNA export from nucleus	36	8.05667E-15	29	4.06E-11
GO:0006099	tricarboxylic acid cycle	12	7.64519E-10	12	6.85E-11
GO:0006413	translational initiation	48	3.26228E-10	46	9.86E-11
GO:0051170	import into nucleus	46	1.3002E-08	45	1.27E-10
GO:0071806	protein transmembrane transport	49	7.19844E-08	49	1.57E-10
GO:0016074	sno(s)RNA metabolic process	36	1.37557E-08	36	2.01E-10
GO:0009150	purine ribonucleotide metabolic process	57	2.178E-07	59	2.20E-10
GO:0072521	purine-containing compound metabolic process	67	2.37189E-07	69	2.44E-10
GO:0042026	protein refolding	24	7.39041E-09	24	2.52E-10
GO:0002183	cytoplasmic translational initiation	27	4.85872E-11	25	3.21E-10

GO:0006406	mRNA export from nucleus	41	5.96855E-08	41	3.24E-10
GO:0071427	mRNA-containing ribonucleoprotein complex export from nucleus	41	5.96855E-08	41	3.24E-10
GO:0009117	nucleotide metabolic process	83	1.05441E-06	85	3.40E-10
GO:0046390	ribose phosphate biosynthetic process	47	3.06032E-07	49	4.90E-10
GO:0016051	carbohydrate biosynthetic process	17	8.74983E-09	17	8.29E-10
GO:0006457	protein folding	62	7.52428E-07	62	1.40E-09
GO:0006753	nucleoside phosphate metabolic process	84	4.45069E-06	86	1.40E-09
GO:0006360	transcription by RNA polymerase I	43	2.47921E-07	42	1.93E-09
GO:0006520	cellular amino acid metabolic process	106	1.19988E-06	105	3.26E-09
GO:0009156	ribonucleoside monophosphate biosynthetic process	28	1.27082E-07	28	3.59E-09
GO:0006613	cotranslational protein targeting to membrane	19	8.4659E-08	19	4.74E-09
GO:0006614	SRP-dependent cotranslational protein targeting to membrane	19	8.4659E-08	19	4.74E-09
GO:0065002	intracellular protein transmembrane transport	43	1.18441E-06	43	4.94E-09
GO:0043624	cellular protein complex disassembly	36	2.63286E-07	35	5.63E-09
GO:0006606	protein import into nucleus	41	2.08163E-07	40	6.11E-09
GO:0055086	nucleobase-containing small molecule metabolic process	93	1.83702E-05	95	6.53E-09
GO:0043038	amino acid activation	29	2.90533E-07	29	7.87E-09
GO:0043039	tRNA aminoacylation	29	2.90533E-07	29	7.87E-09
GO:0009304	tRNA transcription	17	8.01542E-07	17	8.37E-09
GO:0042797	tRNA transcription by RNA polymerase III	17	8.01542E-07	17	8.37E-09
GO:0006418	tRNA aminoacylation for protein translation	27	3.2048E-07	27	9.94E-09
GO:0031123	RNA 3'-end processing	45	4.64237E-07	44	1.10E-08
GO:0000055	ribosomal large subunit export from nucleus	29	2.67242E-11	25	1.14E-08

GO:0006400	tRNA modification	45	1.98454E-06	45	1.39E-08
GO:0009260	ribonucleotide biosynthetic process	42	5.86965E-06	44	1.88E-08
GO:0051656	establishment of organelle localization	68	8.4659E-08	63	2.51E-08
GO:0009060	aerobic respiration	16	1.66659E-07	15	2.69E-08
GO:0071025	RNA surveillance	26	8.10035E-07	26	2.87E-08
GO:0006839	mitochondrial transport	52	5.75442E-05	54	3.83E-08
GO:0072522	purine-containing compound biosynthetic process	41	1.15139E-05	43	4.31E-08
GO:0009124	nucleoside monophosphate biosynthetic process	28	1.53001E-06	28	5.02E-08
GO:0009161	ribonucleoside monophosphate metabolic process	28	1.53001E-06	28	5.02E-08
GO:0030150	protein import into mitochondrial matrix	19	1.53144E-06	19	9.83E-08
GO:0051031	tRNA transport	24	1.57448E-09	19	9.83E-08
GO:0006164	purine nucleotide biosynthetic process	38	2.17249E-05	40	1.05E-07
GO:0006616	SRP-dependent cotranslational protein targeting to membrane translocation	13	1.4817E-06	13	1.54E-07
GO:0043628	ncRNA 3'-end processing	29	5.34447E-06	29	1.92E-07
GO:0010608	posttranscriptional regulation of gene expression	111	7.97633E-10	88	1.93E-07
GO:0016073	snRNA metabolic process	24	4.83315E-06	24	2.33E-07
GO:0071027	nuclear RNA surveillance	24	4.83315E-06	24	2.33E-07
GO:0034637	cellular carbohydrate biosynthetic process	12	9.97668E-07	12	2.66E-07
GO:0006112	energy reserve metabolic process	10	9.97668E-07	10	2.77E-07
GO:0032984	protein-containing complex disassembly	47	1.04321E-05	46	2.99E-07
GO:0051640	organelle localization	91	6.93805E-06	87	3.31E-07
GO:1901293	nucleoside phosphate biosynthetic process	56	0.000109225	58	3.50E-07
GO:0034661	ncRNA catabolic process	29	2.90533E-07	25	4.53E-07

GO:0009165	nucleotide biosynthetic process	55	0.000135245	57	4.76E-07
GO:0045333	cellular respiration	16	1.03216E-06	15	5.24E-07
GO:0001172	transcription RNA-templated	12	4.83315E-06	12	5.89E-07
GO:0010499	proteasomal ubiquitin-independent protein catabolic process	15	6.92109E-06	15	6.66E-07
GO:0000245	spliceosomal complex assembly	22	2.10906E-06	22	7.48E-07
GO:0090150	establishment of protein localization to membrane	53	8.71647E-05	53	7.86E-07
GO:0046112	nucleobase biosynthetic process	17	1.17285E-05	17	9.97E-07
GO:0051204	protein insertion into mitochondrial membrane	18	1.30198E-05	18	1.04E-06
GO:0007006	mitochondrial membrane organization	31	2.1753E-05	32	1.06E-06
GO:0005975	carbohydrate metabolic process	22	8.06901E-06	22	1.32E-06
GO:0005978	glycogen biosynthetic process	8	4.6324E-06	8	1.32E-06
GO:0016075	rRNA catabolic process	27	7.79771E-07	23	1.46E-06
GO:0098781	ncRNA transcription	36	1.32359E-05	34	1.75E-06
GO:0071028	nuclear mRNA surveillance	21	3.05129E-05	21	2.09E-06
GO:0000184	nuclear-transcribed mRNA catabolic process nonsense-mediated decay	24	2.16275E-05	24	2.51E-06
GO:0006458	'de novo' protein folding	19	3.47319E-05	19	2.76E-06
GO:0006407	rRNA export from nucleus	17	1.29582E-07	15	2.82E-06
GO:0051029	rRNA transport	17	1.29582E-07	15	2.82E-06
GO:0006417	regulation of translation	106	2.65263E-09	80	2.93E-06
GO:0000393	spliceosomal conformational changes to generate catalytic conformation	18	4.44933E-06	18	2.95E-06
GO:0006383	transcription by RNA polymerase III	28	2.17249E-05	26	2.95E-06
GO:0000387	spliceosomal snRNP assembly	16	2.3473E-06	16	3.05E-06
GO:0001731	formation of translation preinitiation complex	19	4.06918E-07	17	3.06E-06
GO:0006450	regulation of translational fidelity	24	2.08433E-06	22	3.77E-06
GO:0022411	cellular component disassembly	70	0.000301663	69	3.97E-06
GO:0061077	chaperone-mediated protein folding	19	0.000178477	19	6.69E-06

GO:0034248	regulation of cellular amide metabolic process	106	9.14904E-09	80	7.41E-06
GO:0009123	nucleoside monophosphate metabolic process	29	0.00016288	29	7.44E-06
GO:0006006	glucose metabolic process	11	3.77622E-05	11	7.48E-06
GO:0000294	nuclear-transcribed mRNA catabolic process endonucleolytic cleavage-dependent decay	10	5.23497E-05	10	8.19E-06
GO:0001732	formation of cytoplasmic translation initiation complex	10	5.23497E-05	10	8.19E-06
GO:1902626	assembly of large subunit precursor of preribosome	8	0.000589595	10	8.19E-06
GO:0034504	protein localization to nucleus	45	0.000205235	44	8.33E-06
GO:0006607	NLS-bearing protein import into nucleus	14	7.93085E-05	14	8.76E-06
GO:0070478	nuclear-transcribed mRNA catabolic process 3'-5' exonucleolytic nonsense-mediated decay	14	7.93085E-05	14	8.76E-06
GO:0000291	nuclear-transcribed mRNA catabolic process exonucleolytic	16	9.44955E-05	16	9.07E-06
GO:0008298	intracellular mRNA localization	21	0.000137328	21	9.62E-06
GO:0072657	protein localization to membrane	66	0.000171671	60	1.01E-05
GO:0051205	protein insertion into membrane	23	0.000184873	23	1.18E-05
GO:0031126	sno(s)RNA 3'-end processing	20	0.000158686	20	1.19E-05
GO:0009152	purine ribonucleotide biosynthetic process	31	0.001320009	33	1.50E-05
GO:0044262	cellular carbohydrate metabolic process	16	0.000120751	16	1.96E-05
GO:0044743	protein transmembrane import into intracellular organelle	29	0.000417047	29	2.02E-05
GO:0009250	glucan biosynthetic process	9	9.08607E-05	9	2.05E-05
GO:0045047	protein targeting to ER	25	0.000623987	25	2.32E-05
GO:0033967	box C/D RNA metabolic process	12	0.000178477	12	2.43E-05
GO:0034963	box C/D RNA processing	12	0.000178477	12	2.43E-05

GO:0005977	glycogen metabolic process	8	0.000106576	8	2.50E-05
GO:0033692	cellular polysaccharide biosynthetic process	9	0.000120751	9	2.61E-05
GO:0071038	nuclear polyadenylation-dependent tRNA catabolic process	13	0.000215165	13	2.78E-05
GO:0106354	tRNA surveillance	13	0.000215165	13	2.78E-05
GO:0006409	tRNA export from nucleus	19	4.06918E-07	14	2.79E-05
GO:0034427	nuclear-transcribed mRNA catabolic process exonucleolytic 3'-5'	14	0.000235762	14	2.79E-05
GO:0071431	tRNA-containing ribonucleoprotein complex export from nucleus	19	4.06918E-07	14	2.79E-05
GO:0000271	polysaccharide biosynthetic process	9	0.00013029	9	2.95E-05
GO:0019752	carboxylic acid metabolic process	25	0.000207861	25	3.50E-05
GO:0034243	regulation of transcription elongation from RNA polymerase II promoter	26	0.001278995	27	3.63E-05
GO:0072599	establishment of protein localization to endoplasmic reticulum	25	0.001025418	25	3.91E-05
GO:0009201	ribonucleoside triphosphate biosynthetic process	18	0.002745737	20	4.93E-05
GO:0019318	hexose metabolic process	11	0.000280893	11	5.53E-05
GO:0040031	snRNA modification	10	0.000371602	10	6.13E-05
GO:0006446	regulation of translational initiation	21	1.32064E-05	19	6.38E-05
GO:0043436	oxoacid metabolic process	25	0.00045697	25	7.30E-05
GO:0051084	'de novo' posttranslational protein folding	14	0.000623987	14	7.87E-05
GO:0051085	chaperone cofactor-dependent protein refolding	14	0.000623987	14	7.87E-05
GO:0009607	response to biotic stimulus	11	0.000528099	11	8.01E-05
GO:0006082	organic acid metabolic process	25	0.000574256	25	8.23E-05
GO:0009218	pyrimidine ribonucleotide metabolic process	12	0.001640034	12	8.59E-05
GO:0009220	pyrimidine ribonucleotide biosynthetic process	12	0.000604167	12	8.59E-05

GO:0071042	nuclear polyadenylation-dependent mRNA catabolic process	12	0.000604167	12	8.59E-05
GO:0071047	polyadenylation-dependent mRNA catabolic process	12	0.000604167	12	8.59E-05
GO:0002097	tRNA wobble base modification	18	0.007549267	20	9.07E-05
GO:0009199	ribonucleoside triphosphate metabolic process	18	0.00458139	20	9.07E-05
GO:0032968	positive regulation of transcription elongation from RNA polymerase II promoter	24	0.003022243	25	0.000106421
GO:0006090	pyruvate metabolic process	23	0.001566264	23	0.00011815
GO:0090151	establishment of protein localization to mitochondrial membrane	19	0.001301653	19	0.000119031
GO:0030488	tRNA methylation	16	0.001148557	16	0.000124976
GO:0070651	nonfunctional rRNA decay	18	0.000199283	16	0.000124976
GO:0006073	cellular glucan metabolic process	9	0.000676209	9	0.000140443
GO:0044042	glucan metabolic process	9	0.000676209	9	0.000140443
GO:0005996	monosaccharide metabolic process	11	0.000755006	11	0.000140443
GO:0009127	purine nucleoside monophosphate biosynthetic process	15	0.001306165	15	0.000151758
GO:0009168	purine ribonucleoside monophosphate biosynthetic process	15	0.001306165	15	0.000151758
GO:0043244	regulation of protein-containing complex disassembly	18	0.001551582	18	0.000151758
GO:0043633	polyadenylation-dependent RNA catabolic process	15	0.001306165	15	0.000151758
GO:0043634	polyadenylation-dependent ncRNA catabolic process	14	0.001453627	14	0.000182429
GO:0071029	nuclear ncRNA surveillance	14	0.001453627	14	0.000182429
GO:0071046	nuclear polyadenylation-dependent ncRNA catabolic process	14	0.001453627	14	0.000182429
GO:0006353	DNA-templated transcription termination	23	0.002469238	23	0.00018915

GO:0009113	purine nucleobase biosynthetic process	9	0.001144577	9	0.000198442
GO:0031070	intronic snoRNA processing	9	0.001144577	9	0.000198442
GO:0031120	snRNA pseudouridine synthesis	9	0.001144577	9	0.000198442
GO:0034965	intronic box C/D RNA processing	9	0.001144577	9	0.000198442
GO:0044264	cellular polysaccharide metabolic process	9	0.000977696	9	0.000202821
GO:0018208	peptidyl-proline modification	13	0.001579091	13	0.000210348
GO:0000288	nuclear-transcribed mRNA catabolic process deadenylation-dependent decay	24	0.003022243	24	0.000220401
GO:0006631	fatty acid metabolic process	9	0.00132498	9	0.000222084
GO:0000413	protein peptidyl-prolyl isomerization	12	0.001640034	12	0.000236929
GO:0045039	protein insertion into mitochondrial inner membrane	10	0.001478264	10	0.000242789
GO:0000056	ribosomal small subunit export from nucleus	12	0.000604167	11	0.000246195
GO:0007008	outer mitochondrial membrane organization	10	0.004324153	11	0.000246195
GO:0070972	protein localization to endoplasmic reticulum	29	0.005887348	29	0.000246195
GO:0071034	CUT catabolic process	11	0.001633786	11	0.000246195
GO:0071043	CUT metabolic process	11	0.001633786	11	0.000246195
GO:1990542	mitochondrial transmembrane transport	28	0.027834454	30	0.000260294
GO:0006356	regulation of transcription by RNA polymerase I	21	0.002436445	20	0.000263937
GO:1901607	alpha-amino acid biosynthetic process	47	0.00379688	47	0.000283175
GO:0031125	rRNA 3'-end processing	15	0.002620443	15	0.000301659
GO:0046034	ATP metabolic process	45	0.001284582	41	0.000315872
GO:0043248	proteasome assembly	19	0.003749356	19	0.000348486
GO:0008652	cellular amino acid biosynthetic process	53	0.006367595	52	0.000384576

GO:0009142	nucleoside triphosphate biosynthetic process	18	0.018155853	20	0.000432501
GO:1903311	regulation of mRNA metabolic process	48	4.06918E-07	23	0.000440967
GO:0033865	nucleoside bisphosphate metabolic process	18	0.00458139	18	0.000454373
GO:0033875	ribonucleoside bisphosphate metabolic process	18	0.00458139	18	0.000454373
GO:0034032	purine nucleoside bisphosphate metabolic process	18	0.00458139	18	0.000454373
GO:0071035	nuclear polyadenylation-dependent rRNA catabolic process	13	0.003478552	13	0.000454373
GO:0043648	dicarboxylic acid metabolic process	25	0.007549267	25	0.000542456
GO:0032784	regulation of DNA-templated transcription elongation	26	0.019954552	27	0.000630509
GO:0005976	polysaccharide metabolic process	9	0.002713482	9	0.000630856
GO:0071051	polyadenylation-dependent snoRNA 3'-end processing	11	0.004194019	11	0.000642787
GO:0006449	regulation of translational termination	9	0.004104758	9	0.000729821
GO:0006637	acyl-CoA metabolic process	14	0.005887348	14	0.000751007
GO:0035383	thioester metabolic process	14	0.005887348	14	0.000751007
GO:1901657	glycosyl compound metabolic process	18	0.024392783	22	0.000919196
GO:0006612	protein targeting to membrane	29	0.018922855	29	0.000967119
GO:0006094	gluconeogenesis	6	0.004686433	6	0.00101668
GO:0019319	hexose biosynthetic process	6	0.004686433	6	0.00101668
GO:0051321	meiotic cell cycle	19	0.00610779	19	0.00101668
GO:0007007	inner mitochondrial membrane organization	17	0.003372834	17	0.001058456
GO:0009126	purine nucleoside monophosphate metabolic process	15	0.008713684	15	0.001080333
GO:0009167	purine ribonucleoside monophosphate metabolic process	15	0.008713684	15	0.001080333
GO:0009116	nucleoside metabolic process	16	0.025870359	20	0.001101194

GO:0006730	one-carbon metabolic process	12	0.008223417	12	0.001219935
GO:0009141	nucleoside triphosphate metabolic process	19	0.037776508	21	0.001240744
GO:0046364	monosaccharide biosynthetic process	18	0.011754077	18	0.001275858
GO:1903046	meiotic cell cycle process	17	0.008317444	17	0.001339689
GO:0010906	regulation of glucose metabolic process	6	0.008852837	6	0.001339689
GO:0002098	tRNA wobble uridine modification	16	0.018404204	16	0.001411233
GO:0045943	positive regulation of transcription by RNA polymerase I	14	0.018188109	14	0.001413179
GO:0046822	regulation of nucleocytoplasmic transport	12	0.040757751	14	0.001413179
GO:2000144	positive regulation of DNA-templated transcription initiation	11	0.018404204	11	0.001491804
GO:0032786	positive regulation of DNA-templated transcription elongation	24	0.03984682	25	0.001636229
GO:0006165	nucleoside diphosphate phosphorylation	17	0.014780677	17	0.001698128
GO:0006084	acetyl-CoA metabolic process	10	0.010647576	10	0.001779678
GO:0006222	UMP biosynthetic process	8	0.011267125	8	0.002162343
GO:0009173	pyrimidine ribonucleoside monophosphate metabolic process	8	0.011267125	8	0.002162343
GO:0009174	pyrimidine ribonucleoside monophosphate biosynthetic process	8	0.011267125	8	0.002162343
GO:0009309	amine biosynthetic process	8	0.011267125	8	0.002162343
GO:0042401	cellular biogenic amine biosynthetic process	8	0.011267125	8	0.002162343
GO:0046049	UMP metabolic process	8	0.011267125	8	0.002162343
GO:0051095	regulation of helicase activity	8	0.011267125	8	0.002162343
GO:0070096	mitochondrial outer membrane translocase complex assembly	7	0.026904018	8	0.002162343
GO:0071039	nuclear polyadenylation-dependent CUT catabolic process	8	0.011267125	8	0.002162343

GO:0006096	glycolytic process	16	0.018404204	16	0.002225597
GO:0006757	ATP generation from ADP	16	0.018404204	16	0.002225597
GO:0062012	regulation of small molecule metabolic process	8	0.015385817	8	0.002245151
GO:0071496	cellular response to external stimulus	11	0.014002057	11	0.00225084
GO:0031668	cellular response to extracellular stimulus	11	0.014002057	11	0.00225084
GO:0010675	regulation of cellular carbohydrate metabolic process	6	0.014904008	6	0.002272362
GO:0046939	nucleotide phosphorylation	17	0.020848584	17	0.002645584
GO:0032787	monocarboxylic acid metabolic process	12	0.016831887	12	0.002920249
GO:0006448	regulation of translational elongation	13	0.000631156	11	0.003000151
GO:0045292	mRNA cis splicing via spliceosome	12	0.003898634	11	0.003000151
GO:0090503	RNA phosphodiester bond hydrolysis exonucleolytic	17	0.020848584	18	0.003000151
GO:0009267	cellular response to starvation	8	0.022425879	8	0.003127321
GO:0006812	cation transport	15	0.023030439	15	0.003134726
GO:0006109	regulation of carbohydrate metabolic process	6	0.020915163	6	0.00314124
GO:0009991	response to extracellular stimulus	11	0.022535211	11	0.003736136
GO:0034475	U4 snRNA 3'-end processing	10	0.020340355	10	0.003856085
GO:0060261	positive regulation of transcription initiation from RNA polymerase II promoter	10	0.037380901	10	0.003856085
GO:0046942	carboxylic acid transport	7	0.02248633	7	0.004171845
GO:0000459	exonucleolytic trimming involved in rRNA processing	12	0.025328203	12	0.004348886
GO:0006220	pyrimidine nucleotide metabolic process	12	0.040757751	12	0.004348886
GO:0006221	pyrimidine nucleotide biosynthetic process	12	0.025328203	12	0.004348886
GO:0009112	nucleobase metabolic process	18	0.034205069	18	0.004509761

GO:0031669	cellular response to nutrient levels	10	0.029585504	10	0.004583377
GO:0046040	IMP metabolic process	9	0.023417265	9	0.004786071
GO:0043631	RNA polyadenylation	15	0.032161782	15	0.004893175
GO:0022904	respiratory electron transport chain	21	0.024471082	20	0.00508402
GO:0031124	mRNA 3'-end processing	21	0.024471082	20	0.00508402
GO:0042594	response to starvation	8	0.035145697	8	0.005417334
GO:0006289	nucleotide-excision repair	29	0.014643616	27	0.005436048
GO:0009064	glutamine family amino acid metabolic process	27	0.044678492	26	0.005485039
GO:0046031	ADP metabolic process	16	0.037776508	16	0.005485039
GO:0070481	nuclear-transcribed mRNA catabolic process non-stop decay	13	0.032797712	13	0.005485039
GO:0000973	posttranscriptional tethering of RNA polymerase II gene DNA at nuclear periphery	11	0.030225908	11	0.005596522
GO:0006563	L-serine metabolic process	8	0.025870359	8	0.005596522
GO:0009147	pyrimidine nucleoside triphosphate metabolic process	8	0.025870359	8	0.005596522
GO:0019856	pyrimidine nucleobase biosynthetic process	8	0.025870359	8	0.005596522
GO:0045040	protein insertion into mitochondrial outer membrane	8	0.025870359	8	0.005596522
GO:0071033	nuclear retention of pre-mRNA at the site of transcription	8	0.025870359	8	0.005596522
GO:0070873	regulation of glycogen metabolic process	4	0.029585504	4	0.006036014
GO:0009605	response to external stimulus	11	0.035405137	11	0.006036014
GO:0043255	regulation of carbohydrate biosynthetic process	5	0.0344604	5	0.006780364
GO:0140013	meiotic nuclear division	10	0.042255541	10	0.007120926
GO:0016180	snRNA processing	12	0.040757751	12	0.007357325
GO:0006740	NADPH regeneration	10	0.037380901	10	0.007384684
GO:0031667	response to nutrient levels	10	0.04643064	10	0.007414653

GO:0061013	regulation of mRNA catabolic process	42	2.3473E-06	16	0.008108544
GO:0032881	regulation of polysaccharide metabolic process	4	0.043057793	4	0.008329314
GO:0022900	electron transport chain	26	0.008485706	26	0.009207097
GO:0006119	oxidative phosphorylation	22	0.019673886	19	0.009434515
GO:0070127	tRNA aminoacylation for mitochondrial protein translation	9	0.044783457	9	0.009580447
GO:0031334	positive regulation of protein-containing complex assembly	28	0.022202078	26	0.011929401
GO:0034063	stress granule assembly	9	0.023417265	8	0.012260482
GO:0006376	mRNA splice site selection	9	0.023417265	9	0.017752193
GO:0019646	aerobic electron transport chain	20	0.016742888	10	0.02237036
GO:1903313	positive regulation of mRNA metabolic process	38	5.34447E-06	9	0.030435816
GO:0042773	ATP synthesis coupled electron transport	19	0.037776508	16	0.031280397
GO:0042775	mitochondrial ATP synthesis coupled electron transport	19	0.037776508	16	0.031280397
GO:0072593	reactive oxygen species metabolic process	12	0.015602118	11	0.038268613
GO:0045727	positive regulation of translation	23	0.021437395	19	0.048067267
