# Supplementary material for: Insights into the identification and evolutionary conservation of key genes in the transcriptional circuits of meiosis initiation and commitment in budding yeast
Source: FEBS Open Bio. 2023 Nov 14;13(12):2290–305. doi: 10.1002/2211-5463.13728 (PMC10699112; doi:10.1002/2211-5463.13728)
Supplement: Supplementary file 9 — File S3. Upregulated genes during meiotic commitment in S. cerevisiae. The upregulated genes are obtained from comparing transcriptome of non‐committed cells (2,3 and 4 h of growth in SPM) with committed cells (5,6 and 7 h of growth in SPM) of meiosis and then the genes that are downregulated on transferring committed cells to YPD were eliminated from the upregulated set of genes. The remaining genes are the genes for meiosis commitment. [file FEB4-13-2290-s002.pdf]

<u>Supplementary File 3-</u>* *Upregulated genes during meiotic commitment in S.cerevisiae. The upregulated genes are obtained from comparing transcriptome of non-committed cells (2,3 and 4 hours of growth in SPM) with committed cells (5,6 and 7 hours of growth in SPM) of meiosis and then the genes that are downregulated on transferring commited cells to YPD were eliminated from the upregulated set of genes. The remaining genes are the genes for meiosis commitment

**Upregulated Genes in GSE3815**		
**ORF**	**adj.P.Val**	**logFC**
YFL011W	0.000578	6.811577
YCR045C	0.008551	6.275923
YGL230C	0.00099	5.598323
YNL318C	0.0203	5.460751
YOR339C	0.012604	5.31175
YPR078C	0.021183	5.28191
YGR273C	0.010677	5.105714
YDR523C	0.032271	5.085623
YLR343W	0.020334	5.037485
YDR508C	0.004611	4.989387
YOL024W	0.009808	4.81246
YNL033W	0.015026	4.745473
YHR015W	0.014008	4.633173
YLR341W	0.022506	4.55845
YHR124W	0.039403	4.552023
YOL015W	0.005246	4.520296
YIL099W	0.006949	4.4877
YNL128W	0.011953	4.466423
YBR045C	0.012121	4.45867
YDR218C	0.031005	4.425243
YCR018C	0.002809	4.385837
YOL132W	0.018359	4.3205
YLR054C	0.013275	4.283703
YPR077C	0.049787	4.282183
YNL019C	0.025744	4.267201
YEL023C	0.028945	4.256481
YBR076W	0.013391	4.256277
YCL048W	0.037857	4.235025
YJL038C	0.048852	4.17684
YER085C	0.014949	4.165917
YDR042C	0.035407	4.139577
YGR108W	0.01258	4.11691
YHR185C	0.029801	4.07405
YGL170C	0.043661	4.06415
YKL189W	0.006156	4.061173
YBR148W	0.033362	4.045987
YNR034W	0.003258	4.027953

**Genes downregulated when transferred to YPD (GSE3816)**		
**ORF**	**adj.P.Val**	**logFC**
YIL114C	0.036879	-1.00026
YDR281C	0.045098	-1.03077
YIR004W	0.04182	-1.06916
YJR106W	0.004098	-1.07601
YLR018C	0.036432	-1.09167
YLR246W	0.013573	-1.1096
YJR039W	0.010129	-1.11026
YMR079W	0.004835	-1.11123
YIL113W	0.0313	-1.12591
YNL044W	0.006808	-1.1276
YFR039C	0.01345	-1.13411
YNL126W	0.012503	-1.15668
YLR220W	0.007811	-1.1569
YDR331W	0.019988	-1.15898
YNR026C	0.004835	-1.16436
YDR505C	0.018076	-1.18581
YDR065W	0.017175	-1.18891
YLR290C	0.046998	-1.1902
YIL074C	0.004108	-1.20773
YNL121C	0.007828	-1.22981
YDR219C	0.005843	-1.24698
YNL154C	0.020088	-1.24743
YML117W-A	0.007441	-1.27024
YDR191W	0.012883	-1.2781
YPL179W	0.042154	-1.27929
YHR185C	0.009393	-1.28307
YNL259C	0.0152	-1.29015
YBR168W	0.038053	-1.32903
YGL170C	0.027176	-1.34727
YDR218C	0.01348	-1.36433
YHR124W	0.019863	-1.36823
YDR479C	0.005277	-1.3689
YCR002C	0.010409	-1.37427
YFR028C	0.000933	-1.39202
YPR124W	0.001372	-1.42158
YMR054W	0.015071	-1.42257
YIL112W	0.008377	-1.44211

**Genes for Meiotic commitment**
YFL011W
YGL230C
YNL318C
YGR273C
YDR523C
YDR508C
YOL024W
YIL099W
YNL128W
YBR045C
YCR018C
YOL132W
YPR077C
YBR076W
YCL048W
YER085C
YDR042C
YKL189W
YNR034W
YOR190W
YOR365C
YDR371W
YLR013W
YDR438W
YMR326C
YPR140W
YNL204C
YLL005C
YDR403W
YKL042W
YDR402C
YOR355W
YFR005C
YDR326C
YLR031W
YML047C
YHL028W
YLR012C

YOR190W	0.031082	4.016267
YOR365C	0.005047	3.98661
YDR371W	0.027653	3.98192
YLR013W	0.003264	3.9345
YOL091W	0.048352	3.929685
YDL114W	0.012883	3.921531
YDR438W	0.005458	3.907547
YFL040W	0.010567	3.904157
YER180C	0.006932	3.884503
YMR326C	0.005104	3.884183
YBR018C	0.0243	3.883637
YNL018C	0.01258	3.855882
YPR140W	0.004221	3.839363
YNL204C	0.048675	3.818578
YGL015C	0.017588	3.735197
YKR015C	0.030853	3.722127
YLL005C	0.033772	3.721978
YGL116W	0.016876	3.718667
YDR403W	0.02657	3.6958
YDL247W	0.023592	3.69562
YKL042W	0.014008	3.6892
YNL034W	0.007486	3.684063
YBL084C	0.003827	3.667187
YDR402C	0.008619	3.659859
YOR355W	0.004221	3.64175
YFR005C	0.001723	3.626021
YDR326C	0.00716	3.588233
YJL043W	0.030766	3.56395
YLR031W	0.003264	3.529533
YFR012W	0.022699	3.49772
YLR084C	0.002809	3.484087
YML047C	0.010413	3.38619
YHL028W	0.002809	3.381043
YLR012C	0.019203	3.378066
YBR168W	0.005229	3.354277
YGR287C	0.002809	3.340277
YHL012W	0.002809	3.335157
YOR249C	0.003827	3.318633
YGL078C	0.003264	3.310157
YGR225W	0.033179	3.298707
YER115C	0.033888	3.29263
YML119W	0.041944	3.291013
YIL159W	0.01694	3.27329
YDR263C	0.006156	3.238633
YLR213C	0.020241	3.23233
YJR086W	0.018576	3.226763
YLR227C	0.014537	3.210493
YNL095C	0.003308	3.203197
YAR028W	0.005008	3.14502
YEL057C	0.007245	3.075631

YNL095C	0.016909	-1.45764
YNL188W	0.035268	-1.46865
YGL015C	0.026272	-1.49067
YBR148W	0.003139	-1.49496
YPR027C	0.000904	-1.49847
YNL026W	0.023692	-1.51402
YEL058W	0.027136	-1.52101
YJR099W	0.009445	-1.5242
YHR151C	0.004572	-1.53029
YPL204W	0.028897	-1.53237
YOR249C	0.011352	-1.53333
YJR017C	0.023274	-1.53647
YAL067C	0.02534	-1.54234
YLR318W	0.010129	-1.55177
YMR299C	0.003059	-1.56197
YOR301W	0.004815	-1.5858
YOR297C	0.001846	-1.58814
YDL103C	0.026353	-1.59107
YML034W	0.012043	-1.59509
YGR225W	0.003618	-1.5973
YGL116W	0.020833	-1.60237
YPL184C	0.012923	-1.62026
YOR180C	0.004143	-1.62438
YDR270W	0.004942	-1.63115
YLR368W	0.008332	-1.63935
YNL169C	0.012438	-1.64508
YJL038C	0.029408	-1.65037
YKL100C	0.014562	-1.65209
YDR147W	0.002222	-1.67963
YNL034W	0.016614	-1.68795
YNL054W	0.005754	-1.68876
YDR196C	0.007811	-1.68894
YER180C	0.039241	-1.71923
YGR015C	0.001244	-1.72147
YLL004W	0.043238	-1.72426
YNL172W	0.026276	-1.72515
YLR084C	0.001726	-1.72959
YNL018C	0.041302	-1.73059
YKR019C	0.043145	-1.73341
YDR104C	0.006818	-1.75244
YBR072W	0.005001	-1.76327
YLR271W	0.000422	-1.77541
YPR106W	0.011025	-1.7795
YOR372C	0.0152	-1.79363
YLR260W	0.016357	-1.82724
YER123W	0.009653	-1.83507
YNL223W	0.009756	-1.83535
YBR233W	0.004201	-1.83837
YMR047C	0.004008	-1.84106
YKL049C	0.001794	-1.84533

YGR287C
YHL012W
YGL078C
YER115C
YML119W
YEL057C
YKR007W
YNR014W
YDL055C
YBR071W
YDL135C
YKL190W
YDR496C
YLR049C
YLR195C
YDR177W
YDL241W
YOR081C
YFL017C
YDR454C
YLL029W
YLR314C
YMR313C
YDR516C
YJR119C
YIL037C
YBR069C
YPL192C
YDL076C
YHR052W
YNL219C
YNR077C
YPR079W
YDL122W
YHR020W
YDR448W
YLR265C
YDR103W
YKL022C
YDR239C
YDL051W
YHR061C
YMR272C
YJL212C
YLR380W
YCR054C
YKL167C
YGR202C
YLR072W
YPL167C

YKR007W	0.002809	3.060927
YNR014W	0.010034	3.05737
YBR268W	0.005104	3.032553
YDL055C	0.002996	3.03136
YNL172W	0.012195	3.001663
YFR036W	0.02518	2.973967
YBR071W	0.005104	2.969437
YDL135C	0.009835	2.953897
YIL112W	0.003346	2.937163
YGR109C	0.006514	2.909173
YLR368W	0.005008	2.896358
YLR127C	0.017527	2.881795
YKL190W	0.031082	2.880567
YNL225C	0.01387	2.875233
YDR496C	0.005104	2.871143
YDR273W	0.017221	2.867867
YDL079C	0.02279	2.862406
YLR049C	0.00716	2.857767
YLR195C	0.005852	2.856243
YPR027C	0.028945	2.854133
YDR177W	0.003346	2.85222
YDL241W	0.015169	2.848797
YOR081C	0.002809	2.844803
YFL017C	0.004209	2.787087
YDR454C	0.002577	2.783007
YLL029W	0.003264	2.781397
YLR314C	0.03107	2.776627
YDL028C	0.010506	2.772467
YMR313C	0.011079	2.7689
YDR104C	0.021332	2.763941
YDR065W	0.006156	2.762143
YDR516C	0.013057	2.75819
YOR032C	0.012781	2.757583
YOR301W	0.003264	2.757079
YJR119C	0.011645	2.756803
YOL135C	0.038946	2.746633
YIL037C	0.005104	2.741197
YLR220W	0.010033	2.739237
YBR069C	0.012682	2.738765
YPL192C	0.023128	2.73726
YDL076C	0.002809	2.735967
YGL162W	0.030749	2.732197
YGL003C	0.007486	2.73113
YNR026C	0.007011	2.72704
YLL021W	0.006509	2.724517
YHR052W	0.002809	2.723307
YOR152C	0.00687	2.693207
YNL154C	0.015584	2.6727
YLL004W	0.023255	2.668684
YNL219C	0.002809	2.642737

YLL021W	0.040749	-1.84673
YDL008W	0.02088	-1.85195
YHR150W	0.006808	-1.86144
YBL084C	0.005001	-1.86493
YDL239C	0.000395	-1.9024
YLR341W	0.020852	-1.91274
YPL253C	0.022782	-1.91369
YDR273W	0.023454	-1.92377
YGR109C	0.011284	-1.94263
YBR268W	0.013948	-1.94587
YAR028W	0.011935	-1.96603
YLR127C	0.009751	-1.9676
YLR227C	0.010742	-1.9741
YDR539W	0.013936	-1.98273
YBL079W	0.007095	-1.99458
YLR356W	0.006165	-1.99579
YKL133C	0.000904	-2.00546
YLR054C	0.004725	-2.01223
YNL270C	0.005001	-2.01239
YFR036W	0.013906	-2.0196
YCR045C	0.007441	-2.02123
YHR015W	0.038669	-2.0296
YGR108W	0.001811	-2.04237
YOL091W	0.001133	-2.0705
YBL009W	0.005398	-2.12691
YIL159W	0.02184	-2.13093
YDR118W	0.02588	-2.14599
YFR012W	0.014562	-2.15367
YOR032C	0.004397	-2.17411
YDL115C	0.00123	-2.17704
YDL079C	0.001572	-2.23611
YDR356W	0.002625	-2.23969
YGL003C	0.008557	-2.25399
YPL178W	0.007843	-2.2567
YPL255W	0.008932	-2.26962
YOR212W	0.004108	-2.30769
YFL040W	0.001839	-2.35477
YDL114W	0.025815	-2.36681
YGL162W	0.003418	-2.3737
YIL146C	0.009597	-2.44207
YOL015W	0.001931	-2.459
YNR007C	0.010918	-2.48698
YOL135C	0.020112	-2.5089
YLR102C	0.004232	-2.53636
YDR263C	0.001244	-2.55047
YOR177C	0.00198	-2.58234
YIL155C	0.011723	-2.58712
YNL019C	0.023361	-2.59186
YPR120C	0.003986	-2.61255
YNL225C	0.006913	-2.64195

YPR122W
YOL146W
YLL065W
YGR088W
YIL045W
YGL250W
YKR089C
YGR185C
YML035C
YFL024C
YOR091W
YPR110C
YGR196C
YIL065C
YBR025C
YPL170W
YGR272C
YCR034W
YOR089C
YOR182C
YER100W
YLL008W
YLR372W
YAL026C
YDL194W
YNL217W
YER075C
YLR093C
YHR142W
YNR058W
YER155C
YML025C
YMR071C
YLL032C
YBR282W
YDR480W
YFL022C
YAR029W
YGR066C
YPL046C
YFL041W
YPL049C
YOR115C
YHR036W
YDR157W
YKR100C
YOR286W
YCR035C
YBR285W
YAR003W

YNR077C	0.028755	2.641127
YPR079W	0.016169	2.617173
YDL122W	0.017771	2.60279
YLR030W	0.017899	2.59974
YHR020W	0.012847	2.581343
YDR448W	0.004221	2.573743
YDR281C	0.02597	2.550223
YLR265C	0.002809	2.548427
YDR103W	0.010413	2.54177
YKL022C	0.03724	2.533324
YDL239C	0.009501	2.526767
YDR239C	0.013186	2.525357
YDL051W	0.00362	2.514105
YHR061C	0.032013	2.512723
YMR272C	0.006156	2.503573
YJL212C	0.004733	2.490933
YIR004W	0.007222	2.49038
YLR380W	0.006067	2.485248
YFR039C	0.004611	2.476583
YCR054C	0.004221	2.469443
YKL167C	0.003712	2.469103
YGR202C	0.013186	2.465867
YLR072W	0.008619	2.43036
YPL167C	0.003827	2.429893
YPR122W	0.048176	2.426053
YAL067C	0.013391	2.414073
YOL146W	0.005104	2.412307
YLL065W	0.033068	2.402942
YMR079W	0.012727	2.393755
YGR088W	0.026675	2.381995
YIL045W	0.006156	2.373897
YGL250W	0.008931	2.371643
YKR089C	0.015019	2.36933
YDL008W	0.02067	2.362257
YGR185C	0.002809	2.36061
YML035C	0.003308	2.36021
YFL024C	0.01258	2.35432
YIL074C	0.014864	2.341377
YPL255W	0.02034	2.338327
YOR091W	0.005008	2.327477
YPR110C	0.00739	2.32256
YML034W	0.031573	2.31448
YGR196C	0.007245	2.31344
YIL111W	0.018576	2.29575
YDR196C	0.005583	2.289033
YIL065C	0.005008	2.28529
YKR019C	0.002809	2.282183
YBR025C	0.004358	2.282113
YPL170W	0.022269	2.280857
YNL270C	0.023359	2.27881

YLR030W	0.015071	-2.65125
YLR343W	0.002939	-2.6524
YDL247W	0.006138	-2.65476
YJL043W	0.005943	-2.66017
YIL139C	0.003687	-2.69272
YPR078C	0.00145	-2.72507
YKR015C	0.003077	-2.72758
YOR090C	0.001761	-2.73758
YDL028C	0.002494	-2.75558
YIL111W	0.009108	-2.77442
YJR086W	0.00555	-2.86196
YLR213C	0.004673	-2.89228
YMR184W	0.004265	-2.92343
YOR152C	0.011057	-3.08706
YLR045C	0.001187	-3.30586
YEL023C	0.001374	-3.36905
YNL033W	0.004399	-3.40724
YBR018C	0.003952	-3.53441
YOR339C	0.001399	-4.48103

YGR055W
YGR229C
YDL024C
YBR166C
YIL048W
YGR266W
YDR370C
YIL103W
YOL041C
YOR323C
YOR279C
YNL070W
YKL183W
YJR052W
YGR043C
YER132C
YGR238C
YKL094W
YNL155W
YDR380W
YML130C
YMR114C
YBR068C
YHR166C
YDL026W
YLR138W
YHR183W
YPR159W
YKR066C
YER092W
YGL248W
YKR031C
YKR085C
YDR487C
YCL010C
YDL088C
YBR179C
YIL038C
YMR243C
YIL055C
YMR260C
YER076C
YBR150C
YOR360C
YEL005C
YNL014W
YJL216C
YEL046C
YJL016W
YBR157C

YGR272C	0.00675	2.275037
YCR034W	0.00716	2.273437
YJR099W	0.005047	2.270233
YIL139C	0.029148	2.25397
YOR089C	0.006156	2.252227
YOR182C	0.047889	2.247513
YER100W	0.005104	2.236303
YLL008W	0.04446	2.22321
YEL058W	0.006156	2.209863
YKL133C	0.014004	2.20773
YLR372W	0.033362	2.204853
YHR150W	0.007738	2.20485
YER123W	0.043091	2.188237
YAL026C	0.007245	2.18754
YDR191W	0.017372	2.166957
YDL194W	0.008619	2.16378
YNL217W	0.008572	2.163527
YPL204W	0.033372	2.16221
YER075C	0.012883	2.152623
YLR093C	0.004831	2.149913
YHR142W	0.00289	2.147727
YNR058W	0.022177	2.139023
YDR331W	0.013186	2.133737
YKL100C	0.005008	2.132799
YER155C	0.011278	2.130533
YML025C	0.009202	2.122493
YOR212W	0.041719	2.121836
YMR071C	0.008931	2.115337
YLL032C	0.033362	2.114837
YBR282W	0.004221	2.112411
YDR480W	0.005652	2.095133
YFL022C	0.010384	2.089007
YAR029W	0.006156	2.088577
YGR066C	0.019441	2.08744
YPL046C	0.004507	2.08096
YFL041W	0.01987	2.077847
YPL049C	0.006156	2.072937
YOR115C	0.002841	2.072133
YPL178W	0.030389	2.071443
YHR036W	0.012121	2.06911
YDR157W	0.013049	2.065899
YKR100C	0.01258	2.06543
YLR045C	0.047014	2.048
YCR002C	0.031788	2.04757
YOR286W	0.01765	2.040653
YHR151C	0.00739	2.039378
YCR035C	0.006156	2.033493
YOR180C	0.004358	2.02297
YBR285W	0.029204	2.0058
YAR003W	0.005237	2.003847

YKR093W
YDR117C
YBR167C
YCL054W
YDL236W
YER166W
YDR146C
YPL245W
YIL117C
YIL161W
YLR214W
YBR264C
YKR004C
YMR281W
YJR055W
YML091C
YDL030W
YDR543C
YNR055C
YMR252C
YOR267C
YKL121W
YJL104W
YKL096W
YOR165W
YKL168C
YNL153C
YGL231C
YDR472W
YER149C
YJR110W
YKR002W
YLR209C
YIL104C
YCL011C
YBL051C
YHR179W
YML097C
YDL065C
YMR211W
YFR006W
YBR023C
YDR449C
YHR115C
YEL074W
YBR260C
YGR072W
YDR253C
YDL141W
YJR061W

YLR018C	0.014351	1.999853
YGR055W	0.005583	1.99543
YGR229C	0.042631	1.991911
YDL024C	0.012296	1.986957
YBR166C	0.015899	1.97661
YIL048W	0.023208	1.971367
YGR266W	0.021049	1.961689
YDR370C	0.005008	1.95697
YIL103W	0.006089	1.93718
YOL041C	0.014101	1.936963
YOR323C	0.003264	1.935027
YOR279C	0.004221	1.93096
YNL070W	0.009808	1.928867
YKL183W	0.049474	1.926325
YOR297C	0.011308	1.921937
YNL188W	0.032879	1.91741
YJR052W	0.00716	1.915261
YGR043C	0.01709	1.91087
YER132C	0.028945	1.91073
YGR238C	0.015899	1.90414
YKL094W	0.003524	1.901337
YNL155W	0.017935	1.899
YDR479C	0.005008	1.897232
YDR380W	0.016846	1.89425
YML130C	0.020172	1.887913
YMR114C	0.006156	1.88681
YBR068C	0.048741	1.88654
YHR166C	0.040687	1.8842
YDL026W	0.047989	1.88384
YLR138W	0.003346	1.880907
YDR270W	0.005104	1.877387
YHR183W	0.032271	1.875393
YLR260W	0.03899	1.865973
YPR159W	0.010252	1.865796
YKR066C	0.009048	1.862306
YER092W	0.011953	1.860073
YGL248W	0.006509	1.852087
YMR184W	0.033372	1.84087
YKR031C	0.013391	1.83731
YKR085C	0.003264	1.830143
YDR487C	0.006156	1.822531
YCL010C	0.018125	1.820308
YNL223W	0.02034	1.813907
YDL088C	0.008622	1.80924
YBR179C	0.008931	1.808473
YNL121C	0.016042	1.796337
YNL026W	0.00716	1.794554
YIL038C	0.008931	1.794112
YMR243C	0.006156	1.783013
YIL055C	0.017899	1.78265

YNL053W
YNL238W
YOL130W
YGL211W
YFL067W
YDL148C
YKL148C
YPL174C
YGL171W
YGR038W
YDR158W
YKR102W
YJL198W
YNL124W
YBL109W
YPL159C
YKL032C
YHR084W
YDR240C
YKL138C
YNL268W
YDR115W
YDR533C
YIL106W
YPL030W
YEL004W
YDR119W
YGL181W
YFL031W
YNL096C
YKL122C
YJL133W
YPR022C
YBL095W
YDR041W
YBR155W
YMR193W
YBR091C
YGL106W
YER168C
YML023C
YOR250C
YIR035C
YMR082C
YDR515W
YDR504C
YBR243C
YER038C
YMR146C
YNL039W

YMR260C	0.012121	1.7732
YER076C	0.035236	1.771215
YMR054W	0.009877	1.76974
YBR150C	0.04037	1.76777
YOR360C	0.004221	1.743235
YEL005C	0.004507	1.736593
YDR118W	0.016674	1.724897
YNL014W	0.035481	1.72183
YOR177C	0.041858	1.718717
YJL216C	0.006089	1.718353
YEL046C	0.009808	1.718069
YJL016W	0.02084	1.715737
YBR157C	0.02279	1.715653
YNR007C	0.025487	1.709737
YKR093W	0.007354	1.70504
YDR117C	0.011142	1.704973
YBR167C	0.019973	1.700047
YCL054W	0.00716	1.698447
YFR028C	0.008551	1.697633
YDL236W	0.006156	1.69465
YER166W	0.012928	1.692077
YIL113W	0.01616	1.681964
YDR146C	0.010082	1.679049
YPL245W	0.040302	1.67446
YIL117C	0.012937	1.66503
YIL161W	0.005147	1.664273
YLR214W	0.015899	1.662713
YBR264C	0.007245	1.66245
YKR004C	0.006089	1.65203
YIL114C	0.01159	1.652017
YMR281W	0.00739	1.64932
YJR055W	0.013391	1.647117
YML091C	0.016674	1.640749
YDL030W	0.007245	1.635627
YDR543C	0.020834	1.633843
YNR055C	0.014694	1.628934
YMR252C	0.010858	1.62143
YOR267C	0.010384	1.607463
YDR356W	0.045902	1.606242
YKL121W	0.046585	1.603743
YDL103C	0.008619	1.6032
YJL104W	0.00687	1.59476
YDR219C	0.007245	1.593897
YKL096W	0.044041	1.588172
YOR165W	0.008931	1.58607
YKL168C	0.019106	1.585834
YNL153C	0.01258	1.583367
YGL231C	0.00716	1.582063
YDR472W	0.01159	1.581778
YER149C	0.016042	1.578993

YLR355C
YDR395W
YLR463C
YOR187W
YDL235C
YJL187C
YKL120W
YPL126W
YHR081W
YAL055W
YDL062W
YOR112W
YNR070W
YMR200W
YOR233W
YER041W
YER103W
YIL002C
YML118W
YEL045C
YAL043C
YLR205C
YLR266C
YGR071C
YDR333C
YKR095W
YPL072W
YDL246C
YAL068C
YFL023W
YOL056W
YLR061W
YML103C
YDL001W
YML010W-A
YOR161C
YER165W
YDR493W
YDR311W
YDR268W
YPL149W
YGL220W
YOR232W
YGR247W
YGR110W
YJL200C
YGL112C
YER087C-A
YOR172W
YOR306C

YJR110W	0.044303	1.577037
YKR002W	0.020654	1.57328
YDR505C	0.021186	1.558861
YNL044W	0.009202	1.556367
YLR209C	0.042088	1.54818
YIL104C	0.00716	1.545923
YCL011C	0.019319	1.540414
YBL051C	0.011252	1.53884
YHR179W	0.010303	1.531013
YML097C	0.030749	1.529071
YDL065C	0.012336	1.52854
YMR211W	0.016674	1.521253
YPL253C	0.023153	1.51956
YFR006W	0.023751	1.51791
YPR120C	0.012195	1.512667
YBR023C	0.009563	1.505392
YDR449C	0.010817	1.504073
YHR115C	0.016169	1.502377
YMR047C	0.011308	1.501511
YEL074W	0.012604	1.498787
YBR260C	0.044763	1.495567
YGR072W	0.02084	1.491529
YDR253C	0.020582	1.484165
YDL141W	0.028561	1.47993
YJR061W	0.008931	1.47894
YNL053W	0.023264	1.476353
YNL238W	0.031164	1.475616
YLR246W	0.028989	1.474997
YOL130W	0.006156	1.466597
YGL211W	0.017899	1.464397
YBR233W	0.043661	1.463833
YFL067W	0.011161	1.459849
YNL126W	0.022699	1.455493
YDL148C	0.011028	1.45476
YKL148C	0.02034	1.450917
YPL174C	0.01258	1.446603
YGL171W	0.008638	1.446597
YGR038W	0.038548	1.445293
YDR158W	0.029192	1.44431
YKR102W	0.013279	1.441293
YJL198W	0.023466	1.439369
YNL124W	0.008638	1.439287
YBL109W	0.02168	1.438987
YPL159C	0.030389	1.42951
YKL032C	0.013391	1.425054
YHR084W	0.022403	1.423997
YDR240C	0.031929	1.420578
YKL138C	0.026675	1.41447
YNL268W	0.016169	1.41317
YLR102C	0.009493	1.411767

YBR122C
YBR114W
YDR082W
YDR457W
YCR051W
YEL076W-C
YOR346W
YHR206W
YDL089W
YML117W
YOR278W
YOR175C
YOR151C
YJL161W
YGR081C
YLR465C
YKL173W
YOR045W
YLR188W
YMR293C
YOR251C
YLL064C
YGR049W
YHR176W
YDR181C
YOR327C
YLR275W
YOR156C
YDR490C
YIL140W
YKL178C
YIR020C
YBL088C
YHR213W
YKL043W
YHR009C
YER133W
YDR372C
YDR264C
YLR020C
YBR128C
YNR039C
YER116C
YPL254W
YHL017W
YGR079W
YDL009C
YDR066C
YOR367W
YDR175C

YLR271W	0.010303	1.404767
YBL009W	0.01008	1.401343
YDR115W	0.023359	1.399791
YDR533C	0.01258	1.3957
YIL106W	0.031526	1.385687
YPL030W	0.012121	1.385147
YEL004W	0.033372	1.38242
YDR119W	0.013186	1.381278
YGL181W	0.01159	1.380447
YFL031W	0.01765	1.375483
YNL096C	0.02168	1.373623
YKL122C	0.015368	1.370227
YJL133W	0.022886	1.370154
YPR022C	0.01258	1.369973
YBL095W	0.012781	1.361837
YJR106W	0.016169	1.355743
YDR041W	0.012937	1.355483
YBR155W	0.008638	1.351781
YMR193W	0.010763	1.351243
YOR372C	0.023466	1.35111
YBR091C	0.009481	1.350333
YNL169C	0.009931	1.3455
YGL106W	0.007921	1.336708
YER168C	0.016674	1.33223
YML023C	0.008436	1.330381
YOR250C	0.010026	1.329233
YIR035C	0.00739	1.321573
YMR082C	0.019623	1.320918
YDR515W	0.039924	1.320137
YDR504C	0.02488	1.319659
YBR243C	0.033494	1.318047
YDR147W	0.020654	1.316653
YPL184C	0.016674	1.315233
YER038C	0.035686	1.31487
YMR146C	0.023218	1.314313
YNL039W	0.015899	1.30906
YLR355C	0.013391	1.308295
YDL115C	0.04624	1.305497
YDR395W	0.010413	1.303657
YLR463C	0.011252	1.299567
YOR187W	0.020654	1.298993
YDL235C	0.012928	1.297834
YJL187C	0.049326	1.29702
YKL120W	0.02328	1.29462
YPL126W	0.012883	1.294593
YGR015C	0.01885	1.293713
YHR081W	0.031573	1.292007
YAL055W	0.015026	1.291663
YDL062W	0.018435	1.290856
YOR112W	0.012883	1.281676

YER016W
YPL208W
YKL170W
YJL215C
YJR127C
YPR101W
YIL150C
YGR153W
YDL245C
YOR013W
YLR126C
YOL080C
YGR156W
YLR407W
YHL013C
YJL140W
YBR141C
YML105C
YOR106W
YBL021C
YGL143C
YJL017W

YNR070W	0.029121	1.276883
YMR200W	0.017899	1.276813
YMR299C	0.009076	1.276233
YOR233W	0.047064	1.275213
YER041W	0.012812	1.274062
YER103W	0.022487	1.271496
YIL002C	0.022497	1.270283
YML118W	0.013391	1.269367
YEL045C	0.020539	1.264288
YAL043C	0.016674	1.262719
YLR205C	0.022487	1.257921
YLR266C	0.031164	1.25737
YGR071C	0.016674	1.256707
YIL146C	0.022701	1.256033
YLR356W	0.011771	1.255433
YDR333C	0.008551	1.255013
YKR095W	0.042428	1.253277
YPL072W	0.010506	1.252493
YOR090C	0.02681	1.24969
YDL246C	0.028433	1.24886
YAL068C	0.026503	1.245027
YFL023W	0.018918	1.242677
YOL056W	0.018152	1.24163
YLR061W	0.010384	1.2398
YML103C	0.011645	1.236737
YDL001W	0.014949	1.235616
YML010W-A	0.023735	1.23343
YLR318W	0.021575	1.232963
YOR161C	0.012727	1.22972
YER165W	0.045902	1.2254
YDR493W	0.012121	1.223707
YDR311W	0.008619	1.220507
YDR268W	0.013391	1.2171
YPL149W	0.012604	1.216047
YGL220W	0.012773	1.214727
YOR232W	0.015026	1.214405
YGR247W	0.03405	1.19792
YGR110W	0.03396	1.195847
YJL200C	0.031382	1.19132
YGL112C	0.010039	1.190181
YER087C-A	0.0243	1.1776
YOR172W	0.049326	1.173303
YOR306C	0.047444	1.172367
YBR122C	0.010567	1.171283
YBR114W	0.030452	1.171257
YDR082W	0.014046	1.171052
YDR457W	0.038009	1.170361
YCR051W	0.012647	1.169873
YEL076W-C	0.013898	1.1697
YOR346W	0.009202	1.16712

YHR206W	0.04692	1.166077
YDL089W	0.023359	1.163217
YML117W	0.041944	1.15933
YOR278W	0.043424	1.158647
YOR175C	0.025922	1.1571
YOR151C	0.018576	1.156457
YJL161W	0.043661	1.156167
YNL054W	0.031005	1.15358
YLR290C	0.045902	1.153225
YGR081C	0.020172	1.15184
YIL155C	0.02168	1.149405
YLR465C	0.043864	1.148463
YKL173W	0.023466	1.146303
YOR045W	0.017826	1.14458
YKL049C	0.037392	1.143433
YLR188W	0.049321	1.139947
YBR072W	0.016674	1.134843
YMR293C	0.0243	1.134014
YBL079W	0.037857	1.1273
YOR251C	0.025274	1.12652
YML117W-A	0.025683	1.125147
YLL064C	0.018045	1.121707
YGR049W	0.045902	1.121498
YHR176W	0.026997	1.120067
YDR181C	0.026895	1.11828
YPL179W	0.015019	1.116701
YOR327C	0.013186	1.114749
YLR275W	0.018683	1.114203
YOR156C	0.046042	1.111622
YDR490C	0.03246	1.10847
YIL140W	0.02657	1.098234
YDR539W	0.016561	1.0976
YKL178C	0.039403	1.089333
YIR020C	0.041933	1.087633
YBL088C	0.028945	1.086433
YHR213W	0.042439	1.080703
YKL043W	0.03405	1.080532
YPR124W	0.035407	1.07872
YHR009C	0.018745	1.07724
YER133W	0.018384	1.072994
YDR372C	0.022763	1.072666
YDR264C	0.018576	1.07147
YLR020C	0.020172	1.07122
YBR128C	0.01245	1.071201
YPR106W	0.045902	1.0664
YNR039C	0.0279	1.066003
YER116C	0.046695	1.065347
YPL254W	0.036208	1.065298
YHL017W	0.02032	1.06446
YGR079W	0.030766	1.06046

YJR017C	0.02865	1.059913
YDL009C	0.016905	1.057434
YDR066C	0.027236	1.054457
YOR367W	0.023644	1.053773
YJR039W	0.025618	1.053203
YDR175C	0.035095	1.052183
YER016W	0.033372	1.051537
YPL208W	0.040062	1.048033
YKL170W	0.014366	1.047449
YJL215C	0.049326	1.047073
YJR127C	0.019894	1.046832
YPR101W	0.016846	1.044109
YIL150C	0.047944	1.042463
YGR153W	0.021575	1.038453
YDL245C	0.040062	1.037957
YOR013W	0.03561	1.03733
YLR126C	0.023048	1.034533
YNL259C	0.035095	1.03274
YOL080C	0.040716	1.028043
YGR156W	0.030423	1.024317
YLR407W	0.024904	1.02279
YHL013C	0.015109	1.021661
YJL140W	0.036597	1.015225
YBR141C	0.035419	1.00772
YML105C	0.026849	1.006931
YOR106W	0.030389	1.005037
YBL021C	0.017743	1.004527
YGL143C	0.025539	1.002101
YJL017W	0.021774	1.000047
