# Supplementary material for: Insights into the identification and evolutionary conservation of key genes in the transcriptional circuits of meiosis initiation and commitment in budding yeast
Source: FEBS Open Bio. 2023 Nov 14;13(12):2290–305. doi: 10.1002/2211-5463.13728 (PMC10699112; doi:10.1002/2211-5463.13728)
Supplement: Supplementary file 10 — File S4. GO Biological Process Enrichment of PPINcommit. [file FEB4-13-2290-s005.pdf]

Supplementary File 4 - GO Biological Process Enrichment of PPIN $_{commit}$					
**GO**	**Description**	**Count Metascape**	**FDR Metascape**	**Count Cluster profiler**	**p.adjust Cluster Profiler**
GO:0051128	regulation of cellular component organization	312	4.60E-31	316	6.34E-40
GO:0006974	cellular response to DNA damage stimulus	260	1.06E-26	262	7.10E-34
GO:0006325	chromatin organization	257	2.27E-26	259	7.10E-34
GO:0033043	regulation of organelle organization	213	1.10E-24	221	7.10E-34
GO:0044265	cellular macromolecule catabolic process	309	5.38E-24	310	5.32E-32
GO:0006281	DNA repair	229	1.26E-25	229	6.80E-32
GO:0051603	proteolysis involved in cellular protein catabolic process	202	6.23E-24	203	2.93E-30
GO:0010498	proteasomal protein catabolic process	141	2.27E-24	143	4.81E-30
GO:0044257	cellular protein catabolic process	212	1.72E-22	213	6.33E-29
GO:0002181	cytoplasmic translation	166	1.12E-24	165	1.35E-28
GO:0043632	modification-dependent macromolecule catabolic process	200	4.12E-23	198	2.49E-28
GO:0019941	modification-dependent protein catabolic process	187	2.49E-23	185	2.53E-28
GO:0044087	regulation of cellular component biogenesis	156	1.32E-22	161	5.19E-28
GO:0030163	protein catabolic process	216	1.78E-21	217	5.19E-28
GO:0043161	proteasome-mediated ubiquitin-dependent protein catabolic process	132	1.58E-23	132	5.52E-28
GO:0051726	regulation of cell cycle	212	3.58E-19	220	8.39E-28
GO:0006511	ubiquitin-dependent protein catabolic process	181	5.99E-22	179	9.67E-27
GO:0007010	cytoskeleton organization	205	5.82E-21	204	2.05E-26
GO:0016569	covalent chromatin modification	122	2.24E-21	123	9.19E-26
GO:0016570	histone modification	122	2.24E-21	123	9.19E-26
GO:0006260	DNA replication	126	6.64E-21	126	4.97E-25
GO:0007059	chromosome segregation	171	1.03E-20	169	7.77E-25
GO:0071826	ribonucleoprotein complex subunit organization	147	3.14E-21	149	3.37E-24

GO:0010564	regulation of cell cycle process	183	2.55E-19	169	4.24E-24
GO:0098813	nuclear chromosome segregation	149	1.82E-20	147	4.34E-24
GO:0051130	positive regulation of cellular component organization	133	1.45E-18	134	1.74E-23
GO:0000819	sister chromatid segregation	123	1.28E-19	124	1.92E-23
GO:0000280	nuclear division	189	2.00E-18	190	2.79E-23
GO:0022618	ribonucleoprotein complex assembly	142	1.96E-20	144	2.84E-23
GO:0048285	organelle fission	195	3.20E-18	197	4.01E-23
GO:0071103	DNA conformation change	142	3.73E-16	152	1.23E-22
GO:0043254	regulation of protein-containing complex assembly	90	7.52E-19	91	1.87E-22
GO:0006397	mRNA processing	164	1.03E-17	164	4.01E-22
GO:0010638	positive regulation of organelle organization	102	5.33E-17	103	6.44E-22
GO:0000398	mRNA splicing, via spliceosome	101	2.50E-18	102	1.32E-21
GO:0000377	RNA splicing, via transesterification reactions with bulged adenosine as nucleophile	101	8.93E-18	102	4.82E-21
GO:0097549	chromatin organization involved in negative regulation of transcription	122	4.35E-17	123	7.15E-21
GO:0097435	supramolecular fiber organization	100	5.59E-17	99	1.09E-20
GO:0006261	DNA-dependent DNA replication	109	6.66E-17	109	1.45E-20
GO:0051640	organelle localization	156	1.31E-16	155	1.45E-20
GO:0034401	chromatin organization involved in regulation of transcription	122	1.08E-16	123	1.87E-20
GO:0044770	cell cycle phase transition	165	2.83E-18	139	5.36E-20
GO:0006323	DNA packaging	106	3.82E-16	107	5.42E-20
GO:0045786	negative regulation of cell cycle	107	6.61E-14	111	9.77E-20
GO:0006333	chromatin assembly or disassembly	112	4.56E-15	113	6.00E-19
GO:0000375	RNA splicing, via transesterification reactions	104	1.27E-15	105	6.27E-19
GO:0070925	organelle assembly	147	1.11E-14	149	9.84E-19
GO:0031334	positive regulation of protein-containing complex assembly	59	1.20E-15	60	1.92E-18
GO:0006913	nucleocytoplasmic transport	138	1.24E-14	135	2.95E-18

GO:0051169	nuclear transport	138	1.24E-14	135	2.95E-18
GO:0031497	chromatin assembly	95	1.01E-14	96	3.09E-18
GO:0051129	negative regulation of cellular component organization	105	1.28E-14	104	3.40E-18
GO:0044089	positive regulation of cellular component biogenesis	79	4.54E-15	81	5.08E-18
GO:0018193	peptidyl-amino acid modification	176	1.97E-13	174	7.95E-18
GO:0008380	RNA splicing	116	3.95E-14	117	1.28E-17
GO:0010948	negative regulation of cell cycle process	103	3.97E-14	83	1.72E-17
GO:0010639	negative regulation of organelle organization	89	4.00E-14	88	2.13E-17
GO:0051258	protein polymerization	60	4.56E-15	61	5.20E-17
GO:0016567	protein ubiquitination	112	6.94E-13	113	6.08E-17
GO:0070647	protein modification by small protein conjugation or removal	155	1.93E-12	155	7.67E-17
GO:0006403	RNA localization	116	7.99E-12	137	8.53E-17
GO:0006302	double-strand break repair	109	2.93E-13	108	8.60E-17
GO:0033044	regulation of chromosome organization	85	3.11E-12	85	5.19E-16
GO:0006475	internal protein amino acid acetylation	58	1.12E-13	58	5.22E-16
GO:0018393	internal peptidyl-lysine acetylation	58	1.12E-13	58	5.22E-16
GO:0018394	peptidyl-lysine acetylation	58	1.12E-13	58	5.22E-16
GO:0017038	protein import	101	6.95E-13	100	7.87E-16
GO:0046034	ATP metabolic process	84	8.83E-15	79	9.16E-16
GO:0051493	regulation of cytoskeleton organization	77	6.08E-13	79	9.16E-16
GO:0016573	histone acetylation	57	2.22E-13	57	1.11E-15
GO:0000226	microtubule cytoskeleton organization	86	1.79E-12	88	1.67E-15
GO:0070828	heterochromatin organization	76	1.09E-12	76	2.11E-15
GO:0006906	vesicle fusion	47	6.95E-13	45	2.63E-15
GO:0007017	microtubule-based process	91	4.53E-12	93	3.59E-15
GO:0050657	nucleic acid transport	100	2.51E-10	121	7.82E-15
GO:0006473	protein acetylation	63	2.00E-12	63	7.93E-15
GO:0031503	protein-containing complex localization	120	1.82E-11	117	7.93E-15
GO:0016458	gene silencing	93	8.92E-12	96	8.89E-15

GO:0006342	chromatin silencing	90	7.89E-12	91	1.24E-14
GO:0045814	negative regulation of gene expression, epigenetic	90	7.89E-12	91	1.24E-14
GO:0007015	actin filament organization	72	3.79E-12	71	1.93E-14
GO:0031507	heterochromatin assembly	71	7.07E-12	71	1.93E-14
GO:0030036	actin cytoskeleton organization	111	4.41E-11	110	1.96E-14
GO:0050658	RNA transport	98	6.57E-10	119	2.20E-14
GO:0051236	establishment of RNA localization	98	6.57E-10	119	2.20E-14
GO:0030029	actin filament-based process	112	5.27E-11	111	2.33E-14
GO:0040029	regulation of gene expression, epigenetic	90	1.86E-11	91	3.00E-14
GO:0018205	peptidyl-lysine modification	104	7.69E-11	104	5.97E-14
GO:0032446	protein modification by small protein conjugation	126	2.93E-10	126	6.10E-14
GO:0006338	chromatin remodeling	115	4.90E-11	113	7.04E-14
GO:0006270	DNA replication initiation	49	9.24E-12	48	1.86E-13
GO:0051168	nuclear export	101	7.88E-11	99	2.22E-13
GO:0043543	protein acylation	67	7.41E-11	67	2.99E-13
GO:0032271	regulation of protein polymerization	47	3.71E-11	47	4.09E-13
GO:0051656	establishment of organelle localization	99	4.84E-11	96	5.74E-13
GO:0006611	protein export from nucleus	12	4.08E-02	96	5.74E-13
GO:0016050	vesicle organization	78	1.44E-10	77	6.91E-13
GO:0051052	regulation of DNA metabolic process	78	1.44E-10	77	6.91E-13
GO:0048583	regulation of response to stimulus	128	1.18E-09	131	6.91E-13
GO:0071166	ribonucleoprotein complex localization	97	1.31E-10	94	8.14E-13
GO:0071824	protein-DNA complex subunit organization	125	6.93E-09	127	8.38E-13
GO:0090174	organelle membrane fusion	48	9.90E-11	46	8.55E-13
GO:1902903	regulation of supramolecular fiber organization	54	1.56E-10	54	1.25E-12
GO:0006405	RNA export from nucleus	73	4.84E-09	94	1.74E-12
GO:0019693	ribose phosphate metabolic process	93	6.94E-09	95	2.11E-12
GO:0071426	ribonucleoprotein complex export from nucleus	95	3.35E-10	92	2.50E-12
GO:0065004	protein-DNA complex assembly	93	6.94E-09	94	3.66E-12
GO:0010646	regulation of cell communication	104	3.89E-09	103	3.90E-12

GO:0051495	positive regulation of cytoskeleton organization	52	5.35E-10	52	5.22E-12
GO:0032956	regulation of actin cytoskeleton organization	56	1.74E-09	57	7.55E-12
GO:0032970	regulation of actin filament-based process	56	1.74E-09	57	7.55E-12
GO:0045787	positive regulation of cell cycle	71	6.00E-09	73	7.99E-12
GO:0023051	regulation of signaling	100	6.85E-09	99	8.35E-12
GO:1902905	positive regulation of supramolecular fiber organization	43	5.36E-10	43	8.40E-12
GO:0061025	membrane fusion	51	3.55E-09	51	1.04E-11
GO:0000725	recombinational repair	71	2.52E-09	71	1.06E-11
GO:0009966	regulation of signal transduction	99	1.07E-08	98	1.40E-11
GO:0032273	positive regulation of protein polymerization	37	8.64E-10	37	1.95E-11
GO:0006275	regulation of DNA replication	47	3.43E-09	48	2.23E-11
GO:0031570	DNA integrity checkpoint signaling	46	6.47E-09	48	2.23E-11
GO:0000724	double-strand break repair via homologous recombination	65	4.54E-09	65	2.57E-11
GO:0009259	ribonucleotide metabolic process	87	5.84E-08	89	2.70E-11
GO:0006163	purine nucleotide metabolic process	84	1.70E-08	86	3.29E-11
GO:0032984	protein-containing complex disassembly	69	1.70E-08	69	3.61E-11
GO:0051783	regulation of nuclear division	56	1.50E-08	56	4.61E-11
GO:0016236	macroautophagy	100	4.04E-08	99	5.99E-11
GO:0006119	oxidative phosphorylation	43	1.23E-11	38	6.40E-11
GO:0006348	chromatin silencing at telomere	65	2.68E-08	65	1.71E-10
GO:0030466	silent mating-type cassette heterochromatin assembly	47	1.23E-08	47	1.74E-10
GO:2001251	negative regulation of chromosome organization	47	4.04E-08	47	1.74E-10
GO:0009150	purine ribonucleotide metabolic process	77	1.22E-07	79	1.74E-10
GO:0042773	ATP synthesis coupled electron transport	38	4.53E-11	34	2.06E-10
GO:0042775	mitochondrial ATP synthesis coupled electron transport	38	4.53E-11	34	2.06E-10
GO:0022904	respiratory electron transport chain	40	7.31E-10	38	3.72E-10
GO:0007062	sister chromatid cohesion	51	3.42E-08	51	4.20E-10
GO:0022616	DNA strand elongation	30	1.55E-08	30	5.75E-10
GO:0036503	ERAD pathway	44	6.92E-07	47	6.17E-10

GO:0009408	response to heat	54	4.66E-08	53	9.87E-10
GO:0007051	spindle organization	44	7.85E-08	46	1.24E-09
GO:0006289	nucleotide-excision repair	51	9.64E-08	51	1.27E-09
GO:0008154	actin polymerization or depolymerization	34	4.66E-08	34	1.43E-09
GO:0065002	intracellular protein transmembrane transport	57	7.37E-07	57	1.49E-09
GO:0031577	spindle checkpoint signaling	36	2.61E-07	36	1.68E-09
GO:0051784	negative regulation of nuclear division	40	2.74E-07	40	1.74E-09
GO:1902275	regulation of chromatin organization	50	1.72E-07	52	1.86E-09
GO:0042255	ribosome assembly	60	6.84E-08	61	1.90E-09
GO:0051983	regulation of chromosome segregation	47	3.47E-07	47	1.97E-09
GO:0072521	purine-containing compound metabolic process	90	8.94E-07	92	2.04E-09
GO:0022900	electron transport chain	49	9.24E-12	50	2.39E-09
GO:0043624	cellular protein complex disassembly	46	2.22E-07	45	2.39E-09
GO:0071806	protein transmembrane transport	62	1.21E-06	62	2.42E-09
GO:0048284	organelle fusion	78	2.85E-06	78	2.51E-09
GO:0051170	import into nucleus	58	8.39E-08	56	2.68E-09
GO:0051304	chromosome separation	48	5.10E-07	48	3.01E-09
GO:0070972	protein localization to endoplasmic reticulum	47	2.13E-06	48	3.01E-09
GO:1902531	regulation of intracellular signal transduction	69	3.50E-07	67	3.26E-09
GO:0140013	meiotic nuclear division	93	4.48E-07	91	3.26E-09
GO:0006470	protein dephosphorylation	57	1.29E-04	71	3.26E-09
GO:0008064	regulation of actin polymerization or depolymerization	32	1.83E-07	32	6.24E-09
GO:0008608	attachment of spindle microtubules to kinetochore	32	1.83E-07	32	6.24E-09
GO:0030041	actin filament polymerization	32	1.83E-07	32	6.24E-09
GO:0030832	regulation of actin filament length	32	1.83E-07	32	6.24E-09
GO:0030833	regulation of actin filament polymerization	32	1.83E-07	32	6.24E-09
GO:0006271	DNA strand elongation involved in DNA replication	27	1.40E-07	27	6.25E-09
GO:0110053	regulation of actin filament organization	38	2.74E-07	38	6.84E-09
GO:0009266	response to temperature stimulus	55	1.77E-07	54	8.75E-09
GO:1901293	nucleoside phosphate biosynthetic process	82	4.71E-06	84	9.37E-09

GO:0030433	ubiquitin-dependent ERAD pathway	41	4.75E-07	41	1.05E-08
GO:0031123	RNA 3'-end processing	59	5.81E-07	58	1.05E-08
GO:0046390	ribose phosphate biosynthetic process	60	3.05E-06	62	1.14E-08
GO:0031023	microtubule organizing center organization	31	3.56E-07	31	1.30E-08
GO:0050000	chromosome localization	34	2.31E-07	33	1.45E-08
GO:0006626	protein targeting to mitochondrion	50	2.37E-06	50	1.60E-08
GO:0006164	purine nucleotide biosynthetic process	52	4.17E-06	54	2.07E-08
GO:0006606	protein import into nucleus	52	8.58E-07	51	2.18E-08
GO:0090329	regulation of DNA-dependent DNA replication	38	9.16E-07	38	2.44E-08
GO:0033260	nuclear DNA replication	37	5.06E-07	36	2.76E-08
GO:0044786	cell cycle DNA replication	37	5.06E-07	36	2.76E-08
GO:0051300	spindle pole body organization	30	6.95E-07	30	2.76E-08
GO:0034605	cellular response to heat	42	7.77E-07	41	3.27E-08
GO:0072522	purine-containing compound biosynthetic process	55	8.55E-06	57	4.13E-08
GO:0045132	meiotic chromosome segregation	54	6.92E-07	51	5.24E-08
GO:0000727	double-strand break repair via break-induced replication	24	1.19E-06	24	6.89E-08
GO:0030838	positive regulation of actin filament polymerization	24	1.19E-06	24	6.89E-08
GO:0045010	actin nucleation	24	1.19E-06	24	6.89E-08
GO:0035966	response to topologically incorrect protein	51	2.88E-05	56	7.24E-08
GO:0060341	regulation of cellular localization	55	3.19E-05	60	7.24E-08
GO:0009260	ribonucleotide biosynthetic process	55	1.69E-05	57	8.77E-08
GO:0000028	ribosomal small subunit assembly	26	2.17E-08	26	1.04E-07
GO:0042026	protein refolding	26	1.96E-06	26	1.04E-07
GO:0051028	mRNA transport	70	2.81E-06	68	1.16E-07
GO:0071428	rRNA-containing ribonucleoprotein complex export from nucleus	53	1.16E-06	51	1.19E-07
GO:0034314	Arp2/3 complex-mediated actin nucleation	20	1.96E-06	20	1.50E-07
GO:0070585	protein localization to mitochondrion	53	2.20E-05	53	1.83E-07
GO:0072655	establishment of protein localization to mitochondrion	53	2.20E-05	53	1.83E-07

GO:0006888	endoplasmic reticulum to Golgi vesicle-mediated transport	68	3.64E-05	69	2.37E-07
GO:0034504	protein localization to nucleus	65	1.43E-05	64	2.51E-07
GO:0006099	tricarboxylic acid cycle	27	5.01E-06	27	2.60E-07
GO:0006360	transcription by RNA polymerase I	52	3.49E-05	51	2.60E-07
GO:0033045	regulation of sister chromatid segregation	36	2.36E-05	36	2.93E-07
GO:0072599	establishment of protein localization to endoplasmic reticulum	35	9.29E-05	36	2.93E-07
GO:0006406	mRNA export from nucleus	50	2.37E-06	48	2.96E-07
GO:0071427	mRNA-containing ribonucleoprotein complex export from nucleus	50	2.37E-06	48	2.96E-07
GO:0046785	microtubule polymerization	22	4.94E-06	22	3.36E-07
GO:1902099	regulation of metaphase/anaphase transition of cell cycle	34	2.77E-05	34	3.60E-07
GO:0035967	cellular response to topologically incorrect protein	45	1.19E-04	49	3.67E-07
GO:0006476	protein deacetylation	26	1.96E-06	26	5.23E-07
GO:0016575	histone deacetylation	26	1.96E-06	26	5.23E-07
GO:0009201	ribonucleoside triphosphate biosynthetic process	26	3.77E-05	28	5.23E-07
GO:0033046	negative regulation of sister chromatid segregation	26	3.77E-05	26	5.23E-07
GO:0051985	negative regulation of chromosome segregation	26	3.77E-05	26	5.23E-07
GO:0071173	spindle assembly checkpoint signaling	26	3.77E-05	26	5.23E-07
GO:1902100	negative regulation of metaphase/anaphase transition of cell cycle	26	3.77E-05	26	5.23E-07
GO:1905819	negative regulation of chromosome separation	26	3.77E-05	26	5.23E-07
GO:0009152	purine ribonucleotide biosynthetic process	45	6.05E-05	47	5.23E-07
GO:0000209	protein polyubiquitination	34	2.77E-05	35	5.29E-07
GO:0044784	metaphase/anaphase transition of cell cycle	35	3.91E-05	35	5.29E-07
GO:1905818	regulation of chromosome separation	35	3.91E-05	35	5.29E-07
GO:0045047	protein targeting to ER	34	1.58E-04	35	5.29E-07
GO:0044743	protein transmembrane import into intracellular organelle	41	2.25E-05	41	5.90E-07

GO:0006754	ATP biosynthetic process	19	3.99E-05	21	6.92E-07
GO:0015985	energy coupled proton transport, down electrochemical gradient	19	3.99E-05	21	6.92E-07
GO:0015986	ATP synthesis coupled proton transport	19	3.99E-05	21	6.92E-07
GO:0006890	retrograde vesicle-mediated transport, Golgi to endoplasmic reticulum	29	2.08E-05	29	9.47E-07
GO:0051225	spindle assembly	21	1.02E-05	23	9.62E-07
GO:0031124	mRNA 3'-end processing	35	1.58E-05	34	9.97E-07
GO:0051494	negative regulation of cytoskeleton organization	25	1.93E-05	25	1.05E-06
GO:0045324	late endosome to vacuole transport	47	4.61E-05	47	1.05E-06
GO:0000077	DNA damage checkpoint signaling	31	5.35E-05	32	1.27E-06
GO:0034243	regulation of transcription elongation from RNA polymerase II promoter	37	7.60E-05	38	1.47E-06
GO:0009142	nucleoside triphosphate biosynthetic process	28	1.09E-04	30	1.56E-06
GO:0010499	proteasomal ubiquitin-independent protein catabolic process	17	1.94E-05	17	1.69E-06
GO:0032784	regulation of DNA-templated transcription, elongation	41	1.97E-04	42	1.75E-06
GO:0009199	ribonucleoside triphosphate metabolic process	26	1.20E-04	28	1.85E-06
GO:0006515	protein quality control for misfolded or incompletely synthesized proteins	25	2.09E-04	28	1.85E-06
GO:0030865	cortical cytoskeleton organization	39	1.29E-04	39	1.92E-06
GO:0032968	positive regulation of transcription elongation from RNA polymerase II promoter	35	9.29E-05	36	1.99E-06
GO:0032200	telomere organization	65	4.06E-05	62	2.01E-06
GO:0006301	postreplication repair	26	9.87E-06	26	2.07E-06
GO:0035601	protein deacylation	26	9.87E-06	26	2.07E-06
GO:0043570	maintenance of DNA repeat elements	25	1.93E-05	24	2.14E-06
GO:0031109	microtubule polymerization or depolymerization	24	3.64E-05	24	2.14E-06
GO:0045333	cellular respiration	79	9.59E-06	71	2.18E-06

GO:0032786	positive regulation of DNA-templated transcription, elongation	39	2.54E-04	40	2.43E-06
GO:0032386	regulation of intracellular transport	31	1.39E-04	34	2.64E-06
GO:0006887	exocytosis	39	4.86E-04	41	3.06E-06
GO:0031146	SCF-dependent proteasomal ubiquitin-dependent protein catabolic process	19	3.99E-05	19	3.31E-06
GO:0034502	protein localization to chromosome	31	6.91E-04	35	3.62E-06
GO:0000076	DNA replication checkpoint signaling	16	3.91E-05	16	3.79E-06
GO:0008156	negative regulation of DNA replication	16	3.91E-05	16	3.79E-06
GO:0032527	protein exit from endoplasmic reticulum	16	3.91E-05	16	3.79E-06
GO:0032886	regulation of microtubule-based process	24	3.64E-05	25	4.10E-06
GO:0044396	actin cortical patch organization	21	2.28E-04	21	4.15E-06
GO:2000142	regulation of DNA-templated transcription, initiation	21	2.28E-04	21	4.15E-06
GO:0032535	regulation of cellular component size	47	1.70E-04	47	4.19E-06
GO:0090066	regulation of anatomical structure size	47	1.70E-04	47	4.19E-06
GO:0009060	aerobic respiration	71	1.86E-06	58	4.23E-06
GO:1902904	negative regulation of supramolecular fiber organization	23	6.67E-05	23	4.30E-06
GO:0006353	DNA-templated transcription, termination	34	2.77E-05	33	4.78E-06
GO:0006367	transcription initiation from RNA polymerase II promoter	48	3.45E-04	48	4.79E-06
GO:0048278	vesicle docking	25	5.65E-04	28	5.64E-06
GO:0080134	regulation of response to stress	52	3.49E-05	50	6.18E-06
GO:0098732	macromolecule deacylation	26	3.77E-05	26	6.88E-06
GO:0009896	positive regulation of catabolic process	83	3.50E-07	46	6.89E-06
GO:0080135	regulation of cellular response to stress	48	3.04E-05	46	6.89E-06
GO:0006267	pre-replicative complex assembly involved in nuclear cell cycle DNA replication	18	8.21E-05	18	6.89E-06
GO:1902299	pre-replicative complex assembly involved in cell cycle DNA replication	18	8.21E-05	18	6.89E-06
GO:0036388	pre-replicative complex assembly	18	3.97E-04	18	6.89E-06

GO:0043144	sno(s)RNA processing	35	2.04E-04	35	8.25E-06
GO:0030174	regulation of DNA-dependent DNA replication initiation	20	1.11E-04	20	8.47E-06
GO:0006334	nucleosome assembly	19	2.09E-04	20	8.47E-06
GO:0060260	regulation of transcription initiation from RNA polymerase II promoter	20	4.20E-04	20	8.47E-06
GO:0031331	positive regulation of cellular catabolic process	81	4.58E-07	44	1.01E-05
GO:0010506	regulation of autophagy	44	1.02E-03	44	1.01E-05
GO:0030866	cortical actin cytoskeleton organization	36	5.25E-04	36	1.03E-05
GO:0031929	TOR signaling	35	1.52E-03	36	1.03E-05
GO:0043248	proteasome assembly	27	1.88E-04	27	1.06E-05
GO:0051053	negative regulation of DNA metabolic process	27	1.88E-04	27	1.06E-05
GO:0030162	regulation of proteolysis	32	8.12E-05	30	1.16E-05
GO:0032465	regulation of cytokinesis	25	2.09E-04	25	1.32E-05
GO:0051302	regulation of cell division	25	2.09E-04	25	1.32E-05
GO:2001252	positive regulation of chromosome organization	25	5.65E-04	25	1.32E-05
GO:0019646	aerobic electron transport chain	38	4.53E-11	17	1.47E-05
GO:0034085	establishment of sister chromatid cohesion	17	1.64E-04	17	1.47E-05
GO:0001672	regulation of chromatin assembly or disassembly	16	3.19E-04	17	1.47E-05
GO:0010847	regulation of chromatin assembly	16	3.19E-04	17	1.47E-05
GO:0031445	regulation of heterochromatin assembly	16	3.19E-04	17	1.47E-05
GO:0120261	regulation of heterochromatin organization	16	3.19E-04	17	1.47E-05
GO:2000278	regulation of DNA biosynthetic process	17	7.43E-04	17	1.47E-05
GO:0016074	sno(s)RNA metabolic process	38	4.00E-04	38	1.50E-05
GO:0051347	positive regulation of transferase activity	33	5.42E-04	38	1.50E-05
GO:0070507	regulation of microtubule cytoskeleton organization	22	1.26E-04	23	1.53E-05
GO:0051231	spindle elongation	21	2.28E-04	21	1.72E-05
GO:0006986	response to unfolded protein	37	6.38E-04	39	1.78E-05
GO:0034315	regulation of Arp2/3 complex-mediated actin nucleation	14	1.72E-04	14	1.85E-05
GO:2000104	negative regulation of DNA-dependent DNA replication	14	1.72E-04	14	1.85E-05

GO:0006303	double-strand break repair via nonhomologous end joining	27	4.73E-04	26	1.96E-05
GO:0000183	rDNA heterochromatin assembly	25	5.65E-04	26	1.96E-05
GO:0006900	vesicle budding from membrane	24	3.70E-04	24	2.50E-05
GO:0051648	vesicle localization	24	3.70E-04	24	2.50E-05
GO:0051650	establishment of vesicle localization	24	3.70E-04	24	2.50E-05
GO:1905268	negative regulation of chromatin organization	24	3.70E-04	24	2.50E-05
GO:0031935	regulation of chromatin silencing	26	3.29E-04	27	2.84E-05
GO:0000054	ribosomal subunit export from nucleus	40	1.64E-04	38	3.05E-05
GO:0038202	TORC1 signaling	22	1.14E-03	22	3.05E-05
GO:0009145	purine nucleoside triphosphate biosynthetic process	20	1.30E-03	22	3.05E-05
GO:0009206	purine ribonucleoside triphosphate biosynthetic process	20	1.30E-03	22	3.05E-05
GO:0006123	mitochondrial electron transport, cytochrome c to oxygen	17	1.94E-05	16	3.14E-05
GO:0045862	positive regulation of proteolysis	18	8.21E-05	16	3.14E-05
GO:0034398	telomere tethering at nuclear periphery	17	1.64E-04	16	3.14E-05
GO:0098787	mRNA cleavage involved in mRNA processing	16	3.19E-04	16	3.14E-05
GO:0046822	regulation of nucleocytoplasmic transport	19	2.09E-04	20	3.45E-05
GO:0030150	protein import into mitochondrial matrix	20	4.20E-04	20	3.45E-05
GO:1903432	regulation of TORC1 signaling	20	1.30E-03	20	3.45E-05
GO:0051054	positive regulation of DNA metabolic process	20	3.33E-03	20	3.45E-05
GO:0006090	pyruvate metabolic process	31	6.91E-04	31	3.52E-05
GO:0009141	nucleoside triphosphate metabolic process	29	1.78E-03	31	3.52E-05
GO:0006094	gluconeogenesis	25	5.65E-04	25	3.65E-05
GO:0019319	hexose biosynthetic process	25	5.65E-04	25	3.65E-05
GO:0060968	regulation of gene silencing	27	4.73E-04	28	3.76E-05
GO:0006616	SRP-dependent cotranslational protein targeting to membrane, translocation	13	3.52E-04	13	4.11E-05
GO:0072671	mitochondria-associated ubiquitin-dependent protein catabolic process	13	3.52E-04	13	4.11E-05

GO:0110100	spindle pole body separation	13	3.52E-04	13	4.11E-05
GO:0060627	regulation of vesicle-mediated transport	35	1.52E-03	36	4.28E-05
GO:0070192	chromosome organization involved in meiotic cell cycle	34	3.35E-04	32	4.29E-05
GO:1905037	autophagosome organization	32	1.68E-03	32	4.29E-05
GO:0032511	late endosome to vacuole transport via multivesicular body sorting pathway	37	1.17E-03	37	4.93E-05
GO:0046364	monosaccharide biosynthetic process	26	7.84E-04	26	5.01E-05
GO:0010906	regulation of glucose metabolic process	27	1.09E-03	26	5.01E-05
GO:0033750	ribosome localization	40	3.16E-04	38	5.64E-05
GO:0006369	termination of RNA polymerase II transcription	21	7.26E-04	21	5.70E-05
GO:0010833	telomere maintenance via telomere lengthening	21	7.26E-04	21	5.70E-05
GO:0031113	regulation of microtubule polymerization	15	6.27E-04	15	6.53E-05
GO:0051125	regulation of actin nucleation	15	6.27E-04	15	6.53E-05
GO:0098789	pre-mRNA cleavage required for polyadenylation	15	6.27E-04	15	6.53E-05
GO:0006378	mRNA polyadenylation	19	7.61E-04	19	6.65E-05
GO:0030261	chromosome condensation	19	7.61E-04	19	6.65E-05
GO:0070816	phosphorylation of RNA polymerase II C-terminal domain	19	7.61E-04	19	6.65E-05
GO:0006896	Golgi to vacuole transport	24	9.62E-04	24	6.65E-05
GO:1905269	positive regulation of chromatin organization	19	2.31E-03	19	6.65E-05
GO:0042327	positive regulation of phosphorylation	34	2.37E-03	40	7.02E-05
GO:0000045	autophagosome assembly	31	2.63E-03	31	7.35E-05
GO:0010675	regulation of cellular carbohydrate metabolic process	29	1.78E-03	28	8.54E-05
GO:0031938	regulation of chromatin silencing at telomere	22	1.14E-03	22	8.70E-05
GO:0006898	receptor-mediated endocytosis	21	1.98E-03	22	8.70E-05
GO:0009205	purine ribonucleoside triphosphate metabolic process	20	3.33E-03	22	8.70E-05
GO:0034620	cellular response to unfolded protein	31	2.63E-03	32	8.80E-05
GO:0006122	mitochondrial electron transport, ubiquinol to cytochrome c	15	8.21E-05	12	9.00E-05
GO:0001172	transcription, RNA-templated	12	7.26E-04	12	9.00E-05

GO:0006272	leading strand elongation	12	7.26E-04	12	9.00E-05
GO:0033313	meiotic cell cycle checkpoint signaling	12	7.26E-04	12	9.00E-05
GO:0035493	SNARE complex assembly	12	7.26E-04	12	9.00E-05
GO:0042276	error-prone translesion synthesis	12	7.26E-04	12	9.00E-05
GO:0051988	regulation of attachment of spindle microtubules to kinetochore	12	7.26E-04	12	9.00E-05
GO:0016233	telomere capping	11	1.49E-03	12	9.00E-05
GO:0051445	regulation of meiotic cell cycle	29	1.78E-03	29	1.04E-04
GO:0000393	spliceosomal conformational changes to generate catalytic conformation	20	1.11E-04	20	1.08E-04
GO:0006298	mismatch repair	20	1.30E-03	20	1.08E-04
GO:0007004	telomere maintenance via telomerase	20	1.30E-03	20	1.08E-04
GO:0022406	membrane docking	31	8.19E-03	34	1.17E-04
GO:0140056	organelle localization by membrane tethering	31	8.19E-03	34	1.17E-04
GO:0043244	regulation of protein-containing complex disassembly	23	1.63E-03	23	1.21E-04
GO:0009144	purine nucleoside triphosphate metabolic process	21	4.55E-03	23	1.21E-04
GO:0006379	mRNA cleavage	18	1.40E-03	18	1.29E-04
GO:0006613	cotranslational protein targeting to membrane	18	1.40E-03	18	1.29E-04
GO:0006614	SRP-dependent cotranslational protein targeting to membrane	18	1.40E-03	18	1.29E-04
GO:0007135	meiosis II	17	2.55E-03	18	1.29E-04
GO:0061983	meiosis II cell cycle process	17	2.55E-03	18	1.29E-04
GO:0051261	protein depolymerization	18	3.98E-03	18	1.29E-04
GO:0061408	positive regulation of transcription from RNA polymerase II promoter in response to heat stress	14	1.22E-03	14	1.35E-04
GO:0006407	rRNA export from nucleus	16	1.40E-03	16	1.43E-04
GO:0051029	rRNA transport	16	1.40E-03	16	1.43E-04
GO:0000027	ribosomal large subunit assembly	28	5.32E-03	31	1.47E-04
GO:0006415	translational termination	22	1.14E-03	21	1.60E-04
GO:0006458	'de novo' protein folding	21	1.98E-03	21	1.60E-04

GO:0032006	regulation of TOR signaling	20	7.63E-03	21	1.60E-04
GO:0000245	spliceosomal complex assembly	24	3.70E-04	24	1.60E-04
GO:0033865	nucleoside bisphosphate metabolic process	24	2.26E-03	24	1.60E-04
GO:0033875	ribonucleoside bisphosphate metabolic process	24	2.26E-03	24	1.60E-04
GO:0034032	purine nucleoside bisphosphate metabolic process	24	2.26E-03	24	1.60E-04
GO:2000242	negative regulation of reproductive process	24	2.26E-03	24	1.60E-04
GO:0043628	ncRNA 3'-end processing	32	3.06E-03	32	1.69E-04
GO:0000710	meiotic mismatch repair	11	1.49E-03	11	2.00E-04
GO:0006268	DNA unwinding involved in DNA replication	11	1.49E-03	11	2.00E-04
GO:0034501	protein localization to kinetochore	11	1.49E-03	11	2.00E-04
GO:0035753	maintenance of DNA trinucleotide repeats	11	1.49E-03	11	2.00E-04
GO:0051987	positive regulation of attachment of spindle microtubules to kinetochore	11	1.49E-03	11	2.00E-04
GO:0051031	tRNA transport	22	1.14E-03	19	2.05E-04
GO:0000055	ribosomal large subunit export from nucleus	26	7.84E-04	25	2.05E-04
GO:0006450	regulation of translational fidelity	26	7.84E-04	25	2.05E-04
GO:0006109	regulation of carbohydrate metabolic process	30	4.07E-03	29	2.11E-04
GO:0018105	peptidyl-serine phosphorylation	35	2.74E-03	34	2.15E-04
GO:0032506	cytokinetic process	34	4.12E-03	34	2.15E-04
GO:0006928	movement of cell or subcellular component	22	2.76E-03	22	2.19E-04
GO:0031032	actomyosin structure organization	22	2.76E-03	22	2.19E-04
GO:0044837	actomyosin contractile ring organization	22	2.76E-03	22	2.19E-04
GO:0018209	peptidyl-serine modification	36	3.07E-03	35	2.39E-04
GO:0000731	DNA synthesis involved in DNA repair	17	2.55E-03	17	2.51E-04
GO:0045144	meiotic sister chromatid segregation	16	4.50E-03	17	2.51E-04
GO:0010508	positive regulation of autophagy	26	3.65E-03	26	2.52E-04
GO:0005977	glycogen metabolic process	25	5.85E-03	26	2.52E-04
GO:0031112	positive regulation of microtubule polymerization or depolymerization	13	2.36E-03	13	2.81E-04
GO:0031116	positive regulation of microtubule polymerization	13	2.36E-03	13	2.81E-04

GO:0031452	negative regulation of heterochromatin assembly	13	2.36E-03	13	2.81E-04
GO:0045798	negative regulation of chromatin assembly or disassembly	13	2.36E-03	13	2.81E-04
GO:0045898	regulation of RNA polymerase II transcription preinitiation complex assembly	13	2.36E-03	13	2.81E-04
GO:0051984	positive regulation of chromosome segregation	13	2.36E-03	13	2.81E-04
GO:0120262	negative regulation of heterochromatin organization	13	2.36E-03	13	2.81E-04
GO:0043966	histone H3 acetylation	11	8.62E-03	13	2.81E-04
GO:0051017	actin filament bundle assembly	24	2.26E-03	23	2.83E-04
GO:0061572	actin filament bundle organization	24	2.26E-03	23	2.83E-04
GO:0036003	positive regulation of transcription from RNA polymerase II promoter in response to stress	23	7.58E-03	23	2.83E-04
GO:0031058	positive regulation of histone modification	15	8.05E-03	15	2.83E-04
GO:0006904	vesicle docking involved in exocytosis	11	2.82E-02	15	2.83E-04
GO:0016973	poly(A)+ mRNA export from nucleus	29	8.53E-04	27	3.00E-04
GO:0033674	positive regulation of kinase activity	27	4.50E-03	32	3.12E-04
GO:0072593	reactive oxygen species metabolic process	17	7.43E-04	18	3.84E-04
GO:0000912	assembly of actomyosin apparatus involved in cytokinesis	18	3.98E-03	18	3.84E-04
GO:0000915	actomyosin contractile ring assembly	18	3.98E-03	18	3.84E-04
GO:0000972	transcription-dependent tethering of RNA polymerase II gene DNA at nuclear periphery	18	3.98E-03	18	3.84E-04
GO:0045454	cell redox homeostasis	18	3.98E-03	18	3.84E-04
GO:0006620	posttranslational protein targeting to endoplasmic reticulum membrane	18	2.04E-02	18	3.84E-04
GO:0061077	chaperone-mediated protein folding	22	2.76E-03	21	3.93E-04
GO:0007035	vacuolar acidification	21	4.55E-03	21	3.93E-04
GO:0045005	DNA-dependent DNA replication maintenance of fidelity	21	4.55E-03	21	3.93E-04
GO:0045851	pH reduction	21	4.55E-03	21	3.93E-04

GO:0051452	intracellular pH reduction	21	4.55E-03	21	3.93E-04
GO:0061912	selective autophagy	29	6.25E-03	29	4.03E-04
GO:0001173	DNA-templated transcriptional start site selection	10	3.05E-03	10	4.38E-04
GO:0001174	transcriptional start site selection at RNA polymerase II promoter	10	3.05E-03	10	4.38E-04
GO:0030970	retrograde protein transport, ER to cytosol	10	3.05E-03	10	4.38E-04
GO:0031134	sister chromatid biorientation	10	3.05E-03	10	4.38E-04
GO:0032210	regulation of telomere maintenance via telomerase	10	3.05E-03	10	4.38E-04
GO:0048280	vesicle fusion with Golgi apparatus	10	3.05E-03	10	4.38E-04
GO:1903513	endoplasmic reticulum to cytosol transport	10	3.05E-03	10	4.38E-04
GO:1904356	regulation of telomere maintenance via telomere lengthening	10	3.05E-03	10	4.38E-04
GO:0061187	regulation of ribosomal DNA heterochromatin assembly	9	6.25E-03	10	4.38E-04
GO:0031110	regulation of microtubule polymerization or depolymerization	16	1.40E-03	16	4.76E-04
GO:0034397	telomere localization	17	2.55E-03	16	4.76E-04
GO:0000011	vacuole inheritance	16	4.50E-03	16	4.76E-04
GO:0019985	translesion synthesis	16	4.50E-03	16	4.76E-04
GO:0051084	'de novo' posttranslational protein folding	16	4.50E-03	16	4.76E-04
GO:0051085	chaperone cofactor-dependent protein refolding	16	4.50E-03	16	4.76E-04
GO:0071459	protein localization to chromosome, centromeric region	16	4.50E-03	16	4.76E-04
GO:0140029	exocytic process	14	3.21E-02	16	4.76E-04
GO:0040020	regulation of meiotic nuclear division	22	6.06E-03	22	4.98E-04
GO:0000288	nuclear-transcribed mRNA catabolic process, deadenylation-dependent decay	31	8.19E-03	31	5.00E-04
GO:0051788	response to misfolded protein	16	2.60E-02	19	5.30E-04
GO:0071218	cellular response to misfolded protein	16	2.60E-02	19	5.30E-04
GO:0006111	regulation of gluconeogenesis	14	4.77E-03	14	5.60E-04
GO:0072583	clathrin-dependent endocytosis	14	4.77E-03	14	5.60E-04

GO:2000144	positive regulation of DNA-templated transcription, initiation	14	1.41E-02	14	5.60E-04
GO:0090054	regulation of silent mating-type cassette heterochromatin assembly	12	4.50E-03	12	5.81E-04
GO:0043255	regulation of carbohydrate biosynthetic process	23	7.58E-03	23	6.15E-04
GO:0046112	nucleobase biosynthetic process	17	6.85E-03	17	7.21E-04
GO:0090503	RNA phosphodiester bond hydrolysis, exonucleolytic	23	1.43E-02	25	8.70E-04
GO:0034968	histone lysine methylation	21	9.66E-03	21	8.97E-04
GO:0045143	homologous chromosome segregation	29	3.41E-03	26	9.88E-04
GO:0032507	maintenance of protein location in cell	26	1.28E-02	26	9.88E-04
GO:0051205	protein insertion into membrane	26	1.28E-02	26	9.88E-04
GO:0006112	energy reserve metabolic process	25	1.92E-02	26	9.88E-04
GO:0006739	NADP metabolic process	18	9.50E-03	18	9.90E-04
GO:0006903	vesicle targeting	18	9.50E-03	18	9.90E-04
GO:0000018	regulation of DNA recombination	21	9.66E-03	22	1.08E-03
GO:0000289	nuclear-transcribed mRNA poly(A) tail shortening	13	8.74E-03	13	1.14E-03
GO:0045053	protein retention in Golgi apparatus	13	8.74E-03	13	1.14E-03
GO:0051785	positive regulation of nuclear division	13	8.74E-03	13	1.14E-03
GO:0061587	transfer RNA gene-mediated silencing	13	8.74E-03	13	1.14E-03
GO:0060261	positive regulation of transcription initiation from RNA polymerase II promoter	13	2.49E-02	13	1.14E-03
GO:0006265	DNA topological change	11	8.62E-03	11	1.24E-03
GO:0043687	post-translational protein modification	11	8.62E-03	11	1.24E-03
GO:0045899	positive regulation of RNA polymerase II transcription preinitiation complex assembly	11	8.62E-03	11	1.24E-03
GO:0016571	histone methylation	23	2.59E-02	23	1.26E-03
GO:0030705	cytoskeleton-dependent intracellular transport	19	1.27E-02	19	1.27E-03
GO:0000387	spliceosomal snRNP assembly	16	1.40E-03	16	1.35E-03
GO:0031936	negative regulation of chromatin silencing	16	1.19E-02	16	1.35E-03
GO:0032954	regulation of cytokinetic process	16	1.19E-02	16	1.35E-03

GO:1902115	regulation of organelle assembly	23	1.43E-02	24	1.45E-03
GO:0006607	NLS-bearing protein import into nucleus	15	2.63E-03	14	1.81E-03
GO:0010677	negative regulation of cellular carbohydrate metabolic process	14	1.41E-02	14	1.81E-03
GO:0045835	negative regulation of meiotic nuclear division	14	1.41E-02	14	1.81E-03
GO:0045912	negative regulation of carbohydrate metabolic process	14	1.41E-02	14	1.81E-03
GO:0070786	positive regulation of growth of unicellular organism as a thread of attached cells	14	1.41E-02	14	1.81E-03
GO:0051447	negative regulation of meiotic cell cycle	17	1.60E-02	17	1.81E-03
GO:0060969	negative regulation of gene silencing	17	1.60E-02	17	1.81E-03
GO:0006096	glycolytic process	21	1.89E-02	21	1.86E-03
GO:0006757	ATP generation from ADP	21	1.89E-02	21	1.86E-03
GO:0045860	positive regulation of protein kinase activity	23	2.59E-02	28	2.13E-03
GO:2001020	regulation of response to DNA damage stimulus	21	9.66E-03	22	2.15E-03
GO:0006165	nucleoside diphosphate phosphorylation	22	2.24E-02	22	2.15E-03
GO:0030641	regulation of cellular pH	22	2.24E-02	22	2.15E-03
GO:0051453	regulation of intracellular pH	22	2.24E-02	22	2.15E-03
GO:0032272	negative regulation of protein polymerization	12	1.60E-02	12	2.28E-03
GO:0000147	actin cortical patch assembly	12	4.08E-02	12	2.28E-03
GO:0006284	base-excision repair	15	2.01E-02	15	2.54E-03
GO:0045732	positive regulation of protein catabolic process	13	2.36E-03	10	2.59E-03
GO:0006273	lagging strand elongation	10	1.63E-02	10	2.59E-03
GO:0031990	mRNA export from nucleus in response to heat stress	10	1.63E-02	10	2.59E-03
GO:0032007	negative regulation of TOR signaling	10	1.63E-02	10	2.59E-03
GO:0045721	negative regulation of gluconeogenesis	10	1.63E-02	10	2.59E-03
GO:0061186	negative regulation of silent mating-type cassette heterochromatin assembly	10	1.63E-02	10	2.59E-03
GO:0070682	proteasome regulatory particle assembly	10	1.63E-02	10	2.59E-03
GO:1904262	negative regulation of TORC1 signaling	10	1.63E-02	10	2.59E-03
GO:2000279	negative regulation of DNA biosynthetic process	10	1.63E-02	10	2.59E-03

GO:0006356	regulation of transcription by RNA polymerase I	25	3.08E-02	24	2.66E-03
GO:0031056	regulation of histone modification	19	2.52E-02	19	2.71E-03
GO:0006418	tRNA aminoacylation for protein translation	27	4.50E-03	25	2.91E-03
GO:0009070	serine family amino acid biosynthetic process	19	1.27E-02	20	3.14E-03
GO:0043631	RNA polyadenylation	20	2.87E-02	20	3.14E-03
GO:0045815	positive regulation of gene expression, epigenetic	16	2.60E-02	16	3.24E-03
GO:0031098	stress-activated protein kinase signaling cascade	17	3.13E-02	16	3.24E-03
GO:0043038	amino acid activation	29	6.25E-03	27	3.32E-03
GO:0043039	tRNA aminoacylation	29	6.25E-03	27	3.32E-03
GO:0030046	parallel actin filament bundle assembly	13	2.49E-02	13	3.39E-03
GO:0030474	spindle pole body duplication	13	2.49E-02	13	3.39E-03
GO:0051307	meiotic chromosome separation	13	2.49E-02	13	3.39E-03
GO:0070649	formin-nucleated actin cable assembly	13	2.49E-02	13	3.39E-03
GO:0110009	formin-nucleated actin cable organization	13	2.49E-02	13	3.39E-03
GO:0031126	sno(s)RNA 3'-end processing	21	3.24E-02	21	3.53E-03
GO:0042176	regulation of protein catabolic process	20	7.63E-03	17	3.92E-03
GO:0007018	microtubule-based movement	17	3.13E-02	17	3.92E-03
GO:0051204	protein insertion into mitochondrial membrane	17	3.13E-02	17	3.92E-03
GO:0006885	regulation of pH	22	3.70E-02	22	3.92E-03
GO:0008298	intracellular mRNA localization	22	3.70E-02	22	3.92E-03
GO:0046939	nucleotide phosphorylation	22	3.70E-02	22	3.92E-03
GO:0051056	regulation of small GTPase mediated signal transduction	22	3.70E-02	22	3.92E-03
GO:0032204	regulation of telomere maintenance	13	8.74E-03	11	4.42E-03
GO:0009607	response to biotic stimulus	11	2.82E-02	11	4.42E-03
GO:0043242	negative regulation of protein-containing complex disassembly	11	2.82E-02	11	4.42E-03
GO:0052547	regulation of peptidase activity	11	2.82E-02	11	4.42E-03
GO:0070550	rDNA condensation	11	2.82E-02	11	4.42E-03
GO:2000785	regulation of autophagosome assembly	11	2.82E-02	11	4.42E-03
GO:0006409	tRNA export from nucleus	17	1.60E-02	14	4.52E-03

GO:0071431	tRNA-containing ribonucleoprotein complex export from nucleus	17	1.60E-02	14	4.52E-03
GO:0000973	posttranscriptional tethering of RNA polymerase II gene DNA at nuclear periphery	14	3.21E-02	14	4.52E-03
GO:0051568	histone H3-K4 methylation	14	3.21E-02	14	4.52E-03
GO:0009113	purine nucleobase biosynthetic process	9	3.00E-02	9	5.22E-03
GO:0015936	coenzyme A metabolic process	9	3.00E-02	9	5.22E-03
GO:0030042	actin filament depolymerization	9	3.00E-02	9	5.22E-03
GO:0031070	intronic snoRNA processing	9	3.00E-02	9	5.22E-03
GO:0031509	subtelomeric heterochromatin assembly	9	3.00E-02	9	5.22E-03
GO:0032467	positive regulation of cytokinesis	9	3.00E-02	9	5.22E-03
GO:0034965	intronic box C/D RNA processing	9	3.00E-02	9	5.22E-03
GO:0035065	regulation of histone acetylation	9	3.00E-02	9	5.22E-03
GO:0051310	metaphase plate congression	9	3.00E-02	9	5.22E-03
GO:0051446	positive regulation of meiotic cell cycle	9	3.00E-02	9	5.22E-03
GO:0051781	positive regulation of cell division	9	3.00E-02	9	5.22E-03
GO:0140461	subtelomeric heterochromatin organization	9	3.00E-02	9	5.22E-03
GO:1901983	regulation of protein acetylation	9	3.00E-02	9	5.22E-03
GO:2000756	regulation of peptidyl-lysine acetylation	9	3.00E-02	9	5.22E-03
GO:0006282	regulation of DNA repair	15	2.01E-02	15	5.58E-03
GO:0031333	negative regulation of protein-containing complex assembly	15	4.11E-02	15	5.58E-03
GO:0034067	protein localization to Golgi apparatus	15	4.11E-02	15	5.58E-03
GO:0044380	protein localization to cytoskeleton	15	4.11E-02	15	5.58E-03
GO:0031939	negative regulation of chromatin silencing at telomere	12	4.08E-02	12	6.23E-03
GO:0033866	nucleoside bisphosphate biosynthetic process	12	4.08E-02	12	6.23E-03
GO:0034030	ribonucleoside bisphosphate biosynthetic process	12	4.08E-02	12	6.23E-03
GO:0034033	purine nucleoside bisphosphate biosynthetic process	12	4.08E-02	12	6.23E-03
GO:0061709	reticulophagy	12	4.08E-02	12	6.23E-03
GO:0007030	Golgi organization	27	1.09E-03	17	7.64E-03

GO:0045292	mRNA cis splicing, via spliceosome	14	1.41E-02	13	8.11E-03
GO:0010992	ubiquitin recycling	11	2.82E-02	10	8.44E-03
GO:0051303	establishment of chromosome localization	12	4.08E-02	11	1.14E-02
GO:0051403	stress-activated MAPK cascade	17	3.13E-02	13	1.65E-02
GO:0006376	mRNA splice site selection	11	2.82E-02	11	2.45E-02
