# Supplementary material for: Insights into the identification and evolutionary conservation of key genes in the transcriptional circuits of meiosis initiation and commitment in budding yeast
Source: FEBS Open Bio. 2023 Nov 14;13(12):2290–305. doi: 10.1002/2211-5463.13728 (PMC10699112; doi:10.1002/2211-5463.13728)
Supplement: Supplementary file 11 — File S5. Functional enrichment of the distinct transcriptional cascade of meiosis initiation and commitment. [file FEB4-13-2290-s009.pdf]

Supplementary File 5 -Functional enrichment of the distinct transcriptional cascade of meiosis initiation and commitment						
**Meiosis Initiation**						
**GO**	**Description**	**Count Metascape**	**FDR Metascape**	**Count Cluster profiler**	**p.adjust Cluster Profiler**	**Category**
GO:0000056	ribosomal small subunit export from nucleus	5	3.98E-02	5	3.12E-03	Biological Process
GO:0000460	maturation of 5.8S rRNA	41	1.00E-27	42	3.25E-31	Biological Process
GO:0000463	maturation of LSU-rRNA from tricistronic rRNA transcript (SSU-rRNA5.8S rRNALSU-rRNA)	24	1.00E-15	47	3.68E-34	Biological Process
GO:0000469	cleavage involved in rRNA processing	33	1.00E-19	34	3.54E-23	Biological Process
GO:0000470	maturation of LSU-rRNA	28	1.00E-17	28	1.04E-20	Biological Process
GO:0000478	endonucleolytic cleavage involved in rRNA processing	29	1.00E-20	30	1.39E-23	Biological Process
GO:0000966	RNA 5'-end processing	21	1.00E-12	22	3.16E-16	Biological Process
GO:0000967	rRNA 5'-end processing	21	1.00E-15	22	6.59E-19	Biological Process
GO:0006082	organic acid metabolic process	46	2.51E-05	44	1.44E-06	Biological Process
GO:0006356	regulation of transcription by RNA polymerase I	8	2.51E-02	8	1.65E-03	Biological Process
GO:0006364	rRNA processing	87	1.00E-36	87	9.77E-42	Biological Process
GO:0006400	tRNA modification	14	3.16E-03	15	3.13E-05	Biological Process

GO:0006520	cellular amino acid metabolic process	41	2.00E-10	39	2.99E-11	Biological Process
GO:0006541	glutamine metabolic process	6	3.98E-02	6	4.73E-03	Biological Process
GO:0006730	one-carbon metabolic process	6	1.26E-02	6	1.38E-03	Biological Process
GO:0007007	inner mitochondrial membrane organization	7	2.51E-02	8	6.24E-04	Biological Process
GO:0008652	cellular amino acid biosynthetic process	24	1.58E-06	24	3.21E-08	Biological Process
GO:0009063	cellular amino acid catabolic process	11	1.00E-02	10	2.25E-03	Biological Process
GO:0009064	glutamine family amino acid metabolic process	12	3.98E-03	12	1.71E-04	Biological Process
GO:0009451	RNA modification	22	5.01E-03	23	9.45E-05	Biological Process
GO:0016053	organic acid biosynthetic process	27	6.31E-05	27	1.65E-06	Biological Process
GO:0016072	rRNA metabolic process	88	1.00E-36	88	4.44E-41	Biological Process
GO:0019752	carboxylic acid metabolic process	45	1.26E-05	43	8.73E-07	Biological Process
GO:0030490	maturation of SSU-rRNA	49	1.00E-30	50	2.63E-35	Biological Process
GO:0031120	snRNA pseudouridine synthesis	5	6.31E-03	5	6.65E-04	Biological Process
GO:0032543	mitochondrial translation	39	1.00E-16	39	2.18E-16	Biological Process
GO:0034470	ncRNA processing	101	1.00E-37	102	3.56E-44	Biological Process

GO:0034471	ncRNA 5'-end processing	21	1.00E-12	22	1.99E-16	Biological Process
GO:0040031	snRNA modification	5	1.00E-02	5	1.12E-03	Biological Process
GO:0042254	ribosome biogenesis	106	1.00E-43	110	2.13E-52	Biological Process
GO:0042273	ribosomal large subunit biogenesis	41	1.00E-20	44	3.86E-27	Biological Process
GO:0042274	ribosomal small subunit biogenesis	55	1.00E-33	58	9.73E-40	Biological Process
GO:0042407	cristae formation	6	6.31E-04	7	9.56E-06	Biological Process
GO:0043436	oxoacid metabolic process	45	5.01E-05	43	2.92E-06	Biological Process
GO:0046394	carboxylic acid biosynthetic process	27	6.31E-05	27	1.26E-06	Biological Process
GO:0071166	ribonucleoprotein complex localization	16	3.16E-02	16	1.78E-03	Biological Process
GO:0071426	ribonucleoprotein complex export from nucleus	16	3.16E-02	16	1.54E-03	Biological Process
GO:0071428	rRNA-containing ribonucleoprotein complex export from nucleus	11	3.16E-02	12	6.24E-04	Biological Process
GO:0090305	nucleic acid phosphodiester bond hydrolysis	37	3.16E-06	39	3.74E-09	Biological Process
GO:0090501	RNA phosphodiester bond hydrolysis	37	1.00E-10	38	1.39E-13	Biological Process
GO:0090502	RNA phosphodiester bond hydrolysisendonucleolytic	32	1.00E-11	33	2.96E-14	Biological Process
GO:0140053	mitochondrial gene expression	41	1.00E-15	41	1.33E-15	Biological Process

GO:1901605	alpha-amino acid metabolic process	28	1.58E-06	28	3.02E-08	Biological Process
GO:1901607	alpha-amino acid biosynthetic process	18	5.01E-04	18	3.24E-05	Biological Process
GO:1902626	assembly of large subunit precursor of preribosome	4	2.51E-02	4	7.22E-03	Biological Process

GO	Description	Count Metascape	FDR Metascape	Count Cluster profiler	p.adjust Cluster Profiler	Category
GO:0000049	tRNA binding	12	1.58E-04	10	3.17E-04	Molecular Function
GO:0003724	RNA helicase activity	11	7.94E-04	11	6.76E-05	Molecular Function
GO:0003735	structural constituent of ribosome	38	3.98E-09	37	5.88E-10	Molecular Function
GO:0003743	translation initiation factor activity	8	2.51E-02	7	7.40E-03	Molecular Function
GO:0003746	translation elongation factor activity	5	3.98E-02	5	4.54E-03	Molecular Function
GO:0005198	structural molecule activity	38	3.98E-04	37	3.22E-05	Molecular Function
GO:0008135	translation factor activity RNA binding	13	3.16E-04	11	4.41E-04	Molecular Function
GO:0008173	RNA methyltransferase activity	8	2.51E-02	8	2.29E-03	Molecular Function
GO:0008483	transaminase activity	7	6.31E-03	7	4.41E-04	Molecular Function
GO:0016769	transferase activity transferring nitrogenous groups	7	6.31E-03	7	4.41E-04	Molecular Function

GO:0019843	rRNA binding	23	1.26E-04	22	3.22E-05	Molecular Function
GO:0030515	snoRNA binding	20	1.00E-16	20	3.81E-18	Molecular Function
GO:0034511	U3 snoRNA binding	11	1.58E-08	11	1.62E-09	Molecular Function
GO:0043021	ribonucleoprotein complex binding	14	3.98E-02	13	1.12E-02	Molecular Function
GO:0051082	unfolded protein binding	15	7.94E-04	15	6.76E-05	Molecular Function
GO:0070180	large ribosomal subunit rRNA binding	7	6.31E-04	6	4.41E-04	Molecular Function
GO:0090079	translation regulator activity nucleic acid binding	14	1.00E-04	12	2.00E-04	Molecular Function
GO:0140098	catalytic activity acting on RNA	37	2.00E-05	35	7.86E-06	Molecular Function

**Meiosis Commitment**						
**GO**	**Description**	**Count Metascape**	**FDR Metascape**	**Count Cluster profiler**	**p.adjust Cluster Profiler**	**Category**
GO:0000011	vacuole inheritance	10	3.98E-03	10	4.77E-04	Biological Process
GO:0000022	mitotic spindle elongation	17	1.58E-07	16	7.97E-08	Biological Process
GO:0000070	mitotic sister chromatid segregation	64	1.00E-16	62	6.37E-18	Biological Process
GO:0000073	initial mitotic spindle pole body separation	12	1.26E-07	12	1.11E-08	Biological Process
GO:0000075	cell cycle checkpoint	51	1.00E-13	52	6.54E-17	Biological Process

GO:0000076	DNA replication checkpoint	9	3.98E-03	9	5.34E-04	Biological Process
GO:0000077	DNA damage checkpoint	18	1.58E-04	19	3.07E-06	Biological Process
GO:0000079	regulation of cyclin-dependent protein serine/threonine kinase activity	16	7.94E-04	16	8.58E-05	Biological Process
GO:0000082	G1/S transition of mitotic cell cycle	27	1.00E-04	28	6.42E-07	Biological Process
GO:0000083	regulation of transcription involved in G1/S transition of mitotic cell cycle	11	3.16E-02	11	3.91E-03	Biological Process
GO:0000086	G2/M transition of mitotic cell cycle	26	6.31E-09	27	2.05E-11	Biological Process
GO:0000122	negative regulation of transcription by RNA polymerase II	44	1.26E-03	46	2.06E-05	Biological Process
GO:0000165	MAPK cascade	14	2.00E-02	15	1.53E-03	Biological Process
GO:0000209	protein polyubiquitination	20	5.01E-05	21	7.69E-07	Biological Process
GO:0000226	microtubule cytoskeleton organization	51	1.00E-12	51	3.21E-14	Biological Process
GO:0000271	polysaccharide biosynthetic process	16	3.16E-02	16	3.74E-03	Biological Process
GO:0000278	mitotic cell cycle	153	1.00E-28	153	2.64E-33	Biological Process
GO:0000280	nuclear division	112	1.00E-22	109	6.96E-25	Biological Process
GO:0000281	mitotic cytokinesis	24	2.51E-02	25	9.16E-04	Biological Process
GO:0000302	response to reactive oxygen species	11	3.98E-02	11	1.18E-02	Biological Process

GO:0000722	telomere maintenance via recombination	14	7.94E-03	14	6.16E-04	Biological Process
GO:0000723	telomere maintenance	36	3.98E-07	36	2.66E-09	Biological Process
GO:0000724	double-strand break repair via homologous recombination	45	1.00E-13	45	6.51E-16	Biological Process
GO:0000725	recombinational repair	48	1.00E-13	48	5.13E-16	Biological Process
GO:0000727	double-strand break repair via break-induced replication	15	2.00E-05	15	1.49E-06	Biological Process
GO:0000729	DNA double-strand break processing	8	2.00E-03	8	2.65E-04	Biological Process
GO:0000819	sister chromatid segregation	73	1.00E-18	71	8.34E-20	Biological Process
GO:0000910	cytokinesis	31	6.31E-03	32	1.52E-04	Biological Process
GO:0000912	assembly of actomyosin apparatus involved in cytokinesis	11	3.98E-03	11	4.05E-04	Biological Process
GO:0000915	actomyosin contractile ring assembly	11	3.98E-03	11	4.05E-04	Biological Process
GO:0001672	regulation of chromatin assembly or disassembly	11	2.00E-04	12	4.71E-06	Biological Process
GO:0001932	regulation of protein phosphorylation	38	1.58E-07	44	7.13E-09	Biological Process
GO:0001934	positive regulation of protein phosphorylation	18	1.00E-03	19	3.39E-04	Biological Process
GO:0005976	polysaccharide metabolic process	23	1.00E-02	23	9.51E-04	Biological Process
GO:0005977	glycogen metabolic process	20	6.31E-07	20	5.60E-08	Biological Process

GO:0005978	glycogen biosynthetic process	13	2.51E-04	13	3.97E-05	Biological Process
GO:0006006	glucose metabolic process	24	1.26E-03	24	7.24E-05	Biological Process
GO:0006073	cellular glucan metabolic process	23	6.31E-04	23	4.21E-05	Biological Process
GO:0006091	generation of precursor metabolites and energy	68	1.26E-06	65	2.72E-07	Biological Process
GO:0006109	regulation of carbohydrate metabolic process	20	5.01E-05	19	4.90E-06	Biological Process
GO:0006111	regulation of gluconeogenesis	8	2.00E-02	8	2.91E-03	Biological Process
GO:0006112	energy reserve metabolic process	20	2.00E-06	20	1.85E-07	Biological Process
GO:0006119	oxidative phosphorylation	16	1.58E-02	13	1.20E-02	Biological Process
GO:0006260	DNA replication	63	1.00E-11	63	9.34E-15	Biological Process
GO:0006261	DNA-dependent DNA replication	54	6.31E-10	54	3.40E-12	Biological Process
GO:0006265	DNA topological change	7	1.26E-02	7	1.96E-03	Biological Process
GO:0006268	DNA unwinding involved in DNA replication	6	3.98E-02	6	6.81E-03	Biological Process
GO:0006270	DNA replication initiation	21	6.31E-04	21	3.09E-05	Biological Process
GO:0006271	DNA strand elongation involved in DNA replication	14	6.31E-04	14	5.71E-05	Biological Process
GO:0006272	leading strand elongation	7	1.26E-02	7	1.96E-03	Biological Process

GO:0006273	lagging strand elongation	7	6.31E-03	7	9.95E-04	Biological Process
GO:0006275	regulation of DNA replication	26	1.26E-06	26	6.77E-08	Biological Process
GO:0006281	DNA repair	130	1.00E-26	129	8.06E-31	Biological Process
GO:0006282	regulation of DNA repair	11	7.94E-04	11	1.44E-04	Biological Process
GO:0006289	nucleotide-excision repair	23	1.26E-03	23	7.59E-05	Biological Process
GO:0006301	postreplication repair	12	1.26E-02	12	2.11E-03	Biological Process
GO:0006302	double-strand break repair	69	1.00E-17	68	6.56E-20	Biological Process
GO:0006303	double-strand break repair via nonhomologous end joining	17	1.00E-04	17	2.54E-06	Biological Process
GO:0006310	DNA recombination	74	5.01E-06	73	1.91E-08	Biological Process
GO:0006312	mitotic recombination	21	2.51E-03	18	9.94E-04	Biological Process
GO:0006323	DNA packaging	58	1.00E-12	58	6.45E-15	Biological Process
GO:0006325	chromatin organization	141	1.00E-25	141	6.25E-31	Biological Process
GO:0006333	chromatin assembly or disassembly	61	1.00E-12	61	8.22E-15	Biological Process
GO:0006334	nucleosome assembly	12	3.98E-04	12	7.69E-05	Biological Process
GO:0006338	chromatin remodeling	63	1.00E-10	62	2.98E-13	Biological Process

GO:0006342	chromatin silencing	48	2.51E-09	48	3.99E-11	Biological Process
GO:0006348	chromatin silencing at telomere	33	7.94E-06	33	1.98E-07	Biological Process
GO:0006352	DNA-templated transcription initiation	30	5.01E-04	29	1.87E-05	Biological Process
GO:0006354	DNA-templated transcription elongation	32	6.31E-03	32	2.21E-04	Biological Process
GO:0006357	regulation of transcription by RNA polymerase II	144	1.00E-13	148	2.29E-18	Biological Process
GO:0006367	transcription initiation from RNA polymerase II promoter	25	7.94E-04	25	3.05E-05	Biological Process
GO:0006368	transcription elongation from RNA polymerase II promoter	29	7.94E-03	29	4.65E-04	Biological Process
GO:0006369	termination of RNA polymerase II transcription	10	3.98E-02	10	5.36E-03	Biological Process
GO:0006378	mRNA polyadenylation	11	3.98E-03	11	4.05E-04	Biological Process
GO:0006379	mRNA cleavage	9	3.98E-02	9	5.14E-03	Biological Process
GO:0006468	protein phosphorylation	77	7.94E-10	81	5.95E-13	Biological Process
GO:0006470	protein dephosphorylation	39	2.51E-09	48	1.57E-14	Biological Process
GO:0006473	protein acetylation	37	3.16E-10	36	1.99E-11	Biological Process
GO:0006475	internal protein amino acid acetylation	35	1.00E-10	34	5.37E-12	Biological Process
GO:0006476	protein deacetylation	14	6.31E-04	14	8.95E-05	Biological Process

GO:0006508	proteolysis	121	1.26E-08	114	8.41E-10	Biological Process
GO:0006511	ubiquitin-dependent protein catabolic process	84	1.00E-11	80	5.17E-13	Biological Process
GO:0006515	protein quality control for misfolded or incompletely synthesized proteins	12	2.00E-02	12	5.14E-03	Biological Process
GO:0006623	protein targeting to vacuole	29	3.16E-02	31	5.88E-04	Biological Process
GO:0006754	ATP biosynthetic process	9	2.51E-02	9	7.22E-03	Biological Process
GO:0006885	regulation of pH	19	1.26E-06	19	5.60E-08	Biological Process
GO:0006887	exocytosis	24	2.51E-05	26	6.77E-08	Biological Process
GO:0006888	endoplasmic reticulum to Golgi vesicle-mediated transport	42	3.16E-08	43	1.20E-10	Biological Process
GO:0006890	retrograde vesicle-mediated transport Golgi to endoplasmic reticulum	17	1.00E-04	17	7.35E-06	Biological Process
GO:0006892	post-Golgi vesicle-mediated transport	30	1.26E-04	30	3.34E-06	Biological Process
GO:0006896	Golgi to vacuole transport	15	3.98E-04	16	5.01E-06	Biological Process
GO:0006897	endocytosis	48	1.26E-06	47	1.88E-08	Biological Process
GO:0006900	vesicle budding from membrane	14	1.00E-03	14	8.95E-05	Biological Process
GO:0006906	vesicle fusion	29	5.01E-10	28	1.13E-11	Biological Process
GO:0006914	autophagy	79	2.00E-10	78	3.86E-13	Biological Process

GO:0006970	response to osmotic stress	25	2.00E-03	28	2.48E-04	Biological Process
GO:0006974	cellular response to DNA damage stimulus	146	1.00E-27	146	3.29E-33	Biological Process
GO:0006979	response to oxidative stress	34	2.00E-02	33	2.56E-03	Biological Process
GO:0007004	telomere maintenance via telomerase	15	1.00E-05	15	7.19E-07	Biological Process
GO:0007010	cytoskeleton organization	118	1.00E-23	117	1.61E-27	Biological Process
GO:0007015	actin filament organization	42	1.00E-10	42	5.67E-13	Biological Process
GO:0007017	microtubule-based process	54	1.00E-12	54	1.57E-14	Biological Process
GO:0007030	Golgi organization	17	1.58E-04	10	3.83E-03	Biological Process
GO:0007032	endosome organization	9	3.98E-02	9	5.14E-03	Biological Process
GO:0007033	vacuole organization	41	6.31E-06	40	1.31E-07	Biological Process
GO:0007034	vacuolar transport	60	2.00E-06	59	3.09E-08	Biological Process
GO:0007035	vacuolar acidification	18	2.00E-07	18	1.15E-08	Biological Process
GO:0007051	spindle organization	27	1.26E-07	27	1.46E-08	Biological Process
GO:0007052	mitotic spindle organization	24	1.26E-07	24	2.40E-08	Biological Process
GO:0007059	chromosome segregation	105	1.00E-25	100	3.87E-26	Biological Process

GO:0007062	sister chromatid cohesion	31	2.51E-08	31	6.07E-10	Biological Process
GO:0007064	mitotic sister chromatid cohesion	21	6.31E-06	21	2.84E-07	Biological Process
GO:0007076	mitotic chromosome condensation	6	3.98E-02	6	6.81E-03	Biological Process
GO:0007088	regulation of mitotic nuclear division	21	6.31E-06	21	1.66E-07	Biological Process
GO:0007091	metaphase/anaphase transition of mitotic cell cycle	22	5.01E-06	22	1.44E-07	Biological Process
GO:0007093	mitotic cell cycle checkpoint	39	1.00E-10	40	5.14E-14	Biological Process
GO:0007094	mitotic spindle assembly checkpoint	18	2.00E-06	18	5.26E-08	Biological Process
GO:0007096	regulation of exit from mitosis	17	7.94E-04	17	6.70E-05	Biological Process
GO:0007127	meiosis I	36	1.00E-05	34	1.19E-06	Biological Process
GO:0007129	homologous chromosome pairing at meiosis	13	1.26E-03	11	9.68E-04	Biological Process
GO:0007130	synaptonemal complex assembly	9	1.26E-02	8	4.54E-03	Biological Process
GO:0007131	reciprocal meiotic recombination	22	5.01E-03	22	3.60E-04	Biological Process
GO:0007135	meiosis II	11	1.26E-03	12	4.21E-05	Biological Process
GO:0007154	cell communication	173	1.00E-19	129	7.34E-13	Biological Process
GO:0007165	signal transduction	138	1.00E-15	99	2.42E-10	Biological Process

GO:0007346	regulation of mitotic cell cycle	63	1.00E-12	65	6.54E-17	Biological Process
GO:0007530	sex determination	11	3.16E-02	8	3.99E-02	Biological Process
GO:0007531	mating type determination	11	3.16E-02	8	3.99E-02	Biological Process
GO:0007533	mating type switching	10	1.58E-02	7	2.52E-02	Biological Process
GO:0008064	regulation of actin polymerization or depolymerization	19	6.31E-06	19	3.65E-07	Biological Process
GO:0008154	actin polymerization or depolymerization	21	6.31E-07	21	3.10E-08	Biological Process
GO:0008608	attachment of spindle microtubules to kinetochore	20	1.26E-06	19	3.65E-07	Biological Process
GO:0009057	macromolecule catabolic process	126	1.58E-07	121	2.23E-09	Biological Process
GO:0009165	nucleotide biosynthetic process	33	1.26E-02	34	3.98E-04	Biological Process
GO:0009250	glucan biosynthetic process	16	1.58E-02	16	1.86E-03	Biological Process
GO:0009266	response to temperature stimulus	30	3.16E-06	30	8.49E-08	Biological Process
GO:0009267	cellular response to starvation	28	5.01E-04	22	2.72E-03	Biological Process
GO:0009408	response to heat	29	3.98E-06	29	7.59E-08	Biological Process
GO:0009605	response to external stimulus	48	1.00E-04	43	8.32E-05	Biological Process
GO:0009628	response to abiotic stimulus	55	3.16E-06	61	1.09E-08	Biological Process

GO:0009651	response to salt stress	11	1.58E-02	12	7.53E-04	Biological Process
GO:0009890	negative regulation of biosynthetic process	132	1.00E-10	126	7.07E-15	Biological Process
GO:0009891	positive regulation of biosynthetic process	125	1.58E-10	128	1.05E-14	Biological Process
GO:0009894	regulation of catabolic process	49	1.26E-03	42	2.13E-06	Biological Process
GO:0009896	positive regulation of catabolic process	31	3.98E-02	22	3.60E-04	Biological Process
GO:0009966	regulation of signal transduction	59	1.00E-11	54	1.77E-11	Biological Process
GO:0009967	positive regulation of signal transduction	13	2.51E-02	13	7.81E-03	Biological Process
GO:0009968	negative regulation of signal transduction	19	7.94E-03	20	2.48E-04	Biological Process
GO:0009991	response to extracellular stimulus	46	7.94E-05	41	7.95E-05	Biological Process
GO:0010033	response to organic substance	65	3.16E-03	65	8.95E-05	Biological Process
GO:0010243	response to organonitrogen compound	24	1.58E-02	24	2.08E-03	Biological Process
GO:0010256	endomembrane system organization	39	5.01E-05	29	3.87E-04	Biological Process
GO:0010389	regulation of G2/M transition of mitotic cell cycle	9	2.51E-02	11	2.48E-04	Biological Process
GO:0010458	exit from mitosis	19	1.00E-03	19	7.74E-05	Biological Process
GO:0010498	proteasomal protein catabolic process	59	3.98E-08	57	2.57E-09	Biological Process

GO:0010506	regulation of autophagy	22	3.98E-03	22	1.72E-04	Biological Process
GO:0010557	positive regulation of macromolecule biosynthetic process	122	1.00E-10	125	7.07E-15	Biological Process
GO:0010558	negative regulation of macromolecule biosynthetic process	119	1.26E-08	113	5.81E-12	Biological Process
GO:0010562	positive regulation of phosphorus metabolic process	27	5.01E-05	28	1.13E-05	Biological Process
GO:0010563	negative regulation of phosphorus metabolic process	14	2.51E-02	22	7.78E-05	Biological Process
GO:0010564	regulation of cell cycle process	105	1.00E-20	98	1.71E-24	Biological Process
GO:0010570	regulation of filamentous growth	18	5.01E-04	18	3.51E-05	Biological Process
GO:0010638	positive regulation of organelle organization	55	1.00E-11	55	8.26E-15	Biological Process
GO:0010639	negative regulation of organelle organization	52	1.00E-12	52	1.20E-15	Biological Process
GO:0010646	regulation of cell communication	62	1.00E-11	57	3.68E-12	Biological Process
GO:0010647	positive regulation of cell communication	16	1.00E-02	16	2.36E-03	Biological Process
GO:0010648	negative regulation of cell communication	19	1.00E-02	20	3.24E-04	Biological Process
GO:0010675	regulation of cellular carbohydrate metabolic process	19	5.01E-05	18	6.02E-06	Biological Process
GO:0010677	negative regulation of cellular carbohydrate metabolic process	9	7.94E-03	9	9.25E-04	Biological Process
GO:0010833	telomere maintenance via telomere lengthening	16	3.16E-06	16	1.78E-07	Biological Process

GO:0010847	regulation of chromatin assembly	11	2.00E-04	12	4.71E-06	Biological Process
GO:0010906	regulation of glucose metabolic process	17	1.58E-04	16	2.42E-05	Biological Process
GO:0010921	regulation of phosphatase activity	10	1.58E-02	22	8.52E-08	Biological Process
GO:0010948	negative regulation of cell cycle process	61	1.00E-14	50	6.34E-16	Biological Process
GO:0010965	regulation of mitotic sister chromatid separation	22	5.01E-06	22	1.44E-07	Biological Process
GO:0015980	energy derivation by oxidation of organic compounds	54	5.01E-06	51	2.07E-07	Biological Process
GO:0015985	energy coupled proton transport down electrochemical gradient	9	2.51E-02	9	7.22E-03	Biological Process
GO:0015986	ATP synthesis coupled proton transport	9	2.51E-02	9	7.22E-03	Biological Process
GO:0016050	vesicle organization	50	1.00E-13	50	6.34E-16	Biological Process
GO:0016051	carbohydrate biosynthetic process	29	3.16E-03	29	1.78E-04	Biological Process
GO:0016192	vesicle-mediated transport	152	1.00E-18	150	4.82E-23	Biological Process
GO:0016197	endosomal transport	36	5.01E-04	35	8.79E-05	Biological Process
GO:0016236	macroautophagy	51	3.98E-07	50	7.16E-09	Biological Process
GO:0016237	lysosomal microautophagy	18	2.51E-02	18	1.59E-03	Biological Process
GO:0016310	phosphorylation	101	3.98E-09	117	6.60E-14	Biological Process

GO:0016311	dephosphorylation	45	6.31E-04	54	5.26E-08	Biological Process
GO:0016458	gene silencing	50	1.26E-09	50	4.33E-11	Biological Process
GO:0016567	protein ubiquitination	55	2.00E-08	54	3.69E-10	Biological Process
GO:0016569	covalent chromatin modification	66	1.00E-14	66	1.48E-17	Biological Process
GO:0016570	histone modification	66	1.00E-14	66	1.48E-17	Biological Process
GO:0016573	histone acetylation	34	2.00E-10	33	1.83E-11	Biological Process
GO:0016575	histone deacetylation	14	6.31E-04	14	8.95E-05	Biological Process
GO:0016925	protein sumoylation	10	3.98E-02	10	5.36E-03	Biological Process
GO:0018105	peptidyl-serine phosphorylation	21	2.51E-04	20	4.13E-05	Biological Process
GO:0018193	peptidyl-amino acid modification	98	1.00E-15	96	9.14E-19	Biological Process
GO:0018205	peptidyl-lysine modification	62	1.00E-12	61	4.17E-15	Biological Process
GO:0018209	peptidyl-serine modification	21	5.01E-04	20	7.86E-05	Biological Process
GO:0018393	internal peptidyl-lysine acetylation	35	1.00E-10	34	5.37E-12	Biological Process
GO:0018394	peptidyl-lysine acetylation	35	1.00E-10	34	5.37E-12	Biological Process
GO:0019220	regulation of phosphate metabolic process	62	1.00E-10	80	6.54E-17	Biological Process

GO:0019318	hexose metabolic process	24	3.98E-02	24	2.91E-03	Biological Process
GO:0019725	cellular homeostasis	58	3.16E-03	57	4.62E-05	Biological Process
GO:0019941	modification-dependent protein catabolic process	85	1.00E-11	81	7.51E-13	Biological Process
GO:0022411	cellular component disassembly	41	3.16E-02	40	2.15E-03	Biological Process
GO:0022414	reproductive process	146	1.00E-12	144	4.62E-17	Biological Process
GO:0022616	DNA strand elongation	16	1.26E-04	16	8.74E-06	Biological Process
GO:0022900	electron transport chain	20	2.00E-03	19	3.31E-03	Biological Process
GO:0022904	respiratory electron transport chain	15	2.51E-02	13	1.47E-02	Biological Process
GO:0023051	regulation of signaling	60	1.00E-11	55	6.72E-12	Biological Process
GO:0023052	signaling	138	1.00E-15	100	1.23E-10	Biological Process
GO:0023056	positive regulation of signaling	14	1.26E-02	14	3.53E-03	Biological Process
GO:0023057	negative regulation of signaling	19	7.94E-03	20	2.48E-04	Biological Process
GO:0030004	cellular monovalent inorganic cation homeostasis	21	3.98E-06	21	1.66E-07	Biological Process
GO:0030029	actin filament-based process	62	1.00E-10	62	5.11E-14	Biological Process
GO:0030036	actin cytoskeleton organization	62	1.00E-11	62	2.53E-14	Biological Process

GO:0030041	actin filament polymerization	19	6.31E-06	19	3.65E-07	Biological Process
GO:0030046	parallel actin filament bundle assembly	10	6.31E-04	10	7.86E-05	Biological Process
GO:0030071	regulation of mitotic metaphase/anaphase transition	22	2.00E-06	22	5.22E-08	Biological Process
GO:0030162	regulation of proteolysis	17	1.26E-03	16	1.78E-04	Biological Process
GO:0030163	protein catabolic process	100	1.00E-12	94	4.81E-13	Biological Process
GO:0030174	regulation of DNA-dependent DNA replication initiation	10	1.58E-02	10	1.85E-03	Biological Process
GO:0030261	chromosome condensation	11	3.98E-03	11	4.05E-04	Biological Process
GO:0030433	ubiquitin-dependent ERAD pathway	16	3.98E-02	16	3.74E-03	Biological Process
GO:0030466	silent mating-type cassette heterochromatin assembly	23	1.26E-04	23	5.76E-06	Biological Process
GO:0030474	spindle pole body duplication	10	6.31E-04	10	7.86E-05	Biological Process
GO:0030641	regulation of cellular pH	19	6.31E-07	19	2.98E-08	Biological Process
GO:0030832	regulation of actin filament length	19	6.31E-06	19	3.65E-07	Biological Process
GO:0030833	regulation of actin filament polymerization	19	6.31E-06	19	3.65E-07	Biological Process
GO:0030838	positive regulation of actin filament polymerization	14	1.26E-04	14	1.10E-05	Biological Process
GO:0030865	cortical cytoskeleton organization	22	1.58E-04	22	5.25E-06	Biological Process

GO:0030866	cortical actin cytoskeleton organization	19	2.00E-03	19	1.05E-04	Biological Process
GO:0031023	microtubule organizing center organization	24	1.58E-10	24	4.38E-12	Biological Process
GO:0031032	actomyosin structure organization	13	2.51E-03	13	2.65E-04	Biological Process
GO:0031098	stress-activated protein kinase signaling cascade	11	6.31E-03	11	2.48E-04	Biological Process
GO:0031109	microtubule polymerization or depolymerization	14	3.98E-04	14	3.50E-05	Biological Process
GO:0031110	regulation of microtubule polymerization or depolymerization	10	2.51E-03	10	4.77E-04	Biological Process
GO:0031112	positive regulation of microtubule polymerization or depolymerization	8	7.94E-03	8	9.95E-04	Biological Process
GO:0031113	regulation of microtubule polymerization	9	3.98E-03	9	5.34E-04	Biological Process
GO:0031116	positive regulation of microtubule polymerization	8	7.94E-03	8	9.95E-04	Biological Process
GO:0031123	RNA 3'-end processing	25	6.31E-03	25	2.73E-04	Biological Process
GO:0031124	mRNA 3'-end processing	17	3.16E-03	17	1.88E-04	Biological Process
GO:0031146	SCF-dependent proteasomal ubiquitin-dependent protein catabolic process	10	6.31E-03	8	1.35E-02	Biological Process
GO:0031327	negative regulation of cellular biosynthetic process	132	1.00E-10	126	7.07E-15	Biological Process
GO:0031328	positive regulation of cellular biosynthetic process	125	1.26E-10	128	8.86E-15	Biological Process
GO:0031329	regulation of cellular catabolic process	46	2.51E-03	39	4.69E-06	Biological Process

GO:0031334	positive regulation of protein-containing complex assembly	31	2.51E-08	31	1.01E-09	Biological Process
GO:0031399	regulation of protein modification process	62	3.98E-10	79	1.37E-15	Biological Process
GO:0031400	negative regulation of protein modification process	14	3.16E-02	22	1.02E-04	Biological Process
GO:0031401	positive regulation of protein modification process	32	1.58E-05	33	1.65E-06	Biological Process
GO:0031445	regulation of heterochromatin assembly	11	2.00E-04	12	4.71E-06	Biological Process
GO:0031452	negative regulation of heterochromatin assembly	8	7.94E-03	8	9.95E-04	Biological Process
GO:0031497	chromatin assembly	50	3.16E-10	50	2.61E-12	Biological Process
GO:0031507	heterochromatin assembly	36	3.16E-07	36	5.75E-09	Biological Process
GO:0031570	DNA integrity checkpoint	27	2.00E-07	28	2.78E-09	Biological Process
GO:0031577	spindle checkpoint	22	1.26E-06	22	2.98E-08	Biological Process
GO:0031667	response to nutrient levels	44	7.94E-05	39	7.61E-05	Biological Process
GO:0031668	cellular response to extracellular stimulus	44	6.31E-05	38	1.42E-04	Biological Process
GO:0031669	cellular response to nutrient levels	42	7.94E-05	36	1.55E-04	Biological Process
GO:0031929	TOR signaling	19	2.00E-03	19	1.05E-04	Biological Process
GO:0031935	regulation of chromatin silencing	12	3.16E-02	12	6.69E-03	Biological Process

GO:0031936	negative regulation of chromatin silencing	10	6.31E-03	10	7.80E-04	Biological Process
GO:0032006	regulation of TOR signaling	11	1.58E-02	11	2.04E-03	Biological Process
GO:0032007	negative regulation of TOR signaling	7	6.31E-03	7	9.95E-04	Biological Process
GO:0032069	regulation of nuclease activity	6	3.98E-02	6	3.79E-03	Biological Process
GO:0032200	telomere organization	39	2.00E-07	39	1.17E-09	Biological Process
GO:0032204	regulation of telomere maintenance	8	1.26E-02	8	5.44E-04	Biological Process
GO:0032210	regulation of telomere maintenance via telomerase	8	3.16E-04	8	3.97E-05	Biological Process
GO:0032231	regulation of actin filament bundle assembly	8	7.94E-03	8	9.95E-04	Biological Process
GO:0032270	positive regulation of cellular protein metabolic process	40	3.98E-03	43	3.72E-05	Biological Process
GO:0032271	regulation of protein polymerization	28	1.26E-08	28	2.99E-10	Biological Process
GO:0032273	positive regulation of protein polymerization	22	3.98E-07	22	1.62E-08	Biological Process
GO:0032392	DNA geometric change	19	2.00E-02	24	1.19E-04	Biological Process
GO:0032446	protein modification by small protein conjugation	67	3.98E-10	65	7.28E-12	Biological Process
GO:0032465	regulation of cytokinesis	15	3.98E-04	15	2.97E-05	Biological Process
GO:0032502	developmental process	63	5.01E-03	61	2.81E-04	Biological Process

GO:0032506	cytokinetic process	17	1.58E-02	17	1.35E-03	Biological Process
GO:0032507	maintenance of protein location in cell	14	1.58E-02	14	1.58E-03	Biological Process
GO:0032508	DNA duplex unwinding	19	2.00E-02	20	1.61E-03	Biological Process
GO:0032509	endosome transport via multivesicular body sorting pathway	27	2.00E-06	25	1.05E-06	Biological Process
GO:0032511	late endosome to vacuole transport via multivesicular body sorting pathway	26	5.01E-07	24	3.99E-07	Biological Process
GO:0032527	protein exit from endoplasmic reticulum	8	2.00E-02	9	5.34E-04	Biological Process
GO:0032535	regulation of cellular component size	27	3.16E-05	27	1.22E-06	Biological Process
GO:0032879	regulation of localization	50	1.26E-05	53	3.88E-08	Biological Process
GO:0032880	regulation of protein localization	28	7.94E-04	31	9.55E-06	Biological Process
GO:0032881	regulation of polysaccharide metabolic process	9	1.58E-02	9	2.34E-03	Biological Process
GO:0032886	regulation of microtubule-based process	17	2.00E-06	18	5.26E-08	Biological Process
GO:0032889	regulation of vacuole fusion non-autophagic	7	1.26E-02	9	9.25E-04	Biological Process
GO:0032940	secretion by cell	29	1.58E-05	31	4.69E-08	Biological Process
GO:0032956	regulation of actin cytoskeleton organization	33	1.26E-08	33	4.12E-10	Biological Process
GO:0032970	regulation of actin filament-based process	33	1.26E-08	33	4.12E-10	Biological Process

GO:0033043	regulation of organelle organization	126	1.00E-27	131	3.02E-35	Biological Process
GO:0033044	regulation of chromosome organization	53	1.00E-13	54	1.61E-17	Biological Process
GO:0033045	regulation of sister chromatid segregation	23	2.00E-06	23	4.68E-08	Biological Process
GO:0033046	negative regulation of sister chromatid segregation	18	2.00E-06	18	5.26E-08	Biological Process
GO:0033047	regulation of mitotic sister chromatid segregation	19	6.31E-07	19	1.41E-08	Biological Process
GO:0033048	negative regulation of mitotic sister chromatid segregation	18	2.00E-06	18	5.26E-08	Biological Process
GO:0033260	nuclear DNA replication	21	1.58E-05	21	4.71E-07	Biological Process
GO:0033365	protein localization to organelle	116	1.26E-05	118	5.42E-09	Biological Process
GO:0033674	positive regulation of kinase activity	16	1.58E-03	17	6.18E-04	Biological Process
GO:0033692	cellular polysaccharide biosynthetic process	16	2.51E-02	16	2.97E-03	Biological Process
GO:0034085	establishment of sister chromatid cohesion	13	5.01E-06	13	4.16E-07	Biological Process
GO:0034087	establishment of mitotic sister chromatid cohesion	13	6.31E-07	13	4.66E-08	Biological Process
GO:0034314	Arp2/3 complex-mediated actin nucleation	11	1.26E-03	11	1.44E-04	Biological Process
GO:0034315	regulation of Arp2/3 complex-mediated actin nucleation	9	1.26E-03	9	1.44E-04	Biological Process
GO:0034401	chromatin organization involved in regulation of transcription	61	1.26E-10	61	7.34E-13	Biological Process

GO:0034501	protein localization to kinetochore	6	3.98E-02	5	3.38E-02	Biological Process
GO:0034502	protein localization to chromosome	17	1.58E-03	18	1.42E-04	Biological Process
GO:0034599	cellular response to oxidative stress	29	3.98E-02	28	8.78E-03	Biological Process
GO:0034605	cellular response to heat	21	3.98E-04	21	1.51E-05	Biological Process
GO:0034637	cellular carbohydrate biosynthetic process	19	1.58E-02	19	1.80E-03	Biological Process
GO:0034727	piecemeal microautophagy of the nucleus	14	3.98E-02	14	4.50E-03	Biological Process
GO:0034728	nucleosome organization	31	1.00E-05	31	7.04E-07	Biological Process
GO:0034976	response to endoplasmic reticulum stress	27	7.94E-03	27	5.88E-04	Biological Process
GO:0035303	regulation of dephosphorylation	16	5.01E-04	28	1.67E-09	Biological Process
GO:0035304	regulation of protein dephosphorylation	12	3.98E-04	24	2.39E-09	Biological Process
GO:0035556	intracellular signal transduction	118	1.00E-17	78	5.44E-11	Biological Process
GO:0035601	protein deacylation	14	1.00E-03	14	1.41E-04	Biological Process
GO:0035753	maintenance of DNA trinucleotide repeats	7	6.31E-03	7	9.95E-04	Biological Process
GO:0035825	homologous recombination	26	1.00E-03	23	3.32E-04	Biological Process
GO:0035966	response to topologically incorrect protein	24	2.51E-03	24	5.65E-04	Biological Process

GO:0035967	cellular response to topologically incorrect protein	20	1.58E-02	20	2.91E-03	Biological Process
GO:0036003	positive regulation of transcription from RNA polymerase II promoter in response to stress	19	6.31E-07	18	1.07E-07	Biological Process
GO:0036503	ERAD pathway	19	7.94E-03	19	9.25E-04	Biological Process
GO:0038202	TORC1 signaling	11	2.51E-02	11	2.04E-03	Biological Process
GO:0040008	regulation of growth	22	5.01E-04	22	3.16E-05	Biological Process
GO:0040020	regulation of meiotic nuclear division	13	3.98E-03	13	4.00E-04	Biological Process
GO:0040029	regulation of gene expression epigenetic	48	3.98E-09	48	5.62E-11	Biological Process
GO:0042144	vacuole fusion non-autophagic	18	2.51E-03	17	4.72E-04	Biological Process
GO:0042325	regulation of phosphorylation	41	1.58E-07	48	3.48E-09	Biological Process
GO:0042327	positive regulation of phosphorylation	20	5.01E-04	21	1.80E-04	Biological Process
GO:0042594	response to starvation	31	1.26E-04	26	3.76E-04	Biological Process
GO:0042773	ATP synthesis coupled electron transport	15	7.94E-03	13	3.74E-03	Biological Process
GO:0042775	mitochondrial ATP synthesis coupled electron transport	15	7.94E-03	13	3.74E-03	Biological Process
GO:0042790	nucleolar large rRNA transcription by RNA polymerase I	8	3.98E-02	8	4.54E-03	Biological Process
GO:0043044	ATP-dependent chromatin remodeling	15	2.00E-02	15	1.53E-03	Biological Process

GO:0043085	positive regulation of catalytic activity	41	6.31E-05	66	3.85E-09	Biological Process
GO:0043086	negative regulation of catalytic activity	29	3.98E-05	33	5.10E-07	Biological Process
GO:0043161	proteasome-mediated ubiquitin-dependent protein catabolic process	53	7.94E-07	51	5.58E-08	Biological Process
GO:0043162	ubiquitin-dependent protein catabolic process via the multivesicular body sorting pathway	14	2.51E-02	14	2.72E-03	Biological Process
GO:0043254	regulation of protein-containing complex assembly	47	1.00E-10	47	5.01E-13	Biological Process
GO:0043255	regulation of carbohydrate biosynthetic process	14	2.51E-03	14	2.09E-04	Biological Process
GO:0043467	regulation of generation of precursor metabolites and energy	14	3.16E-03	14	4.45E-04	Biological Process
GO:0043543	protein acylation	40	2.00E-10	39	1.14E-11	Biological Process
GO:0043549	regulation of kinase activity	35	3.98E-07	42	1.30E-08	Biological Process
GO:0043570	maintenance of DNA repeat elements	13	2.51E-03	13	1.72E-04	Biological Process
GO:0043618	regulation of transcription from RNA polymerase II promoter in response to stress	27	1.26E-07	26	1.09E-08	Biological Process
GO:0043620	regulation of DNA-templated transcription in response to stress	27	1.26E-07	26	1.09E-08	Biological Process
GO:0043631	RNA polyadenylation	11	3.16E-02	11	3.91E-03	Biological Process
GO:0043632	modification-dependent macromolecule catabolic process	88	1.58E-10	84	5.81E-12	Biological Process
GO:0043666	regulation of phosphoprotein phosphatase activity	9	1.26E-02	21	9.58E-08	Biological Process

GO:0044042	glucan metabolic process	23	6.31E-04	23	4.21E-05	Biological Process
GO:0044087	regulation of cellular component biogenesis	84	1.00E-17	88	6.06E-22	Biological Process
GO:0044088	regulation of vacuole organization	12	1.26E-02	14	6.16E-04	Biological Process
GO:0044089	positive regulation of cellular component biogenesis	42	1.00E-09	43	7.17E-12	Biological Process
GO:0044092	negative regulation of molecular function	33	6.31E-05	37	5.90E-07	Biological Process
GO:0044093	positive regulation of molecular function	46	1.00E-05	70	1.59E-09	Biological Process
GO:0044257	cellular protein catabolic process	98	1.00E-12	92	4.01E-13	Biological Process
GO:0044264	cellular polysaccharide metabolic process	23	1.26E-03	23	9.71E-05	Biological Process
GO:0044265	cellular macromolecule catabolic process	121	7.94E-08	116	1.46E-09	Biological Process
GO:0044380	protein localization to cytoskeleton	10	6.31E-03	10	7.80E-04	Biological Process
GO:0044770	cell cycle phase transition	102	1.00E-23	87	1.71E-24	Biological Process
GO:0044772	mitotic cell cycle phase transition	85	1.00E-19	87	1.13E-24	Biological Process
GO:0044773	mitotic DNA damage checkpoint	10	3.98E-03	11	1.44E-04	Biological Process
GO:0044774	mitotic DNA integrity checkpoint	18	2.00E-06	19	5.60E-08	Biological Process
GO:0044784	metaphase/anaphase transition of cell cycle	22	5.01E-06	22	1.44E-07	Biological Process

GO:0044786	cell cycle DNA replication	21	1.58E-05	21	4.71E-07	Biological Process
GO:0044837	actomyosin contractile ring organization	13	2.51E-03	13	2.65E-04	Biological Process
GO:0044839	cell cycle G2/M phase transition	26	6.31E-09	27	2.05E-11	Biological Process
GO:0044843	cell cycle G1/S phase transition	27	1.00E-04	28	6.42E-07	Biological Process
GO:0045003	double-strand break repair via synthesis-dependent strand annealing	9	3.98E-03	9	5.34E-04	Biological Process
GO:0045005	DNA-dependent DNA replication maintenance of fidelity	12	6.31E-03	12	7.53E-04	Biological Process
GO:0045010	actin nucleation	14	1.26E-04	14	1.10E-05	Biological Process
GO:0045132	meiotic chromosome segregation	29	1.00E-05	26	6.44E-06	Biological Process
GO:0045143	homologous chromosome segregation	18	3.16E-04	15	4.71E-04	Biological Process
GO:0045144	meiotic sister chromatid segregation	10	3.98E-03	11	1.44E-04	Biological Process
GO:0045165	cell fate commitment	11	3.16E-02	8	3.99E-02	Biological Process
GO:0045324	late endosome to vacuole transport	30	3.16E-07	28	1.44E-07	Biological Process
GO:0045721	negative regulation of gluconeogenesis	7	6.31E-03	7	9.95E-04	Biological Process
GO:0045740	positive regulation of DNA replication	8	3.98E-03	8	5.44E-04	Biological Process
GO:0045786	negative regulation of cell cycle	65	1.00E-15	66	6.93E-19	Biological Process

GO:0045787	positive regulation of cell cycle	39	7.94E-08	41	1.41E-10	Biological Process
GO:0045798	negative regulation of chromatin assembly or disassembly	8	7.94E-03	8	9.95E-04	Biological Process
GO:0045814	negative regulation of gene expression epigenetic	48	2.51E-09	48	3.99E-11	Biological Process
GO:0045815	positive regulation of gene expression epigenetic	10	1.00E-02	10	1.20E-03	Biological Process
GO:0045835	negative regulation of meiotic nuclear division	9	7.94E-03	9	9.25E-04	Biological Process
GO:0045839	negative regulation of mitotic nuclear division	18	2.00E-06	18	5.26E-08	Biological Process
GO:0045841	negative regulation of mitotic metaphase/anaphase transition	18	2.00E-06	18	5.26E-08	Biological Process
GO:0045851	pH reduction	18	2.00E-07	18	1.15E-08	Biological Process
GO:0045859	regulation of protein kinase activity	33	3.98E-07	39	4.59E-08	Biological Process
GO:0045860	positive regulation of protein kinase activity	15	1.26E-03	16	6.31E-04	Biological Process
GO:0045862	positive regulation of proteolysis	9	1.58E-02	10	1.52E-04	Biological Process
GO:0045892	negative regulation of transcription DNA-templated	96	1.00E-11	98	2.62E-15	Biological Process
GO:0045893	positive regulation of transcription DNA-templated	116	1.00E-13	114	5.58E-16	Biological Process
GO:0045898	regulation of RNA polymerase II transcription preinitiation complex assembly	7	3.98E-02	7	5.71E-03	Biological Process
GO:0045912	negative regulation of carbohydrate metabolic process	9	7.94E-03	9	9.25E-04	Biological Process

GO:0045927	positive regulation of growth	12	3.16E-03	12	3.34E-04	Biological Process
GO:0045930	negative regulation of mitotic cell cycle	43	1.00E-11	44	1.86E-14	Biological Process
GO:0045934	negative regulation of nucleobase-containing compound metabolic process	111	1.00E-14	113	2.84E-18	Biological Process
GO:0045935	positive regulation of nucleobase-containing compound metabolic process	137	1.00E-13	131	7.93E-19	Biological Process
GO:0045936	negative regulation of phosphate metabolic process	14	2.00E-02	22	5.78E-05	Biological Process
GO:0045937	positive regulation of phosphate metabolic process	27	5.01E-05	28	1.13E-05	Biological Process
GO:0045944	positive regulation of transcription by RNA polymerase II	103	1.00E-12	105	2.37E-16	Biological Process
GO:0046034	ATP metabolic process	36	2.00E-05	34	1.57E-06	Biological Process
GO:0046578	regulation of Ras protein signal transduction	12	2.00E-02	11	7.06E-03	Biological Process
GO:0046785	microtubule polymerization	12	1.26E-03	12	1.30E-04	Biological Process
GO:0046903	secretion	29	1.58E-05	31	4.69E-08	Biological Process
GO:0048193	Golgi vesicle transport	78	1.00E-11	77	8.20E-15	Biological Process
GO:0048280	vesicle fusion with Golgi apparatus	7	3.16E-03	7	4.72E-04	Biological Process
GO:0048284	organelle fusion	48	1.26E-09	46	5.02E-11	Biological Process
GO:0048285	organelle fission	113	1.00E-21	110	1.55E-23	Biological Process

GO:0048583	regulation of response to stimulus	79	1.00E-15	77	1.53E-16	Biological Process
GO:0048584	positive regulation of response to stimulus	22	3.98E-04	23	4.21E-05	Biological Process
GO:0048585	negative regulation of response to stimulus	22	2.51E-03	24	2.33E-05	Biological Process
GO:0050000	chromosome localization	18	1.58E-04	16	1.26E-04	Biological Process
GO:0050790	regulation of catalytic activity	131	1.00E-16	129	1.13E-19	Biological Process
GO:0050793	regulation of developmental process	17	3.98E-02	17	5.12E-03	Biological Process
GO:0051017	actin filament bundle assembly	16	1.26E-04	16	5.01E-06	Biological Process
GO:0051049	regulation of transport	32	1.26E-03	33	5.16E-05	Biological Process
GO:0051050	positive regulation of transport	13	3.16E-02	14	2.07E-03	Biological Process
GO:0051052	regulation of DNA metabolic process	44	1.58E-09	45	1.92E-12	Biological Process
GO:0051053	negative regulation of DNA metabolic process	16	3.16E-04	16	2.42E-05	Biological Process
GO:0051054	positive regulation of DNA metabolic process	12	3.16E-03	12	1.30E-04	Biological Process
GO:0051056	regulation of small GTPase mediated signal transduction	13	1.00E-02	12	3.86E-03	Biological Process
GO:0051098	regulation of binding	11	1.26E-02	11	9.68E-04	Biological Process
GO:0051101	regulation of DNA binding	9	3.98E-03	9	2.91E-04	Biological Process

GO:0051123	RNA polymerase II preinitiation complex assembly	21	2.51E-04	21	1.51E-05	Biological Process
GO:0051125	regulation of actin nucleation	10	6.31E-04	10	7.86E-05	Biological Process
GO:0051128	regulation of cellular component organization	171	1.00E-30	175	6.58E-39	Biological Process
GO:0051129	negative regulation of cellular component organization	60	1.00E-13	60	2.14E-16	Biological Process
GO:0051130	positive regulation of cellular component organization	68	1.00E-12	68	3.40E-15	Biological Process
GO:0051172	negative regulation of nitrogen compound metabolic process	142	1.00E-11	143	8.71E-17	Biological Process
GO:0051174	regulation of phosphorus metabolic process	62	1.00E-10	80	8.65E-17	Biological Process
GO:0051177	meiotic sister chromatid cohesion	6	3.98E-02	7	1.96E-03	Biological Process
GO:0051225	spindle assembly	15	2.00E-06	16	1.78E-07	Biological Process
GO:0051231	spindle elongation	17	1.58E-07	16	7.97E-08	Biological Process
GO:0051247	positive regulation of protein metabolic process	41	2.51E-03	44	2.29E-05	Biological Process
GO:0051253	negative regulation of RNA metabolic process	97	1.00E-10	99	1.39E-14	Biological Process
GO:0051254	positive regulation of RNA metabolic process	126	1.00E-11	119	1.03E-15	Biological Process
GO:0051258	protein polymerization	35	1.00E-10	34	2.74E-11	Biological Process
GO:0051300	spindle pole body organization	23	6.31E-10	23	1.87E-11	Biological Process

GO:0051301	cell division	111	1.00E-16	110	3.50E-20	Biological Process
GO:0051302	regulation of cell division	15	3.98E-04	15	2.97E-05	Biological Process
GO:0051303	establishment of chromosome localization	8	1.26E-02	6	2.49E-02	Biological Process
GO:0051304	chromosome separation	29	2.51E-07	29	3.85E-09	Biological Process
GO:0051306	mitotic sister chromatid separation	22	5.01E-06	22	1.44E-07	Biological Process
GO:0051321	meiotic cell cycle	107	1.00E-12	106	1.37E-15	Biological Process
GO:0051336	regulation of hydrolase activity	35	1.58E-04	58	6.04E-09	Biological Process
GO:0051338	regulation of transferase activity	44	1.00E-08	51	1.19E-10	Biological Process
GO:0051346	negative regulation of hydrolase activity	7	3.98E-02	11	5.34E-03	Biological Process
GO:0051347	positive regulation of transferase activity	19	3.98E-04	20	1.06E-04	Biological Process
GO:0051348	negative regulation of transferase activity	12	2.00E-02	17	3.55E-04	Biological Process
GO:0051403	stress-activated MAPK cascade	11	6.31E-03	9	1.51E-03	Biological Process
GO:0051445	regulation of meiotic cell cycle	16	3.16E-03	16	2.51E-04	Biological Process
GO:0051447	negative regulation of meiotic cell cycle	10	1.58E-02	10	1.85E-03	Biological Process
GO:0051452	intracellular pH reduction	18	2.00E-07	18	1.15E-08	Biological Process

GO:0051453	regulation of intracellular pH	19	6.31E-07	19	2.98E-08	Biological Process
GO:0051493	regulation of cytoskeleton organization	47	1.00E-12	48	4.08E-15	Biological Process
GO:0051494	negative regulation of cytoskeleton organization	14	6.31E-04	14	5.71E-05	Biological Process
GO:0051495	positive regulation of cytoskeleton organization	31	1.00E-08	31	2.14E-10	Biological Process
GO:0051603	proteolysis involved in cellular protein catabolic process	92	1.00E-11	88	1.94E-13	Biological Process
GO:0051640	organelle localization	53	6.31E-03	53	1.58E-04	Biological Process
GO:0051666	actin cortical patch localization	8	3.16E-02	8	2.91E-03	Biological Process
GO:0051726	regulation of cell cycle	125	1.00E-24	129	3.14E-31	Biological Process
GO:0051783	regulation of nuclear division	33	3.16E-08	33	4.12E-10	Biological Process
GO:0051784	negative regulation of nuclear division	27	6.31E-09	27	8.58E-11	Biological Process
GO:0051788	response to misfolded protein	10	1.00E-02	10	3.83E-03	Biological Process
GO:0051983	regulation of chromosome segregation	30	2.00E-08	30	2.43E-10	Biological Process
GO:0051984	positive regulation of chromosome segregation	8	7.94E-03	8	9.95E-04	Biological Process
GO:0051985	negative regulation of chromosome segregation	18	2.00E-06	18	5.26E-08	Biological Process
GO:0051987	positive regulation of attachment of spindle microtubules to kinetochore	7	6.31E-03	7	9.95E-04	Biological Process

GO:0051988	regulation of attachment of spindle microtubules to kinetochore	7	1.26E-02	7	1.96E-03	Biological Process
GO:0055067	monovalent inorganic cation homeostasis	21	1.00E-05	21	4.71E-07	Biological Process
GO:0060341	regulation of cellular localization	30	3.16E-05	33	1.44E-07	Biological Process
GO:0060627	regulation of vesicle-mediated transport	19	2.00E-03	19	1.92E-04	Biological Process
GO:0060968	regulation of gene silencing	13	2.00E-02	13	3.74E-03	Biological Process
GO:0060969	negative regulation of gene silencing	11	3.98E-03	11	4.05E-04	Biological Process
GO:0061024	membrane organization	67	7.94E-06	64	6.25E-07	Biological Process
GO:0061025	membrane fusion	31	1.00E-08	30	7.21E-10	Biological Process
GO:0061186	negative regulation of silent mating-type cassette heterochromatin assembly	6	3.98E-02	6	6.81E-03	Biological Process
GO:0061408	positive regulation of transcription from RNA polymerase II promoter in response to heat stress	10	3.16E-04	10	3.72E-05	Biological Process
GO:0061572	actin filament bundle organization	16	1.26E-04	16	5.01E-06	Biological Process
GO:0061640	cytoskeleton-dependent cytokinesis	24	2.51E-02	25	1.06E-03	Biological Process
GO:0061804	mitotic spindle formation (spindle phase one)	12	1.26E-07	12	1.11E-08	Biological Process
GO:0061912	selective autophagy	18	5.01E-04	18	3.51E-05	Biological Process
GO:0061919	process utilizing autophagic mechanism	81	3.16E-10	80	5.17E-13	Biological Process

GO:0061982	meiosis I cell cycle process	45	1.00E-07	43	7.16E-09	Biological Process
GO:0061983	meiosis II cell cycle process	11	1.26E-03	12	4.21E-05	Biological Process
GO:0062012	regulation of small molecule metabolic process	26	1.00E-03	25	5.23E-05	Biological Process
GO:0062197	cellular response to chemical stress	40	3.98E-03	42	6.10E-04	Biological Process
GO:0065004	protein-DNA complex assembly	53	6.31E-10	53	3.26E-12	Biological Process
GO:0065009	regulation of molecular function	139	1.00E-17	136	2.57E-20	Biological Process
GO:0070192	chromosome organization involved in meiotic cell cycle	22	7.94E-06	20	3.93E-06	Biological Process
GO:0070193	synaptonemal complex organization	10	1.58E-02	9	5.14E-03	Biological Process
GO:0070507	regulation of microtubule cytoskeleton organization	15	2.00E-05	16	7.69E-07	Biological Process
GO:0070646	protein modification by small protein removal	16	1.26E-02	16	1.09E-03	Biological Process
GO:0070647	protein modification by small protein conjugation or removal	83	1.00E-12	81	7.07E-15	Biological Process
GO:0070649	formin-nucleated actin cable assembly	10	6.31E-04	10	7.86E-05	Biological Process
GO:0070784	regulation of growth of unicellular organism as a thread of attached cells	16	7.94E-04	16	5.78E-05	Biological Process
GO:0070786	positive regulation of growth of unicellular organism as a thread of attached cells	10	1.26E-03	10	1.52E-04	Biological Process
GO:0070828	heterochromatin organization	38	2.00E-07	38	3.22E-09	Biological Process

GO:0070873	regulation of glycogen metabolic process	8	3.16E-02	8	4.54E-03	Biological Process
GO:0070897	transcription preinitiation complex assembly	24	1.00E-04	24	4.38E-06	Biological Process
GO:0071103	DNA conformation change	76	1.00E-13	81	6.37E-18	Biological Process
GO:0071173	spindle assembly checkpoint	18	2.00E-06	18	5.26E-08	Biological Process
GO:0071174	mitotic spindle checkpoint	22	1.26E-06	22	2.98E-08	Biological Process
GO:0071214	cellular response to abiotic stimulus	21	5.01E-03	26	3.05E-04	Biological Process
GO:0071218	cellular response to misfolded protein	10	1.00E-02	10	3.83E-03	Biological Process
GO:0071459	protein localization to chromosome centromeric region	9	1.58E-02	8	9.70E-03	Biological Process
GO:0071478	cellular response to radiation	7	6.31E-03	9	1.44E-04	Biological Process
GO:0071482	cellular response to light stimulus	5	3.98E-02	7	4.72E-04	Biological Process
GO:0071496	cellular response to external stimulus	44	6.31E-05	38	1.42E-04	Biological Process
GO:0071824	protein-DNA complex subunit organization	71	1.00E-11	71	2.97E-14	Biological Process
GO:0071900	regulation of protein serine/threonine kinase activity	21	1.00E-04	27	8.54E-07	Biological Process
GO:0071902	positive regulation of protein serine/threonine kinase activity	8	1.26E-02	10	1.85E-03	Biological Process
GO:0071985	multivesicular body sorting pathway	27	2.00E-06	25	1.05E-06	Biological Process

GO:0071988	protein localization to spindle pole body	7	1.26E-02	7	1.96E-03	Biological Process
GO:0072593	reactive oxygen species metabolic process	9	1.58E-02	10	1.85E-03	Biological Process
GO:0072665	protein localization to vacuole	35	3.98E-02	37	7.35E-04	Biological Process
GO:0072698	protein localization to microtubule cytoskeleton	8	7.94E-03	8	9.95E-04	Biological Process
GO:0072741	protein localization to cell division site	8	7.94E-03	8	9.95E-04	Biological Process
GO:0080134	regulation of response to stress	34	3.16E-08	33	2.59E-09	Biological Process
GO:0080135	regulation of cellular response to stress	31	1.26E-07	30	1.21E-08	Biological Process
GO:0090033	positive regulation of filamentous growth	11	2.51E-03	11	2.48E-04	Biological Process
GO:0090054	regulation of silent mating-type cassette heterochromatin assembly	7	2.00E-02	7	3.50E-03	Biological Process
GO:0090066	regulation of anatomical structure size	27	3.16E-05	27	1.22E-06	Biological Process
GO:0090068	positive regulation of cell cycle process	36	2.00E-07	36	2.66E-09	Biological Process
GO:0090174	organelle membrane fusion	29	6.31E-09	28	1.62E-10	Biological Process
GO:0090307	mitotic spindle assembly	13	2.00E-06	14	2.45E-07	Biological Process
GO:0090329	regulation of DNA-dependent DNA replication	20	1.58E-04	20	9.11E-06	Biological Process
GO:0090337	regulation of formin-nucleated actin cable assembly	7	6.31E-03	7	9.95E-04	Biological Process

GO:0097435	supramolecular fiber organization	57	1.00E-13	57	7.12E-17	Biological Process
GO:0097549	chromatin organization involved in negative regulation of transcription	61	1.00E-10	61	5.36E-13	Biological Process
GO:0097576	vacuole fusion	18	3.16E-03	17	4.72E-04	Biological Process
GO:0098732	macromolecule deacylation	14	1.58E-03	14	2.09E-04	Biological Process
GO:0098787	mRNA cleavage involved in mRNA processing	9	7.94E-03	9	9.25E-04	Biological Process
GO:0098789	pre-mRNA cleavage required for polyadenylation	8	2.00E-02	8	2.91E-03	Biological Process
GO:0098813	nuclear chromosome segregation	89	1.00E-21	84	7.81E-22	Biological Process
GO:0104004	cellular response to environmental stimulus	21	5.01E-03	26	3.05E-04	Biological Process
GO:0110009	formin-nucleated actin cable organization	10	6.31E-04	10	7.86E-05	Biological Process
GO:0110053	regulation of actin filament organization	23	1.26E-06	23	4.68E-08	Biological Process
GO:0110100	spindle pole body separation	12	1.26E-07	12	1.11E-08	Biological Process
GO:0120261	regulation of heterochromatin organization	11	2.00E-04	12	4.71E-06	Biological Process
GO:0120262	negative regulation of heterochromatin organization	8	7.94E-03	8	9.95E-04	Biological Process
GO:0140013	meiotic nuclear division	54	1.58E-09	52	6.16E-11	Biological Process
GO:0140014	mitotic nuclear division	81	1.00E-19	80	1.44E-21	Biological Process

GO:0140352	export from cell	29	3.98E-03	31	2.40E-06	Biological Process
GO:0140527	reciprocal homologous recombination	22	5.01E-03	22	3.60E-04	Biological Process
GO:1900101	regulation of endoplasmic reticulum unfolded protein response	7	3.16E-03	7	4.72E-04	Biological Process
GO:1900428	regulation of filamentous growth of a population of unicellular organisms	17	7.94E-04	17	6.70E-05	Biological Process
GO:1900430	positive regulation of filamentous growth of a population of unicellular organisms	11	2.51E-03	11	2.48E-04	Biological Process
GO:1901293	nucleoside phosphate biosynthetic process	33	1.58E-02	34	5.37E-04	Biological Process
GO:1901565	organonitrogen compound catabolic process	116	1.00E-08	110	4.78E-10	Biological Process
GO:1901698	response to nitrogen compound	25	1.26E-02	25	1.55E-03	Biological Process
GO:1901976	regulation of cell cycle checkpoint	8	7.94E-03	8	9.95E-04	Biological Process
GO:1901987	regulation of cell cycle phase transition	69	1.00E-14	53	4.65E-14	Biological Process
GO:1901988	negative regulation of cell cycle phase transition	54	1.00E-14	36	4.42E-13	Biological Process
GO:1901990	regulation of mitotic cell cycle phase transition	51	1.00E-10	53	1.99E-14	Biological Process
GO:1901991	negative regulation of mitotic cell cycle phase transition	35	3.16E-10	36	4.42E-13	Biological Process
GO:1902099	regulation of metaphase/anaphase transition of cell cycle	22	2.00E-06	22	5.22E-08	Biological Process
GO:1902100	negative regulation of metaphase/anaphase transition of cell cycle	18	2.00E-06	18	5.26E-08	Biological Process

GO:1902115	regulation of organelle assembly	12	3.16E-02	14	6.16E-04	Biological Process
GO:1902275	regulation of chromatin organization	27	7.94E-06	27	8.54E-07	Biological Process
GO:1902407	assembly of actomyosin apparatus involved in mitotic cytokinesis	11	1.26E-03	11	1.44E-04	Biological Process
GO:1902410	mitotic cytokinetic process	14	6.31E-04	14	5.71E-05	Biological Process
GO:1902531	regulation of intracellular signal transduction	40	5.01E-08	35	1.46E-07	Biological Process
GO:1902532	negative regulation of intracellular signal transduction	15	2.51E-02	16	6.31E-04	Biological Process
GO:1902533	positive regulation of intracellular signal transduction	12	2.51E-02	12	8.55E-03	Biological Process
GO:1902600	proton transmembrane transport	31	3.98E-03	29	5.44E-04	Biological Process
GO:1902679	negative regulation of RNA biosynthetic process	96	1.00E-11	98	6.70E-15	Biological Process
GO:1902680	positive regulation of RNA biosynthetic process	116	1.00E-13	117	1.25E-16	Biological Process
GO:1902749	regulation of cell cycle G2/M phase transition	9	2.51E-02	11	2.48E-04	Biological Process
GO:1902850	microtubule cytoskeleton organization involved in mitosis	28	1.58E-07	28	1.91E-08	Biological Process
GO:1902903	regulation of supramolecular fiber organization	33	1.00E-09	33	1.83E-11	Biological Process
GO:1902904	negative regulation of supramolecular fiber organization	13	1.26E-03	13	1.09E-04	Biological Process
GO:1902905	positive regulation of supramolecular fiber organization	27	6.31E-09	27	1.66E-10	Biological Process

GO:1902969	mitotic DNA replication	8	2.00E-02	8	1.80E-03	Biological Process
GO:1903046	meiotic cell cycle process	87	5.01E-09	86	1.68E-11	Biological Process
GO:1903047	mitotic cell cycle process	147	1.00E-27	148	3.29E-33	Biological Process
GO:1903432	regulation of TORC1 signaling	11	7.94E-03	11	6.30E-04	Biological Process
GO:1903475	mitotic actomyosin contractile ring assembly	11	1.26E-03	11	1.44E-04	Biological Process
GO:1903507	negative regulation of nucleic acid-templated transcription	96	1.00E-11	98	6.70E-15	Biological Process
GO:1903508	positive regulation of nucleic acid-templated transcription	116	1.00E-13	117	1.25E-16	Biological Process
GO:1903827	regulation of cellular protein localization	23	1.58E-04	27	8.54E-07	Biological Process
GO:1903828	negative regulation of cellular protein localization	7	2.00E-02	8	9.95E-04	Biological Process
GO:1904029	regulation of cyclin-dependent protein kinase activity	17	2.51E-04	17	3.04E-05	Biological Process
GO:1904262	negative regulation of TORC1 signaling	7	6.31E-03	7	9.95E-04	Biological Process
GO:1904356	regulation of telomere maintenance via telomere lengthening	8	3.16E-04	8	3.97E-05	Biological Process
GO:1904669	ATP export	8	3.16E-02	8	4.54E-03	Biological Process
GO:1905047	mitotic spindle pole body organization	12	1.26E-07	12	1.11E-08	Biological Process
GO:1905268	negative regulation of chromatin organization	15	2.00E-04	15	1.76E-05	Biological Process

GO:1905269	positive regulation of chromatin organization	10	2.00E-02	9	7.22E-03	Biological Process
GO:1905508	protein localization to microtubule organizing center	7	1.26E-02	7	1.96E-03	Biological Process
GO:1905818	regulation of chromosome separation	22	5.01E-06	22	1.44E-07	Biological Process
GO:1905819	negative regulation of chromosome separation	18	2.00E-06	18	5.26E-08	Biological Process
GO:1905897	regulation of response to endoplasmic reticulum stress	7	6.31E-03	7	9.95E-04	Biological Process
GO:2000105	positive regulation of DNA-dependent DNA replication	6	2.51E-02	6	3.79E-03	Biological Process
GO:2000113	negative regulation of cellular macromolecule biosynthetic process	119	1.26E-08	113	2.98E-12	Biological Process
GO:2000142	regulation of DNA-templated transcription initiation	10	3.16E-02	10	2.72E-03	Biological Process
GO:2000217	regulation of invasive growth in response to glucose limitation	7	1.26E-02	7	1.96E-03	Biological Process
GO:2000241	regulation of reproductive process	27	2.51E-02	27	1.16E-03	Biological Process
GO:2000242	negative regulation of reproductive process	13	7.94E-03	13	8.27E-04	Biological Process
GO:2000278	regulation of DNA biosynthetic process	11	7.94E-04	11	4.21E-05	Biological Process
GO:2000279	negative regulation of DNA biosynthetic process	6	3.98E-02	6	6.81E-03	Biological Process
GO:2000816	negative regulation of mitotic sister chromatid separation	18	2.00E-06	18	5.26E-08	Biological Process
GO:2001020	regulation of response to DNA damage stimulus	15	1.26E-04	16	8.74E-06	Biological Process

GO:2001251	negative regulation of chromosome organization	31	1.26E-09	31	1.18E-11	Biological Process
GO:2001252	positive regulation of chromosome organization	13	7.94E-03	12	2.11E-03	Biological Process

GO	Description	Count Metascape	FDR Metascape	Count Cluster profiler	p.adjust Cluster Profiler	Category
GO:0000149	SNARE binding	20	5.01E-05	20	3.43E-06	Molecular Function
GO:0000976	transcription regulatory region sequence-specific DNA binding	53	1.58E-05	51	2.32E-07	Molecular Function
GO:0000977	RNA polymerase II transcription regulatory region sequence-specific DNA binding	36	3.16E-03	36	2.73E-05	Molecular Function
GO:0000978	RNA polymerase II cis-regulatory region sequence-specific DNA binding	34	7.94E-04	31	2.73E-05	Molecular Function
GO:0000981	DNA-binding transcription factor activity RNA polymerase II-specific	40	3.16E-02	37	8.27E-03	Molecular Function
GO:0000987	cis-regulatory region sequence-specific DNA binding	38	6.31E-04	35	2.76E-05	Molecular Function
GO:0000993	RNA polymerase II complex binding	11	1.26E-02	11	2.16E-03	Molecular Function
GO:0001067	regulatory region nucleic acid binding	54	1.00E-05	52	1.46E-07	Molecular Function
GO:0001098	basal transcription machinery binding	13	1.58E-02	13	2.29E-03	Molecular Function
GO:0001099	basal RNA polymerase II transcription machinery binding	13	1.58E-02	13	2.29E-03	Molecular Function
GO:0001216	DNA-binding transcription activator activity	23	1.58E-04	21	1.65E-04	Molecular Function

GO:0001228	DNA-binding transcription activator activity RNA polymerase II-specific	23	1.58E-04	21	1.65E-04	Molecular Function
GO:0003682	chromatin binding	63	1.00E-14	64	1.71E-17	Molecular Function
GO:0003684	damaged DNA binding	10	3.98E-02	10	7.74E-03	Molecular Function
GO:0003688	DNA replication origin binding	13	2.51E-02	13	3.01E-03	Molecular Function
GO:0003690	double-stranded DNA binding	94	1.00E-12	92	7.15E-16	Molecular Function
GO:0003697	single-stranded DNA binding	29	3.98E-06	27	2.14E-06	Molecular Function
GO:0003700	DNA-binding transcription factor activity	45	1.58E-02	45	7.76E-04	Molecular Function
GO:0003712	transcription coregulator activity	25	3.98E-02	24	8.82E-03	Molecular Function
GO:0003779	actin binding	23	2.51E-04	23	1.55E-05	Molecular Function
GO:0004402	histone acetyltransferase activity	16	6.31E-06	17	5.65E-07	Molecular Function
GO:0004520	endodeoxyribonuclease activity	11	1.26E-03	13	1.10E-04	Molecular Function
GO:0004536	deoxyribonuclease activity	14	3.16E-03	16	1.42E-04	Molecular Function
GO:0004672	protein kinase activity	43	1.00E-04	45	1.34E-06	Molecular Function
GO:0004674	protein serine/threonine kinase activity	40	2.00E-04	42	3.65E-06	Molecular Function
GO:0004721	phosphoprotein phosphatase activity	23	2.51E-05	23	5.31E-06	Molecular Function

GO:0004722	protein serine/threonine phosphatase activity	15	1.26E-04	13	1.70E-04	Molecular Function
GO:0004842	ubiquitin-protein transferase activity	41	1.26E-08	41	6.27E-10	Molecular Function
GO:0004857	enzyme inhibitor activity	22	3.98E-04	21	2.06E-04	Molecular Function
GO:0005484	SNAP receptor activity	20	1.00E-10	20	8.30E-12	Molecular Function
GO:0005543	phospholipid binding	34	2.00E-03	33	2.04E-04	Molecular Function
GO:0008017	microtubule binding	18	6.31E-04	17	2.79E-04	Molecular Function
GO:0008047	enzyme activator activity	57	3.98E-06	50	8.84E-08	Molecular Function
GO:0008080	N-acetyltransferase activity	18	2.51E-03	19	1.65E-04	Molecular Function
GO:0008092	cytoskeletal protein binding	42	2.51E-06	42	2.32E-07	Molecular Function
GO:0008094	DNA-dependent ATPase activity	29	7.94E-04	24	2.20E-05	Molecular Function
GO:0008134	transcription factor binding	29	6.31E-04	29	1.80E-04	Molecular Function
GO:0008289	lipid binding	42	1.00E-03	39	2.13E-04	Molecular Function
GO:0015078	proton transmembrane transporter activity	29	2.00E-02	27	3.74E-03	Molecular Function
GO:0015252	proton channel activity	9	1.26E-02	9	2.25E-03	Molecular Function
GO:0015631	tubulin binding	18	1.26E-02	17	4.76E-03	Molecular Function

GO:0016251	RNA polymerase II general transcription initiation factor activity	13	6.31E-05	12	1.96E-05	Molecular Function
GO:0016301	kinase activity	63	1.26E-04	64	1.35E-06	Molecular Function
GO:0016407	acetyltransferase activity	19	3.98E-02	20	2.79E-03	Molecular Function
GO:0016410	N-acyltransferase activity	18	1.26E-02	19	8.32E-04	Molecular Function
GO:0016538	cyclin-dependent protein serine/threonine kinase regulator activity	13	1.00E-02	13	1.19E-03	Molecular Function
GO:0016773	phosphotransferase activity alcohol group as acceptor	55	5.01E-04	56	6.27E-06	Molecular Function
GO:0016888	endodeoxyribonuclease activity producing 5'-phosphomonoesters	6	5.01E-03	6	9.58E-03	Molecular Function
GO:0017025	TBP-class protein binding	13	5.01E-06	13	1.17E-06	Molecular Function
GO:0019207	kinase regulator activity	25	3.16E-04	25	2.76E-05	Molecular Function
GO:0019208	phosphatase regulator activity	20	1.00E-05	19	3.92E-06	Molecular Function
GO:0019209	kinase activator activity	10	1.00E-02	10	1.14E-03	Molecular Function
GO:0019787	ubiquitin-like protein transferase activity	48	1.26E-09	47	1.92E-11	Molecular Function
GO:0019789	SUMO transferase activity	7	3.98E-02	7	4.90E-03	Molecular Function
GO:0019829	ATPase-coupled cation transmembrane transporter activity	14	1.00E-03	14	3.12E-05	Molecular Function
GO:0019887	protein kinase regulator activity	24	5.01E-04	24	2.76E-05	Molecular Function

GO:0019888	protein phosphatase regulator activity	20	6.31E-06	19	2.59E-06	Molecular Function
GO:0019899	enzyme binding	58	3.98E-07	54	3.58E-10	Molecular Function
GO:0019901	protein kinase binding	13	3.98E-02	13	6.95E-03	Molecular Function
GO:0019902	phosphatase binding	9	3.98E-03	9	7.93E-04	Molecular Function
GO:0019903	protein phosphatase binding	9	3.98E-03	9	7.93E-04	Molecular Function
GO:0019905	syntaxin binding	6	3.98E-02	6	9.58E-03	Molecular Function
GO:0030234	enzyme regulator activity	100	1.00E-11	100	1.10E-12	Molecular Function
GO:0030276	clathrin binding	11	2.51E-03	10	1.14E-03	Molecular Function
GO:0030295	protein kinase activator activity	10	1.00E-02	10	1.14E-03	Molecular Function
GO:0030674	protein-macromolecule adaptor activity	58	1.00E-10	56	1.10E-12	Molecular Function
GO:0031490	chromatin DNA binding	8	7.94E-04	8	1.73E-04	Molecular Function
GO:0031491	nucleosome binding	12	6.31E-03	13	3.98E-04	Molecular Function
GO:0032182	ubiquitin-like protein binding	22	7.94E-05	22	3.43E-06	Molecular Function
GO:0034212	peptide N-acetyltransferase activity	18	3.98E-05	19	2.59E-06	Molecular Function
GO:0035091	phosphatidylinositol binding	24	2.00E-02	24	1.50E-03	Molecular Function

GO:0035251	UDP-glucosyltransferase activity	7	2.00E-02	7	8.22E-03	Molecular Function
GO:0042162	telomeric DNA binding	16	3.98E-05	14	5.39E-05	Molecular Function
GO:0042393	histone binding	29	6.31E-09	30	2.69E-10	Molecular Function
GO:0042625	ATPase-coupled ion transmembrane transporter activity	14	1.26E-07	15	4.58E-05	Molecular Function
GO:0043130	ubiquitin binding	20	1.00E-04	20	5.11E-06	Molecular Function
GO:0043565	sequence-specific DNA binding	91	2.51E-09	89	1.92E-11	Molecular Function
GO:0044769	ATPase activity coupled to transmembrane movement of ions rotational mechanism	13	6.31E-07	13	4.36E-07	Molecular Function
GO:0044877	protein-containing complex binding	66	3.98E-04	66	1.43E-05	Molecular Function
GO:0046527	glucosyltransferase activity	8	2.00E-02	8	6.57E-03	Molecular Function
GO:0046933	proton-transporting ATP synthase activity rotational mechanism	9	1.26E-02	9	2.25E-03	Molecular Function
GO:0046961	proton-transporting ATPase activity rotational mechanism	13	6.31E-07	13	4.36E-07	Molecular Function
GO:0046982	protein heterodimerization activity	15	1.26E-04	15	2.76E-05	Molecular Function
GO:0046983	protein dimerization activity	20	2.51E-03	20	3.72E-04	Molecular Function
GO:0051010	microtubule plus-end binding	10	1.26E-03	9	7.93E-04	Molecular Function
GO:0051015	actin filament binding	13	1.58E-02	13	2.29E-03	Molecular Function

GO:0061630	ubiquitin protein ligase activity	19	1.00E-02	17	4.76E-03	Molecular Function
GO:0061631	ubiquitin conjugating enzyme activity	8	7.94E-04	10	2.70E-05	Molecular Function
GO:0061650	ubiquitin-like protein conjugating enzyme activity	9	5.01E-04	11	6.25E-06	Molecular Function
GO:0061659	ubiquitin-like protein ligase activity	20	7.94E-03	18	2.79E-03	Molecular Function
GO:0061733	peptide-lysine-N-acetyltransferase activity	16	6.31E-06	17	5.65E-07	Molecular Function
GO:0070063	RNA polymerase binding	12	2.51E-02	11	9.77E-03	Molecular Function
GO:0098772	molecular function regulator	107	1.26E-10	123	2.42E-14	Molecular Function
GO:0140030	modification-dependent protein binding	12	3.16E-03	13	2.58E-04	Molecular Function
GO:0140110	transcription regulator activity	70	2.00E-04	69	5.28E-06	Molecular Function
GO:0140223	general transcription initiation factor activity	20	3.16E-07	18	3.50E-07	Molecular Function
GO:0140296	general transcription initiation factor binding	18	1.26E-05	18	1.84E-06	Molecular Function
GO:1990837	sequence-specific double-stranded DNA binding	68	5.01E-07	66	1.90E-09	Molecular Function
GO:0000217	DNA secondary structure binding	6	1.58E-03	9	2.43E-02	Molecular Function
GO:0000384	first spliceosomal transesterification activity	169	1.00E-12	6	2.42E-02	Molecular Function
GO:0000400	four-way junction DNA binding	4	7.94E-03	6	1.58E-02	Molecular Function

GO:0001102	RNA polymerase II activating transcription factor binding	3	3.98E-02	9	1.89E-02	Molecular Function
GO:0001217	DNA-binding transcription repressor activity	6	1.26E-02	10	2.67E-03	Molecular Function
GO:0001227	DNA-binding transcription repressor activity RNA polymerase II-specific	13	5.01E-06	10	2.67E-03	Molecular Function
GO:0003678	DNA helicase activity	3	3.98E-02	16	3.06E-02	Molecular Function
GO:0003714	transcription corepressor activity	3	3.98E-02	9	3.76E-02	Molecular Function
GO:0004407	histone deacetylase activity	6	5.01E-03	8	4.15E-03	Molecular Function
GO:0004527	exonuclease activity	4	7.94E-03	15	1.33E-02	Molecular Function
GO:0004712	protein serine/threonine/tyrosine kinase activity	4	7.94E-03	5	4.28E-02	Molecular Function
GO:0004864	protein phosphatase inhibitor activity	3	3.98E-02	5	4.28E-02	Molecular Function
GO:0005096	GTPase activator activity	5	2.00E-02	15	3.48E-02	Molecular Function
GO:0005261	cation channel activity	7	1.26E-03	9	3.05E-02	Molecular Function
GO:0005509	calcium ion binding	7	6.31E-03	9	4.54E-02	Molecular Function
GO:0008234	cysteine-type peptidase activity	5	2.00E-02	10	3.40E-02	Molecular Function
GO:0008408	3'-5' exonuclease activity	9	7.94E-03	11	2.09E-02	Molecular Function
GO:0008553	proton-exporting ATPase activity phosphorylative mechanism	4	3.16E-02	5	3.00E-02	Molecular Function

GO:0009055	electron transfer activity	4	3.16E-02	16	1.70E-02	Molecular Function
GO:0015616	DNA translocase activity	13	3.98E-04	10	2.88E-02	Molecular Function
GO:0016462	pyrophosphatase activity	13	3.98E-04	91	7.16E-04	Molecular Function
GO:0016747	transferase activity transferring acyl groups other than amino-acyl groups	29	3.16E-03	27	2.46E-02	Molecular Function
GO:0016772	transferase activity transferring phosphorus-containing groups	29	3.16E-03	75	1.03E-02	Molecular Function
GO:0016791	phosphatase activity	5	2.00E-02	26	3.39E-02	Molecular Function
GO:0016796	exonuclease activity active with either ribo- or deoxyribonucleic acids and producing 5'-phosphomonoesters	192	1.00E-17	11	3.06E-02	Molecular Function
GO:0016817	hydrolase activity acting on acid anhydrides	4	7.94E-03	91	7.16E-04	Molecular Function

**GO**	**Description**	**Count Metascape**	**FDR Metascape**	**Count Cluster profiler**	**p.adjust Cluster Profiler**	**Category**
sce04113	Meiosis - yeast	53	7.94E-10	10	3.62E-03	KEGG Pathway
