# Supplementary material for: Insights into the identification and evolutionary conservation of key genes in the transcriptional circuits of meiosis initiation and commitment in budding yeast
Source: FEBS Open Bio. 2023 Nov 14;13(12):2290–305. doi: 10.1002/2211-5463.13728 (PMC10699112; doi:10.1002/2211-5463.13728)
Supplement: Supplementary file 12 — File S6. dN/dS values of the genes from distinct transcriptional cascade of meiosis initiation and commitment of S. cerevisiae with their orthologs in 12 species. [file FEB4-13-2290-s012.pdf]

Supplementary File 6- ***dN/dS values of the genes from distinct transcriptional cascade of meiosis initiation and commitment of S.cerevisiae with their orthologs in 12 species.***

Ashbya gossypii										
**Meiosis Initiation**						**Meiosis Commitment**				
**S.cerevisae gene name**	**Ashbya gossypii gene name**	**% of S.cerevisae gene identical to target A. gossypii gene**	**dN/dS**	**MEGA P value**		**S.cerevisae gene name**	**Ashbya gossypii gene name**	**% of S.cerevisae gene identical to target A. gossypii gene**	**dN/dS**	**MEGA P value**
ALA1	AGOS_ADR363C	75.7322	0.0073	0		ADA2	AGOS_AER318C	77.4713	0.0211	0
ARG1	AGOS_AER230C	81.6425	0.0016	0		ALD4	AGOS_ADR417W	65.8777	0.0197	7.7E-07
ARG3	AGOS_AFR098W	65.043	0.0031	0		ALG6	AGOS_ACR004W	61.6341	0.0195	0
ARO1	AGOS_AGR066W	67.026	0.0037	0		APL2	AGOS_AFR200W	61.5274	0.0195	0
ARO4	AGOS_AFL047W	82.967	0.0013	0		ARC18	AGOS_AFR584C	71.3513	0.0187	0
BMS1	AGOS_AFL113C	71.6007	0.0141	0		ASF1	AGOS_ADR320C	67.7536	0.0185	0
BNA3	AGOS_AEL170C	60.4804	0.004	0		BET3	AGOS_AGR300W	69.7115	0.018	0
BRX1	AGOS_AER020W	80.4795	0.0015	0		CAF40	AGOS_AEL194W	64.3432	0.017	0
CCT2	AGOS_AER415W	86.6412	0.0125	0		CDC28	AGOS_ADR058C	87.1186	0.0165	0
CCT3	AGOS_AGR314W	87.8505	0.001	0		CDC34	AGOS_AGL203C	81.1111	0.0163	0
CCT5	AGOS_AFR418W	84.2576	0.0113	0.00016		CDC42	AGOS_AGL093W	93.7173	0.0159	0
CCT7	AGOS_AFR067W	85.7664	0.0092	0		CDC5	AGOS_ACL006W	71.9323	0.0158	0
CCT8	AGOS_ACR045W	80.9947	0.0155	0		CDH1	AGOS_AFL007C	64.7913	0.0158	0.00095
DBP3	AGOS_AFL080W	73.0841	0.0102	0		CEG1	AGOS_AFL107W	69.5464	0.0156	0
DBP8	AGOS_AER301C	73.7931	0.0077	0		CKS1	AGOS_ABR109C	88.2353	0.0153	0
DIP2	AGOS_AGL196C	68.2353	0.0275	3E-07		CNB1	AGOS_AER096C	86.8571	0.0147	0
ECM16	AGOS_AFR222W	63.1877	0.0037	0		COF1	AGOS_ADR235W	86.014	0.0145	0

ENP2	AGOS_AFR468W	73.0044	0.0137	0
FCY1	AGOS_ADL328C	66.2252	0.0031	0
FOL2	AGOS_AGR335C	76.5432	0.0024	0
FUN12	AGOS_ADR373W	75.3125	0.0247	0
GAR1	AGOS_AEL096W	76.4151	0.0214	0
GFA1	AGOS_ABL036C	84.7026	0.0013	0
GLN1	AGOS_ACR182C	82.6558	0.0028	0
GLT1	AGOS_ADR290W	77.8588	0.0164	0
GRS1	AGOS_ADL373W	80.3951	0.0133	0
HGH1	AGOS_AAL150W	68.6327	0.0147	0
HIS3	AGOS_ADL270C	77.2727	0.0026	0
HIS4	AGOS_AFR703W	60.794	0.0046	0
IMP4	AGOS_AER430W	83.0389	0.0084	0
KRE33	AGOS_AGR346C	80.8129	0.0022	0
KRR1	AGOS_AFR744W	76.6764	0.0038	0
LOC1	AGOS_AGR001W	63.8191	0.005	0
LYS4	AGOS_ABL106C	66.3265	0.0044	0
MAK16	AGOS_ADR349W	65.9375	0.0025	0
MET13	AGOS_AAR170W	61.194	0.003	0
MRP10	AGOS_AER199C	70.5263	0.0242	0
MRPL19	AGOS_ABL144C	79.8658	0.0055	0
MRPS12	AGOS_AGL222W	70	0.0029	0
NIP7	AGOS_AFL104W	91.1602	0.0073	0
NMD3	AGOS_AER299C	81.4453	0.0199	0
NOC2	AGOS_ADL277W	63.8329	0.0168	0
NOP56	AGOS_ACL144C	79.0528	0.0047	0
NOP58	AGOS_AFR328C	88.1092	0.0054	0
NOP7	AGOS_AAR094W	71.3087	0.0041	0
NOP9	AGOS_ACR046W	63.8643	0.0034	0
NUC1	AGOS_ADR144C	61.0169	0.0258	0

CTA1	AGOS_AGL256W	76.9231	0.0124	0
DID2	AGOS_AFR068C	75.4902	0.012	0
DID4	AGOS_ABR002C	70.0855	0.0115	0
DMC1	AGOS_AGR101C	86.4865	0.0113	0.00044
DTD1	AGOS_ABL152W	66	0.011	0
DUN1	AGOS_AFL188C	65.8898	0.0106	0
DYS1	AGOS_AAL036W	78.534	0.0099	0
ELP3	AGOS_AGR201C	85.5	0.0098	0
ESA1	AGOS_ACR138W	80.4598	0.0096	0
ESS1	AGOS_ACL120W	65.0307	0.0093	0
FBP1	AGOS_AFR593C	75.4386	0.0086	0
FRQ1	AGOS_ACL163W	84.7368	0.0075	0
GCN3	AGOS_AFR084C	78.6184	0.0075	0
GCN5	AGOS_AER297C	75.4425	0.0074	0
GDB1	AGOS_AEL276C	62.78	0.0071	0
GLC3	AGOS_AEL044W	77.6671	0.0071	0
GLC7	AGOS_AFR166C	93.949	0.007	0
GLY1	AGOS_AFR366W	75.6544	0.0066	0
GPA1	AGOS_ADR153C	73.1111	0.0065	0
GTR1	AGOS_AFR446W	80.9365	0.0058	0
GTR2	AGOS_AGL046C	75.8112	0.0057	0
HOG1	AGOS_AGR048C	78.3664	0.0056	0
HOS2	AGOS_AFR172C	80.1782	0.0053	0
HTA1	AGOS_AEL003C	98.4733	0.0053	0
HTB1	AGOS_AEL002W	90.5512	0.0052	0
HTB2	AGOS_AGR183C	93.1818	0.005	0
HTZ1	AGOS_ACL017C	91.7293	0.0044	0
ICL1	AGOS_ADL066C	69.6429	0.0044	9E-08
KAP95	AGOS_ACL187W	72.9384	0.0044	4.7E-05
KIN28	AGOS_ADL283W	74.8366	0.0043	0

PMI40	AGOS_ADR003C	72.7273	0.0287	0
POT1	AGOS_AFR302W	69.4789	0.0036	0
PUS1	AGOS_AFL105C	63.0682	0.0035	3.4E-07
RIO1	AGOS_AER257W	69.1824	0.0028	0
RML2	AGOS_AAR128W	70.1299	0.0031	1.4E-06
RPC25	AGOS_ACR049W	79.5238	0.0017	0
RPF2	AGOS_ADR215C	73.7463	0.0148	0
RRB1	AGOS_ADR242C	71.5931	0.0175	0
RRP9	AGOS_AAR102C	62.4365	0.0035	0
SCH9	AGOS_ADL389W	68.3544	0.0031	0
SOF1	AGOS_AAL157C	76.618	0.0103	0
SPB1	AGOS_AFR734C	76.0241	0.0144	0
TCP1	AGOS_AGL092W	87.8136	0.0011	4E-07
TIF6	AGOS_ADL030C	93.8775	0.0044	0
TMA22	AGOS_ACL112C	65.8291	0.0026	0
TRM1	AGOS_AGR075W	64.2857	0.0033	3.5E-07
TRM11	AGOS_ADL161C	69.2661	0.0029	0
TRM112	AGOS_ADR166W	72.5926	0.0032	0.00054
TUF1	AGOS_AAR143W	85.3488	0.0167	0
UGA1	AGOS_AGL050C	71.2215	0.0025	0
UTP11	AGOS_ABL032C	71.371	0.0043	0
UTP13	AGOS_AGR180W	64.1439	0.0033	0
UTP15	AGOS_ABL044C	71.2355	0.0199	1E-08
UTP22	AGOS_ADR029W	63.9209	0.0034	0
UTP6	AGOS_AER317W	63.8009	0.003	0
UTP7	AGOS_ACL034W	71.0909	0.0254	0
YKE2	AGOS_ACL146C	66.3551	0.0036	0
YTA12	AGOS_ABL041W	60.5134	0.0037	0
YTM1	AGOS_AER337W	76.6292	0.002	0
ZWF1	AGOS_ABL206C	68.75	0.0028	0

LIP5	AGOS_AGR231C	76.1518	0.0043	0.00041
LST8	AGOS_AAL009C	85.8086	0.0042	0.00238
LTP1	AGOS_ADL365W	64.3312	0.0041	0
MAD2	AGOS_ACR043W	70.8543	0.0041	0
MCM3	AGOS_AFR355C	65.5355	0.004	0
MCM5	AGOS_AGR276W	71.5259	0.0039	0
MCM7	AGOS_ADR041W	67.6507	0.0039	0
MET14	AGOS_ABR001W	66.0465	0.0038	0
MET30	AGOS_ADL082C	64.4366	0.0038	0
MPE1	AGOS_ABR070C	60.3535	0.0038	0
NCB2	AGOS_AFR397C	69.7987	0.0038	0
NUP170	AGOS_AER397C	60.084	0.0038	0
PAP1	AGOS_ABL002C	68.5619	0.0037	0
PFK1	AGOS_AEL208W	69.9088	0.0037	1E-08
PFY1	AGOS_ACL168C	83.3333	0.0037	0.00012
PHO88	AGOS_ADL332C	69.3122	0.0036	0
PIL1	AGOS_AEL329W	86.4238	0.0036	0
PMR1	AGOS_AEL301W	71.0554	0.0036	0
PMT4	AGOS_ABL085W	64.0687	0.0035	0
POB3	AGOS_AER138C	76.7528	0.0035	0
PPG1	AGOS_AER265W	76.9679	0.0035	0
PPT1	AGOS_AAR083C	65.0198	0.0035	0
PRE7	AGOS_AGL324W	79.8354	0.0033	0
PRS3	AGOS_AGL080C	88.125	0.0033	2E-08
PSF1	AGOS_AAR188W	69.4581	0.0033	0.00018
PSF2	AGOS_ADR121W	62.0853	0.0032	0
QCR6	AGOS_ADL124C	61.9048	0.0032	0
QCR8	AGOS_AEL121W	75.5319	0.0032	0
QCR9	AGOS_AAL145W	72.7273	0.0031	0
QNS1	AGOS_AGR358W	79.021	0.0031	0

RAD27	AGOS_ABL052C	73.2804	0.0031	0
RAD54	AGOS_AEL297W	66.9274	0.0031	0
RAD6	AGOS_AAR156C	97.6471	0.0031	0
RBG2	AGOS_AAL134W	86.1413	0.003	0
RDI1	AGOS_ADR098C	81.592	0.003	0
RFC5	AGOS_ADL303C	64.7887	0.003	0
RHO2	AGOS_ACL087C	78.6096	0.0029	0
RHO3	AGOS_ADL252W	77.6786	0.0029	0
RPN1	AGOS_ACR072C	72.0202	0.0028	0
RPN5	AGOS_ADR073W	73.4694	0.0028	0
RPN6	AGOS_ADR280W	73.9234	0.0028	0
RPT1	AGOS_ACR050C	87.3684	0.0027	0
RPT2	AGOS_AEL011W	92.9062	0.0027	0
RRD2	AGOS_ACR139C	61.5599	0.0027	0
RRP4	AGOS_AAL034C	69.8006	0.0027	0
RSR1	AGOS_AFR464W	62.8788	0.0026	0
RUB1	AGOS_AGL185W	75	0.0025	0
RVS161	AGOS_AER193W	84.9057	0.0024	0
RVS167	AGOS_AFR140C	65.2062	0.0024	0
SEC14	AGOS_ABR020W	77.5974	0.0024	0
SEC22	AGOS_AGR267W	68.6916	0.0023	0
SEC26	AGOS_ABL112W	64.2636	0.0023	0
SEC27	AGOS_AFL118W	68.6298	0.0023	0
SEH1	AGOS_AFL038C	71.5152	0.0023	0
SIT4	AGOS_AER202C	94.822	0.0023	0
SLY1	AGOS_AGL120W	62.6168	0.0023	0.00081
SMC2	AGOS_AGR236W	60.8547	0.0022	0
SMT3	AGOS_AFR697C	89.1304	0.0022	0
SNF2	AGOS_AFR562C	60.4844	0.0022	0
SOD1	AGOS_AGL321W	72.7273	0.0022	0

SOD2	AGOS_ADR160W	72.2467	0.0022	0
SOH1	AGOS_AAR164C	63.7795	0.0021	0
SPT15	AGOS_AGR134W	83.682	0.0021	0
SPT3	AGOS_AFR388W	77.2036	0.0021	0
SPT4	AGOS_AFR576C	86.2745	0.0021	0
SUB2	AGOS_ADR260C	91.7808	0.0021	0
TAF14	AGOS_ACR099C	71.6599	0.0021	0
TAF6	AGOS_AFL020W	70.0397	0.0021	2E-08
TEM1	AGOS_AER132W	62.069	0.002	0
TFB3	AGOS_ABR202C	72.0126	0.0019	0
TFG2	AGOS_AFR502C	69.0418	0.0019	0
TOA2	AGOS_ABR071W	69.6429	0.0019	0
TPD3	AGOS_ACR279C	73.6318	0.0018	0
TPS1	AGOS_ADL378W	77.7328	0.0018	0
TRA1	AGOS_AER393C	65.4585	0.0018	0
TUP1	AGOS_AGL234W	63.434	0.0018	0
UBA4	AGOS_AER248W	61.851	0.0018	0
UBC13	AGOS_AGR121C	82.4675	0.0018	0.03905
UBC4	AGOS_AER173C	93.8775	0.0017	0
UBC7	AGOS_ABR059W	81.8182	0.0017	0
UBC8	AGOS_AEL045W	71.09	0.0017	0
UBC9	AGOS_AER056C	80.8917	0.0017	0
UFD1	AGOS_AFR662C	70	0.0017	0
URM1	AGOS_ADL014W	71	0.0016	0
VMA11	AGOS_AFL141C	85.9756	0.0016	0
VMA16	AGOS_ACR071W	77.7251	0.0016	0
VMA2	AGOS_ADL380W	88.9535	0.0016	0
VMA4	AGOS_ADR358W	60.262	0.0015	0
VMA6	AGOS_AER146C	78.9625	0.0014	0
VMA7	AGOS_AGL052W	74.5763	0.0013	0

VMA8	AGOS_AAR130C	72.2022	0.0012	0
VPH1	AGOS_ADR177C	60.9058	0.0011	0
VPS4	AGOS_AEL265W	84.6868	0.001	0
VTC4	AGOS_AGR316C	70.4067	0.001	0
YAH1	AGOS_AFL169C	70.8609	0.001	0
YCR034V	AGOS_AFR624W	74.9288	0.001	0
YKT6	AGOS_AER109W	79	0.001	0
YPT32	AGOS_AER434C	86.758	0.001	0
YPT52	AGOS_AAL176C	69.2683	0.001	0
YPT7	AGOS_ACR003C	88.9423	0.001	0
ZPR1	AGOS_ACL137C	75.891	0.001	0

Yarrowia lipolytica									
**Meiosis Initiation**					**Meiosis Commitment**				
**S.cerevisae gene name**	**Yarrowia lipolytica gene name**	**% of S.cerevisae gene identical to target Yarrowia lipolytica gene**	**dN/dS**	**MEGA P value**	**S.cerevisae gene name**	**Yarrowia lipolytica gene name**	**% of S.cerevisae gene identical to target Yarrowia lipolytica gene**	**dN/dS**	**MEGA P value**
ARG3	YALI0_D14894g	53.0488	0.0068	0	AHA1	YALI0_B15840g	44	0.007	0
ARO1	YALI0_F12639g	51.9923	0.0067	0.02086	AIP1	YALI0_E01870g	39.4415	0.0094	2.7E-06
BRX1	YALI0_B11880g	65.7343	0.0036	0	AMD1	YALI0_E11495g	50.6329	0.0047	0
BUD23	YALI0_F06512g	68.3019	0.022	0	APS2	YALI0_B04246g	52.0833	0.0058	0
CCT2	YALI0_F11473g	76.5714	0.0029	0	ARC18	YALI0_B20240g	64	0.0047	0
CCT7	YALI0_C20999g	69.4946	0.0035	0	ARL3	YALI0_D02995g	62.1053	0.0045	3.4E-31
CCT8	YALI0_D11220g	56.328	0.0059	0	ASF1	YALI0_A01375g	62.6415	0.0038	0

CYS3	YALI0_F05874g	66.2437	0.0033	6E-08
DIA4	YALI0_B05918g	40.2128	0.0077	0.0002
DIP2	YALI0_C00913g	45.614	0.0069	0
EBP2	YALI0_E05797g	45.8763	0.0075	0
ECM16	YALI0_D26620g	49.3238	0.0061	0.00029
FCF1	YALI0_E23386g	75.1295	0.0052	0
GDH2	YALI0_E09603g	44.3769	0.0066	0
GIM3	YALI0_A01738g	40.1515	0.008	1.4E-07
GLT1	YALI0_B19998g	64.6531	0.0046	3E-08
GRX5	YALI0_F21219g	58.2781	0.0299	0.00099
GUF1	YALI0_F10725g	55.4878	0.005	0
HGH1	YALI0_F07315g	49.5845	0.0125	0
HIS4	YALI0_A15950g	52.6316	0.0099	0
IFM1	YALI0_B21978g	32.1046	0.0083	0
KRE33	YALI0_B14751g	67.7419	0.0081	0.00006
KRI1	YALI0_D00957g	32.9289	0.0095	0.00717
LOC1	YALI0_D19624g	43.3673	0.0088	5.6E-08
LYS2	YALI0_E06457g	53.0786	0.0058	0
LYS9	YALI0_D22891g	68.4564	0.0037	0
MAK16	YALI0_C08052g	58.0128	0.0041	0
MAK21	YALI0_D15356g	35.5191	0.0083	0.00874
MEF2	YALI0_C18557g	39.6509	0.0088	0.0008
MNP1	YALI0_F17556g	58.8957	0.0069	0
MRP2	YALI0_D21626g	30.4762	0.0093	9E-11
MRPL16	YALI0_C18139g	38.2883	0.0092	4.8E-11
MRPL19	YALI0_D22088g	55.9211	0.0116	0
MRPL37	YALI0_F18150g	33.0189	0.027	4.3E-05
MRPL7	YALI0_B01892g	45.7912	0.0264	5.4E-09
MRPS16	YALI0_E09691g	58.4906	0.0057	0
MRPS9	YALI0_B06116g	30.4075	0.0106	0

ATP18	YALI0_D17490g	46.7742	0.0074	0
ATP19	YALI0_B11913g	41.4286	0.0075	5E-08
ATP20	YALI0_B21527g	36.9565	0.0076	3E-08
ATP4	YALI0_F20306g	52.9954	0.0058	0.00116
BNA5	YALI0_B22902g	50.8009	0.0058	0.00381
CBK1	YALI0_B04268g	61.7347	0.0041	7.8E-33
CCP1	YALI0_F20504g	45.8824	0.006	5.6E-34
CCS1	YALI0_F30877g	42.735	0.0155	2.3E-07
CDC28	YALI0_B10758g	68.6709	0.0029	9E-12
CDC45	YALI0_B06369g	34.29	0.0092	0
CKS1	YALI0_F18700g	75.7576	0.0029	8E-11
CNB1	YALI0_A19976g	64.7399	0.0043	0
COF1	YALI0_F20856g	72.549	0.0023	0
COX6	YALI0_E10144g	62	0.0036	5.7E-29
DID2	YALI0_C10098g	55.6701	0.0048	0
EMP24	YALI0_E34852g	52	0.0041	1.3E-07
ERV46	YALI0_C13112g	36.9077	0.0146	0.00345
GLC7	YALI0_A08077g	85.0153	0.0013	0
GLY1	YALI0_A21417g	49.4382	0.0067	0
GOS1	YALI0_D23353g	35.6164	0.0087	0.00361
GPA1	YALI0_E11627g	45.6098	0.0068	0.00017
GPH1	YALI0_F04169g	61.3739	0.0039	0
GPN2	YALI0_D19888g	56.1047	0.0131	0
GTR2	YALI0_D10307g	61.2308	0.004	0
GYP7	YALI0_F31911g	34.5205	0.0138	0.00035
HOS3	YALI0_A20834g	31.4685	0.0084	0.00359
HTA1	YALI0_E26477g	83.7037	0.0037	0
HTZ1	YALI0_F02827g	71.4286	0.0033	4.5E-29
HUB1	YALI0_A02871g	67.1429	0.0075	0
IST1	YALI0_B04048g	35.6364	0.0099	0

NIP7	YALI0_E34287g	76.1111	0.0024	0
NOC4	YALI0_F20284g	31.5094	0.0381	0
NOP10	YALI0_C00693g	82.2581	0.004	0
NOP58	YALI0_B00946g	66.9903	0.0034	0
NOP7	YALI0_B23342g	57.1186	0.0043	0
NOP9	YALI0_D11242g	32.4586	0.0087	6E-08
NUC1	YALI0_D05071g	58.7302	0.0161	0
PCM1	YALI0_E29579g	49.8113	0.0076	0.02101
RKI1	YALI0_B06941g	53.4979	0.0194	0.00021
RPA12	YALI0_A08151g	66.129	0.0064	0
RPA49	YALI0_E05225g	33.4146	0.0107	4.5E-06
RPC19	YALI0_E27676g	47.8571	0.0081	0
RPC25	YALI0_D11396g	50.495	0.0063	0.00112
RPF2	YALI0_A03905g	61.9938	0.0036	0
RRB1	YALI0_C16203g	47.3373	0.0111	0.00149
RRP12	YALI0_E07425g	39.8986	0.0089	0
RRP36	YALI0_B03784g	32.8571	0.0122	7.8E-08
RRP9	YALI0_A20614g	48.4787	0.0071	2E-08
RRS1	YALI0_A12067g	58.2524	0.0133	0
SAS10	YALI0_F20372g	33.9893	0.0098	0.00016
SDO1	YALI0_F25267g	51.2295	0.0055	1.4E-06
SOF1	YALI0_D14080g	54.7771	0.01	0
SPB1	YALI0_D09251g	53.5294	0.0046	0
TIF6	YALI0_E27940g	74.3902	0.0032	0
TRM1	YALI0_E30723g	49.187	0.0226	0
TRM10	YALI0_F12771g	32.0755	0.0076	2.3E-05
TRM11	YALI0_C13882g	50.2304	0.0057	1.2E-05
TRP5	YALI0_F24893g	65.6069	0.004	0
TSR1	YALI0_B08756g	36.9748	0.0088	0
TUF1	YALI0_F24387g	72.6636	0.0047	0

KGD2	YALI0_E16929g	57.7181	0.0068	0
LIP5	YALI0_E10571g	68.661	0.0036	7.7E-28
MET17	YALI0_D25168g	66.3529	0.0052	2E-08
MOT1	YALI0_F31053g	48.3146	0.01	0
MSH6	YALI0_F26499g	44.3864	0.0071	0
MTR10	YALI0_D01133g	34.1102	0.0103	1.7E-05
NRK1	YALI0_B14729g	35.0394	0.0091	0.00111
NUP57	YALI0_A20702g	31.6288	0.0106	0.02748
PHO23	YALI0_E08822g	30	0.0116	0.00021
PHO81	YALI0_A00759g	34.1117	0.0098	0
PMR1	YALI0_E09471g	55.9267	0.0072	0
POB3	YALI0_D25058g	49.0809	0.0057	0
POL1	YALI0_E11869g	37.0909	0.0087	0
PPT1	YALI0_B06963g	45.929	0.007	0
PRB1	YALI0_A06435g	55.6263	0.0059	0
PRC1	YALI0_A18810g	60.6491	0.0035	3.2E-06
PRE7	YALI0_D06523g	65.3226	0.004	8.9E-31
PRI2	YALI0_F04576g	35.1812	0.0088	0
PRP16	YALI0_B09053g	41.3185	0.007	0
PRX1	YALI0_F08195g	56.746	0.0047	1E-05
QNS1	YALI0_A20108g	72.1986	0.003	0
RAD14	YALI0_B01474g	40.4332	0.0077	0.00223
RAD16	YALI0_F01232g	56.2796	0.0043	0
RAD50	YALI0_D15246g	35.3715	0.0088	0
RAD54	YALI0_B07513g	56.1338	0.0049	3.4E-34
RAD6	YALI0_F26697g	74.8344	0.0027	0
RDS2	YALI0_E10087g	44.3515	0.0062	4.5E-41
RET2	YALI0_E32542g	35.8382	0.009	0
RHO3	YALI0_F17270g	74.0196	0.0137	0
RPN5	YALI0_D09977g	55.0336	0.005	0

UGA1	YALI0_E18238g	54.2169	0.0047	0
UTP11	YALI0_E01936g	46.7213	0.0065	0
UTP15	YALI0_E01628g	45.0902	0.0075	0
UTP21	YALI0_B13442g	43.0657	0.0078	0
UTP22	YALI0_E31625g	30.7692	0.0115	0
UTP6	YALI0_F14377g	30.9829	0.0091	0.01814
YAR1	YALI0_B20900g	38.6598	0.0089	6.8E-06
YFH1	YALI0_E15268g	36.3636	0.0096	0
YGR054W	YALI0_C08877g	36.7201	0.0262	2.9E-06
YKE2	YALI0_D17086g	37.2881	0.0088	0
YMR035W	YALI0_B10978g	32.4607	0.0108	6.6E-06

RPT1	YALI0_D11418g	86.4679	0.004	6.7E-33
RPT2	YALI0_F02585g	80.7339	0.0017	0
RRD1	YALI0_B02772g	32.1759	0.012	0.01685
RRD2	YALI0_E04642g	46.0317	0.0053	9.9E-34
RRP4	YALI0_D05511g	54.955	0.006	1.2E-22
RSR1	YALI0_F23177g	46.4567	0.0074	0
RUB1	YALI0_F18403g	51.9481	0.0059	0
RVS167	YALI0_D13706g	56.8345	0.0056	2.2E-07
SEA4	YALI0_D18788g	35.3568	0.009	0
SEC17	YALI0_C23947g	42.4658	0.0076	0
SEH1	YALI0_B16610g	53.2915	0.0052	0
SEN15	YALI0_D26576g	32.7103	0.01	0.00016
SIN3	YALI0_D26315g	35.7564	0.008	0.00129
SLY1	YALI0_D20416g	39.322	0.0093	0.00064
SMC1	YALI0_E15620g	33.1967	0.0099	0.0028
SMC2	YALI0_F24783g	44.2833	0.007	0
SMT3	YALI0_F06826g	57.7778	0.0119	0
SOD2	YALI0_B08921g	52.4017	0.0039	6.7E-40
SPT16	YALI0_D14652g	46.2612	0.0065	9.2E-07
SPT3	YALI0_E21417g	51.5152	0.0054	0
SPT4	YALI0_F13354g	49.0909	0.0064	0.04661
TAF2	YALI0_C13904g	32.5949	0.0095	0.04241
TAZ1	YALI0_C14036g	35.7527	0.0095	1.6E-06
TFB4	YALI0_C04367g	35.8824	0.0166	0
TOA2	YALI0_F22136g	41.4414	0.007	0
TPS1	YALI0_E14685g	68.8699	0.0033	3.4E-17
TUP1	YALI0_A14542g	42.5039	0.0071	0
UBA2	YALI0_D06259g	34.876	0.0095	0.04561
UBA4	YALI0_C18095g	45.9596	0.0075	0
UBC11	YALI0_F05764g	58.9888	0.007	8.9E-33

UBC9	YALI0_B22638g	61.6352	0.0046	4.5E-33
URM1	YALI0_E19481g	43.4343	0.0081	0.02725
VMA11	YALI0_E31471g	82.6087	0.0022	7.8E-08
VMA4	YALI0_F15631g	50.2203	0.0063	3.4E-34
VPH1	YALI0_F31119g	55.0995	0.0054	0
VPS27	YALI0_B04070g	30.6195	0.0111	2.3E-05
VPS28	YALI0_A18722g	39.0244	0.009	0.00064
VTI1	YALI0_B02244g	45.2055	0.0077	0.00011
YAH1	YALI0_B02222g	63.9752	0.0069	0
YCH1	YALI0_D18293g	32.5	0.0134	0.00044
YIP1	YALI0_D04829g	46.6387	0.0081	0
YKT6	YALI0_E21329g	63.5	0.004	0
YRA1	YALI0_A20867g	30.0448	0.0116	9E-05
YRB1	YALI0_B15081g	43.4944	0.0036	0
YTA7	YALI0_F23397g	38.4263	0.0079	0

Debaryomyces hansenii									
**Meiosis Inititation**					**Meiosis Commitment**				
**S.cerevisae gene name**	**Debaryomyces hansenii RefSeq nucleotide ID**	**% of S.cerevisae gene identical to target D. hansenii gene**	**dN/dS**	**MEGA P value**	**S.cerevisae gene name**	**Debaryomyces hansenii RefSeq nucleotide ID**	**% of S.cerevisae gene identical to target D. hansenii gene**	**dN/dS**	**MEGA P value**
RPS10A	XM_461673.1	75.728	0.0017	0	HHF1	XM_456790.1	96.117	0.0013	0
RPS10B	XM_461673.1	73.786	0.0018	0	ELP3	XM_459174.1	90.128	0.0013	0
CCT3	XM_462464.1	79.588	0.002	0	RPT1	XM_459634.1	82.227	0.0014	0

TMA19	XM_460061.1	73.054	0.0023	0
CCT4	XM_457113.1	78.394	0.0024	5.2E-06
RPO31	XM_462102.1	76.876	0.0024	0
GUK1	XM_461891.1	70.968	0.0027	0
GAR1	XM_457810.1	82.727	0.0035	0
HYP2	XM_460907.2	85.806	0.0036	0
KRR1	XM_459920.1	83.013	0.004	0
NSA2	XM_458305.1	84.674	0.0047	0
MAK16	XM_460444.1	71.901	0.0065	0
TCP1	XM_462158.1	83.453	0.0066	2.2E-06
CCT5	XM_2770840.1	75.315	0.0068	0.00365
ANB1	XM_460907.2	83.226	0.007	0
TIF6	XM_459497.1	93.469	0.0079	0
NOP56	XM_461822.1	78.571	0.0083	0
KRE33	XM_461271.1	75.215	0.0084	0
MSN2	XM_456693.1	72.414	0.0086	0.02056
EFT2	XM_461796.1	86.698	0.0087	0
SAM1	XM_459923.1	79.634	0.0091	0
SAM2	XM_459923.1	79.894	0.0094	0
TUF1	XM_458853.1	77.882	0.0098	0.00579
CCT2	XM_456801.1	78.558	0.0099	0
HIS3	XM_458336.1	75.113	0.0112	0
NIP7	XM_457873.1	79.444	0.0113	4E-08
IMD2	XM_460442.1	70.385	0.0118	0.00078
HAS1	XM_462428.1	86.768	0.0126	0
NOP10	XM_459477.1	87.931	0.0131	0
IMD3	XM_460442.1	72.308	0.0133	8E-07
TRP5	XM_459006.1	76.346	0.0134	0.0086
IMD4	XM_460442.1	73.41	0.0135	0
NMD3	XM_462561.1	73.904	0.0137	0.0068

YKT6	XM_458810.1	74.5	0.0019	0
ARF1	XM_459420.1	82.222	0.002	0
ARF2	XM_459420.1	80.663	0.0024	0
DMC1	XM_460030.1	75.46	0.0024	0
TRR1	XM_461900.2	75.235	0.0026	0
RVS161	XM_462051.1	70.943	0.0026	0
GCN5	XM_462560.1	74.595	0.0028	0
YPT32	XM_457123.1	71.493	0.0028	0
QNS1	XM_456405.1	70.085	0.003	0
ASF1	XM_456774.1	89.82	0.0031	0
CBK1	XM_460922.1	79	0.0031	0
SPT15	XM_2770577.1	80.342	0.0036	0
NHP6B	XM_459256.1	75	0.0051	0
SFP1	XM_458979.1	80.952	0.0055	0
ACT1	XM_458703.2	97.067	0.0059	0
VPS4	XM_459738.1	76.212	0.0064	0
VMA2	XM_460251.1	92.149	0.0067	0
SAH1	XM_462409.1	82.405	0.0067	0
UBC4	XM_461820.1	91.096	0.0074	0
ACO2	XM_460250.1	76.726	0.0076	0
GLC7	XM_459484.1	92.409	0.0077	0
PRS3	XM_461698.1	85.938	0.0079	0
PRE7	XM_462322.1	77.917	0.0087	0
SNZ2	XM_460422.1	72.054	0.0087	0
MSN4	XM_456693.1	74.576	0.0088	0
GAT1	XM_459410.2	81.481	0.0092	0
SIT4	XM_459045.1	82.803	0.0098	0
COF1	XM_457295.1	86.014	0.0099	0
HTB1	XM_459208.1	90.625	0.0103	0
ATG8	XM_458639.2	80.172	0.0103	0

LYS9	XM_461119.1	71.749	0.0138	0
ASN2	XM_457374.1	75.439	0.0151	0.25561
ASN1	XM_457374.1	75.35	0.0154	0
GLN1	XM_462371.1	77.747	0.0156	0
GRS1	XM_459646.1	71.233	0.0165	0
ARG1	XM_457073.1	71.19	0.0171	0
GFA1	XM_461639.1	73.78	0.0177	0
CYS3	XM_458365.1	70.361	0.0185	0.00045
BRX1	XM_461968.1	70.447	0.022	0

RNR1	XM_461790.1	71.094	0.0106	0
CDC42	XM_462156.1	87.435	0.011	0
RAD6	XM_458283.1	78.395	0.011	0
YEF3	XM_461401.1	77.29	0.011	0
YJL045W	XM_459635.1	76.863	0.0112	0
SUB2	XM_460627.2	77.803	0.0115	0
DYS1	XM_461646.1	78.307	0.0119	0
TRR2	XM_461900.2	71.473	0.0119	0
TPS1	XM_460256.1	75.372	0.012	0
HOG1	XM_460213.1	80.679	0.0123	0
LST8	XM_462013.1	72.671	0.0131	0
VMA8	XM_459696.1	73.684	0.0142	0
KAE1	XM_460295.1	74.536	0.0143	0
CDC21	XM_2770134.1	72.669	0.0145	0
HAP3	XM_2770822.1	87.629	0.0157	0
PPH22	XM_459147.1	84.483	0.0158	0
UBC13	XM_459842.1	79.054	0.0158	0
PYC2	XM_458082.1	74.377	0.016	0
PIL1	XM_460437.1	81.752	0.0208	0
CKS1	XM_460281.1	83.333	0.0209	0
HTA1	XM_461153.1	90.909	0.0039	0
HTA2	XM_461153.1	90.909	0.0042	0
SEC14	XM_459849.1	72.185	0.0056	0
SNC1	XM_2770216.1	71.552	0.0024	0
SOD2	XM_461755.1	70.135	0.0031	0
KIN28	XM_462466.1	78.792	0.0033	0
RRP4	XM_462289.1	77.674	0.0036	0
YRB1	XM_457079.1	76.995	0.0069	0
PRB1	XM_458282.1	76.744	0.0046	0
ECM22	XM_462155.1	76.139	0.0058	0

CLA4	XM_457495.1	76.061	0.006	0
RDS2	XM_2777682.1	82.629	0.0044	3E-07
UBC7	XM_456449.1	72.84	0.0035	2.5E-06
PPZ2	XM_459586.1	83.706	0.0041	3.8E-06
FKS1	XM_457762.1	74.102	0.0164	1.9E-05
HTZ1	XM_459571.1	86.607	0.0018	0.00408
RSP5	XM_458629.1	73.659	0.002	0.00471
GPN2	XM_461157.2	73.125	0.0035	0.00471
ENO1	XM_462151.1	78.36	0.0103	0.01084
VMA16	XM_460013.2	77.368	0.0043	0.01358
ADH1	XM_462457.1	74.64	0.0094	0.01385
YAH1	XM_458973.1	79.839	0.0031	0.019
ENO2	XM_462151.1	77.677	0.0099	0.02121
SNC2	XM_2770216.1	73.913	0.0022	0.02178
LIP5	XM_2770617.1	75.637	0.0072	0.029

Aspergillus fumigatus									
**Initiation**					**Commitment**				
**S.cerevisae gene name**	**A. fumigatus gene name**	**% of S.cerevisae gene identical to target A. fumigatus gene**	**dN/dS**	**MEGA P value**	**S.cerevisae gene name**	**A. fumigatus gene name**	**% of S.cerevisae gene identical to target A. fumigatus gene**	**dN/dS**	**MEGA P value**
HAS1	AFUB_070250	58.3601	0.0027	0	AIP1	AFUB_023120	35.182	0.0011	0
KRR1	AFUB_027140	62.5698	0.0029	0	GLC7	AFUB_005320	87.3065	0.0012	0
NSA2	AFUB_041020	61.0561	0.003	0	MCM3	AFUB_004320	48.0942	0.0012	0.00027
TIF6	AFUB_053550	73.2794	0.0031	0	SPT3	AFUB_013520	40.884	0.0012	0.00329
ARG1	AFUB_021370	68.2692	0.0032	0	SAH1	AFUB_009540	80.4933	0.0018	0
CCT3	AFUB_036160	70.7407	0.0032	0	ERV1	AFUB_040250	33.1818	0.0019	0.00046
TCP1	AFUB_031700	70.6714	0.0032	0	RAD6	AFUB_000540	76.8212	0.002	0

RET1	AFUB_002850	63.6667	0.0036	0
NOP56	AFUB_039570	60.5364	0.0038	0
GUK1	AFUB_008250	51.3158	0.004	0
TUF1	AFUB_011640	64.5455	0.004	0
NMD3	AFUB_032420	56.9549	0.0042	0
ALA1	AFUB_083700	57.8635	0.0043	0
CCT2	AFUB_002140	70.9156	0.0044	0
MAK16	AFUB_094040	52.1605	0.0045	0
RPO31	AFUB_034860	55.1961	0.0051	0
MET13	AFUB_027060	50.2439	0.0051	1.2E-05
CCT8	AFUB_066850	56.7901	0.0053	0
FCF1	AFUB_020410	53.6458	0.0054	0
BMS1	AFUB_029210	50	0.0056	0
MRPL23	AFUB_026300	48.2353	0.0058	4.2E-05
DBP3	AFUB_023390	52.0875	0.006	0.01215
HIS3	AFUB_093600	56.1475	0.0061	0
BNA3	AFUB_068220	49.5536	0.0062	3E-07
PMI40	AFUB_012770	43.326	0.0069	1.1E-07
UTP7	AFUB_028510	45.2336	0.0069	0.00594
ARG3	AFUB_064280	44.7439	0.0072	0
RML2	AFUB_069150	42.3267	0.0072	0.00167
HIS4	AFUB_014100	45.5275	0.0074	0
MNP1	AFUB_066860	45.4545	0.0075	0
SDO1	AFUB_001210	31.9767	0.0075	0
YTM1	AFUB_064720	41.8033	0.0079	0.00725
DRS1	AFUB_014550	36.8675	0.008	0
ENP2	AFUB_021820	38.5576	0.008	0
RRB1	AFUB_076370	43.5484	0.0081	0.0067
NCL1	AFUB_013720	30.9255	0.0081	0.009
GUD1	AFUB_099250	35.3591	0.0081	5.9E-05

GPN2	AFUB_076670	47.5066	0.0025	0
SIT4	AFUB_077480	57.2165	0.003	0
SUB2	AFUB_095680	70.7589	0.0032	0
CNB1	AFUB_093760	56.9948	0.0036	0
DID4	AFUB_061110	60.5634	0.0037	0
RBG2	AFUB_054330	66.5746	0.0037	0
VMA8	AFUB_059480	71.8232	0.0037	0
SPT15	AFUB_039050	60.424	0.004	0
LST8	AFUB_009010	46.747	0.0041	4.3E-05
RHO3	AFUB_042360	64.467	0.0042	0
SOD1	AFUB_056780	63.2911	0.0043	0
KAE1	AFUB_093790	68.4659	0.0043	0.0005
GPH1	AFUB_012400	60.6371	0.0044	0
MET16	AFUB_042510	50	0.0044	0
VMA16	AFUB_031210	64.3216	0.0044	0.00658
VMA6	AFUB_035820	51.7906	0.0046	0.01529
MCM5	AFUB_051050	62.5348	0.0046	0.01989
UBC7	AFUB_052570	60.8434	0.0046	0.00067
DYS1	AFUB_050270	58.3554	0.0049	0
PPH3	AFUB_059590	43.6709	0.0049	0
BET2	AFUB_090000	57.8073	0.0051	0
HOS2	AFUB_020880	50.924	0.0051	0
VTC4	AFUB_024930	50.437	0.0051	4E-08
HTZ1	AFUB_050470	70.2899	0.0051	0.00007
UBC11	AFUB_001590	51.9337	0.0052	0.00657
MNN10	AFUB_030560	37.2294	0.0053	0
COX6	AFUB_051280	47.4684	0.0053	0.0006
CDC31	AFUB_068190	31.0078	0.0054	0
SOD2	AFUB_068610	52.8384	0.0055	0
VMA11	AFUB_056100	62.1795	0.0055	2.3E-06

MET12	AFUB_051990	42.09	0.0082	0.00067
HGH1	AFUB_043720	37.6238	0.0086	1E-08
DBP7	AFUB_058620	36.0158	0.0088	0
MRPL16	AFUB_027920	37.6623	0.0091	6.8E-05
SPB4	AFUB_035920	34.5622	0.0092	3.8E-06
TMA22	AFUB_083860	36.5979	0.0093	0.00492
NOP14	AFUB_083330	30.6681	0.0095	0
DIA4	AFUB_036530	33.0754	0.0097	0.02493
UTP21	AFUB_043690	33.7617	0.0098	0.00006
MRPL8	AFUB_008860	35.567	0.0103	5.7E-07
MRPS9	AFUB_093740	32.381	0.0106	0.00723
MEF2	AFUB_054700	31.9565	0.011	0
ARO3	AFUB_002490	60.1064	0.011	0.00203
RCL1	AFUB_082860	35.4839	0.0111	0.00058
ERB1	AFUB_008620	48.7437	0.012	0.00519
EBP2	AFUB_059900	40.7895	0.0133	7.9E-05
NIP7	AFUB_032720	58.3784	0.0136	0
IMP4	AFUB_012550	56.5657	0.0203	0.00833
UTP25	AFUB_008740	34.7243	0.0215	0
TRM11	AFUB_069120	38.4458	0.0239	0
LYS2	AFUB_068270	48.9123	0.027	0.00011

TOA2	AFUB_031330	51.3514	0.0055	0.00056
ARL3	AFUB_016360	43.7751	0.0058	6E-05
SEC22	AFUB_094140	49.3023	0.006	0.03241
ASF1	AFUB_038110	47.5177	0.0061	0
ICL1	AFUB_070430	58.3643	0.0061	0
YAH1	AFUB_052400	45.7711	0.0061	0
PRP22	AFUB_058430	45.0406	0.0062	0
RFC5	AFUB_027930	49.8592	0.0062	0.00251
PMR1	AFUB_022890	49.3874	0.0063	0
RSR1	AFUB_056480	48.0769	0.0063	0
ADA2	AFUB_026420	39.1732	0.0064	0
PRS5	AFUB_035830	50.9174	0.0064	6.5E-06
POB3	AFUB_090940	43.4555	0.0064	0.00077
RPN1	AFUB_059290	48.6188	0.0065	0.01554
RSP5	AFUB_008950	63.5603	0.0066	0
SEC27	AFUB_026390	48.8889	0.0066	0
APC11	AFUB_087950	44.7619	0.0066	0.00042
SPT16	AFUB_008050	44.2591	0.0068	0
ALG7	AFUB_027000	48.0769	0.0069	0
TPS2	AFUB_043350	39.726	0.007	0
RPN6	AFUB_006680	41.8259	0.0071	0
POL30	AFUB_005270	48.4018	0.0073	0
YAF9	AFUB_066930	37.3016	0.0073	0
TPD3	AFUB_005980	45.4545	0.0074	0
MRE11	AFUB_077420	34.4388	0.0075	0
MOT1	AFUB_006220	43.7335	0.0076	0
MSH6	AFUB_065410	41.5499	0.0076	0
UBC6	AFUB_098390	37.9061	0.008	0
CRN1	AFUB_029890	40.813	0.0082	0
RPT2	AFUB_054610	62.7953	0.0082	0

VPS60	AFUB_068060	39.3519	0.0083	0.00929
GCS1	AFUB_054690	32.0388	0.0084	0
TUP1	AFUB_093130	35.8974	0.0084	0
ATP4	AFUB_082100	44.3983	0.0085	0
GCN3	AFUB_058910	41.4634	0.0085	0.00046
PRI2	AFUB_003820	39.2	0.0087	0
UBA4	AFUB_058070	37.3225	0.0087	0.00011
SLY1	AFUB_034910	36.7978	0.0089	0.00056
PSD1	AFUB_015290	34.3066	0.0091	0.0038
CEG1	AFUB_022810	33.012	0.0092	0
GYP7	AFUB_094350	30.5725	0.0092	6.8E-06
CDH1	AFUB_040820	35.8209	0.0093	0.00026
SEC17	AFUB_028490	41.4384	0.0093	0.01568
KAP95	AFUB_015250	37.6147	0.0098	0
PSE1	AFUB_007170	38.2648	0.0099	0
CNS1	AFUB_001140	31.3084	0.0101	0.01038
GCV3	AFUB_011540	30.2857	0.0104	0.00627
SLN1	AFUB_017740	30.7407	0.0105	0
VPS28	AFUB_015660	36.8852	0.0106	2.5E-05
DPH2	AFUB_073040	30.2655	0.0109	1.5E-05

Neurospora crassa									
**Initiation**					**Commitment**				
**S.cerevisae gene name**	**N. crassa gene stable ID**	**% of S.cerevisae gene identical to target N. crassa gene**	**dN/dS**	**MEGA P value**	**S.cerevisae gene name**	**N. crassa gene stable ID**	**% of S.cerevisae gene identical to target N. crassa gene**	**dN/dS**	**MEGA P value**
MNP1	NCU06469	48.8636	0.0182	2.8E-07	ADA2	NCU04459	37.7395	0.0167	0
DIP2	NCU03051	44.6058	0.0145	0	AIP1	NCU03944	36.4078	0.0105	0

NUC1	NCU00030	53.9157	0.0142	6.8E-10
DBP9	NCU07070	40.0888	0.0136	0.00499
BUD23	NCU00777	56.1151	0.0131	0.0291
DRS1	NCU11175	39.0832	0.0124	6.8E-06
MET13	NCU07690	49.9184	0.0121	1E-08
MEF2	NCU07021	30.9859	0.0118	4.2E-07
DBP3	NCU05782	44.2997	0.0111	0
RCL1	NCU02675	35.7143	0.011	0
UTP13	NCU03628	30.1518	0.0109	4.1E-05
HIS3	NCU01300	62.8821	0.0107	6E-09
NOC4	NCU00501	32.7239	0.0105	5.9E-07
MAK16	NCU04150	45.9877	0.0102	0
CCT5	NCU03980	67.0909	0.0101	0
DIA4	NCU09594	30.127	0.01	0.02322
DBP7	NCU06520	31.5725	0.0098	2E-08
UTP21	NCU07011	31.1383	0.0098	0
MRPS9	NCU03827	31.746	0.0096	5.8E-08
CCT4	NCU02839	66.4165	0.0096	0
BRX1	NCU01524	51.3846	0.0089	0
ERB1	NCU03321	47.7535	0.0089	0
NMD3	NCU08663	51.8304	0.0088	0
TMA22	NCU02984	39.0374	0.0087	0
UTP23	NCU02748	30	0.0086	0
MRPL8	NCU01023	33.6788	0.0085	2.5E-07
TSR1	NCU01503	36.5973	0.0085	0.0019
BNA3	NCU03347	48.8172	0.0084	0.00044
HGH1	NCU08324	42.2535	0.0084	1.2E-05
NOC2	NCU02066	33.924	0.008	0
MET12	NCU09545	42.1439	0.0075	0.00359
MRPS16	NCU08071	48.5981	0.0074	0

ATG1	NCU00188	31.9215	0.0099	0
ATP4	NCU00502	43.1535	0.0079	0
CBK1	NCU07296	46.3211	0.0066	0
CDC21	NCU10053	51.3587	0.0045	0
COX6	NCU06695	50	0.0055	0
DID4	NCU00435	52.8634	0.0052	0
DPH1	NCU08503	50.3268	0.0056	0
EMP24	NCU08339	47.2906	0.0057	0
GPN2	NCU09745	50	0.0063	0
HTA1	NCU02437	85.0746	0.0034	0
KAE1	NCU03836	65.8192	0.0132	0
KAP95	NCU02011	37.8995	0.0097	0
LST8	NCU04281	61.9497	0.004	0
MCM3	NCU08009	46.6102	0.0135	0
MET16	NCU02005	35.6784	0.0047	0
MNN10	NCU03055	35.5263	0.0056	0
POL30	NCU09239	45.5598	0.0063	0
PPH3	NCU08301	47.2813	0.0041	0
PRB1	NCU00673	46.9925	0.0059	0
PSF1	NCU02631	47.2222	0.0057	0
RAD6	NCU09731	78.1457	0.002	0
RFC1	NCU06767	32.965	0.0082	0
RHO3	NCU00600	65.2174	0.0044	0
RPT2	NCU01224	69.9346	0.0027	0
RSP5	NCU03947	64.3819	0.0041	0
RVS167	NCU04637	48.394	0.0162	0
SAH1	NCU07930	80.6236	0.0017	0
SEC26	NCU04404	49.3208	0.0167	0
SEC27	NCU07319	50.9324	0.0061	0
TAF6	NCU07757	37.4194	0.0099	0

TMA20	NCU08678	42.8571	0.0074	0.0046
GDH2	NCU00461	41.96	0.0073	0
RIO1	NCU08767	32.7808	0.0072	0.00059
POT1	NCU04796	49.642	0.0068	0
SDO1	NCU00476	37.7778	0.0068	0
SWS2	NCU09539	47.8992	0.0067	0
PRO2	NCU01412	51.9101	0.0066	0
SCH9	NCU03200	42.8951	0.0066	0
UTP7	NCU01502	43.5018	0.0066	0
MRPL23	NCU07852	41.5301	0.0065	0
BMS1	NCU04348	46.8966	0.0061	0.03162
FCY1	NCU07413	50.3145	0.0058	0
RPO31	NCU03986	51.8591	0.0055	0
RPC19	NCU00419	43.5897	0.0054	0
SPB1	NCU03669	50.4212	0.0053	0
TRP3	NCU00200	37.2703	0.0052	2E-08
UGA1	NCU08998	52.233	0.005	0
ALA1	NCU02566	49.6833	0.0048	0
ARG1	NCU02639	57.554	0.0046	0
KRE33	NCU02284	61.6761	0.0042	5.4E-07
ARO3	NCU09817	62.766	0.0041	0
RET1	NCU01772	62.5974	0.004	0
CCT7	NCU09700	67.325	0.0034	0
NSA2	NCU00981	67.0498	0.0034	0
CCT2	NCU02207	73.2076	0.0033	0
KRR1	NCU07041	65.9375	0.0032	0.00967
TIF6	NCU09004	76.4228	0.0031	0
CCT3	NCU01843	72.037	0.0028	0

TPS2	NCU05041	35.5665	0.0078	0
VMA11	NCU00667	64.6707	0.0046	0
VMA4	NCU07446	43.0435	0.0063	0
VMA8	NCU08035	66.2879	0.0036	0
VPS28	NCU11209	35.0211	0.0112	0
VPS55	NCU06713	46.0938	0.0101	0
VPS60	NCU04541	41.2844	0.0112	0
VTC4	NCU08110	50.8431	0.0053	0
CIN4	NCU00218	35.3591	0.0092	2E-08
MNS1	NCU02778	30.0995	0.017	2E-08
VMA16	NCU09747	64	0.0051	3E-08
VTI1	NCU05959	35.0877	0.0094	5E-08
GCV3	NCU08877	33.1361	0.0086	7E-08
PMR1	NCU03292	47.2195	0.0067	1.1E-07
RPN1	NCU07721	50	0.0061	1.2E-06
APC11	NCU11300	45.283	0.0067	1.1E-05
MCM7	NCU08119	50.9709	0.0055	1.4E-05
PFK2	NCU00629	51.2426	0.006	5.2E-05
CNB1	NCU03833	61.4943	0.0038	7.2E-05
SEC21	NCU01992	40.0655	0.0094	9.5E-05
MOT1	NCU07556	43.0005	0.0183	0.00011
SNF2	NCU06488	39.6086	0.0068	0.00011
PRI2	NCU04646	36.6472	0.0081	0.00073
SOD2	NCU09560	45.7031	0.0049	0.00161
SEC22	NCU06708	49.3088	0.0057	0.00192
SFT1	NCU03889	32.6531	0.0098	0.00223
MET30	NCU08563	32.0388	0.0077	0.01285
MCM5	NCU01171	60.9116	0.007	0.01304
NCP1	NCU09741	41.763	0.007	0.01325
AHA1	NCU04087	37.7301	0.0082	0.02695

SED5	NCU01907	35.9621	0.0097	0.02759
PSD1	NCU03695	36.8421	0.0078	0.03122
VMA5	NCU09897	33.7596	0.0108	0.03135
PRP22	NCU06318	45.8863	0.0066	0.04199
RPN6	NCU01596	46.2264	0.0066	0.02877
ESA1	NCU05218	46.6403	0.0049	0.02103
ACO2	NCU04280	57.2311	0.0051	0
ARL3	NCU00333	50.7042	0.0054	0
CDC28	NCU09778	63.7195	0.0065	0
GCN5	NCU10847	57.561	0.0043	0
HOS2	NCU02795	42.0842	0.0057	0
HTZ1	NCU05347	69.2308	0.0033	0
ICL1	NCU04230	57.2993	0.0053	0
LIP5	NCU00565	52.093	0.0055	0
QNS1	NCU04648	57.7503	0.005	0
RBG2	NCU01183	63.9785	0.0038	0
RPN5	NCU02650	43.5223	0.0064	0
SIT4	NCU03436	58.2474	0.003	0
TOA2	NCU09748	46.087	0.0055	0
VPS4	NCU06942	66.4399	0.0038	0
YAH1	NCU07794	49.2147	0.0046	0

Kluyveromyces lactis										
**Meiosis initiation**						**Meiosis commitment**				
**S.cerevisae gene name**	**K. lactis UniProt ID**	**% of S.cerevisae gene identical to target K. lactis**	**dN/dS**	**MEGA P value**		**S.cerevisae gene name**	**K. lactis UniProt ID**	**% of S.cerevisae gene identical to target K. lactis gene**	**dN/dS**	**MEGA P value**
RPS10A	Q6CVZ5	80.769	0.1892	1.2E-05		ACO2	Q6CUP0	82.161	0.0395	0

RPS10B	Q6CVZ5	78.846	0.1485	1E-07
NOP10	Q6CSZ0	82.456	0.1241	2.8E-05
HYP2	Q6CM83	87.261	0.1185	3E-08
TMA19	Q6CTH3	83.832	0.1071	0
ANB1	Q6CM83	87.898	0.105	0
ARO4	Q6CL12	84.046	0.104	0
EFT2	Q6CPQ9	92.874	0.1082	0
GLN1	Q874T6	87.363	0.1072	0
UGA1	Q6CJ86	77.119	0.0885	0
MRPL7	Q6CT55	73.498	0.0925	0
GRS1	Q6CVW3	80.543	0.0911	0
MSN2	Q6CIG0	82.258	0.0687	0.00012
RPA49	Q6CUR0	70.361	0.0667	0
LYS9	Q6CSQ4	83.973	0.0629	0
NCL1	Q6CK91	70.728	0.0614	0
SAM2	Q6CUW4	88.021	0.0588	0
GAR1	Q6CJ45	84.615	0.057	0
GUK1	Q6CWT3	80.749	0.0525	0
IMD4	Q6CWA8	80.843	0.0511	0
IMD3	Q6CWA8	80.115	0.05	0
MRPL39	Q6CWX0	75.714	0.0478	0
RPF2	Q6CXI6	73.295	0.0445	0
PMI40	Q6CQW4	76.056	0.0432	0
NMD3	Q6CXW2	82.157	0.043	0
IMD2	Q6CWA8	77.055	0.0378	0
TMA20	Q6CQX1	77.901	0.0363	0
DBP9	Q6CUI6	72.879	0.0349	0
ASN2	Q6CVM7	85.639	0.0337	0
GFA1	Q6CK47	82.287	0.0337	0
CGR1	Q6CNQ3	72.727	0.0316	0

ACS2	Q9Y7B5	84.559	0.0373	0
ACT1	P17128	97.333	0.0295	0
ADH1	P20369	85.591	0.1287	0
ALD4	Q6CLU0	76.099	0.0563	0
ARF1	Q6CL72	87.293	0.059	0
ARF2	Q6CL72	87.845	0.0332	0
ASF1	Q6CN69	90.385	0.0415	0
ATG8	Q6CMF8	85.345	0.0083	0
ATP17	B4UN98	74.747	0.0546	5.7E-05
CDC21	Q6CXJ0	81.25	0.0276	0
CDC34	Q6CRG7	81.132	0.0245	0
CDC42	Q6CY03	96.859	0.0073	0
CNB1	Q874T7	83.429	0.0018	0
COF1	Q6CQ22	85.315	0.0443	0
COX7	Q6CVX3	73.684	0.094	9.2E-07
DID2	Q6CJ52	76.585	0.0021	0
DID4	Q6CVB4	70.661	0.0185	0
DOT6	Q6CMI6	79.661	0.1044	6E-08
DYS1	Q6CNG7	80.952	0.0133	0
EMP24	Q6CK93	75.269	0.0776	0
ENO1	Q70CP7	88.33	0.1875	0
ENO2	Q70CP7	86.728	0.1913	0
ERP2	Q6CQT0	70.466	0.049	0
ERR3	Q70CP7	70.023	0.0235	0
ERV25	Q6CWW7	78.351	0.0812	0
ESA1	Q6CKE9	76.854	0.0142	0
FBP1	Q05079	76.149	0.0268	0
FRQ1	Q6CPT3	78.421	0.0173	0
GCN3	Q6CK30	78.105	0.0177	0
GLC7	Q6CK94	94.855	0.0121	0

TRP5	Q6CNQ8	78.784	0.0311	8.9E-07
CCT5	Q6CV90	82.609	0.031	0
ENP2	Q6CXY5	75.389	0.0308	0
DBP8	Q6CXW0	75.587	0.0307	0
LYS2	Q6CVV0	73.353	0.0301	0
MAK16	Q6CY11	70.684	0.0294	0
KRR1	Q6CV23	83.923	0.0269	0
UTP15	Q6CK39	74.038	0.0209	0
NIP7	Q6CWQ7	89.503	0.0184	0
TRM112	Q9P892	71.852	0.0181	0
ISU2	Q6CRQ9	81.884	0.018	0
CCT4	Q6CL82	85.361	0.0178	0
TIF6	Q6CSD0	94.694	0.0176	0
HAS1	Q6CXB7	88.353	0.0173	0
TMA22	Q6CJ30	72.131	0.0171	0
NSA2	Q6CNC5	93.103	0.0162	0
SOF1	Q6CJV8	78.17	0.0162	0
CCT8	Q6CRG0	79.789	0.0156	2E-08
PRO2	Q6CY14	74.561	0.0153	0
CCT7	Q6CJ51	87.636	0.013	0
AAC1	P49382	76.393	0.0127	0
RCL1	Q6CUC5	80.822	0.0116	0
RLM1	Q6CM05	73.958	0.0109	0
GUF1	Q6CUH2	73.52	0.0074	0
CCT2	Q6CTM8	87.643	0.0058	0
TRP3	Q6CV69	71.193	0.003	0.00016
SDO1	Q6CJW1	72.358	0.0025	0
RRP8	Q6CTL0	71.646	0.0022	0.00012
MRPL19	Q6CRK1	77.848	0.0017	0

GPN2	Q6CTW8	80.98	0.0152	0
GTR1	Q6CUA8	75.806	0.0278	0
GTR2	Q6CXY3	75.466	0.0049	0
HHF1	Q6CMU6	97.087	0.0285	4E-08
HOS2	Q6CKP4	79.596	0.0046	0
HTA1	Q6CK59	97.674	0.0385	0
HTA2	Q6CK59	97.674	0.027	0
HTB1	Q6CK60	94.697	0.0548	0
HTB2	Q6CMV8	93.182	0.0265	0
HTZ1	Q6CUC8	95.385	0.0083	0
ISW1	Q6CL05	76.058	0.0154	0
KAE1	Q6CJ48	84.197	0.0114	0
KIN28	Q8J0L2	78.114	0.0329	0
LST8	Q6CS14	86.139	0.0112	0
MCM5	Q6CRG4	72.165	0.0024	0
MET14	Q6CVB5	87.879	0.0159	0
MSN4	Q6CIG0	80.952	0.0622	1.2E-07
NCB2	Q6CU97	71.014	0.0034	0
NHP6B	Q6CVH3	80.46	0.0488	0
PFY1	Q6CPT8	81.746	0.0821	0
PIL1	Q6CKT9	87.372	0.0268	0
POL30	Q6CVH2	70.543	0.0357	0
PYC1	Q6CUD3	86.364	0.0305	0
QCR9	Q6CJX2	75	0.0496	0
QNS1	Q6CQZ6	81.373	0.0174	0
RAD6	Q6CUD9	97.987	0.0037	0
RHO2	Q6CTC2	79.058	0.0016	0
RHO3	Q6CLF3	77.253	0.0021	0
RNR1	Q6CU38	77.728	0.0578	0
RPN6	Q6CPX9	74.473	0.0385	0

RPT1	Q6CNB8	86.013	0.0208	0
RPT2	Q6CJZ8	90.16	0.0075	0
SEC22	Q6CJA0	72.43	0.0193	0
SIT4	Q9P4W5	92.605	0.0127	0
SNC1	Q6CY19	74.138	0.0094	0
SNC2	Q6CY19	82.609	0.0431	0
SNZ2	Q6CYH5	77.258	0.0224	0
SOD1	Q6CPE2	70.13	0.0381	0
SPT15	Q6CNT4	83.058	0.0301	0
SPT3	O13472	72.997	0.0178	0
SPT4	P81205	78.431	0.0368	0
SPT5	Q6CWW9	71.058	0.0303	0
STB1	Q6CP62	71.053	0.1072	0.00208
SUB2	Q6CM95	87.416	0.0403	0
TEM1	Q6CWD1	72.139	0.0033	0
TFB3	Q6CT73	74.455	0.0195	0
TPD3	Q6CKW1	71.287	0.0311	0
TRR2	Q6HA24	78.107	0.0426	0
UBC13	Q6CSV2	89.262	0.0012	0
UBC4	Q6CNH3	93.243	0.0248	0
UBC8	Q6CX52	73.81	0.0337	0
UBC9	Q6CPG7	82.166	0.0264	0
VMA16	Q6CNL9	79.245	0.0116	0
VMA2	Q6CRG9	96.099	0.0317	0
VMA6	Q6CWE4	78.098	0.0183	0
VMA7	Q6CS31	77.966	0.007	0
VMA8	Q6CYA1	81.159	0.0202	0
YEF3	Q6CKA7	85.92	0.1565	0
YPK1	Q6CVE2	73.852	0.0489	0
YPK2	Q6CVE2	70.957	0.004	0.0053

YPT31	Q6CWX9	77.13	0.0142	0
YPT32	Q6CWX9	79.279	0.0039	0
YRB1	Q6CMW3	72.222	0.0667	0
ZPR1	Q6CN09	73.469	0.0217	0

Schizosaccharomyces pombe									
**Initiation**					**Commitment**				
**S.cerevisae gene name**	**S. pombe gene name**	**% of S.cerevisae gene identical to target S. pombe**	**dN/dS**	**MEGA P value**	**S.cerevisae gene name**	**S. pombe gene name**	**% of S.cerevisae gene identical to target S. pombe gene**	**dN/dS**	**MEGA P value**
ALA1	ala1	59.1241	0.0081	0	TAF2	taf2	30.2901	0.0035	2.7E-05
ARG1	arg12	60.7317	0.0066	0	MTR10	mtr10	30.3665	0.0105	0
BRX1	brx1	50.5085	0.007	0.00915	APL5	apl5	30.4242	0.0096	0
CCT4	cct4	67.1727	0.0067	1E-06	SPT5	spt5	31.3131	0.0086	0
CCT8	cct8	55.4945	0.0067	0	GYP7	gyp7	31.8977	0.0044	0
DBP7	dbp7	35.9661	0.0114	0.00038	CDC45	cdc45	31.9749	0.0091	0.00047
DBP9	dbp9	48.9076	0.005	0	HYM1	pmo25	33.1307	0.0096	0.00961
DIP2	dip2	41.5401	0.007	2.1E-07	PFS2	pfs2	33.5953	0.0077	8E-08
ERB1	erb1	41.2162	0.013	0	ISC1	css1	33.7264	0.0094	8.5E-05
FUN12	SPAC56F8.03	46.7099	0.008	0	MPE1	mpe1	33.8174	0.0073	1.7E-06
GCV1	gcv1	44.9612	0.0068	0.00356	FCP1	fcp1	33.8866	0.0089	0.00248
GCV2	gcv2	50.3395	0.0051	0	ERV46	SPAC24B11.08c	34.359	0.0094	0.02776
GDH2	gdh2	37.7034	0.0077	0.04437	APL2	apl2	34.407	0.0092	0
GLN1	gln1	67.1309	0.0076	0	CIN4	alp41	34.4086	0.0089	0
GRX5	grx5	47.2603	0.0063	2.5E-06	AIP1	aip1	34.4538	0.0096	2.4E-06
GUF1	SPAC1B3.04c	47.546	0.0057	0.00075	VTI1	vti1	34.5794	0.0081	7E-06
HAS1	has1	64.5329	0.0052	0	CNS1	cns1	34.6369	0.0086	0
HGH1	hgh1	42.6966	0.0066	0.0016	MET30	pof1	34.7107	0.0079	0

KRE33	nat10	62.8267	0.0096	0
LYS2	lys1	49.1896	0.0058	0
LYS4	lys2	59.2233	0.0051	0
MAK16	mak16	52.3179	0.006	0
MET12	met11	42.2777	0.0072	0
MET13	met9	47.2637	0.0061	0
MRPL19	mrpl19	52.7778	0.0065	3E-06
MRPL8	mrpl8	35.7488	0.01	0
MRPS12	mrps12	45.0617	0.0069	3.9E-06
MRPS16	mrps16	50	0.0074	7E-08
MSS1	mss1	36.8952	0.0086	4.5E-05
NHP2	nhp2	64.9351	0.0084	0
NIP7	nip7	70.5556	0.0073	0.00023
NOC2	SPAC1142.04	33.8048	0.0084	1E-08
NOC4	noc4	34.6392	0.0096	0.04127
NOP58	nop58	56.2992	0.0054	5.4E-05
RCL1	rcl1	40.7714	0.0076	0.00217
RET1	rpc2	70.3863	0.0055	0
RML2	rml2	50.5525	0.0055	0.00542
RNH201	rnh201	36.1963	0.0082	1.8E-05
RPF1	SPAC4F8.04	43.7908	0.0053	0.00622
RPO31	rpc1	59.3594	0.0074	5.8E-07
RRB1	rrb1	40	0.007	0.00519
RRP3	rrp3	52.4731	0.007	0
RRS1	rrs1	49.3976	0.0052	0
SDO1	sdo1	46.748	0.0059	0
SPB1	spb1	48.8778	0.0109	0.02667
SPB4	SPBC24C6.02	34.9835	0.0081	0.00595
SPE2	spe2	34.9206	0.0092	0
TCP1	cct1	76.259	0.0062	0

RRP40	rrp40	35	0.0074	0.00468
NRK1	SPBP22H7.06	35.2174	0.0086	1E-05
TFB3	pmh1	35.2201	0.0088	2.5E-07
CDC5	plo1	35.2855	0.0078	0.01108
TFB4	tfb4	35.3535	0.0075	0.00022
NCP1	ccr1	35.8407	0.0096	0.00014
SED5	sed5	35.9223	0.0102	0.00034
ERV25	erv25	36.1111	0.0084	6.2E-05
MRE11	mre11	37.2881	0.0061	0.00033
SEC21	sec21	37.5691	0.0087	0.00229
VMA4	vma4	37.8855	0.0094	1E-08
ATP4	atp4	38.5246	0.0097	4.2E-07
RRD1	ypa1	38.7692	0.009	0
SKI6	rrp41	39.2562	0.0087	0
KAP95	kap95	39.2816	0.0085	0
MOT1	mot1	40.041	0.0068	0.00027
YIP1	SPCC61.04c	40.0881	0.008	1E-08
PMT4	ogm4	40.617	0.007	0
RAD16	rhp16	41.7445	0.005	0.00261
UFD1	ufd1	42.1053	0.0071	1E-08
TAF6	taf6	42.2566	0.0077	0
SMC2	cut14	42.4061	0.0067	0
GCN3	SPCC11E10.07c	42.8153	0.0066	4E-08
ADA2	ada2	43.2494	0.0064	0
SEC17	sec17	43.2526	0.0092	7.6E-05
POL30	pcn1	43.8462	0.0068	0
NCB2	ncb2	44.0994	0.0086	0.00011
PRI2	spp2	44.4444	0.0066	0.00373
TOP3	top3	45.3376	0.0068	0
CRN1	crn1	45.7571	0.0063	4.5E-05

TIF6	tif6	77.0492	0.0071	0
TMA20	tma20	56.3536	0.0067	0
TMA22	SPBC16C6.05	38.4211	0.0082	0
TRP5	trp2	61.9799	0.0058	0
TSR1	tsr1	38.9527	0.007	1.4E-06
UTP13	utp13	36.1647	0.0083	3E-08
UTP15	utp15	33.6032	0.0092	0
UTP25	utp25	35.5828	0.0122	0.00022
UTP7	utp7	46.3462	0.0058	0.0007
YIH1	yih1	37.5	0.0099	0.00155
YKE2	gim1	38.5965	0.0066	2.2E-05
YTM1	SPAC890.04c	42.0455	0.0078	2.3E-06

YAF9	yaf9	46.083	0.0064	0
SOD2	SPAC1486.01	46.3303	0.0071	0
MOB1	mob1	46.6667	0.0063	0.00034
RPN1	mts4	47.2503	0.0058	0
MCM3	mcm3	47.3265	0.0062	0.00196
SEC22	sec22	47.4419	0.0065	5E-08
KIN28	mcs6	47.7612	0.006	0
DID2	did2	48	0.0077	0
RPN6	rpn6	48.6936	0.0064	0
ALG7	gpt2	49.1031	0.0068	0
PFK2	pfk1	49.1507	0.006	0
VMA8	vma8	49.4737	0.0044	0.00905
ASF1	cia1	49.6183	0.005	0
DID4	did4	50	0.0048	0
VMA6	vma6	50.7289	0.0049	0.00143
MET16	met16	50.7519	0.0049	0
VTC4	vtc4	53.1207	0.0047	0
TOA2	toa2	53.211	0.0051	0.00049
PMR1	pmr1	53.7264	0.0055	3E-08
ARC18	arc3	54.5977	0.005	5.8E-06
HOS2	hos2	55.0691	0.0043	0.0016
TEM1	spg1	56.0606	0.0028	0.01012
VMA7	vma7	56.6667	0.0048	0.00076
UBC11	ubc11	57.3864	0.0039	0
PRS5	prs5	57.7713	0.0046	1.6E-06
YKT6	ykt6	58.8832	0.0045	0
VMA11	vma11	61.7284	0.0044	0
MET17	met17	64.3357	0.0042	0
LST8	pop3	64.6497	0.0032	0
KAE1	pgp2	67.6301	0.0036	0

RPT2	rpt2	70.7589	0.0023	0
SIT4	ppe1	72.1311	0.0106	0
SPT15	tbp1	74.8918	0.0028	0
RAD6	rhp6	76.8212	0.002	0
ACO2	aco2	53.9216	0.0043	0.00003
CKS1	suc1	67.2566	0.0043	0.00004
DMC1	dmc1	61.747	0.0045	5.6E-06
GCN5	gcn5	51.3216	0.0042	3.4E-08
HTZ1	pht1	65.4676	0.0033	4.4E-08
MCM5	mcm5	60.8333	0.0041	4.5E-10
MCM7	mcm7	58.6842	0.0042	2.5E-11
PPH3	pph3	54.023	0.0037	2E-07
RHO3	rho3	68.2927	0.0032	2.6E-08
RRD2	ypa2	48.0114	0.0052	2.5E-08

Schizosaccharomyces cryophilus

Meiosis Initiation

S.cerevisae gene name	S. cryophilus gene stable ID	% of S.cerevisae gene identical to target S. cryophilus gene	dN/dS	MEGA P value
RPC19	SPOG_00730	52.4194	0.00986	0.049666
TSR1	SPOG_04899	38.6598	0.00909	0.039235
YML6	SPOG_02844	31.3253	0.00908	0.028767
TRP5	SPOG_00313	62.6973	0.00905	0
YIH1	SPOG_02653	36.6548	0.0104	4.98E-06
IMP4	SPOG_01686	54.5139	0.0103	6.88E-05
MEF2	SPOG_01452	32.0823	0.01	0
MRPL7	SPOG_04684	33.564	0.0097	0
RPO31	SPOG_04330	58.9324	0.0096	0
ECM16	SPOG_04706	38.595	0.0095	0.000186
BNA3	SPOG_00293	47.1264	0.0094	0

Meiosis commitment

S.cerevisae gene name	S. cryophilus gene stable ID	% of S.cerevisae gene identical to target S. cryophilus gene	dN/dS	MEGA P value
APL2	SPOG_01331	34.6209	0.0088	0
ARC18	SPOG_02780	56.8966	0.0048	0
CBK1	SPOG_00685	63.2911	0.0035	0
CDC21	SPOG_04744	30.1262	0.0036	0
CRN1	SPOG_00677	43.1973	0.0063	0
DID2	SPOG_03090	49.7512	0.0071	0
DTD1	SPOG_04712	54	0.0056	0
ERV1	SPOG_03692	38.7978	0.008	0
HOS2	SPOG_03918	55.0691	0.0043	0
HTZ1	SPOG_04437	64.2857	0.0036	0
MET30	SPOG_00535	38.1818	0.0068	0

NOC4	SPOG_03162	35.6108	0.0094	0
SPE2	SPOG_02951	36.7454	0.0094	0.003012
UTP15	SPOG_01280	34.6154	0.0092	0.002019
MSS1	SPOG_00706	34.2975	0.0092	0.049666
BUD23	SPOG_00476	51.4925	0.0091	0
MRPL8	SPOG_03672	34.2995	0.0091	1E-08
RRP9	SPOG_00470	32.8794	0.0087	8E-08
PRO2	SPOG_00686	48.5651	0.0086	0
UTP7	SPOG_00495	46.7308	0.0086	0.003761
PMI40	SPOG_03019	45.7421	0.0085	0
RNH201	SPOG_00223	33.9564	0.0085	4.1E-07
MRX14	SPOG_03110	35.1852	0.0085	0.000395
ARG3	SPOG_00231	50.7788	0.0084	0
TRM11	SPOG_03022	33.1897	0.0083	7.98E-06
DBP7	SPOG_04154	36.7989	0.008	0
GUD1	SPOG_03389	35.8925	0.008	0.002197
DRS1	SPOG_00637	42.0168	0.0079	0
MRPS16	SPOG_01290	46.875	0.0078	0.000961
UTP21	SPOG_03385	38.1637	0.0078	0.00187
BMS1	SPOG_01446	48.8475	0.0077	2E-08
RRB1	SPOG_01433	40.249	0.0077	9.6E-07
UTP25	SPOG_03563	37.02	0.0077	6.53E-05
SPB4	SPOG_02210	35.0329	0.0076	1.31E-05
DIP2	SPOG_04347	40.1737	0.0073	0
YTM1	SPOG_01919	40.4348	0.0073	4.18E-05
GLN1	SPOG_04889	63.0556	0.0072	0
SDO1	SPOG_01772	45.935	0.0071	0
RPC25	SPOG_00045	44.8276	0.0062	0.000159
CCT2	SPOG_04082	67.1727	0.006	0.004466
LYS2	SPOG_04753	48.2055	0.0059	0
UGA1	SPOG_00961	47.6793	0.0059	1.44E-06
RPF1	SPOG_01771	45.4248	0.0058	1.03E-06
DBP3	SPOG_00797	47.0919	0.0057	0.0006

MPE1	SPOG_03048	31.6532	0.008	0
PRE7	SPOG_01094	55.3571	0.0045	0
PRP16	SPOG_01423	36.7536	0.0066	0
PSF2	SPOG_04318	39.3443	0.0083	0
RAD50	SPOG_02740	30.7393	0.0082	0
RAD54	SPOG_04728	52.9755	0.0044	0
RAD6	SPOG_01975	76.1589	0.002	0
RBG2	SPOG_01285	57.5758	0.0042	0
RPT1	SPOG_01828	77.8539	0.0022	0
RRP4	SPOG_04051	45.1515	0.0069	0
SEC22	SPOG_02854	45.1163	0.0067	0
SEC27	SPOG_01834	45.5346	0.0063	0
SMC2	SPOG_02952	43.2225	0.0066	0
SOD1	SPOG_00687	63.3987	0.0049	0
SPT15	SPOG_00351	75.3247	0.0029	0
TAF6	SPOG_03333	41.9565	0.0069	0
TOA2	SPOG_03497	51.3761	0.0054	0
VMA16	SPOG_00400	60.804	0.0052	0
VMA7	SPOG_04609	56.1983	0.0051	0
VMA8	SPOG_02355	51.0417	0.0038	0
VTC4	SPOG_00814	53.1381	0.0047	0
MET16	SPOG_00246	52.2556	0.0054	3E-08
TFB4	SPOG_04355	33.2215	0.0088	4E-08
MRE11	SPOG_01556	39.4172	0.0065	4.4E-07
BCY1	SPOG_04710	34.878	0.0083	5.4E-07
CNB1	SPOG_02325	58.6207	0.0043	9.7E-07
UBA4	SPOG_03949	38.6308	0.008	1.9E-06
TPD3	SPOG_03719	46.2712	0.0068	2.7E-06
ALG7	SPOG_02827	46.6667	0.007	2.8E-06
RDI1	SPOG_00554	41.1765	0.0074	3.5E-06
TFB3	SPOG_00744	34.1317	0.0085	3.7E-06
KAP95	SPOG_02081	38.8181	0.0083	6.6E-06
SKI6	SPOG_03914	33.3333	0.0082	8.4E-06

RPF2	SPOG_02692	50.3165	0.0056	0
TUF1	SPOG_04652	65	0.0055	0
DBP8	SPOG_01961	51.9565	0.0052	0
ARG1	SPOG_02277	58.2927	0.005	0
RRP3	SPOG_01740	52.8785	0.0049	0
FUN12	SPOG_00639	49.0138	0.0048	0
DBP9	SPOG_03575	49.7409	0.0047	0
MAK16	SPOG_00705	52.5084	0.0047	0
TMA20	SPOG_03102	56.3536	0.0047	0
NMD3	SPOG_03962	53.1746	0.0045	0
NOP56	SPOG_04190	59.3625	0.0042	0
SOF1	SPOG_04014	54.5872	0.0042	0
ALA1	SPOG_00178	54.1708	0.0041	0
TMA19	SPOG_02732	61.3095	0.0037	0
CCT5	SPOG_00583	64.652	0.0032	0.003606
NIP7	SPOG_03539	72.2222	0.0027	2.58E-05
RET1	SPOG_00229	68.927	0.0025	0
HAS1	SPOG_02570	62.9696	0.0024	0
TCP1	SPOG_04131	75.3597	0.0024	0
TIF6	SPOG_02330	77.8689	0.002	0

CDC5	SPOG_00185	34.9296	0.0085	9.4E-06
FCP1	SPOG_01784	32.5386	0.0084	1E-05
VMA4	SPOG_00931	38.326	0.0081	1.4E-05
ADA2	SPOG_03252	42.7252	0.0064	2.5E-05
TIM18	SPOG_04033	30.1887	0.0085	4.4E-05
RFA1	SPOG_01455	35.8185	0.0079	0.00011
RPT2	SPOG_04304	70.7589	0.0046	0.00013
SPT5	SPOG_00660	30.6334	0.0091	0.00015
AIP1	SPOG_00101	32.6599	0.0086	0.0002
QCR8	SPOG_01108	52.6882	0.0057	0.0003
PRS5	SPOG_04341	55.5556	0.0048	0.0004
POB3	SPOG_02758	46.4844	0.0055	0.00063
RAD16	SPOG_00031	44.1558	0.0049	0.00091
SPT16	SPOG_03052	44.6516	0.0057	0.00098
SKI2	SPOG_00864	42.8336	0.0065	0.00123
CEG1	SPOG_04689	33.4951	0.0076	0.00129
POL30	SPOG_03030	44.0613	0.0068	0.00324
ISC1	SPOG_02308	32.2275	0.0087	0.00697
NRK1	SPOG_04410	33.4783	0.0086	0.00716
UBC7	SPOG_04119	60.241	0.0043	0.00757
PMR1	SPOG_01442	54.5556	0.0055	0.00809
UBX5	SPOG_00398	32.9545	0.0084	0.01042
GCN3	SPOG_02477	43.2353	0.0069	0.01768
TAF2	SPOG_04960	30.1997	0.0089	0.01834
MOB1	SPOG_02287	45.0237	0.0068	0.01874
CDC45	SPOG_01078	32.81	0.009	0.01908
SRV2	SPOG_03460	32.6886	0.0084	0.02025
ERV46	SPOG_02003	33.924	0.0083	0.02197
TAF1	SPOG_03945	30.2781	0.0077	0.02219
SEC17	SPOG_00494	40.8304	0.0074	0.02649
ERV29	SPOG_03414	36.014	0.0077	0.04534
LST8	SPOG_04864	63.0573	0.0034	0.02025
HOG1	SPOG_02005	80.8023	0.0018	0.01768

MCM5	SPOG_03770	60.0835	0.004	0.00098
MCM7	SPOG_03073	56.6838	0.0043	4.4E-05
VMA11	SPOG_00150	60.4938	0.0052	0

Schizosaccharomyces octosporus									
**Meiosis initiation**					**Meiosis commitment**				
**S.cerevisae gene name**	**S. octosporus gene stable ID**	**% of S.cerevisae gene identical to target S. octosporus gene**	**dN/dS**	**MEGA P value**	**S.cerevisae gene name**	**S. octosporus gene stable ID**	**% of S.cerevisae gene identical to target S. octosporus gene**	**dN/dS**	**MEGA P value**
CCT4	SOCG_02655	65.4649	0.014	0	LAS17	SOCG_00703	32.0151	0.012	0
YIH1	SOCG_03049	35.5872	0.0129	0	UBC11	SOCG_04035	57.3034	0.0107	0
MET12	SOCG_02416	42.5	0.0126	2E-08	CNB1	SOCG_02730	59.1954	0.0106	0
RRP8	SOCG_02182	41.0256	0.0126	7E-08	VPS55	SOCG_01562	38.2812	0.0103	6.6E-06
MRPL8	SOCG_04587	32.6923	0.0124	0.0001	MET17	SOCG_02692	51.4768	0.0098	7E-07
SPE2	SOCG_03337	35.958	0.0121	0.01144	PRI2	SOCG_00106	42.6087	0.0098	0
UTP15	SOCG_01767	33.6032	0.0109	2.3E-06	SOH1	SOCG_03833	30.7087	0.0093	0
MSS1	SOCG_02119	34.9174	0.0103	0	ISC1	SOCG_02715	31.7536	0.0091	0.00517
RNH201	SOCG_04317	34.2679	0.0101	0	AIP1	SOCG_04687	32.3232	0.0089	6E-06
DBP7	SOCG_03584	36.5887	0.0101	0.0144	AHA1	SOCG_02570	33.3333	0.0082	0.00371
UTP25	SOCG_03675	36.0681	0.01	4.8E-07	CEG1	SOCG_03203	33.8272	0.0079	1.6E-07
UTP21	SOCG_03852	38.9381	0.0096	3.3E-05	NRK1	SOCG_00066	32.6087	0.0079	0.00169
TRM11	SOCG_01282	32.5328	0.0096	2E-06	ERV46	SOCG_00502	34.6835	0.0077	0.00354
SPB4	SOCG_03266	34.4828	0.0095	0.00039	GYP7	SOCG_00898	31.1231	0.0077	0.02284
RRB1	SOCG_02569	38.8119	0.0094	0.00893	SPT5	SOCG_02165	31.3354	0.0076	6.6E-05
YTM1	SOCG_00586	43.1507	0.0094	0.04949	QCR8	SOCG_00918	51.6129	0.0074	0.00788
MRPS16	SOCG_01758	48.9583	0.0093	1.3E-05	ERV25	SOCG_02390	34.4186	0.0072	0.00013
GLN1	SOCG_02387	62.5	0.0093	1E-08	UBX5	SOCG_04144	34.0961	0.0071	0.00403
SDO1	SOCG_00725	45.5285	0.0092	0	PSE1	SOCG_02746	37.0776	0.0069	0.00022

ECM16	SOCG_03185	38.0165	0.0091	0
RPO31	SOCG_03410	59.4306	0.0091	0
PMI40	SOCG_01279	46.2287	0.009	0.00135
MRPL19	SOCG_01601	51.3889	0.009	0.00815
ARG3	SOCG_04309	51.0903	0.009	0
NUC1	SOCG_04219	45.3125	0.009	0
UTP7	SOCG_04816	46.7308	0.0089	0.01196
GUF1	SOCG_00620	49.3865	0.0089	0
RPF2	SOCG_03087	50.7937	0.0089	1.3E-07
BMS1	SOCG_02556	48.7979	0.0089	0.02569
DBP8	SOCG_00544	52.8509	0.0088	0
IMP4	SOCG_00811	53.8194	0.0087	0
ARG1	SOCG_02681	58.2927	0.0087	0
MAK16	SOCG_02120	53.0201	0.0086	0
NOP58	SOCG_02078	55.4241	0.0086	0
TMA20	SOCG_01359	56.3536	0.0084	0
DBP9	SOCG_03663	50.4318	0.0082	0.00387
RRP3	SOCG_00757	53.9957	0.008	9E-08
TRP5	SOCG_04228	61.406	0.0079	0
SOF1	SOCG_01998	53.8991	0.0079	0.04308
LYS4	SOCG_02422	60.8128	0.0076	0
NOP56	SOCG_03548	59.2445	0.0076	0
ALA1	SOCG_04361	55.054	0.0076	3.5E-07
CCT2	SOCG_01930	66.6034	0.0076	0.00037
TMA19	SOCG_03127	62.5	0.0075	0
TCP1	SOCG_03605	74.6403	0.0073	0
CCT5	SOCG_02242	65.2015	0.0072	0
CCT3	SOCG_01997	68.75	0.0071	0
CCT7	SOCG_00132	66.4286	0.0069	0
NSA2	SOCG_02720	68.0769	0.0069	0

CDC45	SOCG_00948	32.9154	0.0068	0.00166
SPT3	SOCG_04012	47.351	0.0063	0.01157
APL2	SOCG_04441	35.4412	0.0063	0
VPS60	SOCG_02910	34.1232	0.0063	0
SLY1	SOCG_02765	36.6352	0.0061	0.00015
NCP1	SOCG_04720	37.7581	0.0061	0.00017
CNS1	SOCG_00417	32.2034	0.0058	0
VMA4	SOCG_01087	40.0881	0.0055	0
TAF2	SOCG_02311	31.2287	0.0054	8E-07
TFB4	SOCG_03384	32.8859	0.0052	0.0007
CDC73	SOCG_02579	30.563	0.0051	0.01285
SKI6	SOCG_01619	39.834	0.005	0
SBA1	SOCG_04207	34.9206	0.0049	0.0056
MOT1	SOCG_03306	40.1124	0.0047	0
BCY1	SOCG_03180	34.2926	0.0047	0.00392
KAP95	SOCG_00422	39.0498	0.0047	0
CDC5	SOCG_04354	36.6864	0.0046	0.00166
MPE1	SOCG_01307	32.3944	0.0045	1.9E-05
MSH6	SOCG_02938	35.3265	0.0043	0
RFA1	SOCG_02547	35.8185	0.0041	7.6E-05
ERV1	SOCG_04565	38.0435	0.004	9.4E-06
UBA4	SOCG_02063	39.8496	0.004	2.7E-05
RDI1	SOCG_02270	41.3793	0.004	0.01954
PSF1	SOCG_01986	35.468	0.0037	0.03173
ALG7	SOCG_00242	46.4444	0.0035	7.7E-07
SOD2	SOCG_01608	44.0909	0.0034	0
SEC17	SOCG_04817	41.1765	0.0032	0
PMT4	SOCG_00667	39.8726	0.0031	3.3E-05
MRE11	SOCG_02445	39.6024	0.0031	0
MET30	SOCG_02289	37.4545	0.0031	2.3E-06

NIP7	SOCG_03696	71.6667	0.0068	3.8E-05
HAS1	SOCG_02968	64.2735	0.0068	0
TIF6	SOCG_02735	77.8689	0.0067	0

RRP4	SOCG_01961	45.6193	0.0025	0
TPD3	SOCG_04538	46.4407	0.0022	2E-06
MAS2	SOCG_02507	41.9223	0.0021	0
RRD2	SOCG_00916	44.6023	0.0067	0
RVS167	SOCG_00339	43.8636	0.0067	0
TAF6	SOCG_03903	42.2319	0.0067	0
POL30	SOCG_01289	44.0613	0.0067	0.00572
SEC22	SOCG_00268	46.5116	0.0066	0
SMC2	SOCG_03338	43.5635	0.0066	0
DID2	SOCG_01347	48.5149	0.0065	0
RPN6	SOCG_02086	47.981	0.0064	4.9E-06
RFC5	SOCG_00026	44.6927	0.0063	0.00043
SEC27	SOCG_00663	46.9182	0.0062	0
GPN2	SOCG_00951	48.4375	0.0059	0.02801
SPT16	SOCG_01311	44.2478	0.0058	0
PMR1	SOCG_02560	54.2222	0.0057	0
POB3	SOCG_00172	44.1905	0.0055	9.3E-05
TOA2	SOCG_03741	52.2936	0.0054	0
VMA16	SOCG_04142	61.3065	0.0052	0
YPT7	SOCG_00386	50.7246	0.005	0
ARC18	SOCG_00195	56.8966	0.0049	0
CDC21	SOCG_03147	30.6122	0.0049	0
VTC4	SOCG_01219	52.507	0.0049	0
PRS5	SOCG_03399	56.4327	0.0049	0.00011
VMA7	SOCG_01469	57.8512	0.0046	0
SOD1	SOCG_02139	62.5806	0.0045	0
RAD54	SOCG_03162	54.0445	0.0044	1E-08
FBP1	SOCG_01117	59.366	0.0043	0
VMA8	SOCG_02758	50.519	0.0043	0
MCM5	SOCG_04486	59.8053	0.0042	0

HOS2	SOCG_01615	54.8387	0.0042	3.7E-07
RBG2	SOCG_01763	57.5758	0.0042	4.1E-06
HTZ1	SOCG_00040	52.6316	0.0036	0
HUB1	SOCG_02559	61.6438	0.0036	6.9E-05
SPT15	SOCG_04191	75.431	0.0029	0
RPT1	SOCG_00669	75.5011	0.0021	0
RAD6	SOCG_00530	76.1589	0.002	0

Candida albicans

Meiosis initaition

S.cerevisae gene name	C. albicans gene stable ID	% of S.cerevisae gene identical to target C. albicans gene	dN/dS	MEGA P value
RPC25	C1_05230W_A	54.8718	0.0888	2.2E-05
UTP6	C1_10880W_A	38.5151	0.0849	5.2E-06
UGA1	C2_04190C_A	64.9682	0.0803	0
TRM10	C2_06480W_A	36.8421	0.0741	0.01944
ARO3	C2_02030W_A	68.7332	0.0683	0
NHP2	CR_04360C_A	71.0692	0.0657	0
UTP15	C2_08670C_A	45.3901	0.058	0.00077
GNA1	C2_03870W_A	46.9799	0.0505	3.7E-07
NMD3	CR_06720W_A	72.6923	0.0498	0
NIT2	CR_08840C_A	47.8261	0.0492	3.7E-05
NUC1	C5_00280C_A	59.0625	0.0478	0
MAK21	C3_04560W_A	47.4672	0.0474	0
DBP9	C6_01890C_A	62.7178	0.0458	0.02264
BFR2	CR_10470C_A	37.8531	0.0452	1.1E-06
FUN12	C1_08090C_A	67.5516	0.0448	0.00011

Meiosis commitment

S.cerevisae gene name	C. albicans gene stable ID	% of S.cerevisae gene identical to target C. albicans gene	dN/dS	MEGA P value
UBC12	C3_00520W_A	41.7526	0.0884	0
LSB6	C1_05980W_A	32.1839	0.0785	0
COF1	C5_00370W_A	80.8511	0.0769	0
RRP40	C1_01160C_A	42.963	0.0768	0.00061
COX9	C2_05930W_A	42.623	0.0757	0.00935
SRV2	CR_04190W_A	42.7523	0.0747	0
PSE1	C1_08110W_A	50.0458	0.0736	1E-07
BNA5	C1_08490W_A	55.9653	0.0724	0
RVS167	C6_04040C_A	59.7727	0.0696	0
NPR2	C3_03290C_A	31.6667	0.0676	1.3E-05
SIN3	C1_00930C_A	42.3104	0.0614	0.00273
SAH1	C5_04270C_A	83.3333	0.0604	0
CDC5	C1_00950C_A	53.5988	0.0591	0
HCR1	CR_10370W_A	48.7719	0.0591	0
MET17	C4_00200C_A	70	0.0585	0

TMA20	C4_06170C_A	65.7609	0.0444	0
RRP12	C7_01030C_A	45.1109	0.0434	0
LYS2	C1_02820W_A	59.8291	0.0427	0
GRS1	C1_05290W_A	70.8589	0.0424	0
IFM1	C7_02940C_A	38.2008	0.0418	0
TSR1	CR_08490W_A	43.4679	0.0406	0
FCY1	C6_00620W_A	58.6667	0.0392	0
HAS1	C5_04750C_A	68.8496	0.0371	0
RPC19	CR_02520W_A	55.7252	0.037	0
RPF1	C4_03270W_A	59.7973	0.037	0.00025
EBP2	C2_04570W_A	52.6932	0.0365	0.00093
CYS3	CR_08340W_A	68.6717	0.0364	0.02686
SAS10	C4_02790C_A	45.7447	0.036	0.03689
GDH2	C2_07900W_A	48.1061	0.0347	0.00501
NOP7	C2_09320C_A	60.1363	0.0326	0
BRX1	C1_12310C_A	69.5205	0.0324	0
ARG3	C6_03230W_A	60.5797	0.0315	0
ARG1	CR_00620C_A	73.7981	0.0297	0
ERB1	C1_04130W_A	64.0754	0.0297	8.6E-06
GUK1	C5_03790W_A	72.6316	0.0292	0
TRP5	C4_06110C_A	75.9259	0.0284	1.4E-07
RKI1	C3_01480C_A	67.0833	0.0252	0
TUF1	C1_00590W_A	76.7606	0.0247	0
SPB1	C6_04160C_A	63.5503	0.023	0
YTM1	C1_09510W_A	60.515	0.0219	0
TIF6	CR_07080W_A	92.2449	0.0209	0
GCD1	CR_03990C_A	34.5833	0.0197	0.01626
TMA19	CR_00860C_A	73.6527	0.0195	0
MPP10	C2_00070C_A	40.0353	0.0164	0
NOP58	C6_00370C_A	75.7752	0.0164	0

SMC1	CR_03680C_A	37.3387	0.0567	0
HTZ1	C3_03280C_A	81.9549	0.0547	0
UFD1	C2_02730W_A	54.8209	0.0492	0
SPC3	C1_13020C_A	41.1458	0.0489	0.00321
MCM3	C2_07350W_A	55.5809	0.0472	0.03656
DPH1	C2_05840W_A	67.8404	0.0465	0
APL4	CR_02870W_A	35.1449	0.0458	0
CCA1	C4_00880W_A	38.9831	0.0447	0
CTA1	C1_06810W_A	66.3918	0.0447	0
SPT5	C2_01480W_A	36.9247	0.0442	1.6E-07
PFK1	C5_04810W_A	54.8126	0.0436	0
PPG1	C4_05050C_A	45.4327	0.0433	0
YKT6	C1_02860C_A	71.5	0.043	0
RAD54	C1_13660W_A	59.7877	0.0421	0
HOG1	C2_03330C_A	79.0451	0.0412	0
VMA7	C2_04150C_A	71.1864	0.0397	0
UBX5	C1_07390W_A	35.7143	0.0384	0
SUB2	C4_00220C_A	79.4457	0.0381	0
SEC26	CR_04380C_A	55.1471	0.038	0
MCM7	C2_09020W_A	60.4353	0.0378	0
PSF1	C7_01300C_A	45.7627	0.0358	0
MET16	C4_07030W_A	58.7302	0.0352	0.00449
ICL1	C1_04500W_A	66.9091	0.0342	6E-08
GDB1	C4_05140C_A	56.0576	0.0335	0
ERV1	CR_03010C_A	50.5494	0.0317	0
ATP17	CR_00250W_A	58.8235	0.0303	5.1E-06
ATP18	C2_00610C_A	46.875	0.0274	8.9E-06
PRE7	C4_02470C_A	75.9184	0.0273	0.02773
MET14	C5_00430W_A	71	0.0271	0
ACS2	C1_04290C_A	67.7515	0.027	0

TCP1	C1_08560W_A	81.2274	0.016	0
NOP14	C3_04900W_A	36.5796	0.0152	0
KRR1	C1_11420W_A	71.229	0.0126	0
KRE33	CR_04240C_A	74.7601	0.0119	0
NOC2	CR_05520W_A	53.6415	0.0119	0
TAD2	C2_01510C_A	32.2222	0.0117	0
NOC4	C2_07340W_A	37.9004	0.0106	0.00062
EHD3	C1_03320C_A	35.4582	0.0097	0.00022
ZWF1	C1_08980C_A	61.5385	0.0089	0
BUD23	C5_01060C_A	71.5867	0.0086	0
MRP10	C5_03290C_A	46.3158	0.0086	4.5E-07
DBP6	CR_07750C_A	37.1287	0.0081	0
NOP16	C2_09660W_A	52.7523	0.0076	0
MTG1	CR_03380W_A	34.2391	0.0073	0.00011
MRPS17	C4_00660W_A	36.9919	0.0072	0
FCF2	C3_07800C_A	38.6473	0.0068	0
MSS1	C2_00820W_A	41.6834	0.0068	0
UTP20	C3_01200W_A	37.88	0.0067	4E-08
KTI12	CR_03370C_A	45.1389	0.0067	0.01469
YML6	C7_00950W_A	40.6475	0.0066	0.0116
LTV1	CR_10650W_A	48.0418	0.0059	2E-08
PCM1	C1_13760W_A	50.5515	0.0058	0
GUD1	C7_00870W_A	32.459	0.0057	0
MET12	C2_10460C_A	48.9062	0.0057	1E-08
NOP9	C1_04040C_A	48.4722	0.0049	0
RRP9	CR_02710W_A	45.7195	0.0048	2.6E-05
MRPS12	C1_06070W_A	46.1111	0.0048	0.01421
UTP21	C2_02430W_A	55.7325	0.0047	1.8E-05
TRM11	C3_05140C_A	50.4237	0.0045	0
MRPL16	C2_01030W_A	54.4601	0.0045	0.00114

CDC45	CR_07720C_A	37.8238	0.0264	0
TOP3	C3_04740C_A	48.1717	0.0261	0.00014
SEC17	CR_01350C_A	44.3686	0.0258	7.4E-06
DID4	C5_00440C_A	59.1489	0.0253	0.00065
ASF1	CR_07860C_A	68.4211	0.0247	0
RAD6	C7_03870W_A	73.743	0.0241	0
RBG2	C5_04900C_A	70.1897	0.0222	2.9E-06
MOB1	C6_02590C_A	47.2527	0.0221	0
SEC22	CR_03970C_A	54.6296	0.0217	0
VPS4	C5_03090W_A	75.3986	0.0211	0
ATG18	C1_00430W_A	35.4839	0.0199	0.03223
CDC28	CR_06050W_A	73.1861	0.0192	0
CDC34	C7_01920W_A	61.0656	0.0191	0
TPD3	C3_06910C_A	53.0327	0.019	0
CDC42	C1_08450C_A	87.4346	0.0183	0
FRQ1	C1_08680C_A	54.9738	0.0179	0
ACO2	CR_05790C_A	76.359	0.016	0
CDC73	C1_02920W_A	34.0541	0.0158	0
RPT2	C3_00290W_A	81.8594	0.0157	0
SEC31	C1_06930W_A	31.0672	0.0142	1.8E-05
SOD1	C4_02320C_A	68.8312	0.0129	0
EPL1	CR_00100C_A	33.8645	0.0127	0.00013
IST1	C3_00540C_A	40.3162	0.0126	0
KIN28	C1_06710W_A	62.0991	0.0118	0
AIP1	CR_03520C_A	34.7471	0.0109	3.6E-05
MOT1	C2_04500W_A	51.3838	0.0104	1.4E-06
FCP1	C3_07460W_A	35.6771	0.0103	0
ARC18	C6_01140C_A	57.6923	0.0101	0
RAD4	C3_07660W_A	30.1834	0.0098	4.8E-05
DNA2	C6_00320C_A	30.2945	0.0094	0

ARO1	C4_00890W_A	58.9942	0.0044	0
UTP11	CR_03360W_A	55.8704	0.0044	3E-08
DIP2	C1_08290C_A	52.7013	0.0044	6E-07
NOP4	C1_04390C_A	49.5277	0.0043	0
SCH9	C2_03940C_A	52.0966	0.0042	0
SDO1	C4_02880C_A	55.6863	0.0041	0
HGH1	C4_02050W_A	57.1823	0.0038	0
DBP8	CR_05630W_A	65.2273	0.0034	0
IMP4	CR_07950W_A	66.0777	0.0033	0
RCL1	C2_07450C_A	69.5418	0.0033	0
RRB1	C1_12680W_A	62.2824	0.0032	0.0039
ENP2	C7_03540C_A	56.4	0.0031	0
RPA12	C2_07300C_A	73.1707	0.0025	0
CCT5	C2_07310W_A	77.5547	0.0021	0
CCT2	C2_09520C_A	80.038	0.0021	1E-08
RPO31	C4_07060W_A	76.3812	0.002	1E-08
FCF1	C5_03920C_A	76.6169	0.0013	0

MSI1	C2_05510C_A	32.8736	0.0093	3.1E-05
MUS81	C6_00540W_A	32.2476	0.0093	0.00098
MNL1	C2_03910C_A	30.1663	0.0091	0.00455
SMC5	CR_03100W_A	30.8481	0.009	0
PPH3	CR_03800C_A	68.6274	0.009	1E-08
TEM1	C1_03080C_A	56	0.0089	0
KIP2	C2_10310C_A	31.5789	0.0086	5.7E-06
CDC37	C6_02610C_A	36.4173	0.0086	3.4E-05
VPS28	C2_08940C_A	33.4677	0.0086	0.037
ELC1	C1_04550W_A	47	0.0081	3.1E-05
TAF2	C6_04500C_A	30.807	0.008	5.9E-06
YPT7	C2_06910W_A	61.7512	0.0079	0
ESS1	C1_04410C_A	42.3729	0.0077	0
IML1	C1_02610W_A	34.3857	0.0077	0
CCS1	C1_07180W_A	43.5484	0.0077	0.00452
RRD1	C3_07080W_A	34.9057	0.0076	0
RKM4	C5_03250W_A	32.4275	0.0075	0
RET2	C5_02300C_A	38.2199	0.0074	0.01886
CFT1	C4_02430W_A	31.0563	0.0073	0
SAP1	CR_09860W_A	38.5366	0.0073	0.02215
PIF1	CR_07360W_A	30.6843	0.0072	0
VMA11	C7_01820C_A	81.5951	0.0072	0
TFG2	C2_00220C_A	38.0577	0.0072	4.9E-06
ISC1	CR_05830C_A	48.6301	0.0071	0
VPS55	CR_03130W_A	46.5278	0.0071	0
GYP7	C7_03490W_A	36.5979	0.0071	0.00058
PUT3	C1_07020C_A	35.8896	0.0071	0.00088
SKN7	C5_00240W_A	33.6315	0.007	0
NUP57	CR_02610C_A	32.2917	0.0069	0.00361
HAT1	C1_04670W_A	38.9831	0.0068	0

SEA4	C3_04460W_A	37.8134	0.0068	0
YNG2	C2_03430W_A	36.5772	0.0068	1.5E-05
BZZ1	C3_01500C_A	38.7097	0.0067	0
PEX10	C4_00320C_A	41.7219	0.0067	0
END3	C3_01400W_A	30.8861	0.0067	0.00054
TLG2	C2_02210C_A	30.3318	0.0067	0.00477
GPA2	C3_02240C_A	38.5686	0.0067	0.01705
ARG82	C2_09730C_A	38.8571	0.0066	0
UBA2	C1_08020W_A	42.6282	0.0066	0
STE23	C6_02860W_A	38.7187	0.0066	0.00033
CCP1	C3_02480C_A	44.8087	0.0066	0.0024
SLN1	CR_01000C_A	33.9403	0.0064	0
MSH6	C1_13170C_A	47.3641	0.0064	0.00318
CIN4	C1_02380C_A	40.5	0.0063	0
RAD14	C7_02020W_A	34.0909	0.0063	0
SED5	C1_00660C_A	45.6973	0.0063	0
YTA7	C5_04640C_A	39.1172	0.0061	2.2E-06
ATG7	CR_06730W_A	42.8795	0.006	0
IRC5	C3_01310W_A	35.3009	0.006	0
SMC3	C3_02700W_A	38.561	0.0059	0
URM1	C1_11160C_A	54.4554	0.0058	0
TAF6	C3_06650C_A	42.0039	0.0057	0
APC11	CR_10610C_A	43.609	0.0057	7.7E-07
VMA9	C1_10750C_A	37.2093	0.0056	6.6E-06
STV1	C6_00300C_A	43.7964	0.0056	0.01825
MET30	C1_01130W_A	41.2245	0.0055	0
SLY1	C4_06810C_A	50.7812	0.0055	2.4E-06
SKI2	CR_08570W_A	50.6827	0.0054	2.3E-06
CRN1	C7_01850C_A	50.5529	0.0053	0
MAD2	C1_04080W_A	41.1215	0.0052	0

PMT4	C2_06100W_A	52.9801	0.0052	0
TAZ1	C2_09350W_A	51.7073	0.0052	0
KAP95	C1_02240W_A	49.9429	0.0052	9.4E-06
VMA4	CR_01970C_A	57.5221	0.0051	0
UBC8	C1_01830C_A	53.1579	0.005	0
MAS2	C5_01640W_A	46.7433	0.0049	0.00242
MRE11	C7_01340W_A	41.9355	0.0048	0
PRI2	C4_06490C_A	46.593	0.0048	7.5E-07
EMP24	CR_07590W_A	57.5	0.0047	0
CNS1	C1_00560W_A	51.2821	0.0046	3E-08
ADA2	C1_10860C_A	53.2584	0.0044	8.9E-05
RSR1	CR_02140W_A	54.8387	0.0043	0
DID2	C5_02830W_A	40.7534	0.0042	0
RVS161	C7_00020C_A	70.4545	0.0042	0.00365
FBP1	C3_07830W_A	64.9547	0.0041	0
SMC2	C2_08560W_A	53.2024	0.004	0
VPH1	C4_05240C_A	57.1255	0.004	0
APS2	C1_11800C_A	61.3793	0.004	2.1E-05
TOA2	C4_01680W_A	52.3077	0.0039	0
CNB1	C5_05160C_A	66.474	0.0037	0
GPN2	C5_02020C_A	63.0682	0.0037	0
RHO2	C2_07750W_A	64.1711	0.0036	0
ESA1	C3_00490W_A	53.0499	0.0035	0
RRP4	C4_03740W_A	60.1974	0.0035	0
SKI6	C4_02090C_A	62.5	0.0034	0
RAD16	C1_02810W_A	60.2113	0.0033	0
HOS2	C3_00780W_A	65.1982	0.0032	0
HUB1	C4_05040W_A	61.6438	0.003	0
MCM5	C2_06250C_A	67.5824	0.003	0
GPA1	C5_05220W_A	65.7343	0.0029	0

QNS1	C2_01530C_A	69.4678	0.0029	0.01155
GTR1	C2_08600W_A	59.0909	0.0028	0
HAP3	CR_04290W_A	76.1905	0.0027	0
DMC1	C1_12560C_A	73.4568	0.0026	0
PRS3	C2_02510W_A	86.875	0.0022	0
UBC13	C5_00560W_A	69.2308	0.0021	0
SIT4	C1_04380W_A	82.8026	0.0015	0
GLC7	CR_07650W_A	84.8485	0.001	0

Candida tropicalis

Meiosis Initiation

S.cerevisae gene name	C. tropicalis gene stable ID	% of S.cerevisae gene identical to target C. tropicalis gene	dN/dS	MEGA P value
MRP2	CTRG_06135	47.8261	0.1079	8.5E-07
UTP6	CTRG_04074	38.1279	0.0994	0
UGA1	CTRG_01571	63.6943	0.0855	0
PMI40	CTRG_01997	60	0.0839	6.06E-05
SAD1	CTRG_03720	33.5	0.0778	0.000122
GRS1	CTRG_04512	72.3926	0.0752	0
UTP15	CTRG_01866	41.7453	0.0737	0.000934
PUF6	CTRG_01658	47.8519	0.0688	0.000354
GDH2	CTRG_01673	47.7725	0.067	9.9E-07
MRP10	CTRG_02704	46.875	0.0636	8.62E-05
LYS9	CTRG_05710	70.4955	0.0612	1E-08
PRO2	CTRG_05627	61.0478	0.0583	0
BRX1	CTRG_03515	68.8581	0.0579	0
TRM11	CTRG_02524	53.6866	0.0558	0
TSR1	CTRG_03084	51.8277	0.0551	0.001546
RPF1	CTRG_00510	59.322	0.0547	0
MAK21	CTRG_02297	47.7833	0.0547	1.57E-05

Meiosis commitment

S.cerevisae gene name	C. tropicalis gene stable ID	% of S.cerevisae gene identical to target C. tropicalis gene	dN/dS	MEGA P value
COR1	CTRG_06129	41.3636	0.0992	1E-08
MET17	CTRG_00016	70.8428	0.0949	0
SAH1	CTRG_06049	83.1111	0.0932	0
SKN7	CTRG_05246	31.3458	0.0899	0.00297
BRN1	CTRG_03850	31.7073	0.0837	3E-08
POL31	CTRG_06077	37.3166	0.0822	0.0317
CBK1	CTRG_03331	55.3991	0.0764	0
PFY1	CTRG_03999	72.2222	0.0743	0
TYE7	CTRG_03590	31.1787	0.0692	0.02891
VPS45	CTRG_05755	30.2067	0.068	0
VPH1	CTRG_03894	56.9853	0.0661	0.00057
FCP1	CTRG_05610	33.0073	0.0659	0.00035
APL2	CTRG_02422	42.5	0.0636	0
PFK2	CTRG_05115	63.4146	0.0621	0
RVS167	CTRG_05789	59.2506	0.0617	0
SRV2	CTRG_00383	39.8058	0.0607	0.00323
CUZ1	CTRG_04761	30.4498	0.0574	2.1E-05

YIH1	CTRG_00998	35.4701	0.0544	0
ARO4	CTRG_04527	66.8478	0.0503	0
KTI12	CTRG_00763	44.5993	0.0503	0.001704
GUK1	CTRG_03195	56.0166	0.05	0
PUT2	CTRG_06088	63.0952	0.0462	0
MTG1	CTRG_00762	34.2391	0.0457	0
ARO3	CTRG_01425	70.9559	0.0444	0
GNA1	CTRG_01436	49.3333	0.0438	0.045048
CCT8	CTRG_04285	64.8148	0.0383	0
DIP2	CTRG_03177	53.0612	0.0367	4.05E-06
ARG3	CTRG_05401	60.4106	0.0362	0
GCV2	CTRG_03178	63.4366	0.0358	1E-08
ERB1	CTRG_04748	63.2754	0.0351	3.7E-07
NOP58	CTRG_03008	75	0.0335	0
ARG1	CTRG_01005	74.5192	0.0327	0
RCL1	CTRG_01684	67.8378	0.0327	0
RRP12	CTRG_04882	44.8191	0.0306	0.000244
YTM1	CTRG_03409	59.0234	0.0294	0
TMA20	CTRG_04049	69.0217	0.0291	0
GRX5	CTRG_04173	70.0935	0.029	0
FOL2	CTRG_06075	63.3452	0.0282	0
NOP4	CTRG_04719	48.8742	0.0281	0
UTP21	CTRG_01392	56.29	0.0275	0.049003
TRP5	CTRG_04034	75.7489	0.0273	0
TIF6	CTRG_03113	92.6531	0.0249	0
IFM1	CTRG_04993	39.1304	0.0233	0.000269
EBP2	CTRG_01605	56.3855	0.0229	0.003723
NSA2	CTRG_02253	87.3563	0.0165	0
RPO31	CTRG_05772	77.7394	0.0161	0.00032
RET1	CTRG_00585	80.7593	0.0158	0
NOC4	CTRG_01718	39.8927	0.0158	1E-08
HGH1	CTRG_00246	59.116	0.0146	0
SAS10	CTRG_00495	44.6018	0.0134	0.001198

PPG1	CTRG_02208	47.5124	0.0565	0
SPT16	CTRG_03812	51.7034	0.0547	9.6E-06
RRD1	CTRG_03815	37.2611	0.0545	0.02281
HUB1	CTRG_02207	63.0137	0.0544	0
UFD1	CTRG_01346	54.1436	0.0535	0.00065
ARG82	CTRG_02006	41.0256	0.0513	9.7E-07
HTZ1	CTRG_00449	56.4767	0.0487	0
TLG2	CTRG_01431	33.6815	0.0486	0
SEC26	CTRG_00399	55.3459	0.0476	0.00014
SPT5	CTRG_01240	37.551	0.0454	9.6E-06
TPD3	CTRG_05670	53.3333	0.0436	0
SPC3	CTRG_03580	43.8503	0.043	0.03806
OST1	CTRG_02128	33.2627	0.0428	1E-08
SIN3	CTRG_04396	44.207	0.0419	0
RAD6	CTRG_05196	74.6753	0.0417	0
VMA7	CTRG_01568	68.8525	0.0414	0.03098
MET14	CTRG_05262	70.5	0.0391	0.01782
GDB1	CTRG_03951	55.7819	0.0389	0.00309
PSE1	CTRG_04006	49.2209	0.0384	0
PRC1	CTRG_05137	60.9259	0.0368	0
GLC3	CTRG_02108	60.7143	0.0362	6.8E-05
HCR1	CTRG_05817	48.9437	0.0361	6E-07
DTD1	CTRG_02569	54.4118	0.0359	0
SEC17	CTRG_00950	44.3686	0.0359	0.01583
CTA1	CTRG_04203	65.9794	0.0351	0
ICL1	CTRG_04702	65.4545	0.035	2E-08
SMC3	CTRG_02549	39.7318	0.0344	0
SEC14	CTRG_05264	56.25	0.0343	0
HYM1	CTRG_01576	39.1176	0.0338	0.01336
MET16	CTRG_03759	59.3023	0.0327	0
CFT1	CTRG_00150	32.7165	0.0319	0
CDC34	CTRG_05171	62.7049	0.0317	0
ACS2	CTRG_04729	67.6602	0.0296	0.04852

BUD23	CTRG_05332	69.8885	0.0121	0
MRPL23	CTRG_04455	54.5455	0.012	0
CCT2	CTRG_02077	80.6084	0.0119	0.022952
TCP1	CTRG_03169	81.0469	0.0116	0
NOP16	CTRG_01999	50.2203	0.0104	0
MET12	CTRG_02026	49.763	0.0104	0.000266
EHD3	CTRG_04796	34.3874	0.0095	6.8E-07
LTV1	CTRG_05780	44.3069	0.0087	1E-08
RNH201	CTRG_05085	35.3933	0.0087	0.006769
RPC19	CTRG_00921	58.5938	0.0078	0
MRP20	CTRG_04457	31.6151	0.0078	0.040418
BFR2	CTRG_05808	40.6844	0.0076	0
MEF2	CTRG_04186	39.604	0.0075	0.009946
PRP18	CTRG_01091	33.4586	0.0073	0.00108
KRR1	CTRG_04103	84.5494	0.0071	0
MRPS17	CTRG_00104	38.3673	0.0068	0
TAD2	CTRG_01242	32.9749	0.0066	0
DIA4	CTRG_03001	41.6847	0.0065	0
PCM1	CTRG_03651	51.4071	0.006	0
RRP36	CTRG_03137	37.1166	0.0059	0.002747
MRPS16	CTRG_04883	36.3636	0.0058	0.000763
NIT2	CTRG_05966	45.485	0.0055	2E-08
GUD1	CTRG_04898	36.5854	0.0054	0.021983
BMS1	CTRG_04024	59.315	0.0049	0
KRE33	CTRG_00388	75.048	0.0049	0
SCH9	CTRG_01449	51.0519	0.0048	0
NOP7	CTRG_01877	59.799	0.0047	0
UTP11	CTRG_00764	55.0607	0.0047	0
SPB4	CTRG_02676	51.7179	0.0045	2E-08
RIO1	CTRG_04080	49.7967	0.0045	1.4E-07
YKE2	CTRG_05895	46.9565	0.0042	0
MRPL16	CTRG_01166	55.3991	0.0042	0.004073
ARO1	CTRG_00061	60.2192	0.0041	0

DID4	CTRG_05263	61.1354	0.0295	0
HOG1	CTRG_01181	77.2152	0.0292	0
DYS1	CTRG_02401	78.5714	0.028	0
RBG2	CTRG_06086	69.837	0.0272	0
KIN28	CTRG_04213	60.5114	0.027	0
EPL1	CTRG_01074	32.3232	0.027	5.5E-06
URM1	CTRG_04125	56.4356	0.0267	0
ATG7	CTRG_00668	43.239	0.0263	0.03155
PPH3	CTRG_00717	69.8276	0.0252	0
AMD1	CTRG_01184	53.2544	0.0248	5.4E-05
CIN4	CTRG_04632	42.9348	0.0238	3.2E-05
ESS1	CTRG_04716	41.2429	0.0225	0
TPS1	CTRG_00649	74.477	0.0225	0
CDC42	CTRG_03158	86.911	0.0225	1E-08
DRN1	CTRG_04605	32.4895	0.0223	0
LIP5	CTRG_00165	72.4138	0.0206	0
CCA1	CTRG_00060	39.9274	0.0203	0
ACO2	CTRG_00642	76.9911	0.0201	0
RPT2	CTRG_06174	81.4059	0.02	0
VPS4	CTRG_05558	78.2407	0.0195	0
PRE7	CTRG_00145	77.1429	0.0191	0
PSD1	CTRG_04366	34.1503	0.0191	0.00125
IST1	CTRG_06198	42.2925	0.0187	0
RPN13	CTRG_04734	31.6129	0.0184	0.00075
VMA11	CTRG_05117	82.2086	0.018	0
RRD2	CTRG_06216	44.6602	0.0178	0
TAF10	CTRG_00972	41.0788	0.0174	0
ASF1	CTRG_03143	71.7949	0.0171	0
TRA1	CTRG_02827	50.6623	0.0169	0
KNS1	CTRG_03622	30.3077	0.0157	0.04388
DMC1	CTRG_03542	79.1855	0.0143	0
CDC28	CTRG_00650	78.8396	0.014	0
YPT7	CTRG_01883	61.1111	0.0134	0

RPC25	CTRG_04517	52.7919	0.0041	0
BNA3	CTRG_01368	53.2009	0.0039	0
NOP9	CTRG_04757	48.7943	0.0039	2.6E-07
SDO1	CTRG_00261	56.0784	0.0038	2.6E-07
SPB1	CTRG_05404	59.4937	0.0032	0
DBP8	CTRG_00592	65.9091	0.0029	0
RPA12	CTRG_01727	73.9837	0.0022	0
FCF1	CTRG_03183	76.5306	0.0013	0

SMC1	CTRG_00730	37.1109	0.0133	8.6E-07
RFC5	CTRG_01161	53.0387	0.0121	0
GOS1	CTRG_05064	41.629	0.0118	3.9E-07
ARP8	CTRG_04468	32.2508	0.0107	1.2E-05
UBP15	CTRG_02092	32.7447	0.0104	0
PRS3	CTRG_01399	86.25	0.0098	0
DNA2	CTRG_03009	30.3521	0.0092	0
RQC1	CTRG_01694	30.057	0.0092	0
MNL1	CTRG_01440	31.3415	0.0091	0.00951
CGI121	CTRG_05279	30.4348	0.0089	0
HAT1	CTRG_04691	37.8897	0.0088	1E-08
YCH1	CTRG_00087	36	0.0088	0.01388
CTK1	CTRG_02406	36.2429	0.0086	0
APL4	CTRG_00816	36.0339	0.0083	0
CDC45	CTRG_02651	38.9982	0.0083	0
AIP1	CTRG_00746	36.5854	0.0081	0
PHO81	CTRG_01018	30.5601	0.0081	0
SAP1	CTRG_05876	39.3899	0.0081	0
TAF2	CTRG_05759	30.8954	0.0081	0.00025
RHO4	CTRG_04441	31.6109	0.008	2E-08
PNG1	CTRG_01802	33.1507	0.0078	0.01106
PHO23	CTRG_02063	30.6931	0.0077	4.7E-05
GYP7	CTRG_05146	36.828	0.0076	0.00043
GLY1	CTRG_00533	58.1522	0.0075	0
BZZ1	CTRG_02164	36.4078	0.0074	0
LSB6	CTRG_04279	30.8886	0.0074	0.00221
SOH1	CTRG_05743	33.3333	0.0073	2.8E-06
HSE1	CTRG_00967	33.6798	0.0072	0
VPS55	CTRG_00787	47.2222	0.0071	5.8E-07
TAF1	CTRG_03966	30.2415	0.007	0
PMS1	CTRG_02080	34.981	0.0069	4.9E-07
WHI2	CTRG_04873	43.6548	0.0068	2.9E-06
BCY1	CTRG_01300	47.7974	0.0067	0

GTR2	CTRG_00782	59.7403	0.0067	0
SLN1	CTRG_00987	38.5358	0.0067	0
NUP57	CTRG_00837	33.3333	0.0067	0.00095
RET2	CTRG_05521	37.9433	0.0066	0
RKM4	CTRG_02707	36.1446	0.0066	0.00013
TFB4	CTRG_02929	36.3144	0.0065	3.1E-07
TIP41	CTRG_06028	36.342	0.0064	2.3E-06
SEA4	CTRG_02300	38.4152	0.0064	0.0269
SED5	CTRG_04361	46.4497	0.0062	0
UBX5	CTRG_04161	37.2047	0.0062	0.00055
TOP3	CTRG_02493	50.2423	0.006	0
UBA2	CTRG_03998	42.3015	0.006	2.5E-06
ALG7	CTRG_01670	49.7976	0.0059	0
APC11	CTRG_05784	39.2593	0.0059	0
MRE11	CTRG_04860	36.351	0.0058	0.00014
YTA7	CTRG_06068	38.1764	0.0058	0.03421
PRI1	CTRG_06141	41.3187	0.0057	0
SLY1	CTRG_03772	49.6075	0.0055	0
STV1	CTRG_03012	43.9701	0.0054	0
YKT6	CTRG_04319	70.5	0.0054	0
IPL1	CTRG_02814	36.0277	0.0054	3.5E-05
EMP24	CTRG_00421	53.202	0.0053	0
HOS2	CTRG_05503	65.1885	0.0053	0
MSH6	CTRG_03593	48.6088	0.0053	0.02506
RVS161	CTRG_04953	68.4211	0.0052	2E-08
SKI2	CTRG_03099	51.243	0.0051	1.4E-06
SEC27	CTRG_02922	48.4783	0.0051	6.1E-05
MAD2	CTRG_04753	42.7907	0.005	0.00193
RPN6	CTRG_00321	56.7376	0.005	0.00196
GCN3	CTRG_04854	59.6825	0.0049	0
TEM1	CTRG_04343	56.4706	0.0049	0
ADA2	CTRG_04073	51.0112	0.0049	4.6E-05
UBC12	CTRG_06192	43.9153	0.0048	0

RAD14	CTRG_05009	42.4658	0.0048	3E-08
RRP40	CTRG_04417	40.3704	0.0048	8E-08
CNS1	CTRG_04372	50.3817	0.0048	0.00296
PMT4	CTRG_01500	51.6469	0.0048	0.00895
ATP17	CTRG_01055	59.4937	0.0048	0.01822
CNB1	CTRG_06124	64.7399	0.0047	0
MAS2	CTRG_05290	49.0196	0.0047	0.00295
TAF6	CTRG_05699	43.3925	0.0046	0
UBC8	CTRG_04589	53.125	0.0046	0
PXR1	CTRG_03943	52.0548	0.0046	0.00105
VMA4	CTRG_00878	57.5221	0.0045	0
RFC1	CTRG_01529	51.9424	0.0044	0
TAZ1	CTRG_01874	49.2857	0.0044	0.00759
PRI2	CTRG_03811	50.2982	0.0043	0
PSF1	CTRG_04863	37.4172	0.0043	0
APS2	CTRG_03467	60.6897	0.0043	0.01231
SMC2	CTRG_01853	54.5687	0.0041	0
TOA2	CTRG_00204	32.0755	0.0039	0
RHO2	CTRG_01714	64.1711	0.0039	3E-08
BNA5	CTRG_03162	54.6638	0.0038	0
RSR1	CTRG_00897	54.4715	0.0038	0
DID2	CTRG_05973	58.5	0.0038	1E-08
VTC4	CTRG_00507	60.9958	0.0037	0
GPN2	CTRG_05322	61.0795	0.0035	0
HAP3	CTRG_00393	33.8983	0.0035	0
CDC31	CTRG_00365	41.8605	0.0033	0
GTR1	CTRG_01857	61.0619	0.0033	0
SKI6	CTRG_00132	59.5833	0.0032	0
RAD16	CTRG_04680	61.1696	0.003	0
YAH1	CTRG_00466	63.8037	0.0029	0
GPA1	CTRG_06128	63.9344	0.0027	0.00347
ESA1	CTRG_06195	63.6888	0.0021	0
SUB2	CTRG_00129	82.069	0.0014	0

Candida auris									
**Meiosis Initiation**					**Meiosis commitment**				
**S.cerevisae gene name**	**C. auris gene stable ID**	**% of S.cerevisae gene identical to target C. auris gene**	**dN/dS**	**MEGA P value**	**S.cerevisae gene name**	**C. auris gene stable ID**	**% of S.cerevisae gene identical to target C. auris gene**	**dN/dS**	**MEGA P value**
PMI40	B9J08_002392	62.4146	0.0468	0	QCR10	B9J08_001820	30.2632	0.2618	0.00073
ARG1	B9J08_000686	74.7596	0.0453	0	APC11	B9J08_004121	40.5797	0.0902	4E-06
TMA20	B9J08_000908	64.0884	0.0473	0	PSF2	B9J08_003880	41.9162	0.0827	0
PUT2	B9J08_004317	60.9966	0.0356	0	AIM7	B9J08_001869	44.1176	0.0783	0
FCF1	B9J08_000673	78.0749	0.0362	0	ESS1	B9J08_000025	34.375	0.0765	2E-08
GLN1	B9J08_004561	76.9648	0.0356	0	TLG2	B9J08_002179	36.9748	0.0742	0.00033
RPP2B	B9J08_003756	72.0721	0.0208	0	SOD1	B9J08_001381	71.4286	0.0663	0
TUF1	B9J08_003238	76.2791	0.0495	0	QCR8	B9J08_003126	62.766	0.0654	0
MTG1	B9J08_002983	36.5123	0.0339	0	RUB1	B9J08_000165	59.7403	0.0548	0
TCP1	B9J08_000264	81.0469	0.0133	0.01739	ATP17	B9J08_002610	57	0.0484	0
RPC25	B9J08_001337	53.5714	0.0131	0	ATP19	B9J08_003926	42.8571	0.0455	0
MRPS17	B9J08_003530	39.3443	0.0074	0	UFD1	B9J08_003077	54.3909	0.0401	0
BUD23	B9J08_002808	68.7732	0.0073	0	HTA2	B9J08_005144	88.4615	0.0396	0
KTI12	B9J08_002982	45.1049	0.0071	0	MET14	B9J08_002995	74.8744	0.0387	0
RML2	B9J08_004006	51.1299	0.0068	0	MET17	B9J08_003514	69.4323	0.0376	0
DBP9	B9J08_005373	58.4211	0.0051	0	CTA1	B9J08_002298	66.0494	0.0373	0
MRPL23	B9J08_001046	55.7471	0.0043	0.00135	ATG8	B9J08_005081	73.8095	0.0368	0
RPC19	B9J08_004169	57.1429	0.0041	0	SKI6	B9J08_001396	56.9106	0.0324	0
CCT5	B9J08_001136	73.9526	0.0028	3.8E-06	ICL1	B9J08_003374	63.7681	0.0295	1E-05
NHP2	B9J08_004689	75	0.0027	0	CDC34	B9J08_001564	64.7059	0.0261	0
TIF6	B9J08_000475	92.2449	0.0011	0	COF1	B9J08_001311	83.9161	0.0259	0
					HTZ1	B9J08_003996	81.8182	0.0257	0

SAH1	B9J08_004352	82.6281	0.019	0
RPT2	B9J08_001085	82.7273	0.0172	0
PRE7	B9J08_001406	79.2531	0.0172	9.4E-06
SUB2	B9J08_003512	78.7529	0.0167	0
ASF1	B9J08_002085	71.6667	0.0143	0
GLC7	B9J08_000462	85.3659	0.0127	0
RRP40	B9J08_003406	43.1818	0.0077	0
STV1	B9J08_001677	43.8743	0.0064	2.4E-06
VMA9	B9J08_001360	60	0.0059	5.5E-05
TOA2	B9J08_005236	52.8926	0.0041	0
VMA7	B9J08_000867	65.3226	0.0037	0.00446
SOD2	B9J08_000528	65.2174	0.0032	0
DPH1	B9J08_003177	70.1235	0.003	0
PRS3	B9J08_001772	86.875	0.0013	0
CDC42	B9J08_000269	88.4817	0.001	0
